# Weight and Glucose Reduction Observed with a Combination of Nutritional Agents in Rodent Models Does Not Translate to Humans in a Randomized Clinical Trial with Healthy Volunteers and Subjects with Type 2 Diabetes

**DOI:** 10.1371/journal.pone.0153151

**Published:** 2016-04-19

**Authors:** Rebecca J. Hodge, Mark A. Paulik, Ann Walker, Joyce A. Boucheron, Susan L. McMullen, Dawn S. Gillmor, Derek J. Nunez

**Affiliations:** 1 Discovery Medicine, Metabolic Pathways Cardiovascular Unit, GlaxoSmithKline, Research Triangle Park, North Carolina, United States of America; 2 Biology, Metabolic Pathways Cardiovascular Unit, GlaxoSmithKline, Research Triangle Park, North Carolina, United States of America; 3 Quantitative Sciences - Clinical Statistics, R&D Projects Clinical Platforms and Sciences, GlaxoSmithKline, Research Triangle Park, North Carolina, United States of America; 4 Clinical Pharmacology Science and Study Operations, GlaxoSmithKline, Research Triangle Park, North Carolina, United States of America; Florida International University Herbert Wertheim College of Medicine, UNITED STATES

## Abstract

**Background:**

Nutritional agents have modest efficacy in reducing weight and blood glucose in animal models and humans, but combinations are less well characterized. GSK2890457 (GSK457) is a combination of 4 nutritional agents, discovered by the systematic assessment of 16 potential components using the diet-induced obese mouse model, which was subsequently evaluated in a human study.

**Nonclinical Results:**

In the diet-induced obese mouse model, GSK457 (15% w/w in chow) given with a long-acting glucagon-like peptide -1 receptor agonist, exendin-4 AlbudAb, produced weight loss of 30.8% after 28 days of treatment. In *db/db* mice, a model of diabetes, GSK457 (10% w/w) combined with the exendin-4 AlbudAb reduced glucose by 217 mg/dL and HbA1c by 1.2% after 14 days.

**Clinical Results:**

GSK457 was evaluated in a 6 week randomized, placebo-controlled study that enrolled healthy subjects and subjects with type 2 diabetes to investigate changes in weight and glucose. In healthy subjects, GSK457 well tolerated when titrated up to 40 g/day, and it reduced systemic exposure of metformin by ~ 30%. In subjects with diabetes taking liraglutide 1.8 mg/day, GSK457 did not reduce weight, but it slightly decreased mean glucose by 0.356 mmol/L (95% CI: -1.409, 0.698) and HbAlc by 0.065% (95% CI: -0.495, 0.365), compared to placebo. In subjects with diabetes taking metformin, weight increased in the GSK457-treated group [adjusted mean % increase from baseline: 1.26% (95% CI: -0.24, 2.75)], and mean glucose and HbA1c were decreased slightly compared to placebo [adjusted mean glucose change from baseline: -1.22 mmol/L (95% CI: -2.45, 0.01); adjusted mean HbA1c change from baseline: -0.219% (95% CI: -0.910, 0.472)].

**Conclusions:**

Our data demonstrate remarkable effects of GSK457 in rodent models of obesity and diabetes, but a marked lack of translation to humans. Caution should be exercised with nutritional agents when predicting human efficacy from rodent models of obesity and diabetes.

**Trial Registration:**

ClinicalTrials.gov NCT01725126

## Introduction

Between 1980 and 2013, the number of overweight and obese people worldwide increased from 857 million to 2.1 billion, and no countries showed a decrease in obesity levels during this timeframe [1]. Excess weight is associated with a range of health risks, including type 2 diabetes (T2D), hypertension, cardiovascular disease, sleep apnea, joint pain, cancer and impaired fertility [2]. The need for safe and effective therapies to promote weight loss is high.

Obesity and associated metabolic diseases can be impacted by the interplay between specific nutrients, the composition of the gut microbiome, and gut peptide release [3]. Obesity is associated with changes in the gut microbiome [4, 5], and modulation of the gut microbiota can be effective in reducing metabolic endotoxemia and inflammation [6, 7]. Gut peptide release is stimulated by specific nutrients [8], as well as by metabolic by-products of the microbiota such as short chain fatty acid [9,10]. These gut peptides, such as glucagon-like peptide 1 (GLP-1), peptide tyrosine-tyrosine (PYY), glucagon-like peptide-2 (GLP-2), and oxyntomodulin, have been shown to have direct effects on food intake, insulin secretion, glucose control, gut barrier integrity, and energy expenditure [11, 12]. Several exogenous GLP-1 receptor (GLP-1R) agonists have been approved as therapies for T2D, including exenatide, liraglutide, dulaglutide, and albiglutide [13]; liraglutide has also been approved for the treatment of obesity [14]. Investigational work into the potential therapeutic benefits of other peptides is ongoing [15].

The intersection of these research areas relating to gut peptides and nutrients led to a key question: Would nutritional agents that stimulate gut peptide release combined with a GLP-1R agonist or metformin, which itself stimulates endogenous release of GLP-1 [16], enhance weight loss and improve glucose metabolism? GSK2890457 (GSK457) was developed as a mixture of nutritional agents to address this question.

The components of GSK457 were identified based on extensive preliminary nonclinical work using the high-fat fed diet-induced obese (DIO) mouse model. Sixteen nutritional agents were screened initially alone and in combination with a long-acting GLP-1 receptor agonist, referred to here as an exendin-4 AlbudAb. Preference was given to agents that (i) are generally recognized as safe (GRAS status), (ii) have no known safety issues, and (iii) stimulate gut peptide release in nonclinical and/or clinical studies. The single nutritional agents, as well as dual, triple and quadruple combinations were tested in the DIO mouse with weight loss as the primary endpoint.

GSK457 is a mixture of four ingredients: oligofructosaccharide (OFS), apple pectin, blackcurrant extract (BCE), and oleic acid in a ratio of 5:5:2:3. Each of these GRAS components is found in small amounts in a normal diet and they are used as food ingredients. (See S1 Materials and Methods for further information.)

The objective of our studies was to investigate the effects of GSK457 on weight and glucose metabolism in mouse models and humans. Here we report the remarkable weight loss and glucose lowering effects of GSK457 when administered alone and in combination with the exendin-4 AlbudAb in the DIO and *db/db* mouse models, respectively. While rodent models are frequently used to predict the effect of nutritional modulation in humans, here we report the lack of clinical translation when GSK457 was administered in combination with liraglutide or metformin to subjects with T2D.

**Fig 1:**
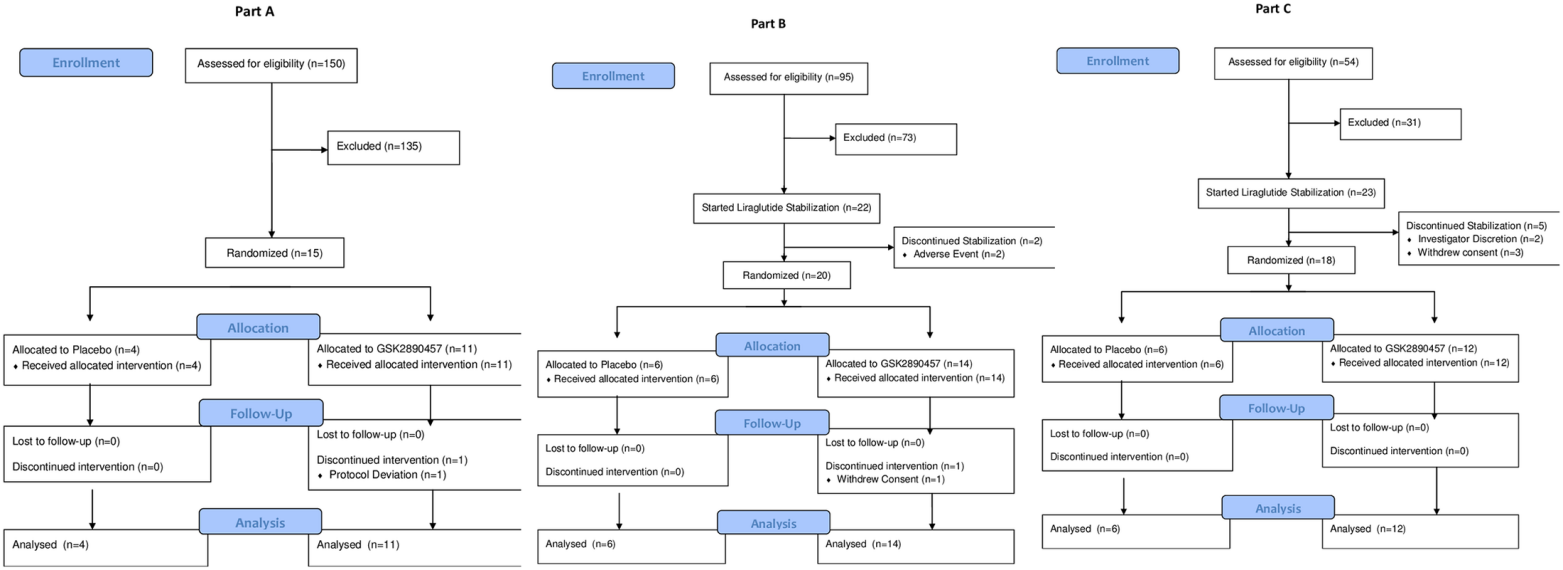
CONSORT flow diagram.

Fig 1 shows the CONSORT flow diagram for the study.

## Materials and Methods

### Nonclinical Materials and Methods

*In vivo* studies were conducted to investigate the effects of GSK457 alone and in combination with a long-acting GLP-1R agonist using the DIO C57BL6NTac mouse model of obesity and the *db/db* mouse model of T2D.

In the nonclinical studies, the GLP-1 receptor agonist co-administered with GSK457 was an exendin-4 AlbudAb. This recombinant fusion protein consists of exendin-4 (exenatide) genetically fused to an AlbudAb, a domain antibody consisting of a small (~14 kDa) human antibody light chain variable domain that binds to serum albumin to significantly increase half-life following subcutaneous injection. In rodents, the AlbudAbs have half-lives of ~ 20–40 hours, much longer than the 4 to 6 hour half-life of exendin-4. AlbudAb is a trademark of the GlaxoSmithKline group of companies.

#### Nonclinical ethics statement

Animals were housed and maintained in an AAALAC, international accredited care and use program. All procedures were performed in compliance with the Animal Welfare Act, USDA regulations and approved by the GlaxoSmithKline Institutional Animal Care and Use Committee under protocol NC0176.

#### Nonclinical study designs

**Fig 2:**
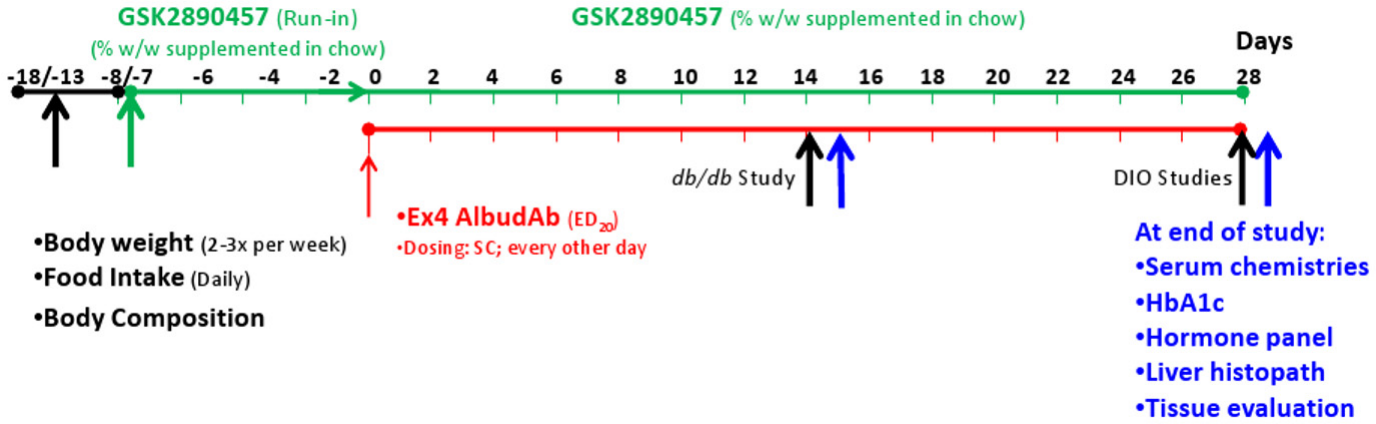
General design of chronic weight loss studies. Baseline body weight and body composition measurements were made during the period Day -18 to -13 (left Black Arrow). Treatment with GSK457 was begun on Day -8 or -7 (Green Arrow). Subcutaneous exendin-4 AlbudAb dosing began on Day 0 (Red Arrow) and ended on Day 28 for the DIO study (right Black Arrow) and on Day 14 for the *db/db* study (middle Black Arrow); final body composition measurements were also made on that day. Blood was collected on the following day for serum chemistry, HbA1c and hormone analyses, and tissues were collected for histopathology (Blue Arrows).

Male DIO C57BL/6 mice, *db/db* (B6.Cg-m +/+ Lepr ^db^/J) mice and age-matched lean controls were used in these experiments. Fig 2 illustrates the general study designs. The first body weight and body composition measurements were used to randomize the animals to ensure similar baseline weights and percent body fat in each group.

The lean C57BL/6 control mice were fed Lab Diet 5001 (PMI Nutrition International, Brentwood, MO, USA) and *db/db* mice and their controls were fed 5K67 chow (LabDiet, St. Louis, MO, USA). The DIO mice were maintained on Research Diets (New Brunswick, NJ, USA) D12451 high-fat chow to obtain a stable obese weight prior to study-start. All the chows (except for lean controls) were augmented with 25% Nutella^®^ (w/w) as a masking/palatability agent for 1 to 2 weeks prior to incorporating GSK457. At randomization, GSK457 was added to the Nutella^®^-containing high fat chow, and this diet was maintained for the duration of the studies. The exendin-4 AlbudAb was dosed subcutaneously every other day beginning 7–8 days after starting GSK457. Fourteen (*db/db* study) or 28 (DIO study) days after the start of exendin-4 AlbudAb, animals were fasted for at least 4 hours before blood was collected under isoflurane anesthesia. HbA1c, plasma gastrointestinal hormone levels and serum clinical chemistry parameters were measured. Major organs and tissues were collected for macroscopic and microscopic histological examination at termination of the study.

Details relating to animal management, preparation of drug treatments, measurements of body weight, body composition and food consumption, and methods of clinical chemistry, hormone and histopathology analyses are included in S2 Materials and Methods.

#### Nonclinical data analyses

All of the data are presented as mean ± SEM where the experimental unit is an individual animal. The data were analyzed with JMP (SAS Institute, Cary, NC, USA), Prism (GraphPad Software, Inc., La Jolla, CA, USA) or Microsoft Excel (Redmond, WA, USA) software. A one-way ANOVA with post-hoc t-test was used to compare all pairs of treatments. All hormone data and non-normally distributed clinical chemistry parameter data were log-transformed prior to statistical analysis. Significance tests were performed using a 2-sided hypothesis at the 0.05 level. A t-test was used to compare the average response of animals on the GSK457 + exendin-4 AlbudAb combination therapy to the average responses of the animals on GSK457 alone and exendin-4 AlbudAb alone to determine whether the combination had a greater than additive effect.

### Clinical Materials and Methods

This study was conducted between 10 February 2013 and 12 September 2013.

#### Clinical ethics statement

This study (SMP116623; www.clinicaltrials.gov NCT01725126) enrolled healthy subjects and subjects with T2D in accordance with ICH Good Clinical Practice guidelines [17], subject privacy requirements, and the principles of the Declaration of Helsinki [18]. One site participated in Part A (Quintiles Early Clinical Development, Overland Park, KS, USA), and two sites participated in Parts B and C (Elite Research Institute, Miami, FL, USA; Profil Institute for Clinical Research, Inc. Chula Vista, CA, USA). The study protocol was approved by Schulman Associates Institutional Review Board (formerly Independent Institutional Review Board; Sunrise, FL, USA). All subjects provided written informed consent before enrollment.

#### Clinical study design

The protocol for this trial and the supporting CONSORT checklist are available as supporting information; see S1 CONSORT Checklist and S1 Protocol.

This study was conducted in three parts. All three parts were parallel group, double-blind (sponsor unblind), randomized, and placebo-controlled.

**Fig 3:**
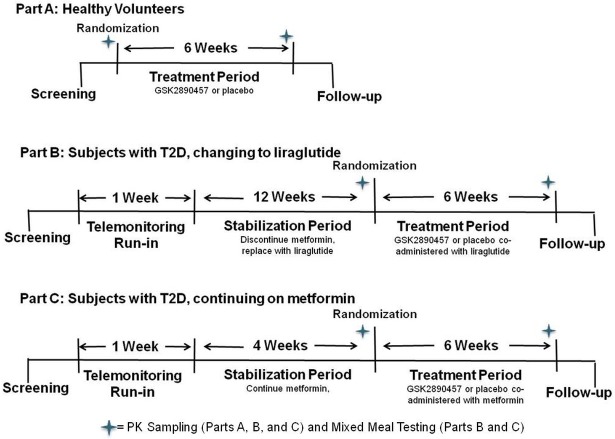
Clinical Study Design Schematic.

Part A enrolled healthy subjects. Subjects who passed the Screening procedures were randomized to receive GSK457 or placebo (5:2 allocation, respectively) for 6 weeks to explore safety and tolerability (Fig 3). A single dose of 500mg IR metformin was given just before breakfast on Day 1 and on Day 42 to evaluate the effect of GSK457 on metformin pharmacokinetics. On Day 42, the subjects took metformin followed by GSK457 or placebo, and then breakfast. The subjects were given standardized meals on these days. On Day 1 (the time of randomization), subjects started GSK457 or placebo under the supervision of an unblinded site staff member at dinner, and received study medication for home administration.

Part B enrolled subjects with T2D who were stable on metformin (Fig 3). Subjects who fulfilled the Screening requirements began a 1-week Run-in “procedures familiarization” Period during which they remained on their usual dose of metformin. At this time, the subjects became familiar with a wireless-enabled glucometer and a weighing scale (AMC Health, New York, NY, USA). The Run-in Period was followed by a 12-week Stabilization Period, during which the subjects discontinued their usual metformin therapy and commenced subcutaneous injections of liraglutide once daily, titrated to a 1.8 mg dose per label directions. After this, the subjects were randomized to a 6-week Treatment Period when they took liraglutide and either GSK457 or placebo (2:1 allocation, respectively). Liraglutide concentrations were measured on Day 1 and Day 42 of the Treatment Period. Subjects returned to their usual metformin dosing regimen on Day 43. The metformin-liraglutide substitution design was employed because a pre-study feasibility assessment had indicated that it would not be possible to enroll sufficient subjects taking liraglutide as their usual T2D medication.

Part C enrolled subjects with T2D who were stable on metformin (Fig 3). Subjects who fulfilled the Screening requirements began a 1-week Run-in “procedures familiarization” Period as in Part B during which they remained on their usual dose of metformin. The Run-in Period was followed by a 4-week Stabilization Period on their usual twice daily metformin therapy, and then the subjects were randomized to a 6-week Treatment Period during which they remained on metformin and received either GSK457 or placebo (2:1 allocation, respectively). Metformin concentrations were measured on Day 1 and Day 42 of the Treatment Period. Metformin pharmacokinetic data from Part A indicated a reduction in metformin area under the curve (AUC) when it was administered at the same time as GSK457. As a result, the protocol was amended, and subjects in Part C were instructed to take each metformin dose 1 hour prior to GSK457.

In all three Parts, to reduce the risk of gastrointestinal adverse events subjects started by taking 15 g of GSK457 daily, in 2 divided doses, and were up−titrated to 30g daily (Day 4) and then to a maximum dose of 40 g daily at Day 7. Subjects unable to tolerate doses of 30 g or 40 g were permitted to reduce their dose to tolerated levels, with a minimum tolerated dose of 15 g required to remain in the study. A unit dose of 5 g of GSK457 consisted of three capsules of oleic acid and a separate sachet of powder for each of the three powder compounds. The powders were mixed by the subject into 12 oz of flavored (Mio^™^ pomegranate/berry flavoring) water in a shaker bottle and consumed just prior to breakfast and dinner. The capsules could be taken with this drink or separately with water. The matching placebo consisted of starch and microcrystalline cellulose in identical sachets and capsules.

In Parts B and C, there were additional clinic visits on Days 7, 14, and 28 for safety evaluations, potential modification of dose (Days 7 and 14), and dispensing of study medications. Subjects monitored their capillary blood glucose levels twice a day using a wireless-enabled glucometer and daily weights were monitored using a wireless enabled weighing instrument (see S3 Materials and Methods for more information). Subjects were fed standardized meals (~2300 kcal; ~60% carbohydrate, 20% fat, and 20% protein) on the pharmacodynamic profiling days.

In Parts A-C, there was a follow−up visit approximately 14 days after discharge from the unit on Day 43.

Investigators, subjects and staff (except for the unblinded pharmacist) were blinded to the allocation of study treatment. The GlaxoSmithKline clinical study team had unblinded access to the data during the course of the study. When in the clinic, subjects were dosed separately from each other by a staff member to minimize the potential for unblinding. The staff member who helped prepare the drinks did not participate in any other study procedures.

Key inclusion/exclusion criteria and study procedures are summarized in Table 1 and the endpoints and procedures are shown in Table 2.

**Table 1:** Clinical Study Design and Main Inclusion-Exclusion Criteria.

	Part A	Part B	Part C
**Population**	Healthy Subjects	Subjects with T2D, on a stable metformin dose of ≥ 850 mg daily	Subjects with T2D, on a stable metformin dose of ≥ 850mg daily
**Run-in**	n/a	One week to familiarize with the wireless-enabled glucometer and weighing scale procedures	
**Stabilization**	n/a	Discontinue metformin, replace with liraglutide for 12 weeks, titrated to 1.8 mg daily.	Continue usual twice daily metformin dose for 4 weeks.
**Treatment**	6 weeks GSK457 or placebo	6 weeks of liraglutide, plus GSK457 or placebo	6 weeks of metformin, plus GSK457 or placebo
**Key Inclusion**	Male or female subjects of non-child-bearing potential, age 18–70, BMI 18–35 kg/m^2^, inclusive. No significant known medical conditions.	Male or female subjects of non-child-bearing potential, age 18–70, BMI 18–35 kg/m^2^, inclusive. No significant known medical conditions except for T2D. Diagnosis of T2D for at least 3 months. HbA1c of 7.5 to 11, inclusive. C-peptide >1 ng/mL. Urine albumin excretion <30mg/g creatinine. Calcitonin <50 pg/mL (Part B only).	
**Key Exclusion**	Use of medications within 14 days of treatment period that might have had the potential to interact with GSK457, including weight-loss products, oral antibiotics, bile acid sequestrants, protein-pump inhibitors, H2 antagonists, probiotics, herbal and nutraceutical products intended to impact gut health and use of stomach ‘coating’ agents. No contraindications per the label for liraglutide (Part B) or metformin (Parts A and C)		

**Table 2:** Endpoints and Associated Procedures.

**Primary Endpoints**	**Associated Procedures**		
	**Part A**	**Part B**	**Part C**
**Safety and Tolerability**	Subject safety was monitored by evaluating Adverse Events including hypoglycemic events, Hematology, Chemistry, Urinalysis, ECGs, and Vital Signs (in all Parts); Columbia Suicide Severity Rating Scale (Part B only)		
	Subject-completed Gastrointestinal Symptoms Rating Scale (GSRS) [19]on Days 1,7, 14, and 42	Subject-completed GSRS at Weeks 1 and 7 of Stabilization (Part B only) and on Days -2, 7, 14, 28, and 41 (Parts B and C).	
	In Part A, adverse events were collected from the start of dosing until the Follow-up visit. In Parts B and C, adverse events were collected from the start of the Stabilization Period until the Follow-up visit. Adverse events were graded as mild (Grade 1), moderate (Grade 2), severe (Grade 3), life-threatening (Grade 4) or death (Grade 5)		
**Body Weight** Change and % change from Baseline to End of Treatment	n/a	Weight was measured at each clinic visit. In addition, subjects were provided with a weighing scale that transmitted daily weight data to a central database (AMC Health). Each investigator was able to review weight data in real time for subjects enrolled at their site.	
**Glucose and Insulin** Changes in weighted mean AUC (0–4 hours, 0–24 hours [glucose only]); Fasting, HOMA-IR, and Matsuda index measures of insulin sensitivity and HbA1c.	n/a	A standardized meal was eaten at T = 0 on Days -1 and 42. Blood samples were collected at hours 0, 0.5, 1, 1.5, 2, 4, 5.5, 10, 11.5, 14, 24.for measurement of glucose and insulin. HbA1c was measured on Days -1 and 42. In addition, subjects were provided with a glucometer, strips and a modem that transmitted glucose data twice-daily to a central database (AMC Health). Each investigator was able to review glucose data in real time for subjects enrolled at their site.	
**Secondary Endpoints**	**Part A**	**Part B**	**Part C**
**Pharmacokinetics**	PK of single doses of metformin evaluated on Day 1 and Day 42. Blood samples were collected at hours 0, 0.25, 0.5, 1, 2, 4, 5.5, 8, 10. T = 0 was just prior to dosing.	PK of liraglutide measured on Days -1 and 42. Blood samples were collected at hours 0, 0.25, 0.5, 1, 2, 4, 5.5, 8, 10, 11.5, 24. T = 0 was just prior to dosing.	PK of metformin measured on Days -1 and 42. Blood samples were collected at hours 0, 0.25, 0.5, 1, 2, 4, 5.5, 8, 10, 11.5, 24. T = 0 was just prior to dosing.
	AUCss, Cmax, tmax, on Day 42 as compared to Day -1 (Day 1 in Part A).		

#### Clinical assays

Venous blood samples were collected for PD and PK analysis in K^+^ EDTA tubes and rapidly placed on ice until centrifuged at 4°C for 10 minutes. Plasma was stored at -70°C until analyzed. Samples for clinical chemistry and hematology were measured by the local certified laboratory. Plasma samples were analyzed for glucose, insulin and leptin by BioAgilytix Labs (Durham, NC, USA). Glucose was measured using a Glucose 2300 STAT Plus Glucose Analyzer with the glucose/lactate standard, catalog number 2747 (Yellow Springs Instrument Co, OH, USA). Insulin was measured using a multiplex assay; catalog number K151BZC from MesoScale Discovery (Gaithersburg, MD, USA), and leptin was measured using the human leptin assay kit, number K151BYC, from Millipore (Billerica, MA, USA).

The plasma concentrations of liraglutide and metformin were analyzed by PPD, Inc. (Richmond, VA, USA) using validated analytical methods. (See S3 Materials and Methods for additional information.)

#### Clinical study statistical methods

The sample size for each part of the study was based on feasibility and no formal hypothesis testing was planned. An estimation approach was used where point estimates for treatment differences and corresponding confidence intervals were determined. The central randomizations and treatment assignments were generated by GlaxoSmithKline using validated internal software. Analyses were performed using SAS, version 9.1 (SAS Institute, Cary, NC, USA). Descriptive statistics and individual subject data were reviewed to evaluate safety. In Parts B and C change from baseline and % change from baseline body weight were analyzed using repeated measures analysis of covariance models (ANCOVA) with effects for treatment, day, treatment x day, and baseline weight. Change from baseline 24-hour weighted mean glucose AUC was analyzed using an ANCOVA model with effects for treatment and baseline covariate. Similar analyses were done for other glycemic endpoints including glucose weighted mean AUCs over 4 hours, fasting plasma glucose (FPG), and HbA1c. Results are presented as differences in least squares means and 95% confidence intervals (CI).

To assess a potential pharmacokinetic interaction due to co-administration of metformin or liraglutide with GSK457, the AUC and Cmax of metformin (Part A) and liraglutide (dose-normalized, Part B) were separately analyzed following log_e_-transformation using mixed effects models with a fixed effect term for day and a random effect for subject. The point estimates for the difference in least squares means and associated 90% confidence intervals were back-transformed to provide point estimates and 90% confidence intervals for the ratios, [Day 42 (co-administration) to Day 1 (metformin or liraglutide alone)].

## Results and Discussion

### Nonclinical Results

#### Chronic 28-day treatment of DIO mice with 15% (w/w) GSK457 in combination with exendin-4 AlbudAb: reductions in body weight, fat mass and food intake

**Fig 4:**
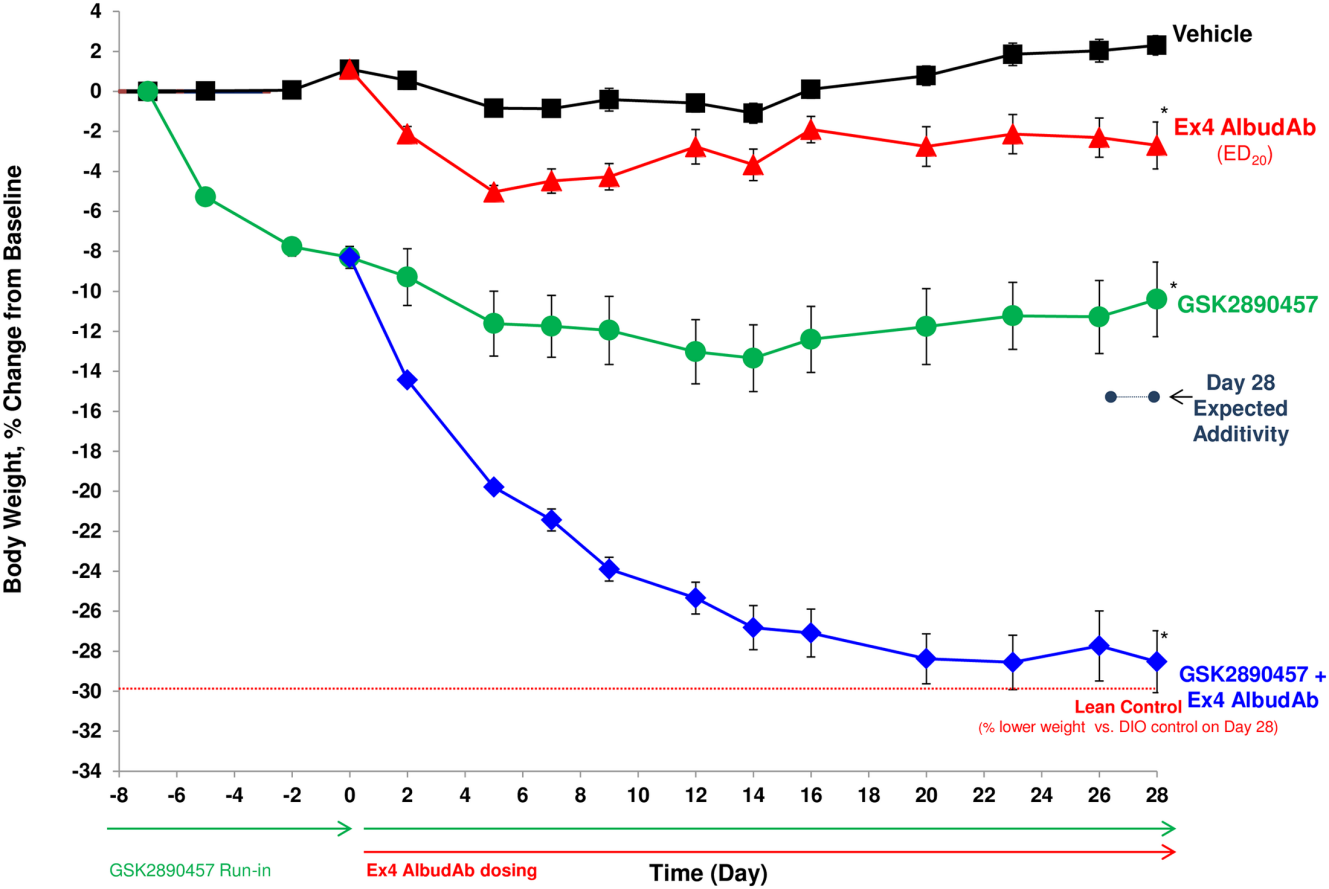
GSK457 + exendin-4 AlbudAb combination treatment returned DIO C57BL/6NTac mice to the body weight of age-matched lean control mice after 28 days. GSK457 administration began on Day -7 (baseline) and exendin-4 AlbudAb SC dosing began on Day 0. An asterisk (*) indicates a significant difference from vehicle (p < 0.05). The small dots/dashed line indicates the additive weight loss.

In the DIO mouse, GSK457 treatment alone produced a vehicle-subtracted weight loss of 12.7% (p<0.05). DIO mice treated with the exendin-4 AlbudAb (ED_20_ dose for weight loss = 0.03 mg/kg) had a vehicle-subtracted weight loss of 5.0% (p<0.05). Pre-dosing GSK457 for a week followed by a combination of GSK457 and the exendin-4 AlbudAb for 28 days resulted in a vehicle-subtracted weight loss of 30.8%, far exceeding the sum of the exendin-4 AlbudAb and GSK457 effects, p < 0.001 (Fig 4). The final body weight (32 ± 0.8 g) of the GSK457 + exendin-4 AlbudAb combination group was not different from the final body weight of the lean control group (31.7 ± 0.6 g) (p = 0.68), reflecting a complete reversal of the diet induced obesity.

There was a significant reduction in the absolute body fat mass in the GSK457 + exendin-4 AlbudAb combination group compared to vehicle (p < 0.05) (S1 Fig), and the percent body fat mass decreased from 40.4% to 24.2% (p < 0.05).

In addition, a 38% inhibition of cumulative food intake was observed in the GSK457 + exendin-4 AlbudAb combination group. There appears to be more than additive efficacy because the individual agents produced much smaller effects on food intake (p < 0.001) (S2 Fig).

Animals treated with the GSK457 + exendin-4 AlbudAb combination exhibited similar activity and behavioural patterns as the lean control animals.

The GSK457 + exendin-4 AlbudAb combination group displayed many significant changes in serum chemistries, all of which reflect the transition from the pathological state of obesity to a more normal lean state. For example, treatment with this combination reduced serum glucose levels by 22% to the level of lean controls (190 and 192 mg/dL, respectively). The liver enzymes aspartate aminotransferase (AST) and alanine transaminase (ALT) were elevated in the vehicle-treated control DIO mice due to the presence of diet-induced hepatic steatosis. Treatment with the GSK457 + exendin-4 AlbudAb combination decreased levels by 87% and 73%, respectively, comparable to those of the lean controls, and ameliorated the hepatic steatosis observed histologically. This was accompanied by a marked reduction of circulating triglycerides and total cholesterol. Overall, the serum chemistries in the DIO combination treated group closely resembled the lean control chemistries, indicating an improvement in the metabolic state in this DIO mouse model. Additional results relating to serum chemistry and hormone panel parameters, and changes in liver steatosis can be found in the S1 Nonclinical Results, S3 and S4 Figs and S1 and S2 Tables.

Similar results were obtained for body weight and composition, food intake and serum chemistry changes when liraglutide was utilized in place of the exendin-4 AlbudAb.

#### Chronic 14-day treatment of *db/db* mice with 10% GSK457 in combination with exendin-4 AlbudAb: reduction of glucose and HbA1c

**Fig 5:**
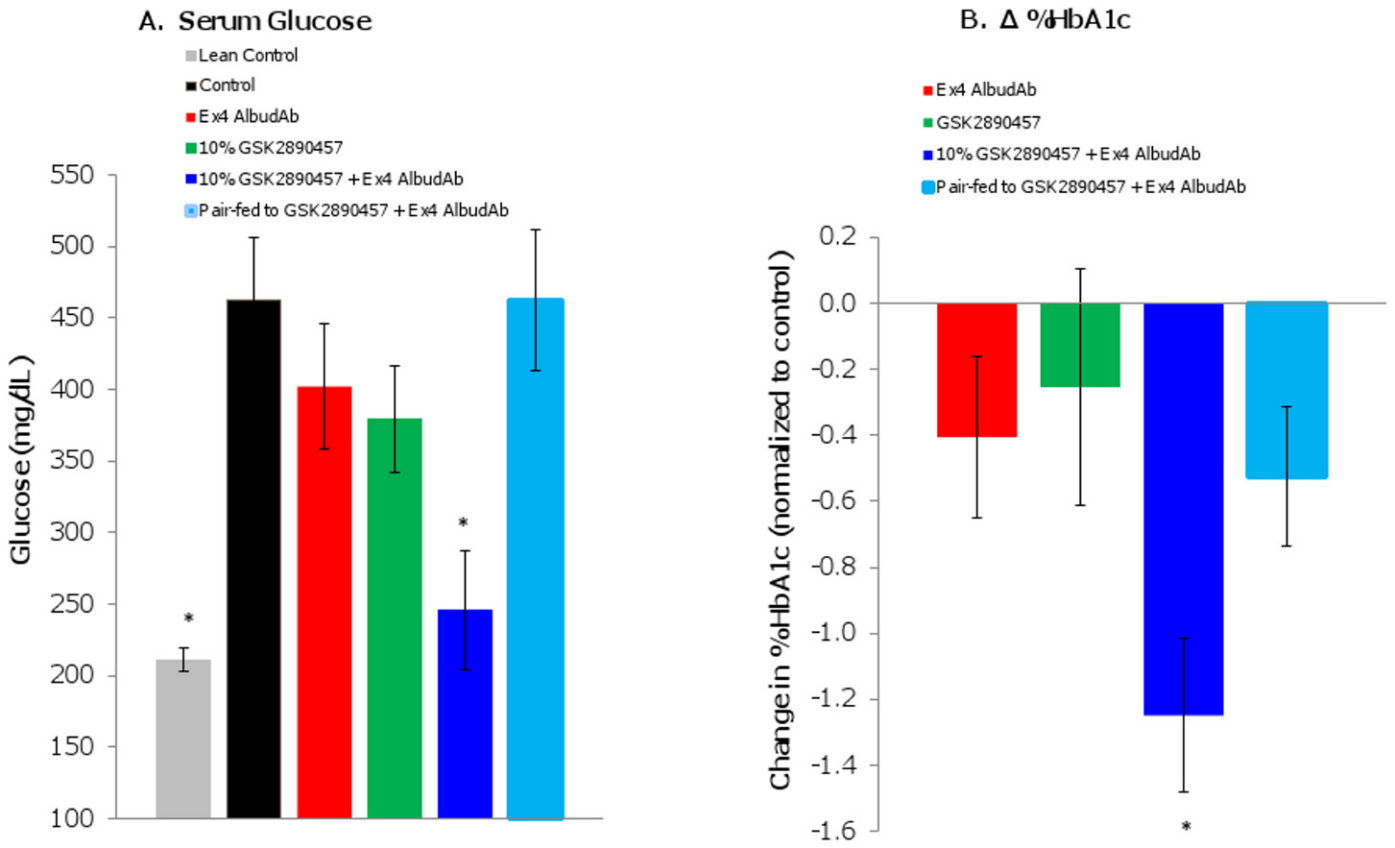
GSK457 + exendin-4 AlbudAb combination treatment reduced glucose and HbA1c levels in *db/db* mice after 14 days. GSK457 treatment began on Day -8 and exendin-4 AlbudAb SC dosing began on Day 0. Blood was collected on Day 15 to measure (A) serum glucose levels (mg/dL) and (B) HbA1c (change (Δ) in %, normalized to control). An asterisk (*) indicates a significant difference from vehicle (p < 0.05).

In *db/db* mice, 14-day administration of 10% GSK457 and exendin-4 AlbudAb in combination significantly reduced serum glucose (change from baseline -217mg/dL; p<0.001) and HbA1c levels (change from baseline -1.2%; p<0.001), while the pair-fed animals lost more weight without a statistically significant improvement in glucose or HbA1c (Fig 5). This demonstrates glycemic improvement in the combination treatment beyond that achieved by weight loss alone. In addition, the reduction in glucose (-217 mg/dL) and HbA1c (-1.2%) demonstrates effects of the combination that are greater than the sum of effects of each component (-142 mg/dL for glucose additivity; -0.7% for HbA1c additivity) (p<0.05).

Consistent with results seen in the DIO model, in the *db/db* mice the 10% GSK457 and exendin-4 AlbudAb combination resulted in normalization of clinical chemistry parameters (*e*.*g*. glucose, HbA1c, cholesterol, triglycerides, AST, ALT) back to lean control values. In addition, histological analysis of livers from combination treated groups confirmed amelioration of the hepatic steatosis in the *db/db* mice. Additional results relating to body weight, fat mass, food intake, serum chemistry parameters, hormones, and liver steatosis can be found in the S2 Nonclinical Results, S5 and S6 Figs and S3 Table.

### Clinical Results

#### Disposition and baseline demographics

The subject disposition in Parts A-C is shown in the CONSORT Fig 1.

In Part A, 1 subject (7%) from GSK457 group was withdrawn from the study due to 2 positive urinary drug tests. In Part B, 3 subjects (14%) were withdrawn; 2 (9%) due to adverse events in the Stabilization period on liraglutide alone, and 1 subject (5%) in the GSK457 group withdrew consent during the Treatment period. In Part C, 5 subjects (22%) were withdrawn in the Stabilization period on metformin alone; 2 (9%) at the investigators’ discretion (1 due to positive urine test for cocaine and the other due to a positive test for blood in stools), and 3 (13%) withdrew consent.

The demographic and baseline characteristics of the subjects are shown in Table 3.

**Table 3:** Clinical Study: Demographic and Baseline Characteristics.

Demographics	Part A		Part B		Part C	
	Placebo N = 4	GSK457 N = 11	Placebo N = 6	GSK457 N = 14	Placebo N = 6	GSK457 N = 12
**Age** (years), Median (Min, Max)	37.0 (24, 54)	29.0 (22, 42)	56.5 (53, 68)	57.0 (41, 63)	58.0 (52, 63)	56.5 (42, 66)
**Sex**, n (%)						
Female	1 (25)	1 (9)	3 (50)	3 (21)	3 (50)	4 (33)
Male	3 (75)	10 (91)	3 (50)	11 (79)	3 (50)	8 (67)
**BMI** (kg/m^2^), Mean (SD)	27.30 (3.509)	28.43 (3.722)	33.42 (2.032)	33.32 (2.898)	33.33 (2.976)	33.63 (3.435)
**Height** (cm), Mean (SD)	176.8 (4.99)	176.1 (7.13)	165.3 (6.35)	168.5 (8.01)	161.7 (10.15)	166.8 (8.82)
**Weight** (kg), Mean (SD)	85.48 (13.859)	88.14 (12.593)	91.53 (9.764)	94.94 (13.229)	87.0 (8.874)	93.70 (12.927)
**HbA1c** (%)	n/a	n/a	8.13 (1.104)	8.24 (0.903)	8.25 (0.807)	8.34 (0.962)
**HbA1c** (mmol/mol)	n/a	n/a	65.4 (12.06)	66.6 (9.87)	66.7 (8.82)	67.7 (10.51)

#### Part A—healthy subjects

GSK457 was tolerated, and all but 1 subject completed the 6 weeks of dosing on 40 g (the remaining subject completed on 30 g). GSK457 reduced metformin systemic exposure by ~30% (S10 Table).

Heart rate and blood pressure were measured in the fasted state first thing in the morning before breakfast after resting in a quiet room for at least 10 minutes. There was a small increase in heart rate which was greater in the placebo group than in the GSK672 group (S7 Fig). After dosing on Day 1, diastolic and systolic blood pressures were reduced in the GSK457 group relative to baseline and to placebo; the effect persisted through Day 42. There appeared to be a rebound of the cardiovascular parameters at the Follow−up visit (S7–S9 Figs).

#### Part B—pharmacodynamic results

**Fig 6:**
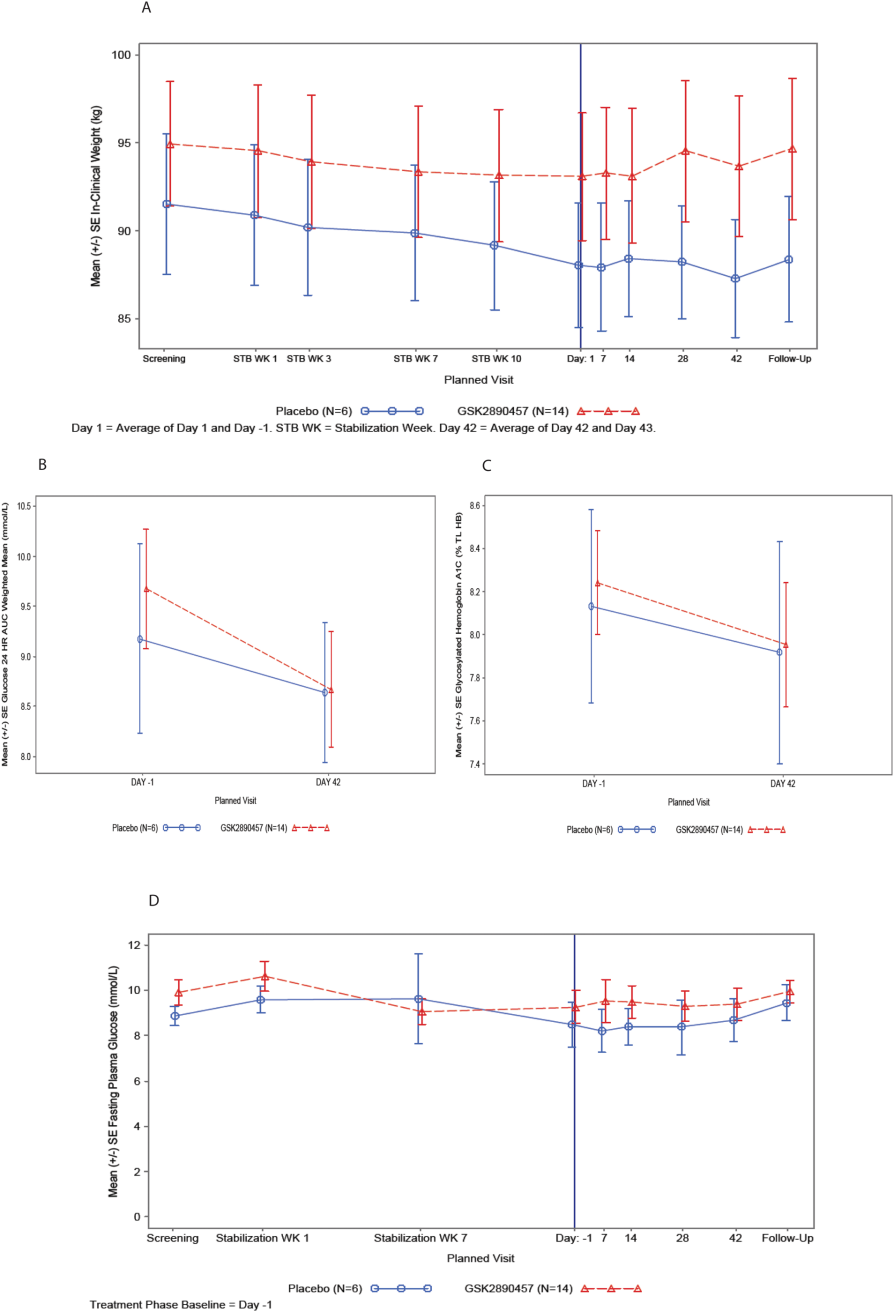
Mean weight, weighted mean glucose AUC (0–24 h), mean % HbA1c and mean fasting plasma glucose in T2D subjects taking liraglutide and GSK457 or placebo in Part B of the clinical study. Panel A shows the mean weight (± SE) from the Screening visit to the Follow-up visit and it includes the 3-month liraglutide Stabilization period and the 6-week Treatment period. Panels B and C show the weighted mean glucose AUC (0–24 h) and % HbA1c (± SE), respectively, at Baseline (Day -1) and at the end of the 6-week Treatment period (Day 42). Panel D shows the mean fasting plasma glucose (± SE) from the safety labs from the Screening visit to the Follow-up visit and it includes the 3-month liraglutide Stabilization period and the 6-week Treatment period. In all panels, the GSK457 group is shown as red symbols and lines and the placebo group as blue symbols and lines.

*Body weight*: At the start of the liraglutide Stabilization phase, mean body weight was slightly higher in subjects who were later randomized to GSK457 than to Placebo. There was a trend for a reduction in mean body weight during the 12-week Stabilization phase in both groups of subjects [mean ± SD GSK457 (−1.29kg ± 2.593) and placebo (−2.55kg ± 4.428)]. The mean (SE) body weight versus time is plotted in Fig 6.

Summary data and ANCOVA results for the Treatment period are presented in S4 Table. At Baseline (after 12 weeks of stabilization on Liraglutide), body weight (mean ± SE) was higher in the subjects randomized to GSK457 (94.0 kg ± 3.81) than those randomized to placebo (88.0 kg ± 3.52). After 6 weeks of randomized treatment, ANCOVA indicates that there was no difference between treatment groups in either change or % change from baseline body weight. The adjusted mean (SE) percent changes from baseline in the GSK457 and Placebo groups are -0.57% (0.463) and -0.69% (0.687), respectively.

*Glucose-related endpoints*: A summary of the 24-hour glucose weighted mean AUCs, change from baseline and the results of the ANCOVA of change from baseline is presented in S5 Table.

Weighted mean glucose AUC(0−24 h) was slightly higher in the GSK457 group than in the Placebo group at baseline. A decrease in adjusted mean (SE) change from baseline was observed in both the GSK457 [-0.968 (0.278) mmol/L] and Placebo [-0.613 (0.410) mmol/L] groups after 6 weeks of randomized treatment **(**S5 Table). Although there was a greater reduction in GSK457-treated subjects, there was no clinically meaningful difference between treatment groups in adjusted mean change from baseline (-0.356 mmol/L; 95% CI -1.409, 0.698).

At baseline, mean (±SD) HbA1c in the GSK457 group (8.23% ± 0.938) was comparable to that of the placebo group (8.13% ± 1.104). HbA1c was reduced in both treatment groups after 6 weeks of randomized treatment with no clinically meaningful difference between treatments [difference in adjusted mean change from baseline -0.065% (95% CI: -0.495, 0.365)] (S6 Table).

The plots for weighted mean glucose AUC (0–24 h), % HbA1c, and FPG mean (SE) over time are presented in Fig 6.

Both treatment groups demonstrated a decrease in FPG from Stabilization Week 1 to end of Stabilization Week 12. Baseline fasting plasma glucose at the start of the 6-week randomized Treatment period was higher in the GSK457 treated subjects (mean ± SE: 9.25 ± 0.729) than in Placebo subjects (8.48 ± 0.973). There was no apparent treatment effect of GSK457 on FPG.

#### Part C—pharmacodynamic results

*Body weight*: At the start of the metformin Stabilization period, mean body weight was slightly higher in subjects who were later randomized to GSK457 than to Placebo. There was a reduction in mean body weight during the 4-week Stabilization phase in both groups of subjects [mean ± SE GSK457 (-1.62 kg ± 1.121) and placebo (-1.17kg ± 0.693)].

**Fig 7:**
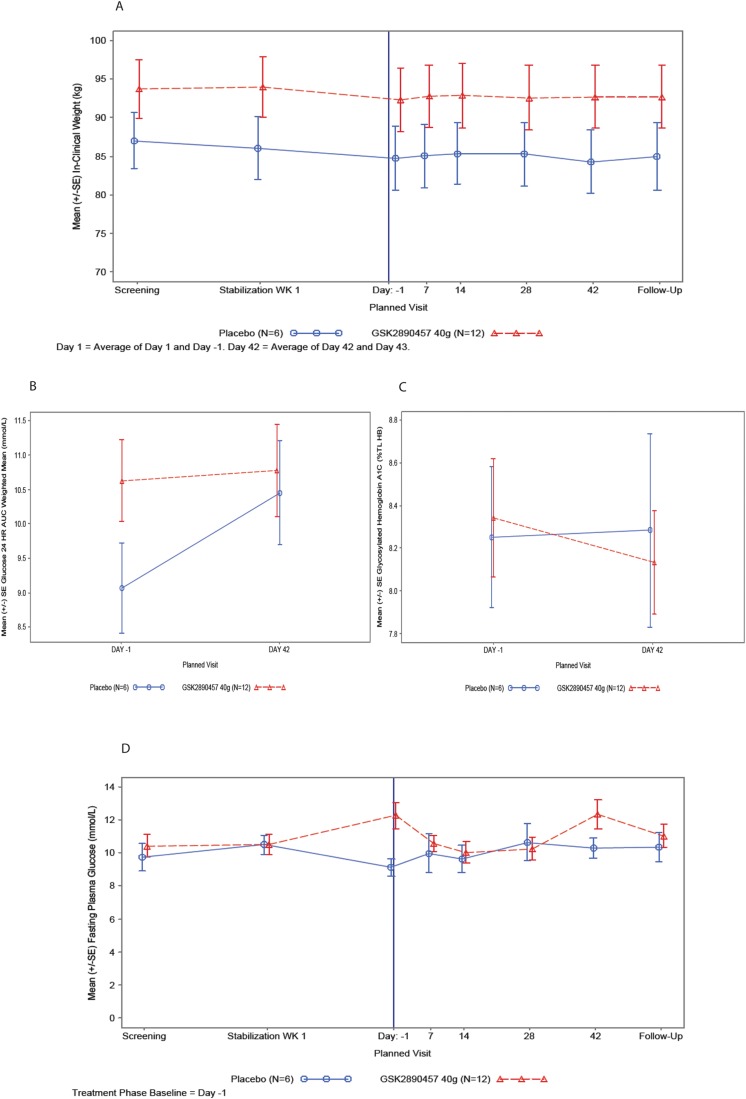
Mean weight, weighted mean glucose AUC (0–24 h), mean % HbA1c and mean fasting plasma glucose in T2D subjects taking metformin and GSK457 or placebo in Part C of the clinical study. Panel A shows the mean weight (± SE) from the Screening visit to the Follow-up visit and it includes the 1-month metformin Stabilization period and the 6-week Treatment period. Panels B and C show the weighted mean glucose AUC (0–24 h) and % HbA1c (± SE), respectively, at baseline (Day -1) and at the end of the 6-week Treatment period (Day 42). Panel D shows the mean fasting plasma glucose (± SE) from the safety labs from the Screening visit to the Follow-up visit and it includes the 1-month metformin Stabilization period and the 6-week Treatment period. In all panels, the GSK457 group is shown as red symbols and lines and the placebo group as blue symbols and lines.

Summary data and ANCOVA results for the Treatment period of Part C are presented in S7 Table. The mean (SE) body weight versus time is plotted in Fig 7.

After 4 weeks of stabilization on metformin, baseline body weight (mean ± SE) was higher in the subjects randomized to GSK457 (92.3 kg ± 3.46) than to placebo (84.7 kg ± 4.13). After 6 weeks of randomized treatment, there was an increase in weight in the GSK457 treated group [adjusted mean (SE) % change from baseline: 0.58% (0.404)] and a decrease in the placebo group [adjusted mean (SE) % change from baseline: −0.68% (0.573)].

*Glucose-related endpoints*: A summary of the 24-hour glucose weighted mean AUCs, change from baseline and the results of the ANCOVA of change from baseline are presented in S8 Table.

Weighted mean glucose (0−24h) was higher in the GSK457 group than in the Placebo group at baseline. After 6 weeks of randomized treatment, the difference between treatments in adjusted mean change from baseline is −1.22 mmol/L (95% CI −2.447, 0.156), indicating a small reduction in mean glucose in the GSK457 group relative to placebo, due to an increase in the placebo group.

At baseline, mean (±SE) HbA1c in the GSK457 group (8.34% ± 0.278) was comparable to the placebo group (8.25% ± 0.329). HbA1c was reduced in the GSK457 treated group after 6 weeks [adjusted mean (SE) change from baseline: −0.201% (0.187)], with no change occurring in the placebo group [adjusted mean (SE) change from baseline: 0.018% (0.264)] (S9 Table).

FPG at the start of the Stabilization phase was similar in both treatment groups. However from the start of Stabilization to the end of Stabilization Week 4, there was an increase in FPG in the GSK457 group and a decrease in placebo (Fig 7). This resulted in a difference in FPG at baseline between the GSK457 group [mean (SE): 12.249 mmol/L (0.780)] and placebo [mean (SE): 9.104 mmol/L (0.529)]. Relative to baseline and when compared to placebo, GSK457 reduced FPG on Days 7, 14 and 28, with a diminished effect on Day 42 (Fig 7). These effects may be due to regression to the mean.

The plots for 24-hour weighted mean glucose AUC (0–24 h) and % HbA1c are presented in Fig 7.

In both Parts B and C, there was no apparent treatment effect of GSK457 on fasting insulin, weighted mean insulin AUC(0-4 h) across the breakfast meal tolerance test, weighted mean insulin AUC(0-24h), HOMA−IR and the Matsuda Insulin Sensitivity Index. However, the variability was relatively high for the insulin-based parameters.

*Pharmacokinetics of metformin*: Plasma metformin PK parameters from Part A are presented in S10 Table. A statistical analysis of these parameters is presented in S11 Table. These analyses showed that co−administration of metformin with GSK457 resulted in a reduction in metformin plasma exposures. Metformin AUC(0−10 h) and Cmax decreased by 32% and 34%, respectively, in healthy subjects. As a result, in Part C the metformin dose was taken 1 hour before taking GSK457/placebo.

Statistical comparisons of metformin PK parameters from Part C were not performed. However, the comparative plot of individual metformin PK parameters showed that when the metformin and GSK457 doses were separated by 1 hour there was no apparent effect on Cmax, but there was a slight decrease in AUC(0−10 h).

*Pharmacokinetics of liraglutide*: Liraglutide PK parameters from Part B are presented in S12 Table, and the results of the statistical comparisons of liraglutide PK parameters are in S13 Table.

The results show that co−administration of liraglutide with GSK457 had no effect on liraglutide plasma exposures in T2D patients. However, when the data from 1 outlier were excluded, there was an increase in liraglutide plasma exposures (liraglutide AUC(0−t) and Cmax increased by 28% and 22%, respectively (S14 Table).

*Safety and tolerability*: In general, GSK457 was tolerated by the subjects. All but 1 subject completed the 6 weeks of dosing on 40 g (the remaining subject completed on 30 g) in each of Parts A and B. In Part C, all subjects completed the 6 weeks of dosing on 40 g.

The change from baseline in overall GSRS score was used to assess the ‘bothersomeness’ of gastrointestinal symptoms reported by the subjects. These scores indicated that GSK457 had not caused bothersome gastrointestinal disturbances in Parts A-C of the study.

In Parts A-C, all adverse events were mild or moderate in intensity (Table 4), and there were no subject withdrawals due to an adverse event attributable to GSK457. There were no reports of SAEs, deaths or pregnancies during this study. Overall, there were no clinically meaningful changes in the safety parameters during any part of the study.

**Table 4:** Adverse Events during the Treatment Period Reported by More than 1 Subject.

System Organ Class Preferred Term	Part A		Part B		Part C	
	Placebo N = 4n (%)	GSK457 N = 11n (%)	Placebo N = 6n (%)	GSK457 N = 14n (%)	Placebo N = 6n (%)	GSK457 N = 14n (%)
ANY EVENT	3 (75)	10 (91)	1 (17)	3 (21)	3 (50)	3 (25)
Flatulence	2 (50)	9 (82)	0	0	0	0
Abdominal distension	0	5 (45)	0	1 (7)	0	0
Feces discolored	0	3 (27)	0	0	0	0
Abdominal pain upper	1 (25)	2 (18)	0	0	0	1 (8)
Diarrhea	0	2 (18)	1 (1)	1 (7)	0	1 (8)
Frequent bowel movements	0	2 (18)	0	0	0	0
Gastrointestinal motility disorder	0	2 (18)	0	0	0	0
Gastrointestinal sounds abnormal	1 (25)	1 (9)	0	1 (7)	0	0
Constipation	0	1 (9)	0	0	1 (17)	0
Eructation	0	1 (9)	0	1 (7)	0	0
Nausea	0	1 (9)	1 (17)	0	0	1 (8)
Upper respiratory tract infection	1 (25)	1 (9)	0	0	0	0

There were no adverse events of hypoglycemia reported in patients with T2D or in healthy subjects during this study. Because the powders used to make up GSK457 might cause upper respiratory tract irritation, respiratory symptoms were carefully monitored, but there did not appear to be an imbalance in reported AEs relating to the respiratory system.

*Part A*: In Part A, the most common drug-related adverse events reported for the GSK457 group were flatulence, abdominal distension, fecal discoloration, upper abdominal pain, diarrhea and frequent bowel movements.

*Part B*: During the Stabilization period with liraglutide the most common drug-related adverse events were nausea, diarrhea, decreased appetite, and headache.

In the treatment period for Part B there were no drug-related adverse events.

Heart rate and blood pressure were measured in the fasted state first thing in the morning before breakfast after resting in a quiet room for at least 10 minutes. There appeared to be a trend for an increase in heart rate and PR interval and a reduction in systolic and diastolic blood pressure during the liraglutide Stabilization period. Asymptomatic elevations of circulating lipase/amylase concentrations were noted in Part B while subjects were on liraglutide, but these were not associated with symptoms or signs of pancreatitis, and there was no clear differentiation between GSK457 and placebo-treated subjects.

*Part C*: There were no drug-related adverse events during the metformin stabilization period.

The drug-related adverse events reported during the Treatment period were diarrhea and nausea.

Relative to baseline and to placebo, there was a suggestion of a small increase in heart rate in the GSK457 group after dosing on Day 1, which peaked at Day 14 and was lower by Day 42 (S10 Fig). This was associated with a decrease in systolic and diastolic blood pressure, but there was little evidence of a rebound at the Follow−up visit, as was seen in Part A (S11 and S12 Figs).

## Discussion

This is the first report of pharmacological synergy observed with a combination of four GRAS agents in rodent models of obesity and T2D. We also describe the lack of fidelity of translation to humans. When we commenced our studies of nutritional agents, there was already precedent for the activity of non-digestible fibers [20–22], oleic acid [23, 24] and polyphenols [25–27]. Building on this background, GSK457 was developed following a systematic assessment of 16 nutritional agents known to have metabolic effects, using weight loss in the DIO mouse as the pharmacodynamic readout. In the DIO mouse weight loss model we observed synergy when GSK457 was combined with the long-acting GLP-1 receptor agonist, exendin-4 AlbudAb (weight loss of 30.8% after 28 days). Synergy was also observed when *db/db* mice were administered GSK457 and the exendin-4 AlbudAb in combination (glucose was reduced by 217 mg/dL and HbA1c was reduced by1.2%). The glycemic improvement was not explained by weight loss because pair-fed animals lost more weight without reductions of HbA1c or glucose. There is no clear explanation for the greater weight-loss observed in pair-fed animals, although untreated pair-fed animals may have blunted glucose utilization in their tissues whereas the treated animals may have better restoration of glucose utilization, tempering weight loss in the treatment group.

A number of potential mechanisms of action have been proposed for the components of GSK457. OFS and apple pectin are non-digestible fibers that are metabolized by bacteria to form short chain fatty acids that can interact with luminal G-protein coupled receptors in the gut, including GPR41 and GPR43, to release GLP-1, GLP-2, PYY and other gut hormones [28]. The fibers also modify the composition of the gut microbiota, favoring species such as *bifidobacteria* and *lactobacilli* that have been associated with metabolic health [29–31]. Immunomodulatory and anti-inflammatory effects have been observed in nonclinical studies and these may be related to direct effects on the microbiota and indirect effects on gut permeability and endotoxin absorption [7]. The weight loss potential of OFS and apple pectin has been studied in humans [20–22]. In the case of OFS, modest weight loss has been associated with increased levels of PYY and decreased levels of ghrelin [20]. Many beneficial effects have been ascribed to anthocyanins and polyphenols which are present at high levels in blackcurrant extract. For example, cyanidin-3-O-glucoside, one of the primary anthocyanins in blackcurrant extract, has been shown to reduce insulin resistance and hepatic steatosis in nonclinical species [26]. Our unpublished data also show that blackcurrant extract strongly stimulates the endogenous release of GLP-1. In addition, it may alter gut bacterial types (with changes of colonic short chain fatty acids), activate AMPK, up-regulate GLUT-4 and down-regulate RBP4, TNFα and MCP-1, resulting in increased insulin sensitivity [27, 32]. Oleic acid is a monounsaturated omega-9 fatty acid (18:1n9) that is the principal fatty acid of olive oil. It stimulates gut peptide release through direct interaction with long-chain fatty-acid receptors, including GPR40 and GPR120 [33, 34]. It is also a direct PPAR alpha ligand and is converted to 1-palmitoyl-2-oleoyl-sn-glycerol-3-phosphocholine, an endogenous ligand in the liver [35]. Furthermore, conversion to oleoylethanolamide creates the opportunity for agonism of GPR119, CB1 & PPAR alpha receptors [36]. It is not surprising that oleic acid is believed to confer the metabolic benefits seen with a Mediterranean diet.

The unprecedented synergy we observed with the GSK457/ long-acting GLP-1 receptor agonist combination in the DIO and *db/db* mouse models was sufficiently compelling to justify testing in humans. In subjects with T2D taking liraglutide, GSK457 did not reduce weight, but it reduced mean glucose by a small amount of doubtful clinical significance. In subjects with T2D taking metformin, there was a small increase in weight in the GSK457 group, and a small reduction in mean glucose and HbA1c. The lack of translation of the nonclinical efficacy to the human subjects with T2D was disappointing and caused us to examine possible causes in detail. Of the potential confounders that we considered, the most important is likely to have been the fact that GSK457 was mixed in the food provided to the mice (15% or 10% w/w in chow), so that food intake would have ensured ingestion of GSK457, whereas the clinical formulation was complicated to prepare and not easy to consume. As we were not able to source a GMP-grade process to manufacture GSK457 as a convenient bar or chew, we resorted to providing three of the components of GSK457 in individual childproof sachets and the oleic acid in capsules. This meant that a subject needed to consume an artificially-flavored 12 oz drink and 12 capsules twice a day when taking the top dose of 40 g that was tested in this study. We were not able to confirm adherence by measuring components of GSK457 in blood because they occur commonly in food components that are part of a normal Western diet. To mitigate the risk of non-adherence, drug accountability was performed at clinic visits, weekly for the first two weeks of treatment and every other week for the remaining treatment period. Phone contact reinforcement was performed in weeks where there was no clinic visit. In addition, we tested the acceptability of GSK457 in Part A with healthy subjects before embarking on Parts B and C with T2D subjects. Throughout the study we did not receive feedback that subjects could not consume the GSK457 drink and/or capsules, but 1 subject in Part A and 1 in Part B did not escalate up to 40 g and remained on 30 g for the duration of the treatment period because of gastrointestinal adverse events. Other factors may also have played a role in the lack of translation. The mice were provided with a chow of consistent composition, with GSK457 added uniformly, but the food choices of our subjects over the 6 weeks of treatment would have varied significantly, even within the constraints of a diabetic diet. The genetic variability of the humans would also have been much greater than that of the mice strains we used. It is also important to note that the mice were housed in close proximity and this would have ensured consistency of the microbial species in the gut, whereas T2D patients are known to have highly variable gut bacterial populations [16], and some of these species may have been less susceptible to the effects of the components of GSK457.

There is some precedent for nutritional components such as pectin affecting the absorption of orally administered drugs (See S1 Materials and Methods for further information). In the clinical study GSK457 appeared to alter the pharmacokinetics of metformin and liraglutide. As outlined in S10 and S11 Tables, metformin AUC(0−10 h) and Cmax decreased by 32% and 34%, respectively, in healthy subjects administered GSK457. As a result, in Part C the metformin dose was taken 1 h before taking GSK457 or placebo. Inspection of the concentration-time profiles indicated that this temporal separation reduced the interaction considerably. Overall, GSK457 did not affect liraglutide plasma exposures in the T2D patients. However, when the data from 1 outlier were excluded, there appeared to be an increase in liraglutide AUC(0−t) and Cmax by 28% and 22%, respectively (S14 Table). We do not have an explanation why oral GSK457 affected the pharmacokinetics of subcutaneously administered liraglutide, and this may simply reflect random variation in a small study population.

Despite the disappointing clinical glucose and weight results, it is worth noting that administration of GSK457 was associated with reductions in systolic and diastolic blood pressures compared to the placebo group, with an inconsistent effect on heart rate. While these were not predefined efficacy parameters, the results suggest that the combination of nutritional agents in GSK457 may have beneficial cardiovascular effects.

This project employed a number of novel approaches for the early drug development of an anti-obesity and anti-diabetic medication. First, the deliberate focus on nutritional agents with GRAS status was intended to maximize the safety profile of the eventual medicine. Secondly, we selected GSK457 following the systematic combinatorial assessment of 16 nutritional agents, and this mixture was found to synergize with a long-acting GLP-1 agonist in the animal models. Thirdly, at the time we were designing the first-in-human study for GSK457 there were no regulatory precedents for developing a combination of GRAS ingredients to treat obese patients with T2D. Advice obtained at a pre-IND meeting with the US Food and Drug Administration and the agency’s *Guidance for Industry*: *Botanical Drug Products (2004)* were used to guide the initial approach for human investigation. As a result, the first-in-human study was 6 weeks long and was supported by a single species (rat) 6-week Good Laboratory Practice general toxicity study, based on available nonclinical and clinical safety information in the literature for the individual agents. The subjects remained at home for the most part, with close supervision to ensure safety, and intermittent visits to the clinical unit.

In addition, we employed a wireless technology that allowed real-time monitoring of capillary blood glucose levels and weights while the subjects were at home. Each glucometer and weighing scale was connected to a wireless modem hub in the subjects’ homes, which transmitted data to a database managed by AMC Health that were viewable in real-time by the investigators, the GlaxoSmithKline study physician and clinical scientist. This bypassed errors that might result from recording of glucose and weight values by a subject. Examples of the fasting blood glucose and weight readings that were obtained are shown in S13 and S14 Figs, respectively. In S13 Fig, Panels A-D show data for individual T2D patients to illustrate how the fasting glucose values at home can be generally concordant with the laboratory values measured in the clinical unit with less (Panel A) or more (Panel B) daily variability. Panel C and D are examples where there is significant discordance between home and clinical unit values created by marked daily variation of fasting glucose. Panel E shows the mean glucose data from Part C for T2D subjects taking metformin and GSK457 or placebo. It illustrates well how the home monitoring of glucose can aid the interpretation of clinical trial data. In this case, the clinical unit laboratory data suggested a reduction of fasting plasma glucose during the first 2 weeks of treatment with GSK457, but the home monitoring capillary glucose values clearly show that the glucose changes during the treatment period were likely to be an artifact. S14 Fig is also revealing in that it shows examples of concordance (Panels A and B) and discordance (Panels C and D) between the weights measured at home and in the clinical unit. The frequent monitoring of weight allows the characterization of weight-change trajectories for each subject and identifies data points that are likely to be artifacts (black dots).

## Conclusions

The combination of four GRAS ingredients in GSK457 co-administered with an exendin-4 AlbudAb produced remarkable reductions of weight and blood glucose in mouse models, but only minor effects were observed in human subjects with T2D who were administered GSK457 with liraglutide or metformin. Our data indicate that caution should be exercised when predicting human efficacy of nutritional agents from rodent models of obesity and diabetes.

## Supporting Information

S1 CONSORT Checklist (DOCX)

S1 Fig GSK457 + exendin-4 AlbudAb combination treatment produced greater than additive weight loss and fat mass loss in DIO C57BL/6NTac mice after 28 days. (A) body weight (% change from baseline (Day -7) compared to vehicle), and (B) change in fat mass (g) and non-fat mass (g) from baseline. An asterisk (*) indicates a significant difference from vehicle (p < 0.05), the red line indicates the sum of the effects of the components GSK457 and exendin-4 AlbudAb, and # indicates a greater than additive effect (p < 0.05).
(DOCX)

S2 Fig GSK457 alone and GSK457 + exendin-4 AlbudAb combination treatment reduced daily and cumulative food intake in DIO C57BL/6NTac mice. (A) daily food intake and (B) cumulative food intake (Day -7 to 28), expressed as percentage reduction from vehicle values. An asterisk (*) indicates a significant difference from vehicle (p < 0.05), a red line indicates the sum of the effects of the components GSK457 and the exendin-4 AlbudAb, and # indicates a greater than additive effect (p < 0.05).
(DOCX)

S3 Fig GSK457 + Exendin-4 AlbudAb Combination—Change (Δ) in Chemistry and Hormone Parameters (% change from DIO C57BL6 Vehicle Control Mice). (DOCX)

S4 Fig GSK457 + exendin-4 AlbudAb combination treatment decreased cytoplasmic lipid droplets in the livers of DIO C57BL/6NTac mice after 28 days. Osmium staining, with similar magnification of liver from (A) DIO control and (B) GSK457 + exendin-4 AlbudAb treated mice. The red arrows point to lipid droplets.
(DOCX)

S5 Fig GSK457 + exendin-4 AlbudAb combination treatment produced inhibition of weight and fat mass gain in *db/db* mice after 14 days. (A) body weight, % change from baseline and (B) change (Δ) in fat mass (g) and non-fat mass (g) from baseline. An asterisk (*) indicates a significant difference from control (p < 0.05).
(DOCX)

S6 Fig GSK457 + exendin-4 AlbudAb combination treatment reduced daily and cumulative food intake in *db/db* mice. (A) daily food intake and (B) cumulative food intake (kcal), expressed as percentage change from control. An asterisk (*) indicates a significant difference from vehicle (p < 0.05), a red line indicates the sum of the effect of the components GSK457 and exendin-4 AlbudAb.
(DOCX)

S7 Fig Clinical Study Part A: Mean (SE) Change from Baseline of Heart Rate in Healthy Subjects. GSK457 (red triangles) or placebo (blue circles) were administered for 6 weeks. Subject titrated up to 40 g over 2 weeks, if tolerated, and then remained on that dose for the duration of the treatment period. There was a small increase in heart rate above baseline over the first 28 days that was greater in the placebo-treated group than in the GSK457 group.
(DOCX)

S8 Fig Clinical Study Part A: Mean (SE) Change from Baseline Systolic Blood Pressure in Healthy Subjects. GSK457 (red triangles) or placebo (blue circles) were administered for 6 weeks. Subject titrated up to 40 g over 2 weeks, if tolerated, and then remained on that dose for the duration of the treatment period. There was a small reduction in systolic pressure below baseline over the 42 days of the treatment period that was greater in the GSK457 group than in the placebo-treated group. Systolic pressure had risen back to baseline at the Follow-up visit.
(DOCX)

S9 Fig Clinical Study Part A: Mean (SE) Change from Baseline Diastolic Blood Pressure in Healthy Subjects. GSK457 (red triangles) or placebo (blue circles) were administered for 6 weeks. Subject titrated up to 40 g over 2 weeks, if tolerated, and then remained on that dose for the duration of the treatment period. There was a small reduction in diastolic pressure below baseline over the 42 days of the treatment period that was greater in the GSK457 group than in the placebo-treated group. Diastolic pressure had risen back to baseline at the Follow-up visit.
(DOCX)

S10 Fig Clinical Study Part C: Mean (SE) Change from Baseline of Heart Rate in T2D subjects on Metformin. GSK457 (red triangles) or placebo (blue circles) were administered for 6 weeks. Subject titrated up to 40 g over 2 weeks, if tolerated, and then remained on that dose for the duration of the treatment period. There was a small increase in heart rate above baseline over the first 28 days that was greater in the GSK457 group than in the placebo-treated group.
(DOCX)

S11 Fig Clinical Study Part C: Mean (SE) Change from Baseline Systolic Blood Pressure in T2D subjects on Metformin. GSK457 (red triangles) or placebo (blue circles) were administered for 6 weeks. Subject titrated up to 40 g over 2 weeks, if tolerated, and then remained on that dose for the duration of the treatment period. There was a reduction in systolic pressure below baseline over the 42 days of the treatment period in the GSK457 group, in contrast to the increase in systolic pressure observed in the placebo-treated group. Systolic pressure was trending towards baseline at the Day 42 and Follow-up visits.
(DOCX)

S12 Fig Clinical Study Part C: Mean (SE) Change from Baseline Diastolic Blood Pressure in T2D subjects on Metformin. GSK457 (red triangles) or placebo (blue circles) were administered for 6 weeks. There was a small reduction in diastolic pressure below baseline over the 42 days of the treatment period in the GSK457 group compared to the placebo-treated group. Diastolic pressure was trending back to baseline at the Follow-up visit.
(DOCX)

S13 Fig Examples of the fasting glucose recordings taken while T2D subjects were at home. Panels A-D show only the daily fasting capillary glucose values for 4 individual T2D subjects while at home. Fasting plasma glucose was part of the safety monitoring panel and was measured when the subject visited the clinic. Fasting capillary glucose values taken at home in some cases were concordant with the laboratory values, with less (Panel A) or more (Panel B) daily variability. Panel C and D are examples where there is significant discordance between home and clinical unit values created by marked daily variation of fasting capillary glucose. Panel E shows the mean data from Part C for subjects taking metformin and GSK457 or placebo. The mean plasma glucose values at the clinic visits suggested a reduction during the first 2 weeks of treatment in the GSK457 group. However, the home monitoring of capillary glucose clearly shows that the glucose changes during the treatment period were likely to be an artifact. Fasting capillary glucose values are shown by the blue symbols and fasting plasma glucose values by the red symbols.
(DOCX)

S14 Fig Examples of the weight recordings taken while T2D subjects were at home. Panels A-D show the daily weights for 4 individual T2D subjects while at home. Weights were also measured when the subject visited the clinic. Panels A and B are examples of concordance between home and clinic weights, with less and more daily variability, respectively, while panels C and D illustrate significant discordance. The frequent monitoring of weight at home allowed the characterization of weight-change trajectories for each subject and identified data points that were likely to be artifacts (black dots).
(DOCX)

S1 Materials and Methods Components of GSK457. (DOCX)

S2 Materials and Methods Nonclinical—Further Information. (DOCX)

S3 Materials and Methods Clinical—Further Information. (DOCX)

S1 Nonclinical Results Chronic 28-day Treatment of DIO Mice with 15% (w/w in chow) GSK457 in Combination with Exendin-4 AlbudAb. (DOCX)

S2 Nonclinical Results Chronic 14-day Treatment of *db/db* Mice with 10% GSK457. (DOCX)

S1 Protocol 116623 Redacted. (PDF)

S1 Table Serum Chemistry Parameters in DIO Mice Treated with GSK457 ± Ex4 AlbudAb. (DOCX)

S2 Table Hormone Panel Parameters in DIO Mice Treated with GSK457 ± Ex4 AlbudAb. (DOCX)

S3 Table Serum Chemistry Parameters in *db/db* Mice Treated with the Exendin-4 AlbudAb and GSK457. (DOCX)

S4 Table Results of ANCOVA of Change and % Change from Baseline In−Clinic Body Weight—Clinical Study Part B (Subjects with T2D taking Liraglutide). (DOCX)

S5 Table Results of the ANCOVA of Change from Baseline Weighted Mean Glucose and Fasting Plasma Glucose—Clinical Study Part B (Subjects with T2D taking Liraglutide). (DOCX)

S6 Table Results of the ANCOVA of Change from Baseline HbA1c − Clinical Study Part B (Subjects with T2D taking Liraglutide). (DOCX)

S7 Table Summary of Results of ANCOVA of Change and %Change from Baseline In−Clinic Body Weight − Clinical Study Part C (Subjects with T2D taking Metformin). (DOCX)

S8 Table Summary of the Results of the ANCOVA of Change from Baseline Weighted Mean Glucose − Clinical Study Part C (Subjects with T2D taking Metformin). (DOCX)

S9 Table Results of the ANCOVA of Change from Baseline HbA1c − Clinical Study Part C (Subjects with T2D taking Metformin). (DOCX)

S10 Table Summary of Selected Plasma Metformin Pharmacokinetic Parameters in Clinical Study Part A (Healthy Subjects). (DOCX)

S11 Table Statistical Comparison of Plasma Metformin Pharmacokinetic Parameters in Clinical Study Part A (Healthy Subjects). (DOCX)

S12 Table Summary of Plasma Liraglutide Pharmacokinetic Parameters in Clinical Study Part B (Subjects with T2D taking Liraglutide). (DOCX)

S13 Table Statistical Comparison of Plasma Liraglutide Pharmacokinetic Parameters—Clinical Study Part B (Subjects with T2D taking Liraglutide). (DOCX)

S14 Table Statistical Comparison of Plasma Liraglutide Pharmacokinetic Parameters, Excluding Outlier—Clinical Study Part B (Subjects with T2D taking Liraglutide). (DOCX)

## References

[1] Ng M, Fleming T, Robinson M, Thomson B, Graetz N, Margono C., et al Global, regional, and national prevalence of overweight and obesity in children and adults during 1980–2013: a systematic analysis for the Global Burden of Disease Study 2013. Lancet. 2014;384(9945):766–81. 24880830 10.1016/S0140-6736(14)60460-8 PMC4624264

[2] NIH Publication No. 98–4083. Health Risks of Overweight and Obesity. Clinical Guidelines on the Identification, Evaluation, and Treatment of Overweight and Obesity in Adults—The Evidence Report. Sep 1998: 12–25.

[3] Cani PD, Everard A, Duparc T. Gut microbiota, enteroendocrine functions and metabolism. Curt Opin Pharmacol. 2013;13;935–40. 10.1016/j.coph.2013.09.008 24075718

[4] Everard A, Cani PD, Diabetes, obesity, and gut microbiota. Best Prac Res Clin Gastroenterol. 2013;27(1):73–83. 10.1016/j.bpg.2013.03.007 23768554

[5] Ley RE, Blackhed F, Turnbaugh P, Lozupone CA, Knight RD, Gordon JI. Obesity alters gut microbial ecology. Proc Nat Acad Sci. 2005;102(31):11070–75. 16033867 10.1073/pnas.0504978102 PMC1176910

[6] Cani PD, Bibiloni R, Knauf C, Waget A, Neyrinck AM, Delzenne NM, et al Changes in Gut Microbiota Control Metabolic Endotoxemia-Induced Inflammation in High-Fat Diet-Induced Obesity and Diabetes in Mice. Diabetes. 2008;57(6):1470–81. 10.2337/db07-1403 18305141

[7] Cani PD, Possemiers S, Van de WT, Guiot Y, Everard A, Rottier O, et al Changes in gut microbiota control inflammation in obese mice through a mechanism involving GLP-2-driven improvement of gut permeability. Gut. 2009;58:1091–1103. 10.1136/gut.2008.165886 19240062 PMC2702831

[8] Janssen S, Deportere I. Nutrient sensing in the gut: new roads to therapeutics? Trends Endocrinol Metab. 2013;24(2):92–100. 10.1016/j.tem.2012.11.006 23266105

[9] Den Besten G, van Eunen K, Groen AK, Venema K, Reijingoud D-J, Bakker BM. The role of short-chain fatty acids in the interplay between diet, gut microbiota, and host energy metabolism. J Lipid Res. 2013;54:2325–40. 10.1194/jlr.R036012 23821742 PMC3735932

[10] Puddu A, Sanguineti R, Montecucco F, Viviani GL. Evidence for the Gut Microbiota Short-Chain Fatty Acids as Key Pathophysiological Molecules Improving Diabetes. Mediators Inflamm. 2014: Article ID 162021, 9 pages, 2014 10.1155/2014/162021 PMC4151858 25214711

[11] Moran TH. Gut peptides in the control of food intake. Int J Obes. 2009;33:S7–S10. 10.1038/ijo.2009.9 19363513

[12] Karra E, Batterham RL. The role of gut hormones in the regulation of body weight and energy homeostasis. Mol Cell Endocrinol. 2010;316(2):120–8. 10.1016/j.mce.2009.06.010 19563862

[13] Inzucchi SE, Bergenstal RM, Buse JB, Diamant M, Ferranini E. Nauck M, et al Management of Hyperglycemia in Type 2 Diabetes 2015 –A Patient-Centered Approach. Diabetes Care. 2015;38:140–9. 25538310 10.2337/dc14-2441

[14] Astrup A, Rossner S, van Gaal L, Rissanen A, Niskanen L, al Hakim M, et al Effects of liraglutide in the treatment of obesity: a randomized, double-blind, placebo-controlled study. Lancet. 2009;374(9701):1606–16. 19853906 10.1016/S0140-6736(09)61375-1

[15] Therakan G, Tan T, Bloom S. Emerging therapies in the treatment of ‘diabesity’: beyond GLP-1. Trends Pharmacol Sci. 2011;32(1):8–15. 21130506 10.1016/j.tips.2010.10.003

[16] Napolitano A, Miller S, Nicholls AW, Baker D, van Horn S. Thomas E, et al Novel Gut-Based Pharmacology of Metformin in Patients with Type 2 Diabetes Mellitus. PLOS ONE. 2014;9(7): e100778 10.1371/journal.pone.0100778 24988476 PMC4079657

[17] International Conference on Harmonisation (1996) ICH Harmonised Tripartite Guideline. Guideline for Good Clinical Practice. Version 10.

[18] World Medical Association (2000) Declaration of Helsinki: ethical principles for medical research involving human subjects. J Am Med Assoc. 284:3043–45. 11122593

[19] Svedlund J., Sjodin I, Dolevall G. GSRS—a clinical rating scale for gastrointestinal symptoms in patients with irritable bowel syndrome and peptic ulcer disease. Dig.Dis.Sci. 1988;33:129–134. 3123181 10.1007/BF01535722

[20] Parnell JA, Reimer RA. Weight loss during oligofructose supplementation is associated with decreased ghrelin and increased peptide YY in overweight and obese adults. Am J Clin Nutr. 2009;89:1751–59. 10.3945/ajcn.2009.27465 19386741 PMC3827013

[21] DiLorenzo C, Williams CM, Hajnal F, Valenzuela JE. Pectin delays Gastric Emptying and Increases Satiety in Obese Subjects. Gastroenterol. 1988:95(5):1211–15. 10.1016/0016-5085(88)90352-6 3169489

[22] Schwartz SE, Levine RA, Weinstock RS, Petokas S, Mills CA, Thomas FD. Sustained pectin ingestion: effect on gastric emptying and glucose tolerance in non-insulin-dependent diabetic patients. Am J Clin Nutr. 1988;48:1413–17. 2849298 10.1093/ajcn/48.6.1413

[23] Carr RD, Larsen MO, Winzell MS, Jelic K, Lindgren O, Deacon CF, et al Incretin and islet hormonal responses to fat and protein ingestion in healthy men. Am J Physiol Endocrinol Metab. 2008;295:E779–E784. 10.1152/ajpendo.90233.2008 18612044

[24] Teres S, Barcelo-Coblijn G, Benet M, Alvarez R, Bressani R, Halver JE, et al Oleic acid content is responsible for the reduction in blood pressure induced by olive oil. PNAS. 2008;105(37):13811–16. 10.1073/pnas.0807500105 18772370 PMC2544536

[25] Munir KM, Chandrasekaran S, Gao F, Quon MJ. Mechanisms for food polyphenols to ameliorate insulin resistance and endothelial dysfunction: therapeutic implications for diabetes and its cardiovascular complications. Am J Physiol Endorcinol Metab. 2013:305(6):E679–E686. 10.1152/ajpendo.00377.2013 PMC4073986 23900418

[26] Guo H, Xia M, Zou T, Ling W, Zhong R, Zhang W. Cyanidin 3-glucoside attenuates obesity-associated insulin resistance and hepatic steatosis in high-fat diet-fed and *db/db* mice via the transcription factor Fox01. J Nutr Biochem. 2012;23(4):349–60. 21543211 10.1016/j.jnutbio.2010.12.013

[27] Tsuda T. Regulation of Adipocyte Function by Anthocyanins; Possibility of Preventing the Metabolic Syndrome. J. Agric. Food Chem. 2008;56:642–46. 10.1021/jf073113b 18211021

[28] Delzenne NM, Cani PD, Daubioul C, Neyrinck AM. Impact of inulin and oligofructose on gastrointestinal peptides. Br J Nutr. 2005;93(Suppl. 1):S157–S161. 15877889 10.1079/bjn20041342

[29] Gibson GR, Beatty ER, Wang X, Cummings JH. Selective stimulation of bifidobacteria in the human colon by oligofructose and inulin. Gastroenterol. 1995;108(4):975–982. 10.1016/0016-5085(95)90192-2 7698613

[30] Howard M D, Gordon DT, Garleb KA, Kerley MS. Dietary fructooligosaccharide, xylooligosaccharide and gum arabic have variable effects on cecal and colonic microbiota and epithelial cell proliferation in mice and rats. J Nutr. 1995;125(10):2604–2609. 7562096 10.1093/jn/125.10.2604

[31] Kleessen B, Hartmann L, Blaut M. Oligofructose and long-chain inulin: Influence on the gut microbial ecology of rats associated with a human faecal flora. Bri J Nutri. 2001;86(2):291–300. 10.1079/bjn2001403 11502244

[32] Sasaki R, Nishimura N, Hoshino H, Isa Y, Kadowaki M, Ichi T, et al Cyanidin 3-glucoside ameliorates hyperglycemia and insulin sensitivity due to downregulation of retinol binding protein 4 expression in diabetic mice. Biochem Pharmacol. 2007;74:1619–1627. 17869225 10.1016/j.bcp.2007.08.008

[33] Burant CF. Activation of GPR40 as a Therapeutic Target for the Treatment of Type 2 Diabetes. Diabetes Care. 2013;36(S2):S175–S179. 23882043 10.2337/dcS13-2037 PMC3920793

[34] Zhang D, Leung PS. Potential roles of GPR120 and its agonists in the management of diabetes. Drug Des, Devel Ther. 2014;8:1013–1027. 10.2147/DDDT.S53892 PMC4122337 25114508

[35] Chakravarthy MV, Lodhi IF, Yin L, Malapaka RRV, Xu HE, Turk J, et al Identification of a Physiologically Relevant Endogenous Ligand for PPARα in Liver. Cell. 2009;138(3):476–488. 19646743 10.1016/j.cell.2009.05.036 PMC2725194

[36] Godlewski G, Offertaler L, Wagner JA, Kunos G. Receptors for acylethanoloamides—GPR55 and GPR119. Prostaglandins Other Lipid Mediat. 2009;89(3–4):105–111. 10.1016/j.prostaglandins.2009.07.001 19615459 PMC2751869

