# Supplementary material for: Weight and Glucose Reduction Observed with a Combination of Nutritional Agents in Rodent Models Does Not Translate to Humans in a Randomized Clinical Trial with Healthy Volunteers and Subjects with Type 2 Diabetes
Source: PLoS One. 2016 Apr 19;11(4):e0153151. doi: 10.1371/journal.pone.0153151 (PMC4836696; doi:10.1371/journal.pone.0153151)
Supplement: S1 Fig — (A) body weight (% change from baseline (Day -7) compared to vehicle), and (B) change in fat mass (g) and non-fat mass (g) from baseline. An asterisk (*) indicates a significant difference from vehicle (p < 0.05), the red line indicates the sum of the effects of the components GSK457 and exendin-4 AlbudAb, and # indicates a greater than additive effect (p < 0.05). (DOCX) [file pone.0153151.s002.docx]

S1 Fig. GSK457 + exendin-4 AlbudAb combination treatment produced greater than additive weight loss and fat mass loss in DIO C57BL/6NTac mice after 28 days**.** (A) body weight (% change from baseline (Day -7) compared to vehicle), and (B) change in fat mass (g) and non-fat mass (g) from baseline. An asterisk (*) indicates a significant difference from vehicle (p < 0.05), the red line indicates the sum of the effects of the components of GSK457 and exendin-4 AlbudAb administered alone, and # indicates a greater than additive effect (p < 0.05).
