# Supplementary material for: Weight and Glucose Reduction Observed with a Combination of Nutritional Agents in Rodent Models Does Not Translate to Humans in a Randomized Clinical Trial with Healthy Volunteers and Subjects with Type 2 Diabetes
Source: PLoS One. 2016 Apr 19;11(4):e0153151. doi: 10.1371/journal.pone.0153151 (PMC4836696; doi:10.1371/journal.pone.0153151)

S2 Fig. GSK457 alone and GSK457 + exendin-4 AlbudAb combination treatment reduced daily and cumulative food intake in DIO C57BL/6NTac mice**.** (A) daily food intake and (B) cumulative food intake (Day -7 to 28), expressed as percentage reduction compared to the vehicle group. An asterisk (*) indicates a significant difference from vehicle (p < 0.05), a red line indicates the sum of the effects of the components of GSK457 and the exendin-4 AlbudAb, and # indicates a greater than additive effect (p < 0.05).


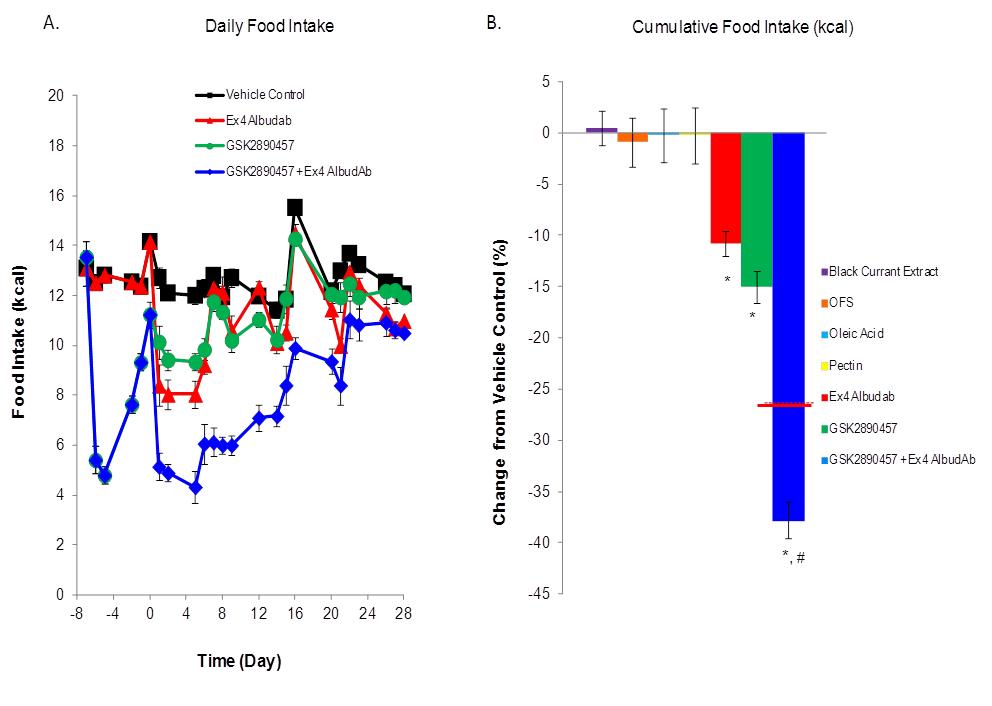

Supplement: S2 Fig — (A) daily food intake and (B) cumulative food intake (Day -7 to 28), expressed as percentage reduction from vehicle values. An asterisk (*) indicates a significant difference from vehicle (p < 0.05), a red line indicates the sum of the effects of the components GSK457 and the exendin-4 AlbudAb, and # indicates a greater than additive effect (p < 0.05). (DOCX) [file pone.0153151.s003.docx]
