# Supplementary material for: Weight and Glucose Reduction Observed with a Combination of Nutritional Agents in Rodent Models Does Not Translate to Humans in a Randomized Clinical Trial with Healthy Volunteers and Subjects with Type 2 Diabetes
Source: PLoS One. 2016 Apr 19;11(4):e0153151. doi: 10.1371/journal.pone.0153151 (PMC4836696; doi:10.1371/journal.pone.0153151)
Supplement: S3 Fig — (DOCX) [file pone.0153151.s004.docx]

## S3 Fig. GSK457 + Exendin-4 AlbudAb Combination - Change (∆) in Chemistry and Hormone Parameters (% change from DIO C57BL6 Vehicle Control Mice)
