# Supplementary material for: Weight and Glucose Reduction Observed with a Combination of Nutritional Agents in Rodent Models Does Not Translate to Humans in a Randomized Clinical Trial with Healthy Volunteers and Subjects with Type 2 Diabetes
Source: PLoS One. 2016 Apr 19;11(4):e0153151. doi: 10.1371/journal.pone.0153151 (PMC4836696; doi:10.1371/journal.pone.0153151)
Supplement: S4 Fig — Osmium staining, with similar magnification of liver from (A) DIO control and (B) GSK457 + exendin-4 AlbudAb treated mice. The red arrows point to lipid droplets. (DOCX) [file pone.0153151.s005.docx]

S4 Fig. GSK457 + exendin-4 AlbudAb combination treatment decreased cytoplasmic lipid droplets in the livers of DIO C57BL/6NTac mice after 28 days**.** Osmium staining of liver sections under similar magnification from (A) DIO control, and (B) GSK457 + exendin-4 AlbudAb treated mice. The red arrows point to lipid droplets.
