# Supplementary material for: Weight and Glucose Reduction Observed with a Combination of Nutritional Agents in Rodent Models Does Not Translate to Humans in a Randomized Clinical Trial with Healthy Volunteers and Subjects with Type 2 Diabetes
Source: PLoS One. 2016 Apr 19;11(4):e0153151. doi: 10.1371/journal.pone.0153151 (PMC4836696; doi:10.1371/journal.pone.0153151)
Supplement: S5 Fig — (A) body weight, % change from baseline and (B) change (Δ) in fat mass (g) and non-fat mass (g) from baseline. An asterisk (*) indicates a significant difference from control (p < 0.05). (DOCX) [file pone.0153151.s006.docx]

S5 Fig. GSK457 + exendin-4 AlbudAb combination treatment induced weight loss and inhibition of fat mass gain in *db/db* mice after 14 days**.** (A) body weight, % change from baseline, and (B) change (Δ) in fat mass (g) and non-fat mass (g) from baseline. An asterisk (*) indicates a significant difference from control (p < 0.05).
