# Supplementary material for: Weight and Glucose Reduction Observed with a Combination of Nutritional Agents in Rodent Models Does Not Translate to Humans in a Randomized Clinical Trial with Healthy Volunteers and Subjects with Type 2 Diabetes
Source: PLoS One. 2016 Apr 19;11(4):e0153151. doi: 10.1371/journal.pone.0153151 (PMC4836696; doi:10.1371/journal.pone.0153151)

S6 Fig. GSK457 + exendin-4 AlbudAb combination treatment reduced daily and cumulative food intake in *db/db* mice. (A) daily food intake, and (B) cumulative food intake (kcal), expressed as percentage change from control. An asterisk (*) indicates a significant difference from vehicle (p < 0.05), a red line indicates the sum of the effect of the components GSK457 and exendin-4 AlbudAb.


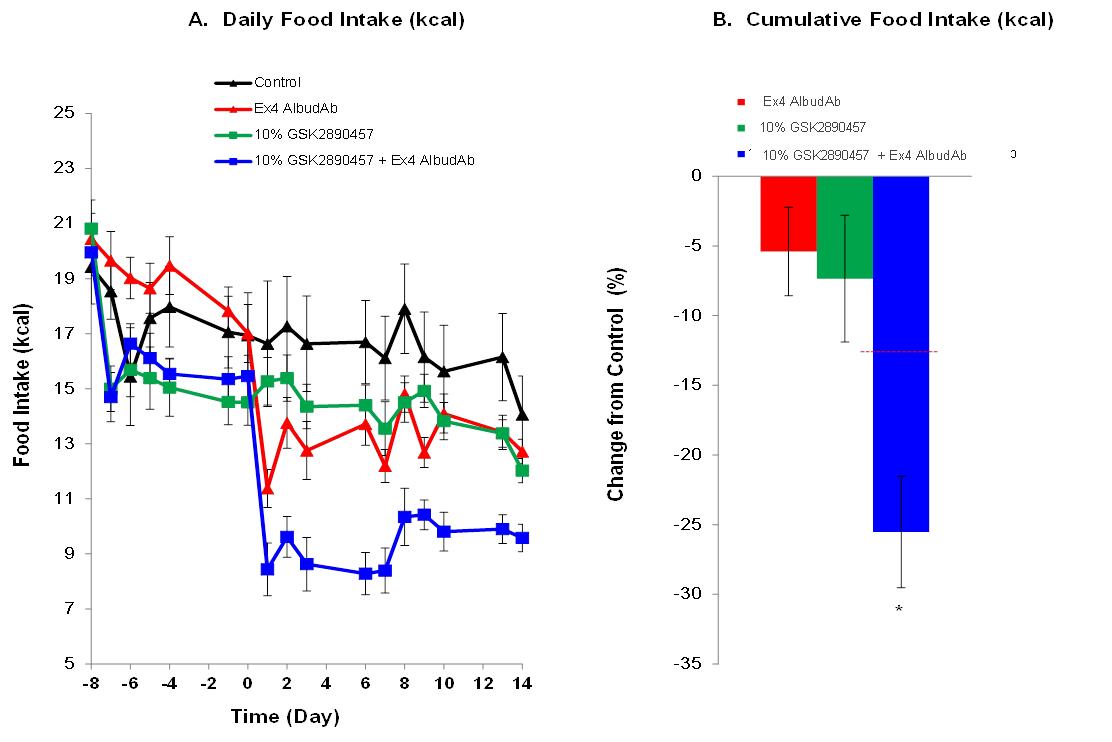

Supplement: S6 Fig — (A) daily food intake and (B) cumulative food intake (kcal), expressed as percentage change from control. An asterisk (*) indicates a significant difference from vehicle (p < 0.05), a red line indicates the sum of the effect of the components GSK457 and exendin-4 AlbudAb. (DOCX) [file pone.0153151.s007.docx]
