# Supplementary material for: Weight and Glucose Reduction Observed with a Combination of Nutritional Agents in Rodent Models Does Not Translate to Humans in a Randomized Clinical Trial with Healthy Volunteers and Subjects with Type 2 Diabetes
Source: PLoS One. 2016 Apr 19;11(4):e0153151. doi: 10.1371/journal.pone.0153151 (PMC4836696; doi:10.1371/journal.pone.0153151)
Supplement: S8 Fig — GSK457 (red triangles) or placebo (blue circles) were administered for 6 weeks. Subject titrated up to 40 g over 2 weeks, if tolerated, and then remained on that dose for the duration of the treatment period. There was a small reduction in systolic pressure below baseline over the 42 days of the treatment period that was greater in the GSK457 group than in the placebo-treated group. Systolic pressure had risen back to baseline at the Follow-up visit. (DOCX) [file pone.0153151.s009.docx]

S8 Fig. Clinical Study Part A: Mean (SE) Change from Baseline Systolic Blood Pressure in Healthy Subjects. GSK457 (red triangles) or placebo (blue circles) were administered for 6 weeks. Subject titrated up to 40 g over 2 weeks, if tolerated, and then remained on that dose for the duration of the treatment period. There was a small reduction in systolic pressure below baseline over the 42 days of the treatment period that was greater in the GSK457 group than in the placebo-treated group. Systolic pressure had risen back to baseline at the Follow-up visit.

Note: Baseline mean systolic blood pressure (SD): Placebo group: 116.3 (6.08) mm Hg; GSK457 group: 120.5 (11.13) mm Hg
