# Supplementary material for: Weight and Glucose Reduction Observed with a Combination of Nutritional Agents in Rodent Models Does Not Translate to Humans in a Randomized Clinical Trial with Healthy Volunteers and Subjects with Type 2 Diabetes
Source: PLoS One. 2016 Apr 19;11(4):e0153151. doi: 10.1371/journal.pone.0153151 (PMC4836696; doi:10.1371/journal.pone.0153151)
Supplement: S10 Fig — GSK457 (red triangles) or placebo (blue circles) were administered for 6 weeks. Subject titrated up to 40 g over 2 weeks, if tolerated, and then remained on that dose for the duration of the treatment period. There was a small increase in heart rate above baseline over the first 28 days that was greater in the GSK457 group than in the placebo-treated group. (DOCX) [file pone.0153151.s011.docx]

S10 Fig. Clinical Study Part C: Mean (SE) Change from Baseline of Heart Rate in T2D subjects on metformin. GSK457 (red triangles) or placebo (blue circles) were administered for 6 weeks. Subject titrated up to 40 g over 2 weeks, if tolerated, and then remained on that dose for the duration of the treatment period. There was a small increase in heart rate above baseline over the first 28 days that was greater in the GSK457 group than in the placebo-treated group.
