# Supplementary material for: Weight and Glucose Reduction Observed with a Combination of Nutritional Agents in Rodent Models Does Not Translate to Humans in a Randomized Clinical Trial with Healthy Volunteers and Subjects with Type 2 Diabetes
Source: PLoS One. 2016 Apr 19;11(4):e0153151. doi: 10.1371/journal.pone.0153151 (PMC4836696; doi:10.1371/journal.pone.0153151)
Supplement: S11 Fig — GSK457 (red triangles) or placebo (blue circles) were administered for 6 weeks. Subject titrated up to 40 g over 2 weeks, if tolerated, and then remained on that dose for the duration of the treatment period. There was a reduction in systolic pressure below baseline over the 42 days of the treatment period in the GSK457 group, in contrast to the increase in systolic pressure observed in the placebo-treated group. Systolic pressure was trending towards baseline at the Day 42 and Follow-up visits. (DOCX) [file pone.0153151.s012.docx]

S11 Fig. Clinical Study Part C: Mean (SE) Change from Baseline Systolic Blood Pressure in T2D subjects on metformin**.** GSK457 (red triangles) or placebo (blue circles) were administered for 6 weeks. Subject titrated up to 40 g over 2 weeks, if tolerated, and then remained on that dose for the duration of the treatment period. There was a reduction in systolic pressure below baseline over the 42 days of the treatment period in the GSK457 group, in contrast to the increase in systolic pressure observed in the placebo-treated group. Systolic pressure was trending towards baseline at the Day 42 and Follow-up visits.

Note: Baseline mean (SD): Placebo 124.5 (15.65) mm Hg; GSK457: 130.9 (14.04) mm Hg
