# Supplementary material for: Weight and Glucose Reduction Observed with a Combination of Nutritional Agents in Rodent Models Does Not Translate to Humans in a Randomized Clinical Trial with Healthy Volunteers and Subjects with Type 2 Diabetes
Source: PLoS One. 2016 Apr 19;11(4):e0153151. doi: 10.1371/journal.pone.0153151 (PMC4836696; doi:10.1371/journal.pone.0153151)
Supplement: S12 Fig — GSK457 (red triangles) or placebo (blue circles) were administered for 6 weeks. There was a small reduction in diastolic pressure below baseline over the 42 days of the treatment period in the GSK457 group compared to the placebo-treated group. Diastolic pressure was trending back to baseline at the Follow-up visit. (DOCX) [file pone.0153151.s013.docx]

S12 Fig. Clinical Study Part C: Mean (SE) Change from Baseline Diastolic Blood Pressure in T2D subjects on metformin. GSK457 (red triangles) or placebo (blue circles) were administered for 6 weeks. Subject titrated up to 40 g over 2 weeks, if tolerated, and then remained on that dose for the duration of the treatment period. There was a small reduction in diastolic pressure below baseline over the 42 days of the treatment period in the GSK457 group compared to the placebo-treated group. Diastolic pressure was trending back to baseline at the Follow-up visit.

Note: Baseline mean (SD): Placebo 76.4 (5.81) mm Hg; GSK457: 80.0 (7.48) mm Hg
