# Supplementary material for: Weight and Glucose Reduction Observed with a Combination of Nutritional Agents in Rodent Models Does Not Translate to Humans in a Randomized Clinical Trial with Healthy Volunteers and Subjects with Type 2 Diabetes
Source: PLoS One. 2016 Apr 19;11(4):e0153151. doi: 10.1371/journal.pone.0153151 (PMC4836696; doi:10.1371/journal.pone.0153151)

S13 Fig. Examples of the fasting glucose recordings taken while T2D subjects were at home**.**  Panels A-D show only the daily fasting capillary glucose values for 4 individual T2D subjects while at home. Fasting plasma glucose was part of the safety monitoring panel and was measured when the subject visited the clinic. Fasting capillary glucose values taken at home in some cases were concordant with the laboratory values, with less (Panel A) or more (Panel B) daily variability. Panel C and D are examples where there is significant discordance between home and clinical unit values created by marked daily variation of fasting capillary glucose. Panel E shows the mean data from Part C for subjects taking metformin and GSK457 or placebo. The mean plasma glucose values at the clinic visits suggested a reduction during the first 2 weeks of treatment in the GSK457 group. However, the home monitoring of capillary glucose clearly shows that the glucose changes during the treatment period were likely to be an artifact. Fasting capillary glucose values are shown by the blue symbols and fasting plasma glucose values by the red symbols.


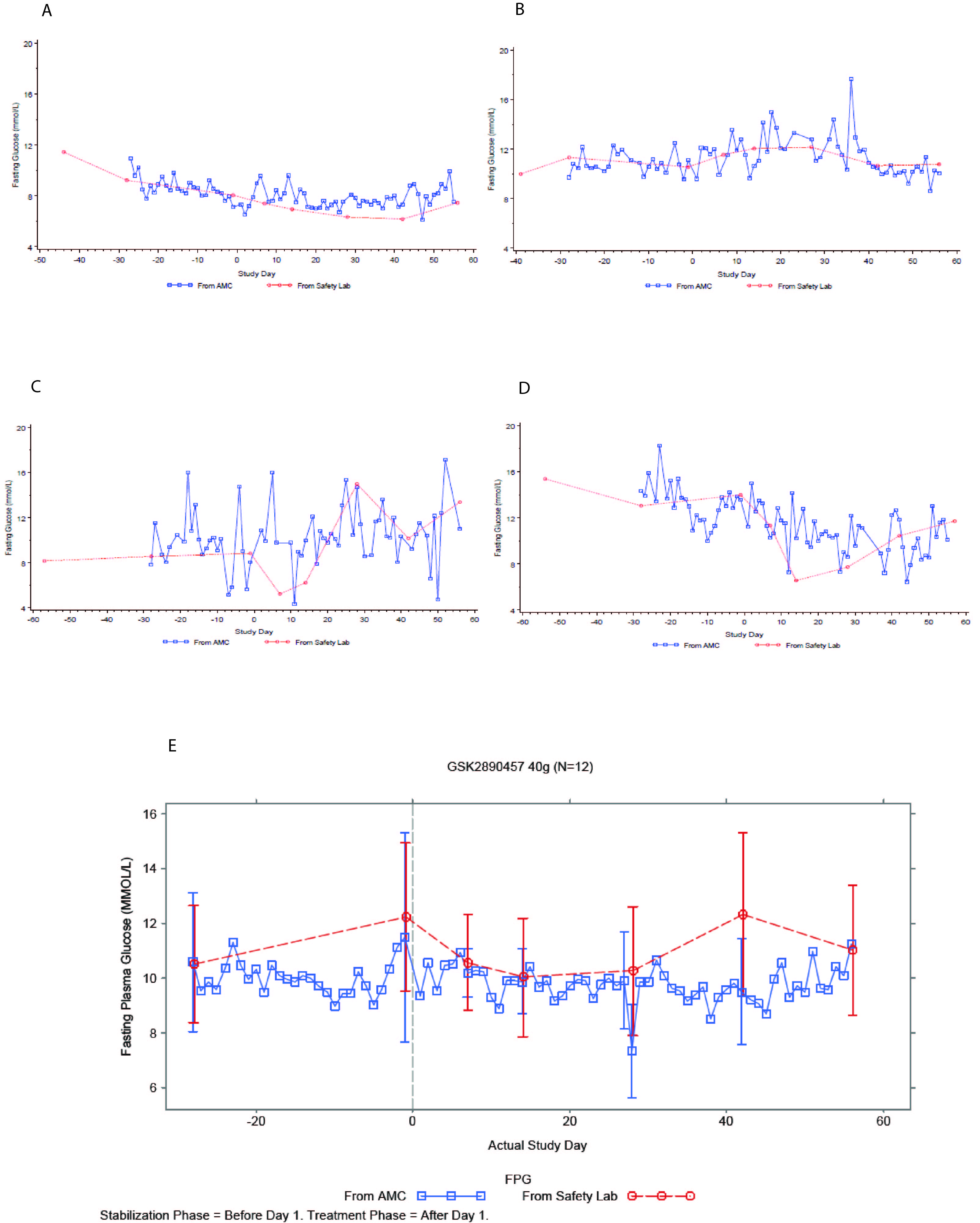

Supplement: S13 Fig — Panels A-D show only the daily fasting capillary glucose values for 4 individual T2D subjects while at home. Fasting plasma glucose was part of the safety monitoring panel and was measured when the subject visited the clinic. Fasting capillary glucose values taken at home in some cases were concordant with the laboratory values, with less (Panel A) or more (Panel B) daily variability. Panel C and D are examples where there is significant discordance between home and clinical unit values created by marked daily variation of fasting capillary glucose. Panel E shows the mean data from Part C for subjects taking metformin and GSK457 or placebo. The mean plasma glucose values at the clinic visits suggested a reduction during the first 2 weeks of treatment in the GSK457 group. However, the home monitoring of capillary glucose clearly shows that the glucose changes during the treatment period were likely to be an artifact. Fasting capillary glucose values are shown by the blue symbols and fasting plasma glucose values by the red symbols. (DOCX) [file pone.0153151.s014.docx]
