# Supplementary material for: Weight and Glucose Reduction Observed with a Combination of Nutritional Agents in Rodent Models Does Not Translate to Humans in a Randomized Clinical Trial with Healthy Volunteers and Subjects with Type 2 Diabetes
Source: PLoS One. 2016 Apr 19;11(4):e0153151. doi: 10.1371/journal.pone.0153151 (PMC4836696; doi:10.1371/journal.pone.0153151)

S14 Fig. Examples of the weight recordings taken while T2D subjects were at home. Panels A-D show the daily weights for 4 individual T2D subjects while at home. Weights were also measured when the subject visited the clinic. Panels A and B are examples of concordance between home and clinic weights, with less and more daily variability, respectively, while panels C and D illustrate significant discordance. The frequent monitoring of weight at home allowed the characterization of weight-change trajectories for each subject and identified data points that were likely to be artifacts (black dots).


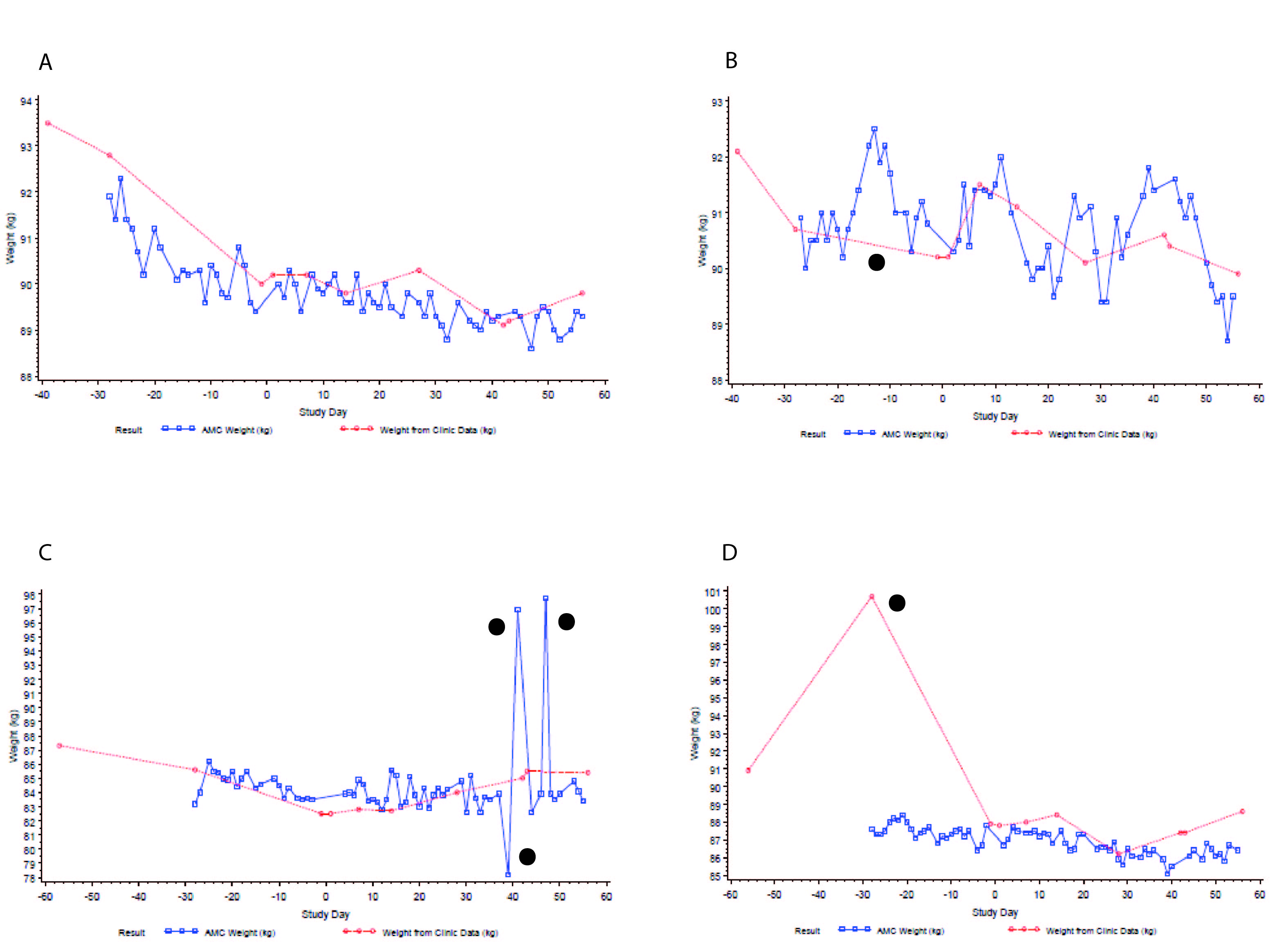

Supplement: S14 Fig — Panels A-D show the daily weights for 4 individual T2D subjects while at home. Weights were also measured when the subject visited the clinic. Panels A and B are examples of concordance between home and clinic weights, with less and more daily variability, respectively, while panels C and D illustrate significant discordance. The frequent monitoring of weight at home allowed the characterization of weight-change trajectories for each subject and identified data points that were likely to be artifacts (black dots). (DOCX) [file pone.0153151.s015.docx]
