# Supplementary material for: Weight and Glucose Reduction Observed with a Combination of Nutritional Agents in Rodent Models Does Not Translate to Humans in a Randomized Clinical Trial with Healthy Volunteers and Subjects with Type 2 Diabetes
Source: PLoS One. 2016 Apr 19;11(4):e0153151. doi: 10.1371/journal.pone.0153151 (PMC4836696; doi:10.1371/journal.pone.0153151)
Supplement: S1 Materials and Methods — (DOCX) [file pone.0153151.s016.docx]

**S1 Materials and Methods**

**Components of GSK2890457**

OFS is an oligosaccharide shorter-chain form of inulin, a fiber found in many plants. It has been studied extensively for its effects on immune function and inflammation, and it is used as a supplement in food products and infant formula. It has demonstrated modest weight loss effects in humans, in association with increased levels of PYY and decreased levels of ghrelin [19]. The effects of OFS are believed to be driven through modulation of the gut microbiota, because it increases the prevalence of *Bifidobacteria* and *Lactobacillus,*  short-chain fatty acid production, resulting in increased circulating concentrations of a range of gut peptides [7, 27] [Cani, 2007].

Apple pectin is a complex polysaccharide, found in the cell walls of almost all terrestrial plants. Pectin is commonly used as a gelling and thickening agent in foods and was studied extensively in the 1980’s as a potential weight-loss agent [20, 21][Tiwary, 1997]. It may act in the gut both through a direct viscosity effect (slowing gastric emptying and improving satiety) and through fermentation to produce short-chain fatty acids. Pectin has the potential to act as a binding agent and reduce absorption of other drugs. There is a report that in 3 patients with hypercholesterolemia, concomitant use of pectin 15 g and lovastatin 80 mg daily reduced the effectiveness of lovastatin [Richter, 1991], suggesting that pectin may decrease the gastrointestinal absorption of lovastatin. For this reason, metformin pharmacokinetics were evaluated in healthy volunteers, at baseline and following 6 weeks of dosing, to determine whether co-administration of GSK2890457 had an effect.

Blackcurrant extract (BCE), an extract from the blackcurrant berry, has an anthocyanin content of greater than 25% and is marketed as a food additive and dietary supplement. Anthocyanins are credited with many of the beneficial effects that have been associated with berry consumption. Cyanidin-3-O-glucoside (C3G), one of the primary anthocyanins in blackcurrant extract, has been shown to have beneficial effects on insulin resistance and hepatic steatosis in nonclinical species [25, 34] [Tsuda, 2003], and it has been proposed as an anti-obesity agent [26]. Anthocyanins modify the gut microbiota in animal models and may be bitter receptor ligands.

Oleic acid is a monounsaturated omega-9 fatty acid (18:1n9) found in both plant and animal products. It is the principal fatty acid of olive oil and has been credited with many of the metabolic benefits seen with a Mediterranean diet. It stimulates gut peptide release through direct interaction with fatty-acid receptors in the gut and has been shown to have effects in reducing blood pressure [22, 23] [Anini, 1999].

The components of GSK2890457 used in the nonclinical and clinical studies were sourced as follows:

- Oleic Acid: Super Refined Oleic Acid NF-LQ-(MH) (Croda Inc, Mill Hall, PA, USA)
- Pectin: Classic AU201 USP Pectin (Herbstreith & Fox KG, Pektin-Fabriken, Turnstraβe Neuenbürg/Württ, Germany)
- Oligofructosaccharide: OFS Orafti (P95) (Beneo Orafti, Chillan, Chile)
- Blackcurrant Extract: Euro Black Currant PE, 25% Anthocyanins (CHR Hansen, Ciano d’Enza-Canossa [RE], Italy)

**References***Note: References that were cited both in the main article and the S1 Materials and Methods text are identified by number. References cited only in S1 Materials and Methods are listed alphabetically below.*

Anini Y, Fu-Cheng X, Cuber JC, Kervran A, Chariot J, Rozé C. Comparison of the postprandial release of peptide YY and proglucagon-derived peptides in the rat. *Eur J Physiol*.1999;438:299–306.

Cani PD, Hoste S, Guiot Y, Delzenne NM. Dietary non-digestible carbohydrates promote L-cell differentiation in the proximal colon of rats. *Br J Nutr.*2007; 98: 32–7.

Richter WO, Jacob BG, Schwandt P. Interaction between fibre and lovastatin. *Lancet*. 1991;338(8768):706.

Tiwary CM, Ward JA, Jackson BA. Effect of Pectin on Satiety in Healthy US Army Adults. *J Am Coll Nutr*. 1997;16(5):423-8.

Tsuda T, Horio F, Uchida K, Aoki H, Osawa T. Dietary Cyanidin 3-O-β-D-Glucoside-Rich Purple Corn Color Prevents Obesity and Ameliorates Hyperglycemia in Mice. *J Nutr.* 2003;133:2125–30.
