# Supplementary material for: Weight and Glucose Reduction Observed with a Combination of Nutritional Agents in Rodent Models Does Not Translate to Humans in a Randomized Clinical Trial with Healthy Volunteers and Subjects with Type 2 Diabetes
Source: PLoS One. 2016 Apr 19;11(4):e0153151. doi: 10.1371/journal.pone.0153151 (PMC4836696; doi:10.1371/journal.pone.0153151)
Supplement: S2 Materials and Methods — (DOCX) [file pone.0153151.s017.docx]

# S2 Materials and Methods

**Nonclinical – Further Information-- Animals**

Male diet-induced obese (DIO) C57BL/6NTac mice (N=8/group) fed a high-fat diet (60% fat by kcal) (D12492, Research Diets, New Brunswick, NJ, USA) from the time of weaning and age-matched lean controls, N=8 (Taconic, Germantown, NY, USA) were obtained at 19 weeks ± 3 days of age. All animals were drug-naïve and were habituated for a week or more before the start of the studies. At the start of administration og GSK457 or GRAS agents on Day -7, the DIO mouse mean body weight was 45.6 ± 0.1 g and lean mouse mean body weight was 29.6 ± 0.7 g. Male *db/db* (B6.Cg-m +/+ Lepr ^db^/J) (N=10/group) and *db/db* control mice (N=10) were obtained at 8 weeks ± 3 days of age, (Jackson Labs, Bar Harbor, ME, USA). At the start of GSK457 administration on Day -8, the *db/db* mouse mean body weight was 51.5 ± 0.3 g and % HbA1c was 7.5 ± 0.1, and the control mouse mean body weight was 28.3. ± 0.4 g and % HbA1c 4.4 ± 0.03. All mice were single-housed in polycarbonate shoebox cages with *ad libitum* access to water (Allentown Caging Equipment Co., Allentown, NJ, USA). The mice were maintained at 22°C and 50% relative humidity with 12-hour light/dark cycle (5 AM to 5 PM). Welfare-related assessments were made on a daily basis sues. DIO mice were given 45 kcal% fat (4.73kcal/g) pellet chow (Research Diets, New Brunswick, NJ, USA) and age-matched lean mice were fed 13.5 kcal% fat (3.02kcal/g) Lab Diet 5001, (PMI Nutrition International, Brentwood, MO, USA), while *db/db* and control mice were fed 16 kcal% fat (3.17kcal/g) 5K67 chow (LabDiet, St. Louis, MO, USA). All mice were given *ad libitum* access to chow, except for a pair-fed group in the *db/db* mouse study. For this group, pair-feeding was begun on Day 1, the day after the start of GSK457 treatment. Just before lights out, mice in the pair-fed group were given an amount of 25% Nutella^®^ chow to eat that was equal in kcal to the average amount of chow eaten during the previous 24 hours by the 10% GSK457 + Ex4 AlbudAb group.

**Preparation of Nonclinical Drug Treatments**

GSK457, a 5:5:2:3 mixture of oligofructosaccharide (OFS), apple pectin, blackcurrant extract (BCE) and oleic acid, was mixed into meal chows at 10 or 15% w/w with a Hobart Mixer (Hobart Corp., Troy, OH, USA). Nutella^®^ (Ferrero, Somerset, NJ, USA), 25% w/w, was included in the chow as a flavor-masker for all DIO and *db/db mice,* but not lean control mice, and the chow was provided in rodent chow jars, with weekly replacement. OFS was Orafti P95 from Beneo-Orafti, Inc. (Morris Plains, NJ, USA), apple pectin was from Solgar (Leonia, NJ, USA), oleic acid was from Sigma (St. Louis, MO, USA) and blackcurrant extract was from Cyvex Nutrition (Irvine, CA, USA).

Exendin-4-Albudab (Ex4 AlbudAb) dosing solutions were prepared by dilution into sterile, endotoxin-free 20 mM citrate buffer, pH 6.2 + 100 mM NaCl, aliquoted, maintained at -80°C, thawed and stored on ice within an hour of dosing. The effective dose for 20% of maximal effect in mice (ED20), 0.03 mg/kg, was used for all studies. A control AlbudAb which had no GLP-1R agonist activity was used as vehicle for the 14-day *db/db* mouse study while citrate buffer vehicle was used for the 28-day DIO mouse study (there was no difference in effect comparing citrate buffer and control AlbudAb).

**Nonclinical Drug Treatment**

The DIO and *db/db* mice were acclimated to 25% Nutella^®^ w/w, in D12451 or 5K67meal chow, respectively, for 7 days or more, and were dosed subcutaneously once with vehicle to acclimate them to handling stress. The DIO and *db/db* mice were fed 10 or 15% GSK457 (OFS, apple pectin, BCE, oleic acid in a 5:5:2:3 ratio) or single GRAS agents BCE, OFS, oleic acid or apple pectin, mixed into the Nutella^®^- supplemented chow, with vehicle control groups fed 25% Nutella^®^- supplemented chow, for the duration of the study. Chows were provided *ad* libitum in rodent chow jars and replaced weekly.

Exendin-4 AlbudAb was dosed subcutaneously (SC) (5ml/kg) in the loose skin on the back of the neck, every other day, 2-3 hours before lights out beginning 7-8 days after the start of GSK457/GRAS treatment. The designation 28-or 14-day study refers to the length of the GSK457 + exendin-4 combination treatment. Mice not receiving exendin-4 AlbudAb were dosed with a control AlbudAb or citrate buffer as described above.

**Nonclinical Food Consumption**

Chow jar weights (g) were measured at baseline to establish baseline food intake and then daily beginning when the GSK457 treatment was started. Starting on Day 1, just before lights out, the pair-fed mice were fed, an amount of food equal to the average food eaten on the previous day by the active treatment group.

Food intake (kcal) was calculated using the following kcal contents: BCE = 3.5; OFS = 1.5, apple pectin = 3.24, oleic acid = 10, Nutella^®^ = 5.41, D12451 chow = 4.73 kcal/g and 5K67 chow = 3.17 kcal/g. Cumulative food intake (kcal) was calculated for the start of GSK457 treatment.

**Nonclinical Serum Chemistry, Hormone, and Histopathology Analyses**

Beginning at 8-9 AM the morning after the final exendin-4 AlbudAb dose, after a 4‑5 hour fast, blood samples were collected by cardiac puncture under deep isoflurane (Piramal Healthcare Limited, Andhra Pradesh, India) anesthesia and the mice were euthanized by exsanguination. Plasma was prepared for analysis of a gastrointestinal hormone panel, and serum was used to evaluate clinical chemistry parameters. Liver was collected and fixed in 10% formalin for macroscopic and microscopic evaluation.

Plasma and serum were prepared using EDTA and T-MG tubes, respectively (Terumo Medical Corporation, Elkton, MD, USA). EDTA tubes used to prepare plasma for hormone analysis contained 50 μM DPP IV inhibitor (Millipore, St. Charles, MO, USA) and a protease inhibitor cocktail (Sigma-Aldrich, St. Louis, MO, USA). Clinical chemistry analysis was performed using an Olympus AU640 Clinical Chemistry analyzer (Olympus America, Inc., Melville, NY, USA) as described by the manufacturer of each assay. The following parameters were analyzed: glucose (GLU), total cholesterol (CHOL), HDL-cholesterol (HDL), triglycerides (TRIG), non-esterified fatty acids (NEFA), beta hydroxybutyrate (b-HBA), glycerol (GLY), aspartate aminotransferase (AST), alanine aminotransferase (ALT), Total Bilirubin (TBIL), blood urea nitrogen (BUN), creatinine (CREA), creatinine kinase (CK), total protein (TP), albumin (ALB), amylase (AMY), Lipase (LIP). Plasma hormone levels were analyzed using the Milliplex MAP Mouse Gut Hormone Panel (Catalog # MGT-78K-07) for insulin, amylin, leptin, ghrelin, pancreatic polypeptide (PP), glucose-dependent insulinotropic polypeptide (GIP) and peptide tyrosine tyrosine (PYY). MSD assays were used for measurement of active (Cat#: K150JWC-4) and total (Cat#: K150JVC-4) glucagon-like peptide-1 (GLP-1) (Meso Scale Discovery, Gaithersburg, MD, USA).
