# Supplementary material for: Weight and Glucose Reduction Observed with a Combination of Nutritional Agents in Rodent Models Does Not Translate to Humans in a Randomized Clinical Trial with Healthy Volunteers and Subjects with Type 2 Diabetes
Source: PLoS One. 2016 Apr 19;11(4):e0153151. doi: 10.1371/journal.pone.0153151 (PMC4836696; doi:10.1371/journal.pone.0153151)
Supplement: S3 Materials and Methods — (DOCX) [file pone.0153151.s018.docx]

**S3 Materials and Methods**

**Clinical – Further Information**

# Telemonitoring of Capillary Blood Glucose and Weight

The subjects in Parts B and C of the clinical study received a telemonitoring kit for home use (AMC Health NY, NY, USA) which included a weighing scale, a Bayer Contour^®^ glucometer and a modem, as well as an illustrated User Guide with step-by-step instructions. A toll-free helpdesk number was printed in the User Guide if subjects needed assistance with the telemonitoring setup.

Subjects were instructed as follows:

- Measure weight once a day, unclothed, first thing in the morning before breakfast.
- The subject must be the only person using the study scales.
- Use only the Bayer Contour^®^ glucometer provided for all glucose readings in the study (do not use own glucometer). Use Bayer Contour^®^ test strips only. The Bayer Microlet^®^ Lancet or Microlet^®^ 2 Lancing Device could be used.
- Call the clinical site if have any symptoms or readings of hypoglycemia or worried about their blood glucose level. Subjects were given cards with instructions on what to do in case of hypoglycemia.

Note: (i) The subjects left their weighing scale at home when attending the clinical unit, and a designated ‘study weighing scale’ was used at these visits; (ii) In the clinical unit, weight was measured in light clothing, without shoes.

# Metformin Assay

Plasma metformin concentrations were determined by PPD, Inc., Richmond, VA, USA. K2 EDTA plasma samples were analyzed for metformin concentrations using a validated method (PPD Method LCMS 153.5 V 2.00). All samples were shipped frozen on dry ice and stored at approximately -20 °C until analysed. Metformin and its internal standard, N,N-dimethyl-d6-diguanide, were isolated through solid phase extraction using a Waters Oasis WCX 10-mg, 96-well SPE plate and are eluted with 350 μL of 2:98 formic acid / acetonitrile, v/v. The eluate was then further diluted with 1.00 mL of 2:98 formic acid / acetonitrile vol/vol, prior to being analyzed by HPLC with MS/MS detection. The assay was validated over the metformin concentration range of 2.00 to 2000 ng/mL in human K2 EDTA plasma.

# Liraglutide Assay

Plasma liraglutide concentrations were determined by PPD, Inc, Richmond, VA, USA. K2 EDTA plasma samples were analyzed for liraglutide concentrations using a validated method (PPD Method LCMSD 631 V 1.00). All samples were shipped on dry ice and were stored frozen at -70 °C until analysed. Liraglutide and its internal standard, Liraglutide-13C16, were isolated through protein precipitation using 5:25:75:1 TFE / ethanol / acetonitrile / formic acid. After centrifugation, the supernatant was directly analyzed by HPLC with MS/MS detection using positive ion electrospray. The assay was validated over the liraglutide concentration range of 1.00 to 500 ng/mL in human K2 EDTA plasma.
