# Supplementary material for: Weight and Glucose Reduction Observed with a Combination of Nutritional Agents in Rodent Models Does Not Translate to Humans in a Randomized Clinical Trial with Healthy Volunteers and Subjects with Type 2 Diabetes
Source: PLoS One. 2016 Apr 19;11(4):e0153151. doi: 10.1371/journal.pone.0153151 (PMC4836696; doi:10.1371/journal.pone.0153151)
Supplement: S1 Nonclinical Results — (DOCX) [file pone.0153151.s019.docx]

# S1 Nonclinical Results

**Chronic 28-day Treatment of DIO Mice with 15% (w/w in chow) GSK457 in Combination with Exendin-4 AlbudAb**

**Improvement of Serum Chemistry and Hormone Panel Parameters**:

BCE, OFS, apple pectin and oleic acid dosed as single agents had only a small beneficial effect. For example, vs. DIO vehicle, BCE and oleic acid lowered glucose and BCE and apple pectin reduced cholesterol levels (p < 0.05) (S1 Table). Also, each agent tended to lower ALT and AST. GSK457 and the GSK457 + exendin-4 AlbudAb combination groups displayed many significant changes in serum chemistries, all of which reflect the transition from the pathological state of obesity to the normal lean state. For example, treatment with GSK457 + exendin-4 AlbudAb reduced serum glucose by 22% to the level of lean controls and cholesterol was lowered by 42% (Fig. 5; Table 1). AST and ALT were elevated in the vehicle control DIO mice due to the presence of diet-induced hepatic steatosis, but treatment with the GSK457 + exendin-4 AlbudAb combination decreased levels by 81% and 73%, respectively, to those of the lean controls (S3 Fig.).

BCE, OFS, apple pectin and oleic acid had little effect on endocrine hormones when dosed individually (S2 Table). However, hormone profiles improved with GSK457 alone and the GSK457 + exendin-4 AlbudAb combination, with the combination tending to have greater effect on insulin, amylin and leptin, commensurate with the greater weight loss. With the GSK457 + exendin-4 AlbudAb combination, plasma total GLP-1 levels were significantly increased when compared to DIO controls (p<0.05) and plasma PYY levels were trending higher (p=0.0726). Further, insulin levels were significantly lower in the GSK457 + exendin-4 AlbudAb combination group and not statistically significant from lean controls (p = 0.3328). Reduction of the plasma amylin levels in the GSK457 + exendin-4 AlbudAb combination group was commensurate with the insulin reduction, and values were comparable to those of to the lean controls (p = 0.3491). Consistent with the fat mass changes, leptin levels in the GSK457 + exendin-4 AlbudAb combination group were lowered significantly relative to vehicle DIO animals (p<0.05) and were near lean control values (p=0.4755).

**Improvement in Liver Steatosis**:

Compared to lean control animals there was a marked increase in cytoplasmic lipid droplets in the liver (affecting most hepatocytes), confirmed by osmium staining, in the DIO vehicle control mice. The cytoplasmic lipid droplets were substantially decreased (minimal to undetectable) in DIO mice given the combination of GSK457 and exendin-4 AlbudAb (S4 Fig.).
