# Supplementary material for: Weight and Glucose Reduction Observed with a Combination of Nutritional Agents in Rodent Models Does Not Translate to Humans in a Randomized Clinical Trial with Healthy Volunteers and Subjects with Type 2 Diabetes
Source: PLoS One. 2016 Apr 19;11(4):e0153151. doi: 10.1371/journal.pone.0153151 (PMC4836696; doi:10.1371/journal.pone.0153151)
Supplement: S2 Nonclinical Results — (DOCX) [file pone.0153151.s020.docx]

# S2 Nonclinical Results

**Chronic 14-day Treatment of *db/db* Mice with 10% GSK457 and the exendin-4 AlbudAb.**

**Reductions in Body Weight, Fat Mass and Food Intake:**

Treating with either 10% GSK457 or exendin-4 AlbudAb alone marginally inhibited weight gain relative to vehicle controls (not statistically significant, S5 Fig.). The combination of 10% GSK457 and exendin-4 AlbudAb produced a 7.4% reduction in body weight relative to vehicle control, while, the pair-fed group for the 10% GSK457 and exendin-4 AlbudAb combination showed a greater reduction in weight of 13.0% (p<0.05) relative to vehicle controls (S5 Fig., Panel A). The greater weight loss in the pair-fed group relative to the GSK457 + exendin-4 AlbudAb combination group can be ascribed in part to significant non-fat-mass loss. Thus, the weight loss produced by the GSK457 + exendin-4 AlbudAb combination is primarily achieved by a reduction in fat-mass (S5 Fig., Panel B). The combination of 5% GSK457 + exendin-4 AlbudAb did not affect weight, glucose, HbA1c, serum chemistry, or hormone panels compared to vehicle controls.

A significant reduction in cumulative food intake were observed with the 10% GSK457 and exendin-4 AlbudAb combination, relative to vehicle controls, which started on Day 8 of the treatment (S6 Fig.). The combination of 10% GSK457 and exendin-4 AlbudAb significantly reduced cumulative food intake by 25% for the period of Day -8 to 14 (S6B Fig.). GSK457 at 10% alone did not change cumulative food intake compared to vehicle controls.

**Improvement in Serum Chemistry Parameters, Hormones, and Liver Steatosis:**

The 10% GSK457 and exendin-4 AlbudAb combination resulted in normalization of clinical chemistry parameters (e.g. glucose, HbA1c, cholesterol, triglycerides, AST, ALT) back to lean control values (Table S3). Histopathological analysis of the liver confirmed that the hepatic steatosis was markedly reduced and hepatocyte appearance was normalized in the *db/db* mice treated with the GSK457 and exendin-4 AlbudAb combination.
