# Supplementary material for: Weight and Glucose Reduction Observed with a Combination of Nutritional Agents in Rodent Models Does Not Translate to Humans in a Randomized Clinical Trial with Healthy Volunteers and Subjects with Type 2 Diabetes
Source: PLoS One. 2016 Apr 19;11(4):e0153151. doi: 10.1371/journal.pone.0153151 (PMC4836696; doi:10.1371/journal.pone.0153151)
Supplement: S1 Protocol — (PDF) [file pone.0153151.s021.pdf]

TITLE PAGE

Division: Worldwide Development
Retention Category: GRS019
Information Type: Protocol Amendment

**Title:**	A double-blind [sponsor unblinded], randomized, placebo-controlled, staggered-parallel study to investigate the safety, tolerability, and pharmacodynamics of GSK2890457 in healthy volunteers and subjects with type 2 diabetes

Compound Number: GSK2890457

Effective Date: 28-MAY-2013

Protocol Amendment Number: 02

Subject: [diabetes, obesity, GLP-1, metformin, liraglutide]

Author(s):

[redacted] CPSSO, Metabolic Pathways
EE Discovery Medicine, Metabolic Pathways and CV Unit
EE Discovery Medicine, Metabolic Pathways and CV Unit
CPMS, Clinical Pharmacology Modeling and Simulation
Clinical Statistics – Metabolic
Drug Metabolism and Pharmacokinetics
CPSSO, Metabolic Pathways

Copyright 2013 the GlaxoSmithKline group of companies. All rights reserved. Unauthorised copying or use of this information is prohibited.

Revision Chronology:

2012N137064_00	2012-AUG-24	Original
2012N137064_01	2013-JAN-03	Amendment No.: 01 This amendment changes (i) the blood glucose definition of hypoglycaemia in Parts B and C to <70 mg/dL, (ii) adds blood glucose measurement pre-dinner in Parts B and C, (iii) clarifies acceptable surgical sterilization procedures, (iv) clarifies procedures to be followed if a pregnancy occurs during the study; (v) provides more flexibility in the calorie content of standardized breakfast meals, (vi) clarifies the procedures that are required to re-screen subjects who have fulfilled inclusion/exclusion requirements at an earlier visit, (vii) adds more information on the preparation of GSK2890457 and how to handle spills, (viii) adds the potential risk of upper respiratory and ocular irritation from powders in GSK2890457, (ix) excludes subjects with significant hypoglycaemia unawareness, (x) adds conditional wording relating to deuterated creatine procedures in case deuterated creatine is not available, and (xi) corrects minor inconsistencies and typographical and omission errors.

2012N137064_02	2013-MAY-28	Amendment 2 adds that instructions will be given to subjects on how to manage hypoglycemia. Papillary thyroid cancer is added to the risks of liraglutide use. Guidance is given for management of subjects who are not using the telemonitoring equipment correctly. Allowance for flexibility around the time of day that subjects may come in for scheduled visits, allowing for non-fasted sampling, as long as it is noted in the case report form. In response to preliminary data from Part A, metformin in Part C will be dosed one hour prior to GSK2890457. Clarification that doses of GSK2890457 are to be taken before breakfast and dinner. Additional language allowing for dose reduction based on safety and/or tolerability issues. Additional information about proceeding with subjects' normal dose of metformin in Part C, by separating it by 1 hour from the GSK2890457. The time interval between the GSK2890457 and liraglutide doses can be adjusted to improve the ability to take GSK2890457. Additional details about fasting glucose sampling being conducted by the subject at home. Visit window added for visit Days 4 through 35 in Parts B and C (± 1 day). Section 4.7.4 corrected to reflect dispensing of study drug on Day 1 (with randomization). Section 4.7.7 added to show the updated blood sampling scheme and metformin dosing for Part B. C-Peptide measurements added to table in Section 7.3.5. Modified description of the fecal sample collection tube.

SPONSOR SIGNATORY:

[redacted]

28 May 2013

Date

[redacted] MA, MD, FRCP
Director and Head
EnteroEndocrine Discovery Medicine
Metabolic Pathways and Cardiovascular Unit

[redacted]

[redacted]

[redacted]

[redacted]

SPONSOR/MEDICAL MONITOR INFORMATION PAGE

Medical Monitor and Sponsor Contact Information:

Role	Name	Day Time Phone Number & Email	After-hours Phone/Cell/ Pager Number	Fax Number	GSK Address
Primary Medical Monitor	[redacted]	[redacted]	[redacted]	[redacted]	[redacted] 5 Moore Drive RTP, NC 27709
Secondary Medical Monitor	[redacted]	[redacted]	[redacted]	[redacted]	[redacted] 5 Moore Drive RTP, NC 27709
Tertiary Medical Monitor	[redacted]	[redacted]	[redacted]	[redacted]	[redacted] 5 Moore Drive RTP, NC 27709

GlaxoSmithKline Research & Development Limited
980 Great West Road
Brentford
Middlesex, TW8 9GS
UK

In some countries, the clinical trial sponsor may be the local GlaxoSmithKline affiliate company (or designee). If applicable, the details of the alternative Sponsor and contact person in the territory will be provided to the relevant regulatory authority as part of the clinical trial application.

Regulatory Agency Identifying Number(s): IND 114,161

INVESTIGATOR PROTOCOL AGREEMENT PAGE

I confirm agreement to conduct the study in compliance with the protocol, as amended by this protocol amendment.

Investigator Name:	
Investigator Address:	
Investigator Phone Number:	
Investigator Signature	Date

TABLE OF CONTENTS

PAGE

ABBREVIATIONS 12

1. INTRODUCTION 16
   1.1. Background 16
   1.2. Rationale 17
      1.2.1. Study Rationale 17
      1.2.2. Dosing Rationale 17
   1.3. Summary of Risk Management 18
      1.3.1. Potential Risks of GSK2890457 and Their Mitigation 18
      1.3.2. Potential Risks of Liraglutide, Alone and Co-Administered with GSK2890457 and Their Mitigation 18
      1.3.3. Potential Risks of Co-administration of Metformin and GSK2890457 and Their Mitigation 23
      1.3.4. Potential Risks of Deuterated Creatine and Their Mitigation 27

2. OBJECTIVE(S) 27
   2.1. Primary Objectives 27
   2.2. Secondary Objective 28
   2.3. Exploratory Objectives 28

3. ENDPOINT(S) 28
   3.1. Primary Endpoints 28
   3.2. Secondary Endpoints 28
   3.3. Exploratory Endpoints 29

4. INVESTIGATIONAL PLAN 29
   4.1. Part A 29
   4.2. Part B 30
      4.2.1. Run-in Period (1 week) 30
      4.2.2. Stabilization Period (12 weeks) 31
      4.2.3. Treatment Period (6 weeks) 32
   4.3. Part C 34
      4.3.1. Run-in Period (1 week) 34
      4.3.2. Stabilization Period (4 weeks) 34
      4.3.3. Treatment Period (6 weeks) 35
   4.4. Dosage and Administration 37
      4.4.1. GSK2890457 Dosage and Administration (Parts A-C) 37
      4.4.2. Liraglutide (Part B) 39
      4.4.3. Metformin 39
         4.4.3.1. Metformin (Part A) 39
         4.4.3.2. Metformin (Part C) 39
   4.5. Investigational Product and Other Study Treatment Dosage/Administration 41
   4.6. Dose Adjustment/Stopping Criteria 42
      4.6.1. Dose Adjustment/Stopping Safety Criteria 42
         4.6.1.1. Liver Chemistry Stopping Criteria 42
         4.6.1.2. QTc Withdrawal Criteria 43

4.6.1.3. Withdrawal Criteria for Suspected Acute Pancreatitis (Part B) 43
4.6.1.4. Dose Adjustment/Stopping Safety Criteria Based on Blood Glucose Monitoring (Part B and C) 44
4.6.1.4.1. Part A (Healthy Subjects) 45
4.6.1.4.2. Part B (Liraglutide Add-on) 46
4.6.1.4.3. Part C (Metformin Add-on) 46
4.6.1.5. Withdrawal Criteria for Renal Impairment 46
4.6.1.6. Dose Adjustment Based on Reduced Food Intake 46
4.6.1.7. Monitoring and Stopping Criteria for Allergic Reactions to Liraglutide 46
4.6.1.8. Stopping Criteria for Suicidal Ideation 47
4.6.1.9. Other Dose Adjustment and Stopping Criteria for Safety 47
4.6.1.10. Subject Withdrawal for Other Reasons 47
4.7. Time and Events Tables 48
4.7.1. Part A Time and Events Table – Healthy Subjects 48
4.7.2. Part B Time and Events Table: Screening, 1-Week Telemonitoring Run-in and 12-Week Stabilization 50
4.7.3. Part C Time and Events Table: Screening, 1-Week Telemonitoring Run-in and 4-Week Stabilization for Subjects Remaining on Metformin 52
4.7.4. Parts B and C Time and Events Table Day -2 through Study End 54
4.7.5. Part A: Day 1 and Day 42: Sampling Schedule for Metformin PK Timepoints 56
4.7.6. Part B: Day -1 and Day 42: Sampling Schedule for Glucose, Insulin, Biomarkers, and PK; Satiety Questionnaire Timepoints 57
4.7.7. Part C: Day -1 and Day 42: Sampling Schedule for Glucose, Insulin, Biomarkers, and PK; Satiety Questionnaire Timepoints 59

5. STUDY POPULATION 61
5.1. Eligibility Criteria 61
5.1.1. Screening Inclusion Criteria for Part A (Healthy Subjects) 61
5.1.2. Screening Exclusion Criteria for Part A (Healthy Subjects) 62
5.1.3. Screening Inclusion Criteria for Type 2 Diabetic Subjects (Parts B and C) 65
5.1.4. Screening Exclusion Criteria for Type 2 Diabetic Subjects (Parts B and C) 67
5.2. Screen and Run-in Failures 70

6. DATA ANALYSIS AND STATISTICAL CONSIDERATIONS 70
6.1. Hypotheses and Treatment Comparisons 70
6.2. Sample Size Considerations 71
6.2.1. Sample Size Assumptions 71
6.2.2. Sample Size Sensitivity 71
6.2.3. Sample Size Re-estimation 71
6.3. Data Analysis Considerations 71

6.3.1. Interim Analysis 71
6.3.2. Final Analyses 71
6.3.2.1. Safety Analyses 71
6.3.2.2. Pharmacokinetic Analyses 72
6.3.2.3. Pharmacodynamic Analyses 73
6.3.2.4. Biomarker Analyses 74
6.3.2.5. Exploratory Analyses 74
6.3.2.6. Pharmacodynamic/Biomarker Analyses (Part A will include a subset of these) 74
6.3.2.7. Novel and Other Biomarker Analyses in Parts B and C 75

7. STUDY ASSESSMENTS AND PROCEDURES 76
7.1. Demographic/Medical History Assessments 76
7.2. Safety 77
7.3. Physical Exams 77
7.3.1. Vital Signs 77
7.3.2. Electrocardiogram (ECG) 77
7.3.3. Weight 78
7.3.4. Waist Circumference 78
7.3.5. Clinical Laboratory Assessments 78
7.3.6. Re-Screening 79
7.3.7. GI Symptom Rating Scale (GSRS) 80
7.4. Pregnancy 80
7.4.1. Time Period for Collecting Pregnancy Information 80
7.4.2. Action to be Taken if Pregnancy Occurs 81
7.5. Pharmacokinetics 81
7.5.1. Blood Sample Collection 81
7.5.1.1. Blood Samples for Metformin or Liraglutide PK 81
7.5.1.2. Blood Samples for GSK2890457 PK 82
7.5.2. Urine Sample Collection 82
7.5.3. Sample Analysis 82
7.6. Biomarker(s)/Pharmacodynamic Markers 83
7.6.1. Confirmed Pharmacodynamic Markers (Parts B and C) 83
7.6.1.1. Glucose and Insulin 83
7.6.1.2. Other Biomarkers 83
7.6.1.3. Telemonitoring of Weight and CBG 83
7.6.1.4. Fecal Microbiome Analysis 83
7.6.2. Exploratory Biomarkers (Parts B and C) 83
7.6.2.1. Other Exploratory Biomarkers 83
7.6.2.2. Metabolomic Research 84
7.6.2.3. Proteome/Peptide Research 84
7.6.3. Blood Sample Collection for Biomarkers 84
7.6.3.1. Blood Sample Collection for Glucose and Insulin 84
7.6.3.2. Blood Sample Collection for Other Biomarkers 85
7.6.3.3. Blood Sample Collection for Fasting Exploratory Biomarkers 85
7.6.4. Deuterated Creatine Method for Measuring Total Skeletal Muscle Mass 85
7.6.5. Hunger Questionnaire 86

8. LIFESTYLE AND/OR DIETARY RESTRICTIONS 86
8.1. Contraception Requirements 86
8.1.1. Female Subjects 86
8.2. Meals and Dietary Restrictions 86
8.2.1. Standard Test Meals 86
8.2.2. Caffeine, Alcohol, and Tobacco 87
8.3. Activity 87

9. CONCOMITANT MEDICATIONS AND NON-DRUG THERAPIES 88
9.1. Part A – Healthy Subjects 88
9.1.1. Permitted Medications 88
9.1.2. Prohibited Medications 88
9.1.3. Non-Drug Therapies 88
9.2. Parts B and C – T2D Subjects 89
9.2.1. Permitted Medications 89
9.2.2. Prohibited Medications 89
9.2.3. Non-Drug Therapies 90
9.3. Anti-Viral Therapies for Influenza (Parts A, B and C) 90

10. COMPLETION OR EARLY WITHDRAWAL OF SUBJECTS 91
10.1. Subject Completion 91
10.2. Subject Withdrawal Criteria 91
10.2.1. Parts B and C Glucose Withdrawal Criteria 91
10.3. Subject Withdrawal Procedures 92
10.3.1. Subject Withdrawal from Study 92
10.3.2. Subject Withdrawal from Study Treatment 92
10.4. Treatment After the End of the Study 92

11. STUDY TREATMENT 92
11.1. Blinding 92
11.2. Packaging and Labeling 93
11.3. Preparation/Handling/Storage/Accountability 93
11.4. Assessment of Compliance 94
11.5. Treatment of Study Treatment Overdose 94
11.5.1. GSK2890457 Overdose 94
11.5.2. Metformin Overdose 94
11.5.3. Victoza (Liraglutide) Overdose 95

12. ADVERSE EVENTS (AE) AND SERIOUS ADVERSE EVENTS (SAE) 95
12.1. Definition of Adverse Events 95
12.2. Definition of Serious Adverse Events 96
12.3. Method of Detecting AEs and SAEs 97
12.4. Recording of AEs and SAEs 98
12.5. Evaluating AEs and SAEs 98
12.5.1. Assessment of Intensity 98
12.5.2. Assessment of Causality 99
12.6. Follow-up of AEs and SAEs 99
12.7. Prompt Reporting of SAEs to GSK 99
12.8. Regulatory Reporting Requirements for SAEs 100

13. LIVER CHEMISTRY FOLLOW-UP PROCEDURES 100

14. STUDY CONDUCT CONSIDERATIONS 102
14.1. Posting of Information on Publicly Available Clinical Trial Registers 102
14.2. Regulatory and Ethical Considerations, Including the Informed Consent Process 102
14.3. Quality Control (Study Monitoring) 103
14.4. Quality Assurance 103
14.5. Study and Site Closure 104
14.6. Records Retention 104
14.7. Provision of Study Results to Investigators, Posting of Information on Publically Available Clinical Trials Registers and Publication 105
14.8. Data Management 105

15. REFERENCES 106

APPENDICES 107
Appendix 1: Liver Safety Algorithms 107
Appendix 2: Hunger Questionnaire 108
Appendix 3: GI Symptom Rating Scale (GSRS) Questionnaire 109
Appendix 4: Background and End-of-Study Questions 115
Appendix 5: Directions for Taking Study Medicine 116
Appendix 6: Mindful Eating Guide 120
Appendix 7: Victoza (Liraglutide) Medication Guide for Patients 121
Appendix 8: Country Specific Requirements 122
Appendix 9: Protocol Amendment Changes 123

ABBREVIATIONS

ACE	Angiotensin converting enzyme
AE	Adverse Event
ALT	Alanine aminotransferase (SGPT)
ANCOVA	Analysis of covariance
ANOVA	Analysis of Variance
AST	Aspartate aminotransferase (SGOT)
AUC	Area under concentration-time curve
AUCss	Area under concentration-time curve at steady state
AUC(0-∞)	Area under the concentration-time curve from time zero (pre-dose) extrapolated to infinite time
%AUCex	Percentage of AUC(0-∞) obtained by extrapolation
AUC(0-x)	Area under the concentration-time curve from zero (pre-dose) to some fixed nominal time x
AUC(0-t)	Area under the concentration-time curve from time zero (pre-dose) to last time of quantifiable concentration within a subject across all treatments
AUC(0-τ)	Area under the concentration-time curve over the dosing interval
β-HCG	Beta-Human Chorionic Gonadotropin
BID	Twice daily
BMI	Body mass index
BP	Blood pressure
BPM	Beat Per Minute
BQL	Below the quantification limit
BUN	Blood urea nitrogen
CBC	Complete blood count
CBG	Capillary Blood Glucose
CI	Confidence Interval
CIB	Clinical Investigator's Brochure
CLr	Renal clearance
CL	Systemic clearance of parent drug
CL/F	Apparent clearance following oral dosing
Cmax	Maximum observed concentration
Cmin	Minimum observed concentration
CNS	Central nervous system
CO_2	Carbon dioxide
CPK	Creatine phosphokinase
CPMS	Clinical Pharmacokinetics Modeling & Simulation
CRF	Case Report Form
CRP	C-reactive protein
C-SSRS	Columbia Suicide-Severity Rating Scale
CV	Coefficient of variance
DEXA	Dual-energy x-ray absorptiometry
DIO	Diet induced obesity
DMPK	Drug Metabolism and Pharmacokinetics
DNA	Deoxyribonucleic acid

DPP-IV	Dipeptidyl peptidase-IV
ECG	Electrocardiogram
FDA	Food and Drug Administration
FSH	Follicle Stimulating Hormone
FT/MS	Fourier transform mass spectrometry
GC-MS	Gas chromatography-mass spectrometry
GCP	Good Clinical Practice
GCSP	Global Clinical Safety and Pharmacovigilence
GFR	Glomerular filtration rate
GGT	Gamma glutamyltransferase
GI	Gastrointestinal
GLP	Good Laboratory Practice
GLP-1	Glucagon-like peptide-1
GLP-2	Glucagon-like peptide-2
GSK	GlaxoSmithKline
GSRS	Gastrointestinal (GI) Symptom Rating Scale
HBsAg	Hepatitis B surface antigen
hCG	Human chorionic gonadotropin
HDL	High Density Lipoprotein
HIV	Human Immunodeficiency Virus
h/hr	Hour(s)
HOMA-IR	Homeostasis model of assessment – insulin resistance
HPLC	High Pressure/Performance Liquid Chromatography
HR	Heart rate
HRT	Hormone replacement therapy
IB	Investigator’s Brochure
ICH	International Conference on Harmonization of Technical Requirements for Registration of Pharmaceuticals for Human Use
IDSL	Integrated Data Standards Library
IEC	Independent Ethics Committee
IL-6	Interleukin-6
IND	Investigational New Drug
INR	International normalized ratio
IP	Investigational Product
IR	Immediate release
IRB	Institutional Review Board
Kg	Kilogram
L	Liter
LC-MS	Liquid chromatography-mass spectrometry
LDH	Lactate dehydrogenase
LDL	Low Density Lipoprotein
LFTs	Liver function tests
LOQ	Limit of quantification
LLQ	Lower limit of quantification
LSLV	Last subject’s last visit
µg	Microgram

μL	Microliter
MCH	Mean corpuscular hemoglobin
MCHC	Mean corpuscular hemoglobin concentration
MCV	Mean corpuscular volume
MDRD	Modification of Diet in Renal Disease
MedDRA	Medical Dictionary for Regulatory Activities
Mg	Milligrams
mL	Milliliter
MRI	Magnetic resonance imaging
MS	Mass spectrometry
MSDS	Material Safety Data Sheet
msec	Milliseconds
NAFLD	Nonalcoholic fatty liver disease
NCBP	Non-childbearing potential
NMR	Nuclear magnetic resonance
NOAEL	No observed adverse effect level
OTC	Over the counter
PD	Pharmacodynamic
PGx	Pharmacogenetics
PK	Pharmacokinetic
PYY	Peptide tyrosine-tyrosine
QC	Quality control
QD	Once daily
QTcB	QT duration corrected for heart rate by Bazett's formula
QTcF	QT duration corrected for heart rate by Fridericia's formula
RAP	Reporting and Analysis Plan
RBC	Red blood cells
RNA	Ribonucleic acid
SAE	Serious adverse event(s)
SAS	Statistical Analysis Software
SD	Standard deviation
SGOT	Serum glutamic-oxaloacetic transaminase
SGPT	Serum glutamic pyruvic transaminase
SOP	Standard Operating Procedure
SPM	Study Procedures Manual
t	Time of last observed quantifiable concentration
τ	Dosing interval
tlag	Lag time before observation of drug concentrations in sampled matrix
tlast	Time of last quantifiable concentration
tmax	Time of occurrence of Cmax
TNFα	Tumor necrosis factor alpha
TSH	Thyroid stimulating hormone
ULN	Upper limit of normal
UK	United Kingdom
US	United States

Vd/F	Apparent volume of distribution after extravascular (e.g., oral) administration
WBC	White blood cells

Trademark Information

Trademarks of the GlaxoSmithKline group of companies
RELENZA

Trademarks not owned by the GlaxoSmithKline group of companies
Chiron RIBA
Glucophage
InForm
Kaopectate
MiO
Pepto-Bismol
SAS
Tamiflu
Victoza
WinNonlin

1. INTRODUCTION

1.1. Background

GSK2890457 is a novel oral agent in development for the treatment of obesity in patients with Type 2 diabetes (T2D) who are taking a glucagon-like peptide-1 (GLP-1) mimetic and/or metformin. GSK2890457 is a mixture of four different components that are food ingredients. In the diet-induced obese (DIO) mouse, a model of obesity, GSK2890457 demonstrates synergistic efficacy when co-administered with a GLP-1 mimetic, returning animals to lean control body weight. In the *db/db* mouse, a leptin-receptor deficient model of diabetes, effects on glucose levels are also greater than additive when GSK2890457 and a GLP-1 mimetic are combined. Because metformin also increases circulating levels of active GLP-1, it has the potential to show synergy with GSK2890457. Unfortunately, metformin efficacy in animal models is not fully predictive of efficacy in human, and so the concomitant administration of metformin and GSK2890457 has not been tested in preclinical models, but will be explored in this initial human study.

GSK2890457 works through multiple mechanisms. All four components stimulate endogenous release of one or more gut peptide hormones (GLP-1 and peptide tyrosine-tyrosine [PYY], and probably GLP-2 and/or oxyntomodulin). It appears that the GSK2890457 components function by direct receptor stimulation or indirectly by conversion to short-chain fatty acids by gut microflora, which subsequently stimulate peptide release from enteroendocrine cells. It is likely that there are additional effects that confer metabolic benefit. In the DIO model of obesity, GSK2890457 demonstrates weight-loss efficacy that is significantly greater than the sum of effects of the individual components, and these effects are further augmented by co-administration of a GLP-1 mimetic. In the non-clinical studies, weight loss appears to be driven primarily by decreased food intake, even though animals continue to have unlimited access to a high-fat diet. However, it appears that the effects on blood glucose control may be greater than those expected from weight loss.

It is important to ensure that weight loss is driven primarily by loss of fat and not lean mass. Accurate measurement of total skeletal muscle mass is confounded either by the complexity of available methods (e.g., quantification of total muscle protein and imaging by computerized axial tomography and magnetic resonance imaging), or by the indirect nature of the estimations, as in the case of dual-energy x-ray absorptiometry (DEXA). In this study, a new exploratory method may be evaluated against the DEXA method of measuring skeletal muscle mass. This involves the estimation of the total creatine pool size because >95% of the total creatine pool is in skeletal muscle. The creatine is converted non-enzymatically to creatinine and excreted in urine. After oral administration of deuterated (non-radioactive, stable-label) creatine, measurement of the amount of deuterated creatinine and deuterated and non-deuterated creatinine and creatine, excreted in the urine allows estimation of total skeletal muscle mass.

1.2. Rationale

1.2.1. Study Rationale

This study investigates the first administration of GSK2890457 to humans, although each of its four components has an extensive history of consumption by humans as single agents.

The study will be conducted in 3 parts:

- Part A (to be conducted at a single investigative site) will determine the safety and tolerability of GSK2890457 alone in healthy subjects during six weeks of dosing, as well as evaluating the potential for a pharmacokinetic interaction with metformin.
- Part B will determine safety, tolerability, and pharmacodynamics (PD) in subjects with T2D when co-dosed for six weeks with liraglutide (Victoza), a GLP-1 mimetic that is approved for the treatment of T2D (VICTOZA, 2012 Prescribing Information and Medication Guide). T2D subjects on metformin monotherapy will have a 'Stabilization Period' of 12 weeks on liraglutide (after stopping their metformin therapy) for assessment of baseline PD parameters before co-dosing with GSK2890457. Part B will be conducted at multiple sites. The main PD endpoints will be weight and glucose/insulin changes during the 6-week co-dosing Treatment Period.
- Part C will determine safety, tolerability, and PD in subjects with T2D subjects when co-dosed for six weeks with metformin [GLUCOPHAGE (Metformin) Prescribing Information]. T2D subjects on metformin monotherapy will have a 'Stabilization Period' of one month on their usual dose of metformin for assessment of baseline PD parameters before co-dosing with GSK2890457. Part C will be conducted at multiple sites. The main PD endpoints for Part C will be weight and glucose/insulin changes during the 6-week co-dosing Treatment Period.

An exploratory deuterated (non-radioactive, stable-label) creatine method for measuring skeletal muscle mass will be assessed in Parts B and C if deuterated creatine is available by the start time of the Treatment period.

1.2.2. Dosing Rationale

The GSK2890457 dose selection for this study has been empirically based on available published human data for the individual agents. The maximal planned dose of 40g is further supported by data from a 6-week rat toxicity study which have demonstrated that there are no test-article related findings, and defined the NOAEL for GSK2890457 as >8000mg/kg/day. See Investigators Brochure [GlaxoSmithKline Document Number 2012N141885_00] for further details.

Subjects will initiate therapy by taking 15g of GSK2890457 daily, in divided doses, and will titrate up to 30g daily, with a further titration over 7 days to a maximum dose of 40g daily. Subjects unable to tolerate doses of 30g or 40g will be permitted to reduce their dose to tolerated levels, with a minimum tolerated dose of 15g required to remain in the study.

If tolerability of the 40g dose proves to be a significant issue in healthy subjects in Part A, the maximal dose used in T2D subjects in Parts B and C may be reduced to 30g daily, and the titration steps may be modified accordingly.

1.3. Summary of Risk Management

1.3.1. Potential Risks of GSK2890457 and Their Mitigation

GSK2890457 has not been administered to humans, but each of the four components of GSK2890457 has a history of human use without significant adverse effects. Additional details relating to prior use of the individual components can be found in the Investigator's Brochure [GlaxoSmithKline Document Number 2012N141885_00].

Table 1 Potential Risks of GSK2890457 and Their Mitigation

Potential Risk	Background	Mitigation
Flatulence, borborygmi, intestinal cramping	Two of the components of GSK2890457 are known to cause dose-dependent gastrointestinal symptoms when administered as single agents. Most individuals develop tolerance over time, but responses may vary.	The dose of GSK2890457 will be titrated gradually over 7 days, and subjects will have the option of decreasing the dose (within protocol-defined limits) if tolerability is unacceptable.
Discoloration of the mouth, teeth, stool and/or urine	One of the components of GSK2890457 is highly pigmented, and animals receiving very high doses have shown variable coloration of the urine and darkening of the stool.	Doses administered to humans are not anticipated to cause coloration of stools or urine, but the teeth and oral cavity may be temporarily discoloured until they are washed. Subjects will be advised that this discoloration, if observed, is harmless.
Hypoglycemia	In animal efficacy studies, GSK2890457 has reduced elevated glucose levels to normal when used in combination with a GLP-1 mimetic – no hypoglycaemia has been observed. In these studies, insulin levels were either significantly reduced or showed non-significant elevations. This is consistent with the potential mechanisms of action of GSK2890457 which are believed to be effective only when glucose levels are elevated. Nevertheless, a risk of hypoglycemia in T2D subjects cannot be excluded when co-dosed with other anti-diabetic medications.	Subjects with T2D will monitor fasting and pre-dinner capillary blood glucose (CBG) values daily using a telemonitored glucometer, and will be advised to report any symptoms of hypoglycemia promptly.<br><br>Subjects will be given instructions on how to handle hypoglycaemia.
Contact exposure and/or inhalation of fine powder	The powders in this formulation of GSK2890457 may cause upper respiratory tract and/or ocular irritation in subjects who are sensitive to environmental dusts or allergens.	Exclude subjects with a past history of (i) sensitivity to environmental dusts or allergens, (ii) asthma or (iii) inhaler use.

1.3.2. Potential Risks of Liraglutide, Alone and Co-Administered with GSK2890457 and Their Mitigation

In Part B, T2D subjects on prior metformin monotherapy will be switched to liraglutide for a 12 week Stabilization Period before entering the 6-week Treatment Period. At the end of the Treatment Period, subjects will be switched back to their usual dose of metformin, unless, in the judgment of the investigator, a dose adjustment is required.

Table 2 Potential Risks of Liraglutide, Alone and Co-Administered with GSK2890457 and Their Mitigation*

Potential Risk	Liraglutide	Liraglutide + GSK2890457	Mitigation
Nausea / Vomiting	Liraglutide is associated with a 15 to 40% incidence of nausea, depending on the dose. Nausea is linked to the rate of change of plasma concentration, as well as to the absolute plasma concentration achieved. For most patients, nausea is transient, and tolerability improves with repeated dosing. Gradual dose titration has been shown to minimize adverse effects.	Nausea and vomiting are not typical of the side effects of the components of GSK2890457<br><br>As a result, it is not anticipated that GSK2890457 will exacerbate GLP-1 receptor mediated nausea and vomiting once subjects have been stabilized on liraglutide.	-- The dose of liraglutide will be increased gradually as indicated in the Victoza Prescribing Information.<br><br>-- The dose of GSK2890457 will be increased over 7 days.<br><br>--Nausea and vomiting will be closely monitored and treated as appropriate.<br><br>-- Subjects with gastroparesis requiring treatment will be excluded.

Potential Risk	Liraglutide	Liraglutide + GSK2890457	Mitigation
Hypoglycemia	In patients with T2D, hypoglycemia has been associated with administration of liraglutide when given in conjunction with other agents, such as insulin and insulin secretagogues such as sulphonylureas that have an intrinsic hypoglycemic risk.<br><br>Experience with liraglutide indicates that the risk of hypoglycemia is very low when it is used as monotherapy in T2D patients. [VICTOZA, 2012 Prescribing Information]	As GSK2890457 does not produce hypoglycemia in animal models and glucose improvement is observed in the absence of elevation of circulating insulin levels, it is unlikely that GSK2890457 will augment the very low risk of hypoglycemia seen with liraglutide.<br><br>Nevertheless, careful monitoring for hypoglycemia is advisable because it may occur when caloric intake is reduced,	-- Exclude subjects with hypoglycemic unawareness<br><br>--Fasting and pre-dinner CBG will be monitored daily using a telemonitored device while subjects are at home.<br><br>--Subjects will be closely monitored for signs and symptoms of hypoglycemia and will be treated appropriately.<br><br>-Subjects will be given instructions on how to handle hypoglycaemia.<br><br>--Ad hoc capillary glucose measurements will be used to check blood glucose if a subject has symptoms of hypoglycemia or is otherwise concerned about their blood glucose level.<br><br>--T2D subjects clearly experiencing episodes of symptomatic hypoglycemia or episodes of significant confirmed hypoglycemia regardless of symptoms should have the dose of liraglutide and/or GSK2890457 reduced as indicated in Section 4.6.1.<br><br>-- If symptoms of hypoglycemia are reported, the Investigator will discuss with the GSK Medical Monitor if it is appropriate to modify doses (e.g., reduce the liraglutide dose to 1.2mg). A subject should be withdrawn if episodes of clinically significant hypoglycemia persist even when the dose of liraglutide has been reduced to 1.2mg.<br><br>-- Subjects may continue if the episodes of hypoglycemia are not considered clinically significant (e.g., because they are a physiological response to fasting).

**Potential Risk**	**Liraglutide**	**Liraglutide + GSK2890457**	**Mitigation**
Pancreatitis	Liraglutide has been associated with acute pancreatitis, including fatal and non-fatal hemorrhagic or necrotizing pancreatitis.	As the components of GSK2890457 are food ingredients and there were no pancreatic lesions in the 6 week rat toxicity study, it is not anticipated that co-dosing for 6 weeks will materially increase the risk to subjects.	--Subjects with a history of pancreatitis, with ongoing symptomatic biliary disease, or with lipase/amylase ≥2xULN at Screening are excluded from the study (Section 5.1.4).<br><br>-- Lipase\amylase monitoring during the Stabilization and Treatment periods, with algorithm for management of abnormalities (Section 4.6.1.3).<br><br>-- Subjects will be closely monitored and will be treated promptly as appropriate.<br><br>--In Part B, in the event of treatment emergent confirmed pancreatitis, dosing with GSK2890457 and liraglutide should be stopped immediately. The subject will not be rechallenged.
Medullary and Papillary Thyroid Carcinoma	Liraglutide may be associated with an increased risk of developing medullary thyroid carcinoma. This association is based on chronic exposure studies in rodents and the relevance to humans is unclear.<br><br>In clinical trials with liraglutide, there were 7 reported cases of papillary thyroid cancer in patients treated with liraglutide and 1 case in a comparator-treated patient (1.5 versus 0.5 cases per 1000 patient-years).	As the components of GSK2890457 are food ingredients and there were no thyroid lesions in the 6 week rat toxicity study, it is not anticipated that co-dosing for 6 weeks will materially increase the risk to subjects.	--Subjects with a personal or family history of medullary or papillary carcinoma of the thyroid or multiple endocrine neoplasia type 2, or with plasma calcitonin levels at screening >50pg/mL are excluded from the study.<br><br>- A careful thyroid physical examination will be conducted at screening and before the start of the 6-week Treatment Period. A subject should be excluded if an abnormal thyroid mass is detected.

Potential Risk	Liraglutide	Liraglutide + GSK2890457	Mitigation
Renal Impairment	Renal impairment has been reported in association with nausea, vomiting, diarrhea, and sometimes hemodialysis. The mechanism is likely to be dehydration resulting in renal hypoperfusion.<br><br>Liraglutide is not directly nephrotoxic in animal studies or clinical trials.	As the components of GSK2890457 are food ingredients and there were no renal lesions in the 6 week rat toxicity study, it is not anticipated that co-dosing for 6 weeks will materially increase the risk to subjects.	-- Only subjects with an estimated GFR ≥ 60mL/min and urine albumin excretion of <30mg albumin/g creatinine will be eligible for the study.<br><br>-- Carefully monitor fluid status of subjects via routine laboratory markers of hemoconcentration. Subjects will be encouraged to drink adequate fluids.<br><br>- Subjects will be advised to report episodes of nausea/vomiting that are significantly compromising food/fluid intake, and will be advised on ways to maintain fluid intake. Dose modification may be required to reduce the severity of nausea/vomiting.<br><br>- Stopping criteria based on estimated GFR are included in Section 4.6.1.
Injection site reactions / acute allergic reactions / immunogenicity	Injection site reactions and acute allergic reactions are a potential risk with all protein therapeutics. As indicated in the Prescribing Information for Victoza, these reactions have not been a common cause for discontinuing treatment with liraglutide.<br><br>As is the case for all protein therapeutics, antibodies may develop against liraglutide (8.6% of patients tested in long-term clinical trials had anti-liraglutide antibodies, and 5 to 7% had antibodies that cross-reacted to native GLP-1). Antibody formation was neutralizing in 1 to 2% of patients.<br><br>Immunogenicity-related events, including urticaria, are more common in liraglutide treated patients (0.8%) than in controls (0.4%).	As the components of GSK2890457 are food ingredients, it is not anticipated that co-dosing for 6 weeks will materially increase the risk to subjects.	--Subjects will be closely monitored for adverse effects, and will be treated promptly as appropriate.<br><br>-- In Part B, dosing should be stopped immediately for subjects with significant acute hypersensitivity reactions (for example, urticaria, anaphylactic reactions or angioedema) or other potentially immune-mediated reactions that are not clearly attributable to other causes (e.g. shrimp consumption with known shellfish allergy).<br><br>-- Subjects will be treated promptly as appropriate if symptoms and/or signs of hypersensitivity occur.

Potential Risk	Liraglutide	Liraglutide + GSK2890457	Mitigation
Suicidal ideation	Liraglutide may have CNS actions. There has been concern that some CNS-active drugs may be associated with an increased risk of suicidal thinking or behaviour when given to some patients with certain conditions. Although liraglutide or other similar drugs in the class have not been shown to be associated with an increased risk of suicidal thinking or behaviour when given to T2D patients, GSK considers it important to monitor for such events in this clinical study.	As the components of GSK2890457 are food ingredients, it is not anticipated that co-dosing for 6 weeks will increase the risk to subjects.	-- Subjects are excluded at Screening if they have a past history of psychiatric illness including depression, suicidal thoughts, schizophrenia, bipolar disorder, or generalized anxiety disorder.<br><br>-- Monitor suicidal ideation or behavior using the Columbia Suicide Severity Rating Scale (C-SSRS) during the Stabilization and co-dosing Treatment periods.<br><br>-- Consideration should be given to discontinuing liraglutide in subjects who experience signs of suicidal ideation or behaviour.
Pharmacokinetics	Liraglutide is injected subcutaneously and is eliminated by the same mechanisms that remove endogenous proteins without a specific organ as the major route of elimination.	As the components of GSK2890457 are food ingredients, it is not anticipated that it will significantly affect the PK of liraglutide during the 6 weeks co-dosing period.<br><br>Liraglutide can affect gastric emptying and may alter the absorption of concomitantly administered oral medications. This is not expected to result in a significant drug interaction because several components of GSK2890457 are poorly absorbed and it is likely that most of the pharmacological effects of GSK2890457 come from intraluminal, gut-based mechanisms.	-- Steady-state PK of liraglutide will be measured.<br><br>-- If validated, the systemic exposure of one of the components of GSK2890457 will be monitored.
*See the VICTOZA, 2012 Prescribing Information for further information on potential risks.			

1.3.3. Potential Risks of Co-administration of Metformin and GSK2890457 and Their Mitigation

In Part A, subjects will receive a single 500mg IR dose of metformin at baseline and after 6 weeks of dosing with GSK2890457 to investigate the potential for a pharmacokinetic interaction between these agents.

In Part C, subjects on prior metformin monotherapy will have a 4 week Stabilization period on their usual metformin therapy before entering the 6 week Treatment Period. Subjects will be discharged at the Follow-up visit on their usual dose of metformin.

Table 3 Potential Risks of Metformin and Co-administration with GSK2890457

Potential Risk	Metformin	Metformin + GSK2890457	Mitigation
Gastrointestinal side-effects	Diarrhea, nausea, vomiting, flatulence, indigestion and abdominal discomfort were more common in metformin- than placebo-treated patients in clinical trials. [GLUCOPHAGE, 2009 Prescribing Information] Diarrhea led to discontinuation of metformin in 6% of patients treated with the drug.	As 2 components of GSK2890457 are known to cause dose-dependent gastrointestinal symptoms, there is the potential for an increase in gastrointestinal side effects during the 6-week co-dosing Treatment Period.	The dose of GSK2890457 will be titrated gradually over 7 days, and subjects will have the option of decreasing the dose (within protocol-defined limits) if tolerability is unacceptable.

Potential Risk	Metformin	Metformin + GSK2890457	Mitigation
Hypoglycemia	While hypoglycemia has been reported in ≥1.0% to ≤5.0% of metformin patients (and more commonly than placebo) in clinical studies, the risk is very low when it is used as monotherapy in T2D patients.<br><br>This is because metformin improves fasting and prandial glucose levels, without increasing circulating insulin levels.	As GSK2890457 does not produce hypoglycemia in animal models and glucose improvement is observed in the absence of elevation of circulating insulin levels, it is unlikely that GSK2890457 will augment the very low risk of hypoglycemia seen with metformin.<br><br>Nevertheless, careful monitoring for hypoglycemia is advisable because it may occur when caloric intake is reduced.	--Exclude subjects with hypoglycemic unawareness<br><br>--Fasting and pre-dinner CBG will be monitored daily using a telemonitored device while subjects are at home.<br><br>--Subjects will be closely monitored for signs and symptoms of hypoglycemia and will be treated appropriately.<br><br>-Subjects will be given instructions on how to handle hypoglycaemia.<br><br>--Ad hoc CBG measurements to check status if a subject has symptoms of hypoglycemia or is otherwise concerned about their blood glucose level.<br><br>--Subjects clearly experiencing episodes of symptomatic hypoglycemia or episodes of significant confirmed hypoglycemia regardless of symptoms should have the dose of GSK2890457 and/or metformin reduced as indicated in Section 4.6.1.<br><br>If symptoms of hypoglycemia are reported, the Investigator can discuss with the GSK Medical Monitor if it is appropriate to modify the doses of study medication. A subject should be withdrawn if episodes of clinically-significant hypoglycemia persist even after dose adjustment.<br><br>Subjects may continue if the episodes of hypoglycemia are not considered clinically significant (e.g., because they are a physiological response to fasting).

Potential Risk	Metformin	Metformin + GSK2890457	Mitigation
Lactic acidosis	Lactic acidosis is a rare, but serious, metabolic complication that can occur due to metformin accumulation during treatment. It is fatal in approximately 50% of cases. Lactic acidosis may also occur in T2D and whenever there is significant tissue hypoperfusion and hypoxemia.<br><br>In more than 20,000 patient-years exposure to metformin in clinical trials, there were no reports of lactic acidosis. Reported cases have occurred primarily in T2D patients with significant renal insufficiency, often in the setting of multiple concomitant medical/surgical problems and multiple concomitant medications. Patients with congestive heart failure are also at increased risk of lactic acidosis. The risk of lactic acidosis increases with the degree of renal dysfunction and the patient's age.	As the components of GSK2890457 are food ingredients and no food-related events of lactic acidosis have been reported for T2D patients on metformin, it is not anticipated that co-dosing for 6 weeks will materially increase the risk to subjects.	--Subjects with a history of renal or cardiac disease at Screening are excluded from the study (Section 5.1.4).<br><br>-- Only subjects without evidence of hepatic impairment are eligible because impaired hepatic function may limit the clearance of lactate.<br><br>-- Patients will be cautioned against excessive alcohol intake (either acute or chronic), as this may potentiate the effect of metformin on lactate metabolism<br><br>--Subjects will be closely monitored, and will be treated promptly as appropriate.<br><br>--In Part C, in the event of treatment emergent symptoms and/or signs of lactic acidosis (malaise, myalgias, respiratory distress, increasing somnolence, and nonspecific abdominal distress, associated hypothermia, hypotension, and resistant bradyarrhythmias with more marked acidosis), dosing with GSK2890457 and/or metformin should be reviewed and stopped, if appropriate.
Vitamin B12	In controlled clinical trials, a decrease to subnormal levels of previously normal serum vitamin B12 levels, without clinical manifestations, was observed in approximately 7% of patients. However, such a decrease, possibly due to interference with B12 absorption from the B12-intrinsic factor complex, is very rarely associated with anemia, and appears to be rapidly reversible with discontinuation of metformin or vitamin B12 supplementation.	As some components of GSK2890457 may alter the absorption of certain minerals, and potentially some vitamins, a risk of exacerbation of vitamin B12 malabsorption cannot be excluded.	--Subjects with a history of untreated pernicious anemia will be excluded.<br><br>-- Exclude subjects at Screening who have lab parameters suggestive of subclinical megaloblastic anemia (e.g., increased MCV with low RBC count and/or Hb level).<br><br>-- Hematologic parameters will be monitored carefully. Any apparent abnormalities should be appropriately investigated and managed.

Potential Risk	Metformin	Metformin + GSK2890457	Mitigation
Pharmacokinetics	Metformin is absorbed slowly from the upper gastrointestinal tract, and has a bioavailability of ~40-60%. About 20-30% of a dose appears in the feces. It accumulates in the gastrointestinal tract because of its highly cationic structure. After absorption, metformin is eliminated by glomerular filtration and active secretion into the nephron.<br><br>The risk of metformin accumulation and lactic acidosis increases with the degree of impairment of renal function.	As the absorption of metformin is reduced by food, GSK2890457 may alter its bioavailability, but it should not have any effect on metformin elimination by the kidney or uptake into tissues via organic cation transporters.<br><br>Conversely, it is unlikely that metformin will have an impact on the bioavailability of the components of GSK2890457 because they are poorly absorbed on their own.	- PK of metformin measured in Part A<br><br>- Steady-state PK of metformin will be measured in Part C.<br><br>- If validated, systemic exposure of one of the components of GSK2890457 will be monitored.

1.3.4. Potential Risks of Deuterated Creatine and Their Mitigation

There are no reported safety issues with creatine at the dose specified for this study, (single doses of 30mg). One of the main natural sources of dietary creatine is red meat, especially lean meat. It is estimated that every one pound of raw meat contains ~2g of creatine. Another source of natural creatine is fish like tuna and salmon. A small minimal amount of creatine can be found in milk and cranberries. Creatine is used widely as a nutritional supplement for a variety of reasons including individuals with low body weight or muscle mass. A small number of clinical studies have been performed in special populations with doses ranging from 5g qd, to 15g bid with no reported adverse effects related to the creatine except diarrhea at the high dose (healthy swimmers – 5g qid for one week, [Mendes, 2004]; amyotrophic lateral sclerosis -5 to 15g bid for one week, [Atassi, 2010] and T2DM-5g qd for 12 weeks, [Gualano 2010].

There is no apparent risk to subjects from the deuterated creatine. Deuterium is a stable, naturally occurring isotope that is non-radioactive.

Based on this established safety record, no compound-specific withdrawal criteria are included for deuterated creatine.

2. OBJECTIVE(S)

2.1. Primary Objectives

- To investigate the safety and tolerability of GSK2890457 when administered for 6 weeks to healthy subjects (Part A), and when co-administered to subjects with T2D for 6 weeks with liraglutide (Part B) or metformin (Part C).
- To evaluate the pharmacodynamic effects of GSK2890457 in subjects with T2D when co-administered for 6 weeks with liraglutide (Part B) or metformin (Part C).

2.2. Secondary Objective

- To evaluate the effects of GSK2890457 on pharmacokinetic parameters of liraglutide (Part B) and metformin (Parts A and C) when the agents are co-administered, as data permit.

2.3. Exploratory Objectives

- To explore the exposure of one component of GSK2890457 in subjects with T2D, as data permit (Parts B and C).
- To explore biomarkers of obesity, T2D and/or related metabolic diseases and/or inflammatory diseases or conditions, and biomarkers of GSK2890457 pharmacodynamics and/or safety when co-administered to subjects with T2D for six weeks with liraglutide (Part B) or metformin (Part C).
- To attempt preliminary identification of the major GSK2890457-related components and metabolites in plasma and urine in pooled subject samples (Parts B and C).
- To evaluate a novel method of measuring total skeletal muscle mass using deuterated creatine if it is available for dosing (Parts B and C).

3. ENDPOINT(S)

3.1. Primary Endpoints

- Safety and tolerability parameters including but not limited to adverse events, clinical laboratory, ECG and vital signs assessments, including hypoglycaemia events, and results from the patient-completed Gastrointestinal (GI) Symptoms Questionnaire (GSRS) (Parts A through C). [Appendix 3: GI Symptom Rating Scale (GSRS) Questionnaire]
- Change and % change in body weight from baseline to end of treatment, and differences in weight-loss and weight-gain patterns from baseline through to end of treatment, as compared to patterns during the last four weeks of the baseline Stabilization Period, in subjects with T2D (Parts B and C).
- Area under the curve weighted mean AUCs (0 to 24 hours) for glucose on Days -1 and 42, in subjects with T2D (Parts B and C).
- Fasting blood glucose patterns from Day-1 through Day 42, as compared to patterns during the baseline Stabilization Period in subjects with T2D (Parts B and C).
- Fasting plasma glucose and insulin levels, HOMA-IR and Matsuda index measures of insulin sensitivity, and weighted mean AUCs (0 to 4h) for glucose and insulin after a standardized breakfast meal on Days -1 and 42 in subjects with T2D (Parts B and C).
- Change in HbA1c from Day -1 to Day 42 in subjects with T2D (Parts B and C).

3.2. Secondary Endpoints

- Pharmacokinetic parameters of liraglutide (Part B) and metformin (Parts A and C): AUCss, Cmax, tmax, on Day 42 as compared to Day -1(Day 1 in Part A).

3.3. Exploratory Endpoints

- Pharmacokinetic parameters of the representative plasma marker(s) for one component of GSK2890457: AUCs, Cmax, tmax, area under the plasma drug concentration versus time curve on Day 42 in subjects with T2D (Parts B and C).
- Change from Day -1 to Day 42 in waist circumference (Parts B and C).
- Change from Day -1 to Day 42 in the patient-completed Hunger Questionnaire [Appendix 2: Hunger Questionnaire] (Parts B and C).
- Fasting plasma leptin, CRP and lipid [HDLc, LDLc, triglycerides] on Day-1 compared to Day 42 (Parts B and C)].
- DEXA measurement of total lean mass and appendicular on Day 41 as compared to Day -2(Parts B and C).
- Skeletal muscle mass estimated from deuterated creatinine enrichment in urine on Day 43 as compared to Day 1(Parts B and C).
- Liver enzymes and non-alcoholic fatty liver disease (NAFLD) Fibrosis score on Day 42 as compared to Day -1(Parts B and C).
- Fecal microbiome at baseline (Day-2 to Day -1) compared to end-of-treatment (Day 41-42) (Parts B and C).
- Exploratory analyses relating to biomarkers of obesity, T2D and/or related metabolic diseases and/or inflammatory diseases or conditions, and biomarkers of GSK2890457 pharmacodynamics or safety may be performed using (i) small molecular weight metabolites, (ii) blood polypeptide analytes, and (iii) novel biomarkers derived from peptidomic, lipidomic and metabolomic analysis of blood and/or urine, as data permit. Exploratory analyses may be conducted with biomarkers of adiposity, inflammation, insulin sensitivity, beta cell function, gut peptide secretion and other exploratory biomarkers, as data permit.
- Exploratory analysis relating to circulating metabolites of GSK2890457 may be performed using analytical techniques such as Liquid Chromatography Mass Spectrometry and NMR Spectroscopy.

4. INVESTIGATIONAL PLAN

4.1. Part A

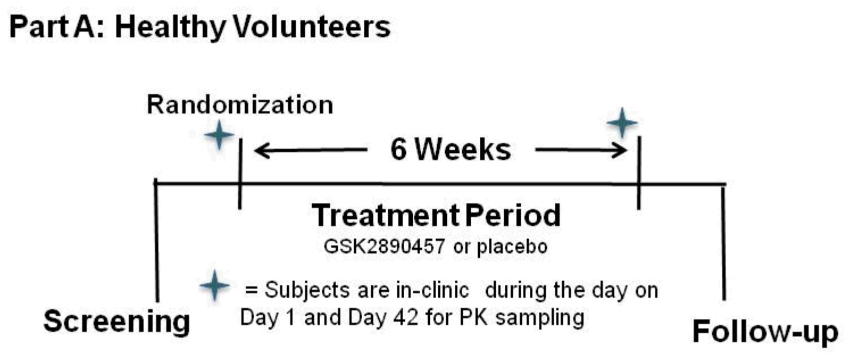

- Healthy subjects who fulfil the Screening requirements will be randomized to receive either GSK2890457 or placebo for 6 weeks, to evaluate safety and tolerability. Note: subjects who have fulfilled inclusion/exclusion requirements, but who do not start the Treatment period within 28 days of Screening may be re-screened using the procedures outline in Section 7.3.6.
- The subjects will be instructed how to mix a 5g dose of the study medication and will take it under the supervision of an unblinded site staff member at dinner on Day 1, the time of randomization. Study medication for home administration will be dispensed at that time. As GSK2890457 had limited efficacy on its own in the animal models of T2D, the risk of hypoglycemia is considered to be low and the healthy subjects will not be required to measure daily CBG levels.
- On Days 1 and 42, subjects will come in to the unit fasting (nothing after midnight the night before; minimum 8-hour fast) and receive a single 500mg IR tablet of metformin just before eating breakfast, and will stay in the clinical unit for 10h to allow collection of blood samples for analysis of metformin concentrations. On Day 1, GSK2890457 will be dosed immediately prior to dinner. On Day 42, the subjects will also take GSK2890457/placebo immediately before breakfast (the order will be: take metformin followed by GSK2890457 or placebo and then breakfast) and then they will take the second dose before dinner. Identical meals must be served on Days 1 and 42.
- A follow-up visit will occur approximately 14 days after completion of the 6-week dosing period.

4.2. Part B

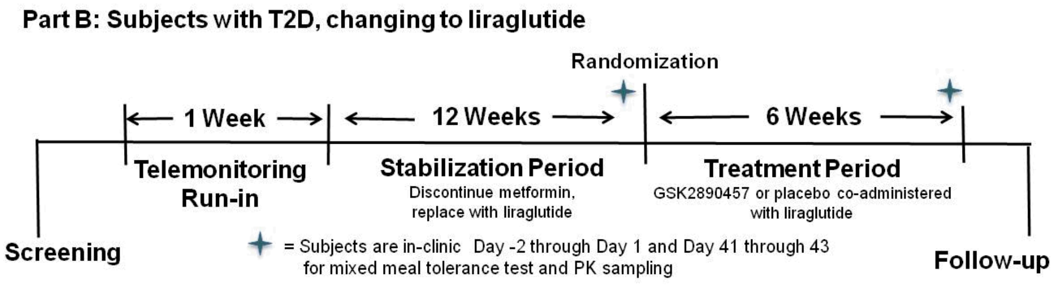

4.2.1. Run-in Period (1 week)

- T2D subjects who fulfil the Screening requirements will begin a one-week run-in “procedures familiarization” period during which they will:
  - Remain on their usual dose of metformin during the Run-in only.
  - Weigh themselves once daily at home fasted, first thing in the morning, using a telemonitoring scale (provided).

   - Check fasting (minimum 8-hour fast) and pre-dinner CBG daily at home, and transmit values daily if possible, but at least every other day via the telemonitoring unit (glucometer, strips and telemonitoring unit will be provided).

- Subjects who are unable or unwilling to demonstrate 80% compliance with the telemonitoring requirements during the run-in “procedures familiarization” period will be withdrawn from the study and will not proceed to the Stabilization Period.

Note: subjects who have fulfilled inclusion/exclusion requirements, but who do not start the Stabilisation period within 28 days of Screening may be re-screened using the procedures outlined in Section 7.3.6.

4.2.2. Stabilization Period (12 weeks)

- During the Stabilization Period, subjects will:
  - Remain on their usual diet and exercise program, as advised by their primary care physician.
  - Stop their usual metformin therapy and commence titration and daily dosing with subcutaneous injections of liraglutide (see Section 4.2.2 for liraglutide titration regimen).
  - Weigh themselves once daily at home fasted, first thing in the morning, using a telemonitoring scale (provided). When weighing at home, subjects should be nude. When subjects are in-house, they will be weighed on a designated scale for this study; they do not need to bring their home telemonitored scale in to the unit for routine visits, only at the end of the study. When weighed in the unit, subjects should be weighed without shoes in light clothing. The same scale should be used throughout the study for all subjects at every designated visit.
  - Check fasting (minimum 8-hour fast) and pre-dinner CBG daily at home, and transmit values daily if possible, but at least every other day via the telemonitoring unit (glucometer, strips and telemonitoring unit will be provided). Subjects may measure their CBG at other times if they are concerned about potential hypoglycaemia or their blood glucose levels. Note: Subjects do not need to bring their telemonitoring wireless hub into the unit for routine visits, only at the end of the study.
  - Subjects not using the telemonitoring glucose and weight equipment appropriately will be counselled on correct use, and technical issues will be addressed, if necessary. If poor compliance continues and does not improve with counselling, this may result in withdrawal from the study, after consultation with the GSK Medical Monitor.
- Subjects will receive guidance on Mindful Eating (Appendix 6: Mindful Eating Guide).
- Suicidality monitoring will be performed at Week 1 and 7 using a subject-reported C-SSRS questionnaire.
- Subjects will return to the clinic for visits at Weeks 1, 3, 7 and 10 of the Stabilization period.

- Urine drug screens will be performed at each clinic visit.

4.2.3. Treatment Period (6 weeks)

- During the Treatment Period, subjects will:
  - Remain on their stabilized dose of liraglutide by subcutaneous injection.
  - Remain on their usual diet and exercise program when at home, as advised by their primary care physician.
  - Weigh themselves once daily at home fasted, first thing in the morning, using a telemonitoring scale (provided). When weighing at home, subjects should be nude. When subjects are in-house, they will be weighed on a designated scale for this study; they do not need to bring their home telemonitored scale in to the unit for routine visits, only at the end of the study. When weighed in the unit, subjects should be weighed without shoes in light clothing. The same scale should be used throughout the study for all subjects at all designated study visits.
  - Check fasting (minimum 8-hour fast) and pre-dinner CBG daily at home, and transmit values daily if possible, but at least every other day via the telemonitoring unit (glucometer, strips and telemonitoring unit will be provided). Subjects may measure their CBG at other times if they are concerned about potential hypoglycaemia or their blood glucose levels. Note: Subjects do not need to bring their telemonitoring wireless hub into the unit for routine visits, only at the end of the study.
  - Subjects not using the telemonitoring glucose and weight equipment appropriately will be counselled on correct use, and technical issues will be addressed, if necessary. If poor compliance continues and does not improve with counselling, this may result in withdrawal from the study, after consultation with the GSK Medical Monitor.
- Subjects will receive guidance on Mindful Eating.
- Suicidality monitoring will be performed at Screening, Day -2, Day 41, and at Follow-up using a subject-reported C-SSRS questionnaire.
- Urine drug screens will be performed at each clinic visit.
- On Day -2, subjects will enter the clinic and will be provided with a standardized dinner. DEXA evaluation of fat/lean body mass, baseline Food Frequency Questionnaire and GSRS will be completed. A dose of 30mg deuterated (non-radioactive) creatine will be administered to subjects in the afternoon about 2 hours after lunch if it is available.
- On Day -1, a standardized breakfast, lunch, and dinner will be provided, and blood samples will be taken throughout the day to measure glucose, insulin, and potentially other biomarkers. Blood for pharmacokinetic measurement will be obtained. An NAFLD score will be determined. The Hunger Questionnaire will be administered at several timepoints. Baseline safety measurements including hematology, clinical chemistry, urinalysis, vital signs, and triplicate ECG will be taken. A fecal sample will be obtained at some point during this in-house stay prior to starting the study treatment.

- On Day 1, subjects will be instructed how to mix the study medication and then they will take it under the supervision of an unblinded site staff member prior to breakfast. Study medication will be dispensed at the time of randomization. Subjects will be discharged following final blood sampling and breakfast. A morning urine sample will also be taken before discharge to measure deuterated-creatine method analytes if the deuterated creatine dose has been administered.
- All standardized meals should *not* contain berry fruits.
- Subjects will return for clinic visits on Days 7, 14, and 28 for safety evaluations, potential modification of dose (Days 7 and 14), and dispensing of study medications. Drug accountability will be performed to ensure subject compliance with dosing. **Note:** If a subject cannot attend the clinic in the morning of these scheduled visits for fasting predose assessments, the subject should take the study treatment at home before eating breakfast and then come to the clinic in the afternoon for study assessments. It will be noted in the CRF that measurements were not completed in a fasted state.
- On Day 41, subjects will enter the clinic, and the Day -2 procedures will be repeated, with the exception that the Food Frequency Questionnaire will not be completed. A urine sample will also be taken prior to the second dose of deuterated-creatine to measure baseline deuterated-creatine method analytes if the deuterated creatine has been dosed. The second dose of deuterated creatine will be administered in the afternoon similar to Day -2 if available. GSK2890457 or placebo will be administered just prior to dinner. Dinner should be identical to that given on Day -2.
- On Day 42, the Day -1 procedures will be repeated, with the exception of Vitamin D measurement, and that only a single ECG will be performed. GSK2890457 or placebo will be taken just prior to breakfast and just prior to dinner, as noted in Section 4.7.6. All meals on Day 42 should be identical to those on Day -1.
- On Day 43, subjects will be discharged following final blood sampling and breakfast. A morning urine sample will also be taken before discharge to measure deuterated creatine method analytes similar to collection and time of day as on Day 1 if the deuterated creatine dose has been administered. No liraglutide will be administered and subjects will be instructed to re-start their usual dose of metformin (the dose may be modified, if appropriate).

A follow-up visit will occur approximately 14 days after discharge from the unit on Day 43.

See the Time and Events Tables (Section 4.7) for additional detail about study assessments.

4.3. Part C

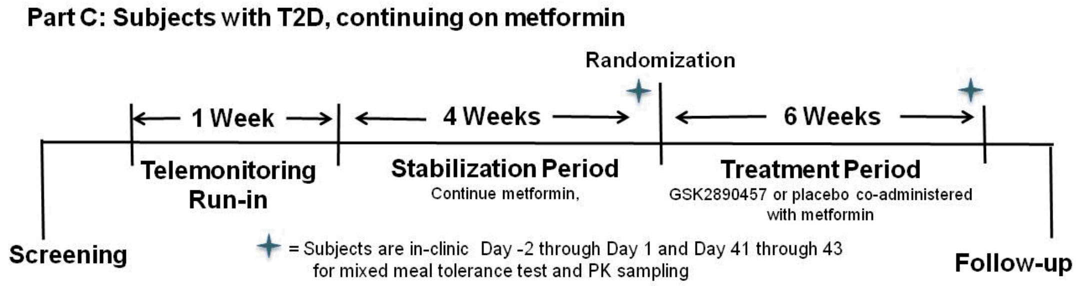

4.3.1. Run-in Period (1 week)

- T2D subjects who fulfil the Screening requirements will begin a one-week run-in "procedures familiarization" period during which they will:
  - Remain on their usual dose of metformin.
  - Weigh themselves once daily at home fasted, first thing in the morning, using a telemonitoring scale (provided).
  - Check fasting (minimum 8-hour fast) and pre-dinner CBG daily at home, and transmit values daily if possible, but at least every other day via the telemonitoring unit (glucometer, strips and telemonitoring unit will be provided).
- Subjects who are unable or unwilling to demonstrate 80% compliance with the telemonitoring requirements during the run-in "procedures familiarization" period will be withdrawn from the study and will not proceed to the Stabilization Period.

Note: subjects who have fulfilled inclusion/exclusion requirements, but who do not start the Stabilisation period within 28 days of Screening may be re-screened using the procedures outline in Section 7.3.6.

4.3.2. Stabilization Period (4 weeks)

- During the Stabilization Period, subjects will:
  - Remain on their current dose of metformin.
  - Remain on their usual diet and exercise program, as advised by their primary care physician.
  - Weigh themselves once daily at home fasted, first thing in the morning, using a telemonitoring scale (provided). When weighing at home, subjects should be nude. When subjects are in-house, they will be weighed on a designated scale for this study; they do not need to bring their home telemonitored scale in to the unit for routine visits, only at the end of the study. When weighed in the unit, subjects should be weighed without shoes in light clothing. The same scale should be used throughout the study for all subjects at each designated study visit.

  - Check fasting (minimum 8-hour fast) and pre-dinner CBG daily at home, and transmit values daily if possible, but at least every other day via the telemonitoring unit (glucometer, strips and telemonitoring unit will be provided). Subjects may measure their CBG at other times if they are concerned about potential hypoglycaemia or their blood glucose levels. Note: Subjects do not need to bring their telemonitoring wireless hub into the unit for routine visits, only at the end of the study.
  - Subjects not using the telemonitoring glucose and weight equipment appropriately will be counselled on correct use, and technical issues will be addressed, if necessary. If poor compliance continues and does not improve with counselling, this may result in withdrawal from the study, after consultation with the GSK Medical Monitor.
- Subjects will receive guidance on Mindful Eating.
- Subjects will return to the clinic for visits at Week 1 and a phone contact will be made at Week 3.
- Urine drug screens will be performed at each clinic visit.

4.3.3. Treatment Period (6 weeks)

- During the Treatment Period, subjects will:
  - Remain on their usual dose of metformin.
  - Remain on their usual diet and exercise program when at home, as advised by their primary care physician.
  - Weigh themselves once daily at home fasted, first thing in the morning, using a telemonitoring scale (provided). When weighing at home, subjects should be nude. When subjects are in-house, they will be weighed on a designated scale for this study; they do not need to bring their home telemonitored scale in to the unit for routine visits, only at the end of the study. When weighed in the unit, subjects should be weighed without shoes in light clothing. The same scale should be used throughout the study for all subjects at each designated study visit.
  - Check fasting (minimum 8-hour fast) and pre-dinner CBG daily at home, and transmit values daily if possible, but at least every other day via the telemonitoring unit (glucometer, strips and telemonitoring unit will be provided). Subjects may measure their capillary blood glucose at other times if they are concerned about potential hypoglycaemia or their blood glucose levels. Note: Subjects do not need to bring their telemonitoring wireless hub into the unit for routine visits, only at the end of the study.
  - Subjects not using the telemonitoring glucose and weight equipment appropriately will be counselled on correct use, and technical issues will be addressed, if necessary. If poor compliance continues and does not improve with counselling, this may result in withdrawal from the study, after consultation with the GSK Medical Monitor.
- Subjects will receive guidance on Mindful Eating.
- Urine drug screens will be performed at each clinic visit.

- On Day -2, subjects will enter the clinic and will be provided with a standardized dinner. DEXA evaluation of fat/lean body mass, baseline Food Frequency Questionnaire and GSRS will be completed. A dose of 30mg deuterated (non-radioactive) creatine will be administered to subjects in the afternoon about 2 hours after lunch if it is available.
- On Day -1, a standardized breakfast, lunch, and dinner will be provided, and blood samples will be taken throughout the day to measure glucose, insulin, and potentially other biomarkers. Blood for pharmacokinetic measurement will be obtained. The Hunger Questionnaire will be administered at several timepoints. Baseline safety measurements including hematology, clinical chemistry, urinalysis, vital signs, and triplicate ECG will be taken. A fecal sample will be obtained at some point during this in-house stay prior to starting the study treatment.
- On Day 1, subjects will be instructed how to mix the study medication and then they will take it under the supervision of an unblinded site staff member prior to breakfast. Study medication will be dispensed at the time of randomization. Subjects will be discharged following final blood sampling and breakfast. A morning urine sample will also be taken before discharge to measure deuterated creatine method analytes (e.g., creatine, creatinine and deuterated creatinine) if the dose of deuterated creatine has been administered.
- The order at dosing should be: take metformin one hour prior to GSK2890457 or placebo, and then the breakfast meal. If the subject is taking metformin BID, the evening dose should be taken one hour prior to dosing of GSK2890457/placebo, and then dinner. Metformin should be taken with a small portion of the meal, with the remainder of the meal consumed after GSK2890457.
- All standardized meals should *not* contain berry fruits.
- Subjects will return for clinic visits on Days 7, 14, and 28 for safety evaluations, potential modification of dose (Days 7 and 14), and dispensing of study medications. Drug accountability will be performed to ensure subject compliance with dosing. **Note:** If a subject cannot attend the clinic in the morning of these scheduled visits for fasting predose assessments, the subject should take the study treatment at home before eating breakfast and then come to the clinic in the afternoon for study assessments. It will be noted in the CRF that measurements were not completed in a fasted state
- On Day 41, subjects will enter the clinic, and the Day -2 procedures will be repeated, with the exception that the Food Frequency Questionnaire will not be completed. If the deuterated creatine has been dosed, a urine sample will also be taken prior to the second dose of deuterated-creatine to measure baseline deuterated-creatine method analytes. The second dose of deuterated creatine will be administered in the afternoon similar to Day -2 if it is available.
- On Day 42, the Day -1 procedures will be repeated, with the exception that only a single ECG will be performed. GSK2890457 or placebo will be taken just prior to breakfast and just prior to dinner, as noted in Section 4.7.6.
- On Day 43, subjects will be discharged following final blood sampling and breakfast. If the deuterated creatine dose has been administered, a morning urine sample will

also be taken before discharge to measure deuterated creatine method analytes similar to collection and time of day as on Day 1.

A follow-up visit will occur approximately 14 days, after discharge from the unit on Day 43.

See the Time and Events Tables (Section 4.7) for additional detail about study assessments.

Protocol waivers or exemptions are not allowed with the exception of circumstances relating to immediate safety concerns. Therefore, adherence to the study design requirements, including those specified in the Time and Events Tables, is essential and required for study conduct (Section 4.7).

4.4. Dosage and Administration

4.4.1. GSK2890457 Dosage and Administration (Parts A-C)

- The planned dosing regimen for Parts A through C is shown in the diagram below (in Part A the Day 1 dose is 5g). The subjects will titrate up from 15g to 40g over a 7 day period, if tolerated. Note that the doses/dosing regimen used in Part B and C may be altered if significant safety or tolerability issues are identified in Part A. Doses of study medication will not be greater than 40g.

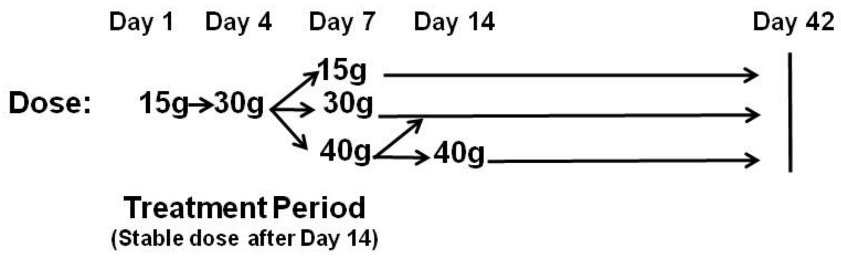

- The 'unit' dose of study medication will be provided as a 5g kit containing three sachets of powder and a bottle containing 3 capsules. For all doses of GSK2890457 the three powders will be mixed by the subject into 12oz of flavored (MiO pomegranate/berry flavoring) water in a special shaker bottle (provided by site) and consumed just prior to breakfast and dinner. The capsules can be taken with the 'powder' drink or separately with approximately 120-240mL of water immediately prior to breakfast and dinner. A subject handout describing dose administration can be found in Appendix 5: Directions for Taking Study Medicine.
- All subjects will begin taking a 15g daily dose of either GSK2890457 or placebo on Day 1, consuming 5g just prior to breakfast and 10g just prior to dinner (except for Part A as noted above). If a subject does not consume both breakfast and dinner on any given day, then the study medication can be taken without food, but should be taken at approximately breakfast time and approximately dinner time.

- On Day 4, all subjects will increase their dose to 30g, consuming 15g (3 unit doses) just prior to breakfast and 15g (3 unit doses) just prior to dinner. A telephone call from the site on Day 4 will remind the subject to increase the dose and will assess the status of the subject.
  - If a subject comes on Day 4 and says they were having problems with 15g, then the investigator and GSK Medical Monitor will decide whether the subject can continue in the study if the side effects are tolerable.
  - If a subject escalated to 30g on Day 4 and then contacts the clinical site to say that they cannot tolerate that dose, the subject should follow the following plan:
    - If the subject took 15g on the morning of the day of contact, they should skip the evening dose, and restart 15g again the next day (5g in morning and 10g in evening). The investigator and GSK Medical Monitor can then decide whether to try the subject again on 30g at a later time.
    - If the subject did not take a dose on the day of contact, they can start on 15g (5g in morning and 10g in evening) that same day. The investigator and GSK Medical Monitor can then decide whether to try the subject again on 30g at a later time.
- At the Day 7 visit, symptoms of tolerability will be reviewed with the subject, and a decision will be made regarding the dose for the next week:
  - If the subject is experiencing intolerable gastrointestinal side effects at 30g, the dose may be decreased to 15g for the remainder of the study.
  - If the subject is experiencing tolerable side effects at 30g but is unwilling to attempt a higher dose, the dose may remain at 30g for the remainder of the study.
  - If the subject is experiencing minimal side effects at 30g and is willing to attempt a higher dose, the dose should be increased to 40g for the next week, to be taken as 20g (4 unit doses) before breakfast and 20g (4 unit doses) before dinner.
- At the Day 14 visit, symptoms of tolerability should be reviewed with the subject.
- Subjects taking 40g may remain on that dose, or may decrease the dose to 30g, if the higher dose is not well tolerated.
- If the subject develops intolerable gastrointestinal side effects at 30g, the dose may be decreased to 15g for the remainder of the study.
- From Day 15 through Day 42, each subject will remain on a constant dose, unless in the opinion of the investigator and GSK Medical Monitor the dose should be reduced because of tolerability or safety issues
- **Important Note 1:** GSK2890457 and placebo are packaged identically, but differences are discernible when the powders are directly compared, both when dry and when mixed with water. For this reason, a specified unblinded member of the study site staff must demonstrate appropriate mixing of study medication with water according to instructions in Appendix 5: Directions for Taking Study Medicine and supervise drug administration in the clinic. When subjects are dosed at the study site, they will receive study treatment directly from the designated unblinded study staff member.

- **Important Note 2:** Some subjects may find it difficult to take GSK2890457, especially at the highest dose of 40g. To improve or maintain compliance with dosing, this protocol allows the Investigator some flexibility, in consultation with the GSK Medical Monitor, to modify the way a subject takes the components of GSK2890457 or placebo, during the 6-week Treatment Period. In all cases, once sachets are mixed with water they must be consumed soon after mixing. Subjects must be encouraged to remain well hydrated by drinking water, but not sugar-containing sodas, flavored drinks or fruit juices.

4.4.2. Liraglutide (Part B)

In Part B, T2D subjects randomized to liraglutide will stop their usual metformin dose and start liraglutide at a dose of 0.6mg QD by subcutaneous injection. Subjects will titrate to 1.2mg QD after 1 week, and will then increase to 1.8mg after another week, if tolerated. Subjects will stay on 1.8mg during the Stabilization and Treatment Periods. If 1.8mg is poorly tolerated, a subject can reduce the dose to 1.2mg for the remainder of the Stabilization and Treatment Periods.

Study staff will demonstrate how to inject liraglutide for subjects receiving liraglutide, and subjects will be provided the Victoza (liraglutide) Medication Guide (Appendix 7: Victoza (Liraglutide) Medication Guide for Patients), and will be made aware of symptoms that may be associated with acute pancreatitis.

After the Stabilization Period, subjects will be randomized to GSK2890457 or placebo in a 2:1 allocation. The randomization will be stratified by baseline HbA1c (<7.5% or ≥7.5%).

Subjects will continue dosing with liraglutide up to, and including, Day 42 of the Treatment Period. During the Treatment period, the morning dose of GSK2890457 should be taken before administration of liraglutide. As the gastric emptying delay produced by liraglutide may impact the ability of a subject to take the full dose of GSK2890457, the time interval between the GSK2890457 and liraglutide doses can be adjusted to improve tolerability and compliance with GSK2890457 dosing.

On Day 43 of the Treatment Period, subjects will re-commence their usual dose of metformin before discharge from the clinical unit, unless a dose modification is appropriate based on the clinical judgment of the investigator.

4.4.3. Metformin

4.4.3.1. Metformin (Part A)

In Part A, subjects will take single doses of metformin (supplied by the site). Subjects should take the Day 1 morning dose with 8oz of water. On Day 42, subjects can drink GSK2890457 in lieu of water with the metformin dose.

4.4.3.2. Metformin (Part C)

In Part C, T2D subjects will stay on their usual dose and regimen of metformin. As preliminary data from Part A indicate that GSK2890457 may reduce the AUC and Cmax

of metformin, subjects taking metformin once-daily should take that dose in the morning one hour prior to GSK2890457/placebo, followed by breakfast, throughout the Treatment period, to minimize a possible pharmacokinetic interaction. Throughout the Treatment period, subjects taking metformin twice-daily should take the morning dose as above, and the evening dose one hour prior to GSK2890457/placebo, and then eat dinner.

After the Stabilization Period, subjects will be randomized to GSK2890457 or placebo in a 2:1 allocation. The randomization will be stratified by baseline HbA1c (<7.5% or ≥7.5%).

This protocol allows the Investigator, in consultation with the GSK Medical Monitor, to modify the metformin dose and/or dosing regimens and/or the GSK2890457 dose and/or dosing regimen in Part C based on emergent PK and tolerability data from Part A.

4.5. Investigational Product and Other Study Treatment Dosage/Administration

	Study Treatment				
**Product name:**	**GSK2890457**	**Placebo**	**Liraglutide**	**Metformin**	**Deuterated Creatine (if available)**
**Dosage form:**	Powders and Capsule	Powders and Capsule	Injection	Tablet	Capsule
**Unit dose strength(s)/Dosage level(s):**	The 'unit' dose is a 5g kit containing three sachets of powder and 3 capsules. The three powders will be mixed into water	The 'unit' dose of placebo is three sachets of powder and 3 capsules. The three powders will be mixed into water	6mg/mL, 3mL injector pen that permits doses of 0.6mg, 1.2mg, and 1.8mg.	Part A: 500mg IR<br><br>Part C: Subject will remain on usual dose and form of metformin.	30mg
**Route/ Administration/ Duration:**	Oral<br>6 weeks dosing (Treatment Period)	Oral<br>6 weeks dosing (Treatment Period)	Subcutaneous injection<br>18 weeks dosing (Stabilization and Treatment Periods, Part B only)	Oral<br>Part A: Single doses on Day 1 and Day 42:<br><br>Part B: Subject continues usual metformin dose through Run-in, and resumes after Treatment Period<br><br>Part C: Subject continues usual metformin dose throughout study	Oral<br>A single dose on Day -2 and on Day 41 Parts B and C only
**Dosing instructions:**	See Section 4.4.1	See Section 4.4.1	See Section 4.4.2	As prescribed Take with 8oz of water or dose of GSK2890457.	See Section 7.6.4

	Study Treatment				
**Product name:**	**GSK2890457**	**Placebo**	**Liraglutide**	**Metformin**	**Deuterated Creatine (if available)**
**Manufacturer/ source of procurement:**	GSK	GSK	Site will provide Victoza (liraglutide) and needles, and will provide subject with the Victoza 'Medication Guide'	Part A: Site will supply metformin<br><br>Parts B & C: Subject will take their own supplies.	GSK

4.6. Dose Adjustment/Stopping Criteria

This protocol allows subjects to reduce their dose of GSK2890457 or liraglutide based on tolerability or other clinically significant AEs, as outlined in Section 4.4.

Subjects who are unable to tolerate a minimum daily dose of 15g of GSK2890457 must withdraw from the study.

Subjects who have clinically significant treatment-related AEs on a daily dose of 0.6mg liraglutide during the Treatment Period must be withdrawn from the study unless in the judgment of the investigator and GSK Medical Monitor there are circumstances that indicate a subject can continue in the study without compromising subject safety and wellbeing or study objectives and conclusions.

The maximum daily dose of GSK2890457 will not exceed 40g in Parts A-C.

The maximum daily dose of liraglutide will not exceed 1.8mg in Part B.

Subjects will remain on their usual metformin dose in Part C unless the dose and/or dosing regimen is altered based on emergent PK and tolerability data from Part A.

See Section 10 for withdrawal procedures for any subject withdrawn early from the study.

4.6.1. Dose Adjustment/Stopping Safety Criteria

4.6.1.1. Liver Chemistry Stopping Criteria

Liver chemistry threshold stopping criteria have been designed to assure subject safety and to evaluate liver event etiology (in alignment with the FDA premarketing clinical liver safety guidance).

Study treatment will be stopped for a subject if the following liver chemistry stopping criteria is met:

- ALT ≥ 3xULN

Refer to Section 13, Liver Chemistry Follow-up Procedures, for details of the required assessments if a subject meets the above criteria.

4.6.1.2. QTc Withdrawal Criteria

A subject who meets the criteria below will be withdrawn from the study. The QT correction formula used to determine discontinuation should be the same one used throughout the study.

1. QTcF > 500msec or
2. Change from baseline: QTcF >60msec

If subject has underlying right bundle branch block then the QTcF withdrawal criteria depends on the baseline value:

**Baseline QTcF value (with underlying bundle branch**	**QTcF withdrawal criteria**
<450msec	>500msec
450-480msec	≥530msec

Subjects with left bundle branch block are excluded from the study.

Withdrawal decisions are to be based on an average QTcF value of triplicate ECGs. If an ECG demonstrates a prolonged QT interval, obtain 2 more ECGs over a brief period, and then use the averaged QTcF values of the 3 ECGs to determine whether the subject should be discontinued from the study.

4.6.1.3. Withdrawal Criteria for Suspected Acute Pancreatitis (Part B)

A subject who, at any time after the first dose of liraglutide through the end of the Treatment Period, presents with signs and symptoms of possible acute pancreatitis (e.g., epigastric pain and abdominal tenderness, potentially associated with nausea and vomiting) will have blood drawn for the measurement of lipase and amylase, and imaging studies, as appropriate, and will be withdrawn from liraglutide while symptoms are investigated.

- If lipase or amylase >3-fold ULN, the subject should be asked to return to the clinical unit to reassess any symptoms and then requested to return for a follow-up value as soon as possible, but at least within one week.
    - Amylase isoenzymes will be measured in the follow up sample for all elevated amylase values.
- If a subject's symptoms resolve and the repeat lipase or amylase is <3-fold ULN, the subject may continue in the study if there are no circumstances that indicate an increased risk for the subject.

- If symptoms are suggestive of pancreatitis or biliary disease, and are supported by additional clinical, laboratory and/or imaging information, liraglutide and study medication will be discontinued immediately and the Investigator will follow the procedures for study termination.
- If a subject is asymptomatic and the lipase is >3-fold ULN or amylase is >3-fold ULN, the subject may continue in the study if there are no circumstances that indicate an increased risk for the subject. The subject should be asked to return to the clinical unit to reassess the clinical status and amylase/lipase levels and then requested to return for a follow-up value as soon as possible, but at least within one week.
- If the subject has a lipase or amylase >3-fold ULN on 2 consecutive visits, the study medicines should be stopped permanently.

For those meeting criteria for study withdrawal due to pancreatic chemistry abnormalities:

- An ultrasound and MRI of the liver, gallbladder, and pancreas should be obtained within 24-48 hours.
- A detailed medical history should be recorded, in association with any reported symptoms, and further gastroenterological assessment should be considered.

4.6.1.4. Dose Adjustment/Stopping Safety Criteria Based on Blood Glucose Monitoring (Part B and C)

Subjects who complete successfully the screening procedures prior to dosing in Parts B and C must check fasting (pre-breakfast; minimum 8-hour fast) and pre-dinner CBG, and at any time symptoms of hypoglycemia or hyperglycemia (e.g., polyuria, polydipsia) are experienced or they are concerned about their blood glucose level. They must transmit the values for 'real-time' monitoring by the Investigator and GSK Medical Monitor.

Subjects enrolled in Parts B and C will be given written instructions on how to recognize and manage hypoglycaemia.

Hypoglycemia is defined as symptoms consistent with hypoglycemia (e.g. dizziness, light-headedness, shakiness) which are confirmed by glucometer measurement of CBG or plasma glucose value of <50mg/dL for Part A or <70mg/dL for Parts B and C (if possible, CBG values should be confirmed with a laboratory measurement). In situations when no glucose sample can be measured at the time of the event, the investigator may, at his or her discretion, characterize an event as 'hypoglycemia' based on reported signs and symptoms alone. Note: healthy subjects may have asymptomatic blood glucose values <70mg/dL as a physiological response to altered food intake (e.g., fasting).

While the risk of hypoglycemia is considered to be low, as summarized in Section 1.3, careful monitoring for signs and symptoms of hypoglycemia is important in this study, particularly if a subject's food intake is significantly reduced.

When subjects are in the clinical unit they should alert study staff any time they experience symptoms that might be related to hypoglycemia. Study staff should then test their blood glucose values.

Subjects are required to call the study coordinator while not in the clinical unit:

When they have symptomatic hypoglycemia, even if not confirmed by their blood glucose values.

- When they have fasting CBG values that are >270mg/dL or < 70mg/dL. (When the subject calls the site, site staff should confirm that values being reported were taken correctly, e.g., in the fasted state. Any deviations must be noted in the eCRF).
- When they have any concerns relating to their CBG levels.
- When they have rapid, unexplained changes in their blood glucose levels.

In order to ensure that hypoglycemia is reported in a consistent manner, the following scales will be used to characterize intensity of event and degree of intervention required (Table 4 and Table 5). Hypoglycemic events will be captured in the eCRF on a designated page rather than the AE page. Any events meeting the criteria of an SAE will also be recorded as an SAE.

Table 4 Hypoglycemia Intensity Scale

Category	Description
Mild	An event that is easily tolerated by the subject, causing minimal discomfort and not interfering with normal everyday activities. This includes events of asymptomatic 'biochemical' hypoglycemia when CBG or plasma glucose is <50mg/dL (Part A) or <70mg/dL (Parts B and C)
Moderate	An event that is sufficiently discomforting to interfere with normal everyday activities.
Severe	An event that prevents normal everyday activities.
Serious	SAE, life-threatening or required the subject to be admitted to the hospital.

Table 5 Hypoglycemia Intervention Scale

Category	Description
1	No intervention needed
2	Minor: administered sugary drinks or sweets
3	Intermediate: glucose drinks and supplements
4	Extensive: glucose injection / infusion / glucagon

All incidents of hypoglycemia will be reviewed to determine the relationship to study medication(s), and will be considered in conjunction with other adverse events in determining appropriate alteration of dose(s). Consideration should be given to simple actions such as having the subject drink orange juice, which provides oral glucose supplementation.

4.6.1.4.1. Part A (Healthy Subjects)

- In the unlikely event that the healthy subjects report clinically significant symptoms of hypoglycemia or CBG values are <50mg/dL on 2 occasions separated by a few

hours, the dose of GK2890457 may be reduced after consultation with the GSK Medical Monitor.

- Subjects may continue on the same dose of GSK2890457 if the episodes of hypoglycemia are not considered clinically significant.

4.6.1.4.2. Part B (Liraglutide Add-on)

Stabilization Period: In the unusual event that a T2D subject randomized to liraglutide experiences symptoms/signs of hypoglycemia confirmed by a CBG value or laboratory plasma glucose value <70mg/dL, the dose of liraglutide may be reduced from 1.8mg to 1.2mg.

If a subject can only tolerate 1.2mg during the initial titration phase, but still experiences hypoglycemia, then the Investigator and GSK Medical Monitor should discuss the circumstances to determine whether (i) the episodes are not clinically significant and the subject can continue on the same dose of liraglutide, or (ii) the subject should be withdrawn from the study.

Treatment Period: In the unlikely event that a T2D subject who has progressed uneventfully through the Stabilization Period experiences symptoms/signs of hypoglycemia confirmed by a capillary glucose value or laboratory plasma glucose value <70mg/dL, reduction of the dose of liraglutide or GSK2890457 or discontinuation of further dosing should be considered for the affected subject, in consultation with the GSK Medical Monitor.

4.6.1.4.3. Part C (Metformin Add-on)

If a subject experiences symptoms/signs of hypoglycemia confirmed by a capillary glucose value or laboratory plasma glucose value <70mg/dL, a reduction of the dose of GSK2890457 and/or metformin or discontinuation of further dosing should be considered for the affected subject, in consultation with the GSK Medical Monitor.

4.6.1.5. Withdrawal Criteria for Renal Impairment

Any subject experiencing an increase in serum creatinine of ≥ 0.5mg/dL from baseline (based on mean of screening and Day -1 values) will be discontinued from further dosing.

4.6.1.6. Dose Adjustment Based on Reduced Food Intake

Based on animal model data, GSK2890457 may reduce food intake in humans.

In order to minimize the risk of hypoglycaemia, the Investigator, in consultation with the GSK Medical Monitor, may adjust the dose of GSK2890457, liraglutide or metformin if the degree of reduction of food intake is considered to pose a potential risk to the subject.

4.6.1.7. Monitoring and Stopping Criteria for Allergic Reactions to Liraglutide

Although it is considered unlikely for acute allergic reactions to occur in response to liraglutide exposure, all subjects will be monitored carefully for evidence of an allergic response and discontinued from further dosing in Part B in the event of an acute allergic reaction. As part of the assessment, the Investigator should document whether there was

any previous exposure to a GLP-1 receptor agonist or participation in a clinical trial involving one of these drugs.

While rare, it is important to recognize early signs of an anaphylactoid reaction and prevent progression to severe anaphylaxis. Patients will be closely monitored in the clinical unit and will be advised while at home to report early signs of dyspnea and angioedema. Antihistamines, such as diphenhydramine; and corticosteroids, such as prednisone, may be given to reduce symptoms and signs. Inhaled bronchodilators may be administered.

If more severe clinical signs arise, then the physician will assess the subject to determine if cardiopulmonary resuscitation is required. Epinephrine may be given by intramuscular injection without delay. Emergency interventions by paramedics or physicians may include endotracheal intubation or tracheostomy. Treatment for shock may include administration of intravenous fluids and medications that support cardiac and circulatory function.

4.6.1.8. Stopping Criteria for Suicidal Ideation

Although liraglutide has not been shown to be associated with an increased risk of suicidal thinking or behaviour when given to T2D patients, GSK considers it important to monitor for such events in this clinical study.

Subjects receiving liraglutide in Part B should be assessed and monitored appropriately for suicidality and unusual changes in behaviour. In consultation with the GSK Medical Monitor, consideration should be given to discontinuing liraglutide in subjects who experience signs of suicidal ideation or behaviour.

Adverse events related to suicidal ideation will be reported in the designated “Possible suicidality related AE” page in the eCRF, rather than the AE page

4.6.1.9. Other Dose Adjustment and Stopping Criteria for Safety

In addition to the criteria specified above, all laboratory abnormalities, ECG abnormalities, changes in vital signs, and other adverse events will be reviewed periodically to determine whether an alteration of the dose of study medication(s) or discontinuation of dosing may be necessary to ensure appropriate subject safety.

Based on safety concerns and/or adverse events, subjects may be discontinued from further dosing as deemed appropriate by the Investigator, in consultation with the GSK Medical Monitor.

4.6.1.10. Subject Withdrawal for Other Reasons

A subject with a positive urine drug screen will be withdrawn unless in the judgment of the Investigator and GSK Medical Monitor there are extenuating circumstances that indicate the absence of illicit drug abuse.

4.7. Time and Events Tables

4.7.1. Part A Time and Events Table – Healthy Subjects

	Screening	Day 1	Day 4 (± 1 Day)	Day 7 (± 1 Day)	Day 14 (± 1 Day)	Day 21 (± 1 Day)	Day 28 (± 1 Day)	Day 35 (± 1 Day)	Day 42 (± 1 Day)	Follow-up Visit
**Visit Timing (relative to Day 1)**	**Maximum of -28 days**									**Day 56 (± 1 Day)**
Clinic Visit	X	X		X	X		X		X	X
Phone Contact			X			X		X		
Informed Consent	X									
Demographics	X									
Complete Physical	X									
Brief Physical										X
Medical/medication/drug/alcohol history	X									
Vital Signs	X	X (pre-metformin)		X	X		X		X	X
12-Lead ECG Single	X								X	X
12-Lead ECG Triplicate		X (pre-metformin)								
Height	X									
Body Weight (in clinic)	X	X		X	X		X		X	X
Urine drug/alcohol screen	X	X (pre-metformin)		X	X		X		X	
Urine beta-HCG (women)		X (pre-metformin)								
Hematology/Chemistry/Urinalysis	X	X (pre- metformin)		X	X		X		X	X
HIV, Hep B and Hep C, TSH Screen	X									
Waist circumference		X							X	
GI Symptoms Rating Scale Questionnaire (GSRS)		X (pre-metformin)		X	X				X	
Metformin PK		X							X	

	Screening	Day 1	Day 4 (± 1 Day)	Day 7 (± 1 Day)	Day 14 (± 1 Day)	Day 21 (± 1 Day)	Day 28 (± 1 Day)	Day 35 (± 1 Day)	Day 42 (± 1 Day)	Follow-up Visit
**Visit Timing (relative to Day 1)**	**Maximum of -28 days**									**Day 56 (± 1 Day)**
Fasting Glucose (minimum 8-hour fast)		X (pre-metformin)		X	X		X		X	
Dispense/Instruct: Study Medication		X		X	X		X			
Randomization		X (pre-metformin)								
Twice-Daily Dosing GSK2890457			------------------------------X------------------------------							
Evening Dose GSK2890457		X								
Metformin Single 500mg dose		X							X	
Identical Meals		X							X	
Concomitant Medication Review	X	X	X	X	X	X	X	X	X	X
AE Assessment		X	X	X	X	X	X	X	X	X

4.7.2. Part B Time and Events Table: Screening, 1-Week Telemonitoring Run-in and 12-Week Stabilization

	Screening	Telemonitoring "Procedures Familiarization" Run-In	Stabilization					
**Visit Timing**	**Maximum of -28 days pre-Stabilization**	**13 weeks prior to Treatment Period**	**Week 1 of Stabilization Period**	**Week 2 of Stabilization Period**	**Week 3 of Stabilization Period**	**Week 5 of Stabilization Period**	**Week 7 of Stabilization Period**	**Week 10 of Stabilization Period**
Clinic Visit	X	X	X		X[1]		X	X
Phone Contact				X		X		
Informed Consent	X							
Demographics	X							
Complete Physical (including detailed psychiatric history)	X							
Brief Physical (including thyroid examination)			X		X		X	X
Suicidality Monitoring with C-SSRS	X		X				X	
Medical/medication/drug/alcohol history	X							
Vital Signs	X		X				X	X
12-Lead ECG Single	X							
Height	X							
Body Weight (in clinic)	X		X		X		X	X
Waist circumference			X		X		X	X
Urine drug/alcohol screen	X		X		X		X	X
Hematology/Chemistry/Urinalysis (urine albumin and creatinine only at Screening)	X		X					
HbA1c	X		X					
Fasting Glucose and Insulin (minimum 8-hour fast)	X		X				X	
C-peptide	X							
HIV, Hep B and Hep C Screen, TSH	X							
Calcitonin	X							

	**Screening**	**Telemonitoring "Procedures Familiarization" Run-In**	**Stabilization**					
**Visit Timing**	**Maximum of - 28 days pre-Stabilization**	**13 weeks prior to Treatment Period**	**Week 1 of Stabilization Period**	**Week 2 of Stabilization Period**	**Week 3 of Stabilization Period**	**Week 5 of Stabilization Period**	**Week 7 of Stabilization Period**	**Week 10 of Stabilization Period**
Lipase and Amylase	X		X		X			X
Urine beta-HCG (women)			X					
Dispense/instruct: scale and glucometer		X						
Daily fasting and pre-dinner glucometer readings at home		----------X----------						
Daily fasting weight at home		----------X----------						
GI Symptom Rating Scale Questionnaire (GSRS)			X				X	
Dispense/ Instruct/Reinforce: Mindful Eating Guide to Losing Weight			X		X		X	X
Dose Liraglutide			----------X----------					
Increase Liraglutide Dose				X	X			
Concomitant Medication Review	X	X	X	X	X	X	X	X
AE Assessment		X	X	X	X	X	X	X

1. At Week 3 of the Stabilization Period, a clinic visit will include a short review in unit, and dispensing liraglutide. Because the 1.8 pen lasts less than a month, subject re-supply needs to be managed closely.

4.7.3. Part C Time and Events Table: Screening, 1-Week Telemonitoring Run-in and 4-Week Stabilization for Subjects Remaining on Metformin

	Screening	Telemonitoring "Procedures Familiarization" Run-In	Stabilization	
**Visit Timing**	**Maximum of -28 days pre-Stabilization**	**5 weeks prior to Treatment Period**	**Week 1 of Stabilization Period**	**Week 3 of Stabilization Period**
Clinic Visit	X	X	X	
Phone Contact				X
Informed Consent	X			
Demographics	X			
Complete Physical	X			
Brief Physical				
Medical/medication/drug/alcohol history	X			
Vital Signs	X		X	
12-Lead ECG Single	X			
Height	X			
Body Weight (in clinic)	X		X	
Waist circumference			X	
Urine drug/alcohol screen	X		X	
Hematology/Chemistry/Urinalysis (urine albumin and creatinine only at Screening)	X		X	
HbA1c	X		X	
Fasting Glucose and Insulin (minimum 8-hour fast)	X		X	
C-peptide	X			
HIV, Hep B and Hep C Screen, TSH	X			
Urine beta-HCG (women)			X	
Dispense/instruct: scale and glucometer		X		
Daily fasting and pre-dinner glucometer readings at home		----------------------------X----------------------------		
Daily fasting weight at home		----------------------------X----------------------------		
GI Symptom Rating Scale (GSRS)			X	
Dispense/ Instruct/Reinforce: Mindful Eating			X	X

	Screening	Telemonitoring "Procedures Familiarization" Run-In	Stabilization	
**Visit Timing**	**Maximum of -28 days pre-Stabilization**	**5 weeks prior to Treatment Period**	**Week 1 of Stabilization Period**	**Week 3 of Stabilization Period**
Guide to Losing Weight				
Dose Metformin		----------X----------		
Concomitant Medication Review	X	X	X	X
AE Assessment			X	X

4.7.4. Parts B and C Time and Events Table Day -2 through Study End

Procedure	Day -2	Day -1	Day 1	Day 4 (±1 Day)	Day 7 (±1 Day)	Day 14 (±1 Day)	Day 21 (±1 Day)	Day 28 (±1 Day)	Day 35 (±1 Day)	Day 41	Day 42	Day 43	Follow-up (±1 Day)
**Visit Timing (relative to Day 1)**													**Day 56**
Clinic Visit	--In-clinic---				X	X		X		--In-clinic---			X
Phone Contact				X			X[1]		X[1]				
Brief Physical (including thyroid examination in Part B only)	X									X			X
Suicidality monitoring with C-SSRS (Part B only)	X									X			X
Vital Signs		X	X		X	X		X			X	X	X
12-Lead ECG Triplicate		X											
12-Lead ECG Single											X		X
Body Weight (in clinic)		X	X		X	X		X			X	X	X
Waist circumference		X			X	X		X			X		
Urine drug/alcohol screen	X				X	X		X		X			
Hematology/Chemistry/Urinalysis		X			X	X		X			X		X
HbA1c		X									X		
T3/T4/TSH		X									X		
Urine beta-HCG (women)	X												
Liraglutide subjects: continue liraglutide (Part B)	----------X----------												
Restart Metformin (Part B)												X	
Metformin subjects: continue metformin (Part C)	----------X----------												
Daily fasting and pre-dinner glucometer readings at home	----------X----------												
Daily fasting weight at home	----------X----------												
Standardized evening meal	X									X			
Standardized breakfast, lunch and dinner		X									X		
Food Frequency Questionnaire	X												
GI Symptom Questionnaire (GSRS)	X				X	X		X		X			
Hunger Questionnaire		X									X		
Background Questions/ End of Study Questions	X									X			
CRP, Lipid Panel		X									X		
Deuterated creatine dose (in afternoon, if available)	X									X			

Procedure	Day -2	Day -1	Day 1	Day 4 (±1 Day)	Day 7 (±1 Day)	Day 14 (±1 Day)	Day 21 (±1 Day)	Day 28 (±1 Day)	Day 35 (±1 Day)	Day 41	Day 42	Day 43	Follow-up (±1 Day)
Morning urine for deuterated-creatine analytes (if deuterated creatine dose administered)			X									X	
Urine for background deuterated-creatine (prior to second deuterated creatine dose, if deuterated creatine dose administered)										X			
25 Hydroxy Vitamin D		X											
Lipase and Amylase (Part B only)		X									X		X
PK Blood sampling		X									X		
Glucose/Insulin Blood Sampling (See Section 4.7.6)		X									X		
Fasting Biomarker Samples		X									X		
Drug Metabolism Sampling – blood		X									X		
24-hour urine for assessment of GSK2890457 metabolites		X									X		
DEXA	X									X			
Fecal Sampling	X									X			
Dispense/Instruct: Study Medication			X		X	X		X					
Dispense/ Instruct/Reinforce: Mindful Eating Guide to losing weight		X				X		X					
Randomization			X										
Twice-Daily Dosing of Study Medication							----------------------------X----------------------------						
Concomitant Medication Review	X				X	X		X		X			X
AE Assessment	X	X	X	X	X	X		X		X	X	X	X
Subject Returns Telemonitoring Equipment													X

1. If subjects have difficulty in carrying 2-weeks drug supply home, they may come in to the clinic for weekly drug supply, and the status check performed by the phone calls done at this brief clinic visit.

4.7.5. Part A: Day 1 and Day 42: Sampling Schedule for Metformin PK Timepoints

Timepoint	Pharmacokinetic Sample[1] (metformin)
Fasting	X (Pre-metformin on Day 1 and predose on Day 42 within 15 minutes of dose)
Time 0 minutes	Dose metformin alone (Day 1) or with GSK2890457 (Day 42)
Begin eating breakfast immediately after taking study medication(s) and finish eating in 15min[2]	
15minutes	X
30 minutes	X
1 hour	X
1.5 hours	
2 hours	X
4 hours (Pre-Lunch)	X
4 hours	Eat Lunch
5.5 hours	X
6 hours	
8 hours	X
10 hours (Pre-Dinner)	X
10 hours (Pre-Dinner)	GSK2890457 Dose

1. See Section 7.5 for instructions for PK samples. Samples for analysis of metformin will be collected in EDTA containing tubes.
2. Subjects should make their best effort to consume dose and meal in the allotted time; if unable to complete in time, this will not be considered a protocol violation.

4.7.6. Part B: Day -1 and Day 42: Sampling Schedule for Glucose, Insulin, Biomarkers, and PK; Satiety Questionnaire Timepoints

Timepoint	Glucose and Insulin Sample	Other Biomarker Samples	Pharmacokinetic Sample[1] (liraglutide)	Fasting Exploratory Biomarker Samples	Blood for GSK2890457 components/metabolites[2] Day -1 and Day 42	Hunger Questionnaire	Urine for GSK2890457 components/ metabolites Day -1 and Day 42[3]
**Fasting**	**X[4]**						
**Fasting**	**X[4]**	**X**	**X (predose)**	**X**	**X (pre-dose)**	**X**	
Time 0 minutes: Dose liraglutide[6] alone (Day -1) or with GSK2890457 (Day 42)							
Begin eating breakfast immediately after taking study medication(s) and finishing eating in 15min[5]							
15minutes			X		X		
30 minutes	X	X	X		X		
1 hour	X	X	X		X	X	
1.5 hours	X	X					
2 hours	X	X	X		X	X	
4 hours (Pre-Lunch)	X	X	X		X	X	Pool 0-12hr
4 hours			Eat Lunch				
5.5 hours	X	X	X		X		
6 hours						X	
8 hours			X		X	X	
10 hours (Pre-Dinner)	X	X	X		X		
10 hours	Take evening dose of GSK2890457 and then eat dinner/evening meal						
11.5 hours	X	X	X		X	X	
12 hours							Pool 12-24hr
14 (bedtime)	X	X					
24 hours	X	X	X (predose, morning dose)				

1. See Section 7.5 for instructions for PK samples. Samples for analysis of liraglutide will be collected in EDTA containing tubes.
2. A single sample will be collected at each timepoint and quickly processed as described in Section 7.5.2. As some of the components of GSK2890457 are unstable in plasma, the blood for GSK2890457 PK needs to be collected in special tubes containing Potassium Oxalate/sodium fluoride and the blood quickly processed as described in Section 7.5.
3. Urine will be collected at the various intervals and the volume will be determined. A 100mL aliquot from each time interval will be used for the investigation of GSK2890457 metabolites.

4. Two fasting samples 5min apart will be taken for insulin. Baseline insulin level will be the average of the 2 fasting samples.
5. Subjects should make their best effort to consume dose and meal in the allotted time; if unable to complete in time, this will not be considered a protocol violation.
6. The time interval between the dose of liraglutide and GSK2890457 may be varied if gastric emptying delay impacts the ability to take the full dose of GSK2890457.

4.7.7. Part C: Day -1 and Day 42: Sampling Schedule for Glucose, Insulin, Biomarkers, and PK; Satiety Questionnaire Timepoints

Timepoint	Glucose and Insulin Sample	Other Biomarker Samples	Pharmacokinetic Sample[1] (metformin)	Fasting Exploratory Biomarker Samples	Blood for GSK2890457 components/metabolites[2] Day -1 and Day 42	Hunger Questionnaire	Urine for GSK2890457 components/ metabolites Day -1 and Day 42[3]
**Fasting (F1)**	**X**						
**Metformin Dose + Snack**			**1 hour prior to GSK2890457 dose**				
**F2**	**X**	**X**	**X (pre GSK2890457 dose)**	**X**	**X (pre-dose)**	**X**	
Time 0 minutes: Dose GSK2890457 (finish within 5 min) (Day 42)							Pool 0-12hr
Begin eating breakfast immediately after taking GSK2890457 and finishing eating in 15min[4]							
15minutes			X		X		
30 minutes	X	X	X		X		
1 hour	X	X	X		X	X	
1.5 hours	X	X					
2 hours	X	X	X		X	X	
4 hours (Pre-Lunch)	X	X	X		X	X	
4 hours	Eat Lunch						
5.5 hours	X	X	X		X		
6 hours						X	
8 hours			X		X	X	
9 hours Metformin + snack			1 hour prior to dinner				
10 hours (Pre-Dinner)	X	X	X		X		
10 hours	Take GSK2890457 (Day 42) and then eat dinner/evening meal						
11.5 hours	X	X	X		X	X	
12 hours							Pool 12-24hr
14 (bedtime)	X	X					
24 hours	X	X	X (predose, morning dose)				

1. See Section 7.5 for instructions for PK samples. Samples for analysis of metformin or liraglutide will be collected in EDTA containing tubes.
2. A single sample will be collected at each timepoint and quickly processed as described in Section 7.5.2. As some of the components of GSK2890457 are unstable in plasma, the blood for GSK2890457 PK needs to be collected in special tubes containing Potassium Oxalate/sodium fluoride and the blood quickly processed as described in Section 7.5.
3. Urine will be collected at the various intervals and the volume will be determined. A 100mL aliquot from each time interval will be used for the investigation of GSK2890457 metabolites.
4. Subjects should make their best effort to consume dose and meal in the allotted time; if unable to complete in time, this will not be considered a protocol violation.

5. STUDY POPULATION

One cohort of healthy volunteers and two cohorts of subjects with T2D will be enrolled. Subjects with T2D must be taking metformin as their only antidiabetic therapy, at a total daily dose of at least 850mg:

- **Part A:** Approximately14 healthy subjects (to complete approximately 9 randomized to active treatment / approximately 3 to placebo). Safety and tolerability will be evaluated for this cohort prior to initiation of dosing in Parts B and C.
- **Part B:** Approximately 21 enrolled for the Run-in and Stabilization Periods, to yield approximately 18 subjects with T2D (approximately 12 to be randomized to active treatment / approximately 6 to placebo). These subjects will discontinue metformin and replace it with liraglutide therapy.
- **Part C:** Approximately 21 enrolled for the Run-in and Stabilization Periods, to yield ~18 subjects (approximately 12 to be randomized to active treatment / approximately 6 to placebo). These subjects will continue on their prior metformin dose.

If subjects prematurely discontinue the study, additional subjects may be enrolled as replacement subjects, at the discretion of the Sponsor.

5.1. Eligibility Criteria

Deviations from eligibility criteria are not allowed because they can potentially jeopardize the scientific integrity of the study, regulatory acceptability or subject safety. Therefore, adherence to the criteria as specified in the protocol is essential.

5.1.1. Screening Inclusion Criteria for Part A (Healthy Subjects)

A subject will be eligible for inclusion in Part A of this study only if all of the following criteria apply:

1. Consent

Demonstrates understanding of the study and willingness to participate as evidenced by voluntary written informed consent and has received a signed and dated copy of the informed consent form.

2. Age and Body Mass Index (BMI)
   - 18 - 70 years of age, inclusive, at the time of signing the informed consent.
   - BMI between 18.0 and 35.0, inclusive.
3. Compliance
   - Understands and is willing, able and likely to be compliant with taking study drug and comply with all study procedures and restrictions.
   - Able to take capsules.
   - Subject is willing to consume the foods that are part of the standardized breakfast, lunch, and dinner.

4. General Health

   - In good general health with (in the opinion of the Investigator) no clinically significant and relevant abnormalities of medical history or physical examination that would introduce additional risk factors or interfere with study procedures or objectives.
   - Adequate renal function, as evidenced by the MDRD estimate of glomerular filtration rate ≥ 80mL/min. (A MDRD calculator may be found at http://mdrd.com.)
   - ALT, alkaline phosphatase and bilirubin ≤ 1.5xULN (isolated bilirubin >1.5xULN is acceptable if bilirubin is fractionated and direct bilirubin <35%).
   - QTcF < 450msec; or QTcF < 480msec for subjects with right Bundle Branch Block. Subjects with left Bundle Branch Block are excluded.

5. Female Subjects

   - Females must be post-menopausal, defined as 12 months of spontaneous or post-surgical amenorrhea. In questionable cases, a blood sample with contemporaneous values of follicle stimulating hormone (FSH) > 40mlU/mL and estradiol <40pg/mL (<140pmol/L) is confirmatory in the absence of a clear post-menopausal history.
   - Hormonal replacement therapy is permitted. Females on hormone replacement therapy (HRT) must discontinue HRT to allow confirmation of post-menopausal status prior to study enrollment. For most forms of HRT, at least 2-4 weeks will elapse between the cessation of therapy and the blood draw; this interval depends on the type and dosage of HRT. Following confirmation of their post-menopausal status, they can resume use of HRT during the study. Subjects should be informed that uterine bleeding may occur on withdrawal or after re-starting HRT. Subjects with a history compatible with post-menopausal status as defined above, who subsequently started HRT may be eligible if approved by the GSK Medical Monitor.
   - Females who are > 3 months postpartum and who have undergone one of the following surgical sterilization procedures are eligible to participate:
     - A documented hysterectomy, or
     - A documented bilateral oophorectomy, or
     - A documented tubal ligation.

5.1.2. Screening Exclusion Criteria for Part A (Healthy Subjects)

1. Disease

   - History of GI disease (e.g., irritable bowel disease, chronic or current diarrhea, inflamed bowel, steatorrhea/fat malabsorption, celiac disease, symptomatic lactose intolerance).

- Current or history of liver disease, or known hepatic or biliary abnormalities (with the exception of Gilbert's syndrome or asymptomatic gallstones). . Subjects with a history of cholelithiasis and uncomplicated cholecystectomy more than 3 months before Screening may be eligible if approved by the GSK Medical Monitor.
- History of serious, severe or unstable physical or psychiatric illness, including depression, suicidal thoughts, schizophrenia, bipolar disorder, or generalized anxiety disorder. In addition to elicited symptoms and signs, this should include specific questions relating to known psychiatric diagnoses and medications used.
- History of surgery for weight loss at any time or other gastrointestinal surgery within 3 months of Screening.
- History of sensitivity to environmental dusts or allergens (including hayfever), unless of mild severity in the opinion of the investigator and approved by the GSK Medical Monitor.
- History of asthma and/or use of inhaled bronchodilators or steroids.
- Any documented or reported eating disorder that may impact compliance with study medication.
- Uncontrolled hypertension, as evidenced by systolic pressure>160 or diastolic pressure >90.
- Positive test for HIV, Hepatitis B, or Hepatitis C at Screening.
- Significant ECG abnormalities, defined as follows:

Heart Rate	< 50 and >100bpm
PR Interval	<120 and > 220msec
QRS duration	< 70 and >120msec
QTcF Interval	> 450msec (>480msec if right bundle branch block)

- Current or relevant previous significant medical disorder that may require treatment or make the subject unlikely to fully complete the study, or any condition that, in the opinion of the investigator, presents undue risk from the study medication or procedures.

2. Thyroid Disease
   - Uncorrected Thyroid Dysfunction: Fasting plasma thyroid stimulating hormone (TSH) outside of the normal range, as determined at the Screening visit.
   - Subjects on stable thyroid replacement therapy and with TSH in the normal range are eligible if approved by the GSK Medical Monitor.
3. Diet- and Exercise-Related Criteria
   - Dietary restrictions or behaviors that prevent compliance with consumption of study medication.
   - Reported increased sensitivity to intestinal discomfort after consuming relatively small (e.g., <10g) amounts of any non-digestible carbohydrates and/or fibers.

4. Substance Abuse

   - Current or recent history (within one year of screening) of alcohol or other substance abuse, including abuse of prescription medications, with alcohol abuse defined as an average weekly intake of >14 drinks for males or >7 drinks for females. One drink is equivalent to 12g of alcohol: 12 ounces (360mL) of beer, 5 ounces (150mL) of wine or 1.5 ounces (45mL) of 80 proof distilled spirits.
   - A positive urine drug of abuse screen at Screening.

5. Nicotine Use

   - Unwilling to stop smoking while in the clinical unit.

6. Medication

   - Unable to refrain from the use of non-prescription drugs, including vitamins, herbal and dietary supplements (including St John's Wort) within 7 days (or 14 days if the drug is a potential enzyme inducer) or 5 half-lives (whichever is longer) prior to the first dose of study medication, unless in the opinion of the Investigator and GSK Medical Monitor the medication will not interfere with the study procedures or compromise subject safety.
   - Use of dietary or fiber supplements within 14 days prior to randomization which may have an effect on weight or appetite, including but not limited to nutrition and/or OTC products marketed for appetite or weight control. If high-fiber foods are part of the subject's normal diet, the foods may continue through the trial.
   - Use of any medications within 14 days before start of Treatment Period that may have the potential to interact with the effects of GSK2890457, including oral antibiotics, bile acid sequestrants, protein-pump inhibitors, H2 antagonists, probiotics, herbal and nutraceutical products intended to impact gut health and use of stomach 'coating' agents, e.g., Pepto-Bismol, Kaopectate.

7. Clinical Study/Experimental Medication Participation

   - Current participation in another clinical study or participation in a clinical study involving an investigational drug within 30 days of the screening visit.
   - Exposure to more than four new chemical entities within 12 months prior to the first dosing day.
   - Previous participation in this study.
   - Where donation of blood or blood products pre-study and participation in the study would result in excess of 500mL blood depletion within a 56-day period.

8. Sensitivity/Allergy

   - History of sensitivity to any of the study medications, or components thereof or a history of drug or other allergy that, in the opinion of the investigator or GSK Medical Monitor, contraindicates their participation.
   - Sensitivity to heparin, if heparin may be used to maintain catheter patency.

- If the subject has food allergies, the GSK Medical Monitor should be consulted to confirm enrollment is appropriate.

9. Personnel

- An employee of the sponsor or the study site or members of their immediate family.

5.1.3. Screening Inclusion Criteria for Type 2 Diabetic Subjects (Parts B and C)

A subject will be eligible for inclusion in Parts B and C of the study only if all of the following criteria apply:

1. Consent

- Demonstrates understanding of the study and willingness to participate as evidenced by voluntary written informed consent and has received a signed and dated copy of the informed consent form.

2. Age and Body Mass Index (BMI)

- 18 - 70 years of age, inclusive, at the time of signing the informed consent.
- BMI between 30.0 and 42.0 kg/m2, inclusive.

3. Compliance

- Understands and is willing, able and likely to be compliant with taking study medication and to comply with all study procedures and restrictions, including weighing daily at home with a telemonitored scale and monitoring blood glucose daily throughout the study and downloading those data to a telemonitoring system.
- Able to take capsules.
- Subject is willing to consume the foods that are part of the standardized breakfast, lunch, and dinner.

4. General Health

- In good general health with (in the opinion of the investigator) no clinically significant and relevant abnormalities of medical history or physical examination that would introduce additional risk factors or interfere with study procedures or objectives, based on a medical evaluation including medical history, physical examination, vital signs and laboratory tests.
- ALT, alkaline phosphatase and bilirubin ≤ 1.5xULN (isolated bilirubin >1.5xULN is acceptable if bilirubin is fractionated and direct bilirubin <35%).
- QTcF < 450msec; or QTcF < 480msec in subjects with right Bundle Branch Block. Subjects with left Bundle Branch Block are excluded.

5. Female Subjects

- Females must be post-menopausal, defined as 12 months of spontaneous or post-surgical amenorrhea. In questionable cases, a blood sample with

contemporaneous values of follicle stimulating hormone (FSH) > 40mlU/mL and estradiol <40pg/mL (<140pmol/L) is confirmatory in the absence of a clear post-menopausal history.

- Hormonal replacement therapy is permitted only if dose is constant, i.e., no hormonal fluctuations are occurring that may impact weight. Females on hormone replacement therapy (HRT) must discontinue HRT to allow confirmation of post-menopausal status prior to study enrollment. For most forms of HRT, at least 2-4 weeks will elapse between the cessation of therapy and the blood draw; this interval depends on the type and dosage of HRT. Following confirmation of their post-menopausal status, they can resume use of HRT during the study. Subjects should be informed that uterine bleeding may occur on withdrawal or after re-starting HRT. Subjects with a history compatible with post-menopausal status as defined above, who subsequently started HRT may be eligible if approved by the GSK Medical Monitor.
- Females who are >3 months postpartum and who have undergone documented bilateral oophorectomy are eligible to participate.

6. Type 2 Diabetes
    - Diagnosis of T2D for at least 3 months, as defined by the American Diabetes Association.
    - HbA1c of 7.5 to 11, inclusive, at Screening visit.
    - C-peptide of >1ng/mL at Screening visit.
    - Urine albumin excretion <30mg/g creatinine.
    - Calcitonin at Screening ≤ 50pg/mL (Part B only)
    - Maintained on a stable dose of metformin for at least three months, at a daily dose of at least 850mg.
    - Not taking other anti-diabetic medications.
7. All T2D subjects must meet label recommendations for metformin, including:
    - Adequate renal function, as evidenced by the MDRD estimate of glomerular filtration rate ≥ 60mL/min.
    - No conditions which make hypoxia, dehydration, or sepsis likely.
    - No clinical or laboratory evidence of hepatic disease (including history of cholecystitis or symptomatic gallstones) and cardiac disease (including a history of myocardial infarction or heart failure). Subjects with a history of cholelithiasis and uncomplicated cholecystectomy more than 3 months before Screening may be eligible if approved by the GSK Medical Monitor.
    - No excessive alcohol intake.
8. For Part B only: Subjects must be willing to discontinue metformin and replace it with daily liraglutide administered by subcutaneous injection during the twelve-week Stabilization Period and the six-week Treatment Period. In addition, they must meet label recommendations for liraglutide (Victoza), including:

9. For Part B only: No personal history or family history of medullary or papillary thyroid carcinoma or Multiple Endocrine Neoplasia syndrome type 2.

Specific information regarding warnings, precautions, contraindications, adverse events, and other pertinent information on the GSK investigational product or other study treatment that may impact subject eligibility is provided in the Investigator Brochure for GSK2890457, and Prescribing Information for liraglutide and metformin [VICTOZA, 2012 Prescribing Information and Medication Guide, GLUCOPHAGE, 2009 Prescribing Information].

5.1.4. Screening Exclusion Criteria for Type 2 Diabetic Subjects (Parts B and C)

1. Disease

   - History of GI disease (e.g., irritable bowel disease, chronic or current diarrhea, inflamed bowel, steatorrhoea/fat malabsorption, celiac disease, symptomatic lactose intolerance). Subjects with gastroparesis requiring treatment are excluded.
   - Current or chronic history of liver disease, or known hepatic or biliary abnormalities (with the exception of Gilbert's syndrome or asymptomatic gallstones).
   - History of serious, severe or unstable physical or psychiatric illness including depression, suicidal thoughts, schizophrenia, bipolar disorder, or generalized anxiety disorder. In addition to elicited symptoms and signs, this should include specific questions relating to known psychiatric diagnoses and medications used. C-SSRS responses indicative of depressive thoughts and/or suicidal ideation.
   - History of significant cardiovascular disease including acute myocardial infarction, stroke, hospitalization for acute coronary syndrome, heart failure within the previous 12 months.
   - History of sensitivity to environmental dusts or allergens (including hayfever), unless of mild severity in the opinion of the investigator and approved by the GSK Medical Monitor.
   - History of asthma and/or use of inhaled bronchodilators or steroids.
   - History of surgery for weight loss or gastrointestinal surgery within 3 months of screening.
   - Any documented or reported eating disorder (i.e., anorexia nervosa or bulimia nervosa) including restrictive eating or binge eating disorder.
   - Uncontrolled hypertension, as evidenced by systolic pressure>160 or diastolic pressure >90. Subjects taking anti-hypertensive medications are permitted.
   - Positive test for HIV, Hepatitis B, or Hepatitis C at Screening.
   - Significant ECG abnormalities, defined as follows:

Heart Rate	< 50 and >100bpm
PR Interval	<120 and > 220msec
QRS duration	< 70 and >120msec
QTcF	> 450 msec ms (>480msec if right bundle branch block)

- For subjects in Part C (continuing metformin), history of untreated pernicious anemia or who have laboratory parameters suggestive of subclinical megaloblastic anemia (e.g., increased MCV with low RBC count and/or Hb level).
- Current or relevant previous significant medical disorder that may require treatment or make the subject unlikely to fully complete the study, or any condition that, in the opinion of the investigator, presents undue risk from the study medication or procedures.

2. Hypoglycemia unawareness
    - T2D subjects are excluded if, in the opinion of the investigator, they have significant hypoglycaemia unawareness (for example, no symptoms of hypoglycaemia when the blood glucose level is <60mg/dL).
3. Infection
    - Presence of or symptoms of an active infection. If the subject presents with an active infection on Day -2 the visit may be rescheduled once the signs/symptoms have been absent for at least 5 days and/or antibiotic therapy, if applicable, has ceased for at least 4 weeks.
4. Thyroid Disease
    - Uncorrected Thyroid Dysfunction: Fasting plasma thyroid stimulating hormone (TSH) outside of the normal range, as determined at the screening visit.
    - Subjects on stable thyroid replacement therapy and with TSH in the normal range are eligible if approved by the GSK Medical Monitor.
    - Unevaluated thyroid nodule or goiter at Screening for Part B. (also see Inclusion Criterion #8 in Section 5.1.3)
5. Pancreatitis
    - History of chronic or acute pancreatitis, for Part B (liraglutide cohort).
    - For Part B (liraglutide cohort), subjects with a lipase or amylase value ≥2-fold above the upper limit of normal (ULN) at screening are excluded.
    - For Part C (metformin cohort), approval from the GSK Medical Monitor must be obtained for subjects with a past history of pancreatitis more than 12 months from the start of the Treatment Period (subjects with a history of pancreatitis within 12 months prior to the start of the Treatment Period are excluded).
6. Diet- and Exercise-Related Criteria
    - Currently dieting to lose weight including, but not limited to, participation in a program designed to alter body weight within the last 60 days.

- Dietary restrictions or behaviors that prevent compliance with consuming foods to be provided by the study site.
- Reported increased sensitivity to intestinal discomfort after consuming relatively small (e.g., <10g) amounts of any non-digestible carbohydrates and/or fibers.
- Unwilling to maintain relatively consistent exercise patterns throughout the study. In other words, subject should not be planning on initiating new exercise programs or discontinuing ongoing programs during Stabilization and Treatment Periods.
- Weight change of >2.5kg (increase or decrease) in the 3 months prior to Screening.
- Use of creatine supplements within 1 month of Screening.

7. Substance Abuse
   - Current or recent history (within one year of screening) of alcohol or other substance abuse, including abuse of prescription medications, with alcohol abuse defined as an average weekly intake of >14 drinks for males or >7 drinks for females. One drink is equivalent to 12g of alcohol: 12 ounces (360mL) of beer, 5 ounces (150mL) of wine or 1.5 ounces (45mL) of 80 proof distilled spirits.
   - A positive urine drug screen at Screening or Day-2.
8. Nicotine Use
   - Unwilling to stop smoking while in the clinic.
9. Medication
   - Unable to refrain from the use of non-prescription drugs, including vitamins, herbal and dietary supplements (including St John's Wort) within 7 days (or 14 days if the drug is a potential enzyme inducer) or 5 half-lives (whichever is longer) prior to the first dose of study medication, unless in the opinion of the Investigator and GSK Medical Monitor the medication will not interfere with the study procedures or compromise subject safety.
   - Use of any medication or dietary supplements within 14 days prior to the Run-in Period which may have an effect on weight or appetite, including but not limited to: Orlistat, benzphetamine, diethylpropion hydrochloride, phendimetrazine, phentermine, nutrition and/or OTC products marketed for appetite or weight control.
   - Use of fiber supplements within 14 days prior to the Run-in Period. If high-fiber foods are part of the subject's normal diet, these foods may continue through the trial.
10. Clinical Study/Experimental Medication Participation
    - Current participation in another clinical study or participation in a clinical study involving an investigational drug within 30 days of the screening visit.

- Exposure to more than four new chemical entities within 12 months prior to the first dosing day.
- Previous participation in this study.
- Where donation of blood or blood products pre-study and participation in the study would result in excess of 500mL blood depletion within a 56-day period.

11. Sensitivity/Allergy
    - History of sensitivity to any of the study medications, or components thereof or a history of drug or other allergy that, in the opinion of the investigator or GSK Medical Monitor, contraindicates their participation.
    - Sensitivity to heparin, if heparin is used to maintain catheter patency.
    - If the subject has food allergies, the GSK Medical Monitor should be consulted to confirm that participation in the study is appropriate.
12. Personnel
    - An employee of the sponsor or the study site or members of their immediate family.

5.2. Screen and Run-in Failures

Data for screen and run-in failures will be collected in source documentation at the site but will not be transmitted to GSK via InForm. However, some data for screen failures is captured in RAMOS.

6. DATA ANALYSIS AND STATISTICAL CONSIDERATIONS

6.1. Hypotheses and Treatment Comparisons

Part A of this study is designed to estimate the effect of GSK2890457 relative to placebo on safety and tolerability and the pharmacokinetics of metformin in healthy subjects. Parts B and C of the study are designed to estimate the effects of [GSK2890457+liraglutide] and [GSK2890457+metformin] relative to [placebo+liraglutide] and [placebo + metformin], respectively, on safety, tolerability and PD parameters. No formal hypotheses will be tested. Descriptive statistics will be used to assess safety, tolerability, PD and biomarker objectives. An estimation approach will be used to address the primary PD objectives, where point estimates and corresponding confidence intervals will be constructed as appropriate.

In addition, an exploratory approach using Bayesian methods will be used for Parts B and C to estimate the probability of achieving target effect sizes for % change from baseline body weight and change from baseline weighted mean glucose in subsequent studies.

6.2. Sample Size Considerations

6.2.1. Sample Size Assumptions

No formal power/sample size calculations were performed. The planned sample size for Part A in healthy subjects is based on feasibility. For Parts B and C in T2D, the planned sample size is also based on feasibility. However, the following information is provided to give some context to the sample sizes of 9 and 6 in each of the GSK2890457 and placebo treated groups, respectively. Assuming that change from baseline 24h weighted mean glucose is normally distributed with a standard deviation of 20 mg/dl [GlaxoSmithKline Document Number 2011N113387_00, SGA112534], the width of the 95% confidence interval for the difference means will be 37.4 mg/dl. In other words, the lower and upper bounds of the confidence interval will be determined by the difference in means ± 18.7mg/dl.

6.2.2. Sample Size Sensitivity

Sample size sensitivity was not performed.

6.2.3. Sample Size Re-estimation

Sample size re-estimation is not planned for this study.

6.3. Data Analysis Considerations

Parts A, B and C will be analyzed separately.

6.3.1. Interim Analysis

There will be no formal statistical interim analysis. However, available safety and PD data will be reviewed by the GSK study team on an ongoing basis throughout the study. GSK staff will be unblinded.

Preliminary safety results may be reported prior to database freeze for the purposes of safety review by GSK, the study investigators, and where required by regulatory bodies. In addition, other selected data may be unblinded and reported prior to database freeze for internal decision making.

6.3.2. Final Analyses

Final analyses will be performed after all subjects have completed the study and after database freeze/unblinding. Version 9.1 or higher of the SAS system will be used to analyze the data as well as to generate tables, figures, and listings. Complete details will be documented in the Reporting and Analysis Plan (RAP).

6.3.2.1. Safety Analyses

Safety data will be presented in tabular and/or graphical format and summarized descriptively according to GSK's Integrated Data Standards Library (IDSL) standards.

Graphical techniques will be used to assess shifts in responses to the GSRS.

6.3.2.2. Pharmacokinetic Analyses

Pharmacokinetic analysis will be the responsibility of the Clinical Pharmacokinetics Modeling & Simulation department within GlaxoSmithKline. Plasma liraglutide and metformin concentration-time data will be analyzed by non-compartmental methods with WinNonlin 5.2 or higher. Calculations will be based on the actual sampling times recorded during the study. From the plasma concentration-time data, the following pharmacokinetic parameters will be determined, as data permit: maximum observed plasma concentration (Cmax), time to Cmax (tmax), area under the plasma concentration-time curve at steady state [AUC(0-10) for immediate release metformin) and AUC(0-24 for metformin sustained release formulations and liraglutide)] for Days-1 (Day 1 for Part A)and Day 42. The PK parameters (AUCss and Cmax) will be dose normalized for statistical comparisons.

Pharmacokinetic data will be presented in graphical and/or tabular form and will be summarized descriptively. All pharmacokinetic data will be stored in the Archives, GlaxoSmithKline Pharmaceuticals, R&D.

Statistical analyses of the pharmacokinetic parameter data will be the responsibility of Clinical Statistics, GlaxoSmithKline.

There will be 3 separate PK parameter groups for statistical comparisons: (i) metformin immediate release formulations alone and with GSK2890457, (ii) metformin sustained release alone and with GSK2890457, and (iii) liraglutide alone and with GSK2890457.

Dose normalized AUCs and Cmax of liraglutide or metformin will be separately analyzed following $\log_e$-transformation using a mixed effects model with a fixed effect term for day. Subject will be treated as a random effect in the model. Point estimates and their associated 90% confidence intervals will be constructed for the differences, Day 42(co-administration) - Day 1(metformin alone in Part A) or –Day -1(liraglutide alone in Part B or metformin alone in Part C) to assess any potential interaction effects due to co-administration of liraglutide or metformin with GSK2890457. In Part A, the point estimates and their associated 90% confidence intervals will then be back-transformed to provide point estimates and 90% confidence intervals for the ratios, [Day 42(co-administration) to Day 1 (metformin alone)]. In Part B, the point estimates and their associated 90% confidence intervals will then be back-transformed to provide point estimates and 90% confidence intervals for the ratios, [Day 42(co-administration) to Day -1 (liraglutide alone)]. In Part C, the metformin groups will be separated by immediate release vs. sustained release drug products.

If data permit, GSK2890457- derived components will be monitored in Parts B and C and on Days -1 and 42. Preliminary structural information on potential human metabolites of GSK2890457 in plasma and urine following repeat dosing can identify metabolite(s) with significant circulating exposures. Drug related components of GSK2890457 may represent more stable marker(s) of GSK2890457 dosing in subjects with T2D. If performed, this work will be completed under a separate protocol and results will be reported separately.

For Part A, metformin 500mg IR, the following pharmacokinetic parameters will be calculated: AUC(0-10), (AUC(0-infinity,if data permit), Cmax, tmax and t1/2 (if data permit) and %AUC extrapolated (if data permit). The statistical analysis will be similar to that for GSK2890457.

6.3.2.3. Pharmacodynamic Analyses

Descriptive summaries of observed values and of change from baseline will be produced for all PD endpoints. Graphical displays will be used to examine both individual and treatment group responses to treatment.

Body Weight

Change from baseline and % change from baseline body weight will be analyzed using appropriate repeated measures analysis of covariance models with effects for treatment, day, treatment x day, and baseline weight. Differences in least squares means between the GSK2890457 treated groups and placebo will be calculated – i.e.; [liraglutide + GSK2890457] – [liraglutide + placebo] and [metformin + GSK2890457] – [metformin + placebo], along with associated 95% confidence intervals. In addition, the rate of weight change (slope) will be compared between the GSK2890457 treated subjects and the placebo treated subjects separately for the liraglutide and metformin cohorts. A mixed effects model will be fitted with random slope. Complete details will be provided in the RAP.

Glycemic Endpoints

Repeated measures analyses similar to those described above for body weight may be done for fasted glucose measures with inclusion of a term for baseline x time.

Change from baseline 24h weighted mean glucose will be analyzed using an ANCOVA model with effects for treatment and baseline covariate. Differences in least squares means between the GSK2890457 treated groups and placebo will be reported – i.e.; [liraglutide + GSK2890457] – [liraglutide + placebo] and [metformin + GSK2890457] – [metformin + placebo], along with associated 95% confidence intervals.

Similar analyses may be done for other glycemic endpoints including glucose and insulin weighted means over 4h, fasted insulin, HOMA-IR, Matsuda Index and HbA1c.

Model and distributional assumptions underlying each analysis will be assessed by visual inspection of residual plots. Parameters will be log-transformed prior to analysis if necessary to meet assumptions. If assumptions are still not met after log-transformation alternative methods of analysis will be considered.

Other Pharmacodynamic Endpoints

The Hunger Questionnaire (Appendix 2: Hunger Questionnaire) may be analyzed using repeated measures analyses similar to those planned for body weight.

Changes in waist circumference and DEXA estimates of lean and fat mass may be analyzed using ANCOVA models similar to those described for the glycemic endpoints.

6.3.2.4. Biomarker Analyses

Descriptive summaries of observed values and of change from baseline will be produced for all biomarkers (leptin, lipids, CRP, liver enzymes and NAFLD score). The NAFLD fibrosis score will be derived according to [Angulo, 2007].

Fecal microbiome analysis will result in descriptions of changes in fecal bacterial species from baseline to end-of-treatment. Graphical displays will be used to examine both individual and treatment group responses to treatment.

Change from baseline may be analyzed using an ANCOVA model with effects for treatment and baseline covariate. Differences in least squares means between the GSK2890457 treated groups and placebo will be reported – i.e.; [liraglutide + GSK2890457] – [liraglutide + placebo] and [metformin + GSK2890457] – [metformin + placebo], along with associated 95% confidence intervals.

6.3.2.5. Exploratory Analyses

Deuterated Creatine – Skeletal Muscle Mass

Skeletal muscle mass (kg) will be estimated from the enrichment of deuterated creatinine in urine if the deuterated creatine has been dosed.

Change from baseline may be analyzed using an ANCOVA model with effects for treatment and baseline covariate. Differences in least squares means between the GSK2890457 treated groups and placebo will be reported – i.e.; [liraglutide + GSK2890457] – [liraglutide + placebo] and [metformin + GSK2890457] – [metformin + placebo], along with associated 95% confidence intervals.

DEXA appendicular lean mass will be compared to the deuterated creatine method for muscle mass using Bland-Altman methodology [Bland, 1986] if the deuterated creatine has been dosed.

6.3.2.6. Pharmacodynamic/Biomarker Analyses (Part A will include a subset of these)

- Food Intake:
    - Correlation between Background information and Food Frequency Questionnaire responses collected at baseline and efficacy.
- Body Composition:
    - Leptin
    - DEXA measurement of fat and skeletal muscle mass on Day 41 as compared to Day -2.
    - Waist circumference measurements.

- Gut Pharmacodynamics:
  - Changes in bacterial species using fecal microbiome analysis between baseline and the end of the Treatment Period, and correlation of baseline data and efficacy.
- Cardiovascular markers:
  - Blood pressure
  - Lipids (HDLc, LDLc, triglycerides)
- Inflammatory markers:
  - CRP
- Markers of liver status:
  - ALT, AST
  - NAFLD Fibrosis Score

Graphical techniques will be used to investigate the relationship between response (weight, glycemic endpoints) and potential predictive variables including but not limited to baseline measures of weight, HbA1c, fecal microbiome, background information (e.g., recent antibiotic use, birth method, bottle or breast fed) and responses to the Block Food Frequency Questionnaire (provided by GSK).

6.3.2.7. Novel and Other Biomarker Analyses in Parts B and C

There may be evaluation of some, all, or none of the following biomarkers depending on changes observed in weight and glucose-related endpoints. Evaluation may be made for all subjects or for selected subjects:

- Metabolomic analysis of plasma
- Lipopolysaccharide concentration in blood
- Circulating gut hormones concentrations (GLP-1, PYY, and/or other gut peptide hormones)
- Markers of inflammation in blood (for example TNFα, IL-6 concentrations)
- Markers of insulin sensitivity (for example adiponectin).

Exploratory analyses relating to biomarkers of obesity, T2D and/or related metabolic diseases and/or inflammatory diseases or conditions, and other biomarkers of GSK2890457 PD or safety may be performed using (i) small molecular weight metabolites, (ii) blood polypeptide analytes, and, (iii) novel biomarkers derived from peptidomic, lipidomic and metabolomic analysis of blood and/or urine.

Additional exploratory analyses may be performed to further characterize novel biomarkers.

The results of these biomarker investigations may be reported separately from the main clinical study.

7. STUDY ASSESSMENTS AND PROCEDURES

This section lists the parameters of each planned study assessment. The exact timing of each assessment is listed in the Time and Events Tables (Section 4.7). Detailed procedures for obtaining each assessment are provided in Section 7. Whenever vital signs, 12-lead ECGs and blood draws are scheduled for the same nominal time, the assessments should occur in the following order: 12-lead ECG, vital signs, blood draws. The timing of the assessments should allow the blood draw to occur at the exact nominal time.

The timing and number of planned study assessments, including safety, pharmacokinetic, PD/biomarker or other assessments may be altered during the course of the study based on newly available data (e.g., to obtain data closer to the time of peak plasma concentrations) to ensure appropriate monitoring. The change in timing or addition of time points for any planned study assessments must be approved and documented by GSK, but this will not constitute a protocol amendment. The IRB/IEC will be informed of any safety issues that require alteration of the safety monitoring scheme. No more than 500mL of blood will be collected over the duration of the study, including any extra assessments that may be required.

7.1. Demographic/Medical History Assessments

The following demographic parameters will be captured: year of birth, gender, race and ethnicity.

Medical/medication/alcohol history will be assessed as related to the eligibility criteria listed in Section 5.1. In Part B, special attention will be paid to obtaining a detailed psychiatric history to exclude subject with serious, severe or unstable physical or psychiatric illness including depression, suicidal thoughts, schizophrenia, bipolar disorder, or generalized anxiety disorder. This should include specific questions relating to any known psychiatric disorders that the subject has and medications used.

In Part B and C, cardiovascular and gastrointestinal medical history/risk factors will also be assessed at baseline. Background questions (Appendix 4: Background and End-of-Study Questions) and the Block Brief Food Frequency Questionnaire (a validated questionnaire obtained from and analyzed by NutritionQuest), will be administered at the beginning of the Treatment Period. Baseline Vitamin-D levels (25-hydroxy-Vitamin D) will be obtained. This information is being obtained to permit possible correlation of subject background with efficacy of GSK2890457. End-of-study questions (Appendix 4: Background and End-of-Study Questions) will be used to obtain subject feedback at the end of the Treatment Period.

Female subjects whose post-menopausal status is in doubt should have FSH and estradiol tested to confirm that they are not of child-bearing potential.

HRT must be stopped temporarily to confirm post-menopausal status.

7.2. Safety

Planned timepoints for all safety assessments are listed in the Time and Events Table (Section 4.7). Additional time points for safety tests such as vital signs and laboratory safety tests may be added during the course of the study based on newly available data to ensure appropriate safety monitoring.

For the purpose of assessing renal function in this study, GFR is considered to be equivalent to Creatinine clearance.

7.3. Physical Exams

A complete physical examination will include assessments of the head, eyes, ears, nose, throat, skin, thyroid (especially in Part B), neurological, lungs, cardiovascular, abdomen (liver and spleen), lymph nodes and extremities. Height and weight will also be measured and recorded.

Brief physical examinations will include assessments of the skin, lungs, cardiovascular system, abdomen (liver and spleen) and brief neurological review.

The exam will be performed by a qualified licensed, medical professional (i.e., physician, physician assistant, or nurse practitioner) and include a thorough review of all body systems.

For females of NCBP, best efforts should be made to obtain records to document bilateral oophorectomy (Parts A, B, and C) or hysterectomy or tubal ligation (Part A). When these are not available, self-report is permitted provided there is clear source documentation to this effect and an investigator concurs with the self-report.

7.3.1. Vital Signs

Supine or semi-supine vital sign measurements will include systolic and diastolic blood pressure and pulse rate. Vitals should be taken as close to the nominal time as possible. Vital sign assessments should be performed after resting in a supine or semi-supine position for at least 10 minutes. Extreme changes in room temperature should be avoided (subjects should be comfortable at all times in light clothing).

7.3.2. Electrocardiogram (ECG)

Unless otherwise stated, single 12-lead ECGs will be obtained at each timepoint during the study using an ECG machine that automatically calculates the heart rate and measures PR, QRS, QT, and QTcF intervals. Refer to Section 4.6.1.2 for QTcF withdrawal criteria and additional QTcF readings that may be necessary.

Single 12-lead ECGs will be obtained at each timepoint after subjects have been resting in a supine position for at least 10 minutes. Extreme changes in room temperature should be avoided (subjects should be comfortable at all times in light clothing).

ECGs should be performed within 5 minutes of the nominal time.

Whenever 12-lead ECG measurements are scheduled at the same nominal time as vital signs or blood draws, the ECG data must be obtained first, followed by vital signs and then blood draws. Additional ECGs may be taken at the discretion of the investigator as needed based on symptoms or ECG findings.

If an ECG is found to be abnormal, 2 more ECGs should be obtained over a brief period of time (~5 minutes apart) if the Investigator considers that the abnormality may be transient. If the ECG is not considered to be transient, then only 1 ECG is required.

Note: The early morning ECGs and vitals may be collected up to 60 minutes prior to breakfast.

7.3.3. Weight

Body weight in kilograms (kg) will be assessed; the subject will wear lightweight indoor clothing and remove shoes for the assessment when in the unit. When weighing at home, subjects should be nude.

7.3.4. Waist Circumference

Waist circumference will be measured with the subject in the standing position. The waist area must be free of clothing and will be measured in centimeters using a tape measure held at the level of the top of the iliac crests and parallel to the floor. Assessments will be completed as per the schedule outlined in the Time and Events Table in the protocol. Waist circumference may be rounded to the nearest half centimeter.

7.3.5. Clinical Laboratory Assessments

Hematology, clinical chemistry, urinalysis and additional parameters to be tested are listed below:

Hematology

Platelet Count	*RBC Indices*:	*Automated WBC Differential:*
RBC Count	MCV	Neutrophils
WBC Count (absolute)	MCH	Lymphocytes
Reticulocyte Count	MCHC	Monocytes
Hemoglobin		Eosinophils
Hematocrit		Basophils

Clinical Chemistry

BUN	Potassium	AST (SGOT)	Total and direct bilirubin
Creatinine	Chloride	ALT (SGPT)	Uric Acid
Glucose, fasting	Total CO_2	GGT	Albumin
Sodium	Calcium	Alkaline phosphatase	Total Protein
Magnesium	Lipase (Part B only)	Amylase (Part B only)	Phosphate
T3/T4/TSH (Treatment Period of Parts B and C only)	25 Hydroxy Vitamin D at Day -1, Parts B and C only	High sensitivity CRP (Treatment Period of Parts B and C only)	Leptin (Treatment Period of Parts B and C only)

Clinical Chemistry

HDL cholesterol (Treatment Period of Parts B and C only)	LDL cholesterol (Treatment Period of Parts B and C only)	Fasting triglycerides (Treatment Period of Parts B and C only)	Insulin, fasting
HbA1c (Parts B and C only)			
Alcohol and drug screen (to include at minimum: amphetamines, barbiturates, cocaine, opiates, cannabinoids and benzodiazepines)			

Routine Urinalysis

Specific gravity
pH, glucose, protein, blood and ketones by dipstick
Microscopic examination (if blood or protein is abnormal)
Urine beta-HCG (women) as indicated in Section 4.7

Additional tests conducted at Screening

HIV
Hepatitis B (HBsAg)
Hepatitis C (Hep C antibody -- if second generation Hepatitis C antibody positive, a hepatitis C antibody Chiron RIBA immunoblot assay (or other third generation immunoassay) should be reflexively performed **on the same sample** to confirm the result)
Urine albumin and creatinine (Parts B and C)
Calcitonin (Part B only)
C-peptide (Parts B and C)
FSH and estradiol (as needed in women of non-child bearing potential only)

7.3.6. Re-Screening

Subjects who have fulfilled the Inclusion/Exclusion criteria at Screening, but do not start the Treatment period in Part A or the Stabilization period in Parts B or C within 28 days, may be re-screened within 2 months of the initial screening visit using the following abbreviated procedures:

- Medical history updated from the last Screening visit
- Concomitant medications updated from the last Screening visit
- Vital signs

- Hematology, clinical chemistry and urinalysis to be tested at Re-Screening are listed below:

Hematology

Platelet Count	*RBC Indices*:	*Automated WBC Differential*:
RBC Count	MCV	Neutrophils
WBC Count (absolute)	MCH	Lymphocytes
Reticulocyte Count	MCHC	Monocytes
Hemoglobin		Eosinophils
Hematocrit		Basophils

Clinical Chemistry

BUN	Potassium	AST (SGOT)	Total and direct bilirubin
Creatinine	Chloride	ALT (SGPT)	Uric Acid
Glucose, fasting	Total CO_2	GGT	Albumin
Sodium	Calcium	Alkaline phosphatase	Total Protein
Magnesium	Lipase (Part B only)	Amylase (Part B only)	Phosphate
Alcohol and drug screen (to include at minimum: amphetamines, barbiturates, cocaine, opiates, cannabinoids and benzodiazepines)			

Routine Urinalysis

pH, glucose, protein, blood and ketones by dipstick
Microscopic examination (if blood or protein is abnormal)

7.3.7. GI Symptom Rating Scale (GSRS)

A GI symptom rating scale (Appendix 3: GI Symptom Rating Scale (GSRS) Questionnaire Appendix 3: GI Symptom Rating Scale (GSRS) Questionnaire) will be administered at the start of the Stabilization Period, and at the times specified in the Time and Events Tables (Section 4.7).

7.4. Pregnancy

Females of childbearing potential are not eligible to participate so pregnancies are not expected to occur in this study. In the unlikely event that a pregnancy should occur in a female subject, the investigator will attempt to collect pregnancy information following the guidelines outlined in Section 7.4.2.

The investigator will attempt to collect pregnancy information on any female partner of a male study subject who becomes pregnant while participating in this study. When a pregnancy occurs, the guidelines outlined in Section 7.4.2 are to be followed.

7.4.1. Time Period for Collecting Pregnancy Information

All pregnancies in female subjects and/or female partners of male subjects will be collected after the start of dosing and until the final follow-up visit.

7.4.2. Action to be Taken if Pregnancy Occurs

The Investigator will collect pregnancy information on any female subject and/or female partner of a male subject, who becomes pregnant while participating in this study. The Investigator will record pregnancy information on the appropriate form and submit it to GSK within 2 weeks of learning of a subject's pregnancy. The female subject or female partner of a male subject will also be followed to determine the outcome of the pregnancy. Information on the status of the mother and child will be forwarded to GSK. Generally, follow-up will be no longer than 6 to 8 weeks following the estimated delivery date. Any premature termination of the pregnancy will be reported.

While pregnancy itself is not considered to be an AE or SAE, any pregnancy complication or elective termination of a pregnancy in a female subject or the female partner of a male subject for medical reasons will be recorded as an AE or SAE.

A spontaneous abortion is always considered to be an SAE and will be reported as such. Furthermore, any SAE occurring as a result of a post-study pregnancy and is considered reasonably related to the study treatment by the Investigator, will be reported to GSK as described in Section 12. While the Investigator is not obligated to actively seek this information in former female study participants or female partners of male participants, he or she may learn of an SAE through spontaneous reporting.

Any female subject who becomes pregnant while participating will discontinue study medication.

7.5. Pharmacokinetics

On PK sampling days, subjects should be resting quietly, in a supine or semisupine position for 5 minutes prior to PK timepoints

7.5.1. Blood Sample Collection

Blood samples for pharmacokinetic analysis of GSK2890457 + metabolites, liraglutide and metformin will be collected at the time points indicated in Section 4.7. The actual date and time of each blood sample collection will be recorded. The timing of PK samples may be altered and/or PK samples may be obtained at additional time points, based on emerging data, to ensure thorough PK monitoring.

7.5.1.1. Blood Samples for Metformin or Liraglutide PK

Collect blood into one designated tube (supplied by GSK, 2mL EDTA evacuated tube, or alternative tube as required by the analytical lab) for metformin or liraglutide, according to the scheduled blood draws for each analyte. Refer to Section 4.7.5 and Section 4.7.6 for sampling times. Immediately after collection, gently invert (DO NOT SHAKE) the evacuated blood collection tube 8-10 times to mix the EDTA anticoagulant with the whole blood and place the sample(s) on ice or in a refrigerator. Within 1 hour of sample collection, separate the plasma by refrigerated (4° C) centrifugation at 1,500 to 2,000 x g for a minimum of 10 minutes. Transfer resulting plasma into a 1.8mL cryotube (NUNC). Store in freezer at approximately -20ºC.

7.5.1.2. Blood Samples for GSK2890457 PK

Collect blood into one 2mL Potassium Oxalate/Sodium Fluoride evacuated tube for GSK2890457 + metabolites, according to the scheduled blood draws for each analyte. Refer to Section 4.7.6 for sampling times. *Process samples as quickly as possible. Samples must be protected from light.* Immediately after collection, gently invert (DO NOT SHAKE) the evacuated blood collection tube 8-10 times to mix the Potassium Oxalate/Sodium Fluoride anticoagulant with the whole blood. Immediately after processing, separate the plasma by refrigerated (4° C) centrifugation at 1,500 to 2,000 x g for a minimum of 10 minutes.

Transfer exactly 250µL plasma into an amber 1.4mL Matrix tube containing exactly 250µL 50mM citrate buffer. Transfer a second aliquot of exactly 500µL plasma into an 2mL amber storage tube containing exactly 500µL 50mM citrate buffer. Immediately place both samples in freezer at approximately -80ºC.

Details of PK blood sample processing, and storage procedures, as well as instructions for preparing the buffer solution and shipping procedures are detailed in the SPM.

7.5.2. Urine Sample Collection

Urine samples for pharmacokinetic analysis of GSK2890457 + metabolites will be collected at the timepoints listed in Section 4.7. The timing of urine samples may be altered and/or samples may be obtained at additional time points, based on emerging data, to ensure thorough PK monitoring. Details of PK urine sample processing, storage procedures and shipping procedures are detailed in the SPM.

On Days -1 and 42, the first urine of the day will be used for urinalysis and any remaining urine from the first void will be discarded. Collect all subsequent urine samples in an amber container for each interval. The first urine sample voided on Days 1 and 43 will be added to the collection for the 24-hour interval. At the end of each collection interval, transfer 100mL into a 150mL amber bottle and immediately store at -80ºC.

7.5.3. Sample Analysis

Plasma/serum and urine analysis will be performed under the management of Worldwide Bioanalysis, DMPK, GlaxoSmithKline. Concentrations of GSK2980457, liraglutide and metformin will be determined in plasma and/or urine samples using the currently approved analytical methodology.

Pharmacokinetic analyses may include quantitation of the ratio of deuterium-labeled creatinine to total creatinine by LC-MS, total creatine and creatinine by LC-MS.

Urine analysis will be performed under the management of Worldwide Bioanalysis, DMPK, GlaxoSmithKline.

Raw data will be stored in the GLP Archives, GlaxoSmithKline. Once the plasma has been analyzed, any remaining plasma may be analyzed qualitatively for other circulating

metabolites and the results reported under a separate DMPK protocol. The urine samples may be analyzed for compound-related metabolites and the results will be reported under a separate DMPK protocol.

7.6. Biomarker(s)/Pharmacodynamic Markers

7.6.1. Confirmed Pharmacodynamic Markers (Parts B and C)

7.6.1.1. Glucose and Insulin

Glucose and insulin samples will be collected at the times indicated in Section 4.7.

7.6.1.2. Other Biomarkers

Measurements of fasting plasma leptin, high sensitivity CRP, HDLc, LDLc, triglycerides, and liver enzymes may be performed at the timepoints indicated in Section 4.7.3.

DEXA analysis of fat and lean muscle mass will be performed at the timepoints indicated in Section 4.7.3.

7.6.1.3. Telemonitoring of Weight and CBG

Subjects will be provided with a specific scale, glucometer and strips, and a telemonitoring unit that will transmit weight and glucose data to a central database. Each Investigator will be able to review data in real time for subjects enrolled at his site. Subjects will be asked to weigh themselves unclothed each morning before breakfast, and to collect a fasting glucose sample and a pre-dinner glucose sample, which will need to be downloaded to the telemonitoring device at least every other day.

7.6.1.4. Fecal Microbiome Analysis

Fecal-microbiome analysis will be performed at the timepoints indicated in Section 4.7.4.

Collect 3 spoons of each stool sample and transfer into a pre-labelled Stool Collection Tube provided by GSK. After collection, immediately place tubes in freezer and store frozen at -20°C prior to shipment.

Microbial genomic DNA will be isolated from the fecal samples and 16s rDNA and metagenomic PCRs may be performed from them.

16s rDNA and possibly metagenomic sequencing may provide an understanding of microbiome changes that have occurred in subjects receiving GSK2890457.

7.6.2. Exploratory Biomarkers (Parts B and C)

7.6.2.1. Other Exploratory Biomarkers

With the subject’s consent, blood and/or urine sample(s) will be collected during this study and may be used for the purposes of measuring novel biomarkers to identify factors that may influence T2D, obesity and/or medically related conditions and/or inflammatory

diseases or conditions, as well as the biological and clinical responses to GSK2890457. If relevant, this approach will be extended to include the identification of biomarkers associated with adverse events.

All samples will be retained for a maximum of 15 years after the last subject completes the trial.

7.6.2.2. Metabolomic Research

Plasma metabolome studies may be performed by nuclear magnetic resonance spectroscopy (NMR), mass spectrometry (MS, LC-MS, GC-MS and/or FT/MS) and equivalent methods. This may include analysis of identified or uncharacterized metabolites and lipids that are known to be or emerge in the future as being important in the pathogenesis of T2D, obesity and/or related metabolic disorders and/or inflammatory diseases or conditions, and/or the subject’s response to GSK2890457 or adverse events.

7.6.2.3. Proteome/Peptide Research

Plasma proteome studies may be performed by 2-D gel separation, and/or peptide mass mapping, or an alternative equivalent procedure. Proprietary algorithms and standard statistical techniques, such as ANOVA and ANCOVA, may be used to identify individual proteins exhibiting statistically acceptable changes in their levels between samples, and between groups of samples. These differentially expressed proteins will be identified by mass spectrometry or equivalent technology. This will enable the evaluation of changes in proteome profiles that may correlate with biological response relating to type 2 diabetes mellitus, obesity and the metabolic syndrome and/or related medical conditions and/or inflammatory diseases or conditions, and/or the biological and clinical responses to GSK2890457 or adverse events.

The same samples may also be used to confirm findings by application of alternative technologies.

7.6.3. Blood Sample Collection for Biomarkers

7.6.3.1. Blood Sample Collection for Glucose and Insulin

Collect each serial whole blood PD sample as close as possible to the planned time relative to dosing detailed in Section 4.7. Collect whole blood (2mL) sample into a properly labeled EDTA evacuated blood collection tube (supplied by GSK). Record the date and exact time that each sample is collected in the CRF.

Immediately after collection, gently invert (DO NOT SHAKE) the evacuated blood collection tube 8-10 times to mix the EDTA anticoagulant with the whole blood and place the sample(s) on crushed ice or in a refrigerator. Within 1 hour of sample collection, separate the plasma by refrigerated (4°C) centrifugation at 1,500 to 2,000 x g for a minimum of 10 minutes. Transfer resulting plasma into 1.8mL cryotubes (NUNC); do not overfill. Store in freezer at approximately -80°C.

7.6.3.2. Blood Sample Collection for Other Biomarkers

Collect each serial whole blood PD sample as close as possible to the planned time relative to dosing detailed in Section 4.7. Collect whole blood (5mL) sample into a properly labeled EDTA evacuated blood collection tube (supplied by GSK). Record the date and exact time that each sample is collected in the CRF.

Immediately after collection, gently invert (DO NOT SHAKE) the evacuated blood collection tube 8-10 times to mix the EDTA anticoagulant with the whole blood and place the sample(s) on crushed ice or in a refrigerator. Within 1 hour of sample collection, separate the plasma by refrigerated (4° C) centrifugation at 1,500 to 2,000 x g for a minimum of 10 minutes. Transfer resulting plasma into 1.8mL cryotubes (NUNC); do not overfill. Store in freezer at approximately -80ºC.

7.6.3.3. Blood Sample Collection for Fasting Exploratory Biomarkers

Collect each serial plasma exploratory biomarker sample as close as possible to the planned time relative to dosing detailed in Section 4.7. Collect sample (10mL) into a properly labeled EDTA evacuated blood collection tube (supplied by GSK). Record the date and exact time that each sample is collected in the CRF.

Immediately after collection, gently invert (DO NOT SHAKE) the evacuated blood collection tube 8-10 times to mix the EDTA anticoagulant with the whole blood and place the sample(s) on crushed ice or in a refrigerator. Within 1 hour of sample collection, separate the plasma by refrigerated (4° C) centrifugation at 1,500 to 2,000 x g for a minimum of 10 minutes. Transfer resulting plasma into 1.8mL cryotubes (NUNC); do not overfill. Store in freezer at approximately -80ºC.

7.6.4. Deuterated Creatine Method for Measuring Total Skeletal Muscle Mass

If available prior to the Treatment period, a capsule containing 30mg of deuterated creatine will be taken with ~200mL of water 2h after lunch in the clinic on Days -2 and 41 at the timepoints listed in Section 4.7.

On Days 1 and 43, a fasting morning urine sample, but not the first void, will be collected within four hours of waking, approximately 38 – 46 hrs after the deuterated-creatine dose (if the deuterated creatine has been dosed). Urine collection will be in 2 tubes: 1 tube (1.4mL Matrix TrakMate) for a 500uL urine sample, labelled for deuterated-creatinine/unlabelled creatinine, and a second tube (1.4mL Matrix TrakMate) for a 500uL urine sample for assay of unlabeled creatine.

In addition, on Day 41 prior to second dose of deuterated-creatine a urine sample will be collected for assessment of background deuterated-creatinine if deuterated creatine has been dosed.

The time of day for dosing and time of day for urine collections will be the same at baseline and end of the 6-week Treatment Period.

If deuterated creatine has been dosed, urine samples for pharmacokinetic analysis of deuterium labelled creatinine, total creatine, and total creatinine, will be collected at the timepoints listed in Section 4.7. The timing of urine samples may be altered and/or samples may be obtained at additional time points to ensure thorough PK monitoring.

7.6.5. Hunger Questionnaire

GSK2890457 elevates incretins and PYY in animal models of obesity and these may affect food intake. For this reason, this study includes assessments of eating behaviors.

The Hunger Questionnaire (Appendix 2: Hunger Questionnaire) is designed to assess changes in eating behaviors as a result of study treatment. The Hunger Questionnaire is a self-administered questionnaire that will be used as an exploratory measure to understand patient-reported feelings of hunger and satiety.

The Hunger Questionnaire comprises 4 items to assess hunger, craving, and fullness. Patients rate each item on a scale from 1 to 10. This instrument has not undergone psychometric evaluation.

The Hunger Questionnaire will be administered on Days -1 and 42 at the timepoints indicated in Section 4.7.6.

8. LIFESTYLE AND/OR DIETARY RESTRICTIONS

8.1. Contraception Requirements

8.1.1. Female Subjects

Only females of non-childbearing potential are eligible to participate in this study.

8.2. Meals and Dietary Restrictions

8.2.1. Standard Test Meals

Part A

To assess the PK of metformin, standardized meals will be provided on Days 1 and 42 and will consistently contain approximately 60% carbohydrate, 18% fat, and 22% protein. The breakfast meal should contain approximately 400-500kcal. Within these limits, some variation of components is allowed based on subject preference, but the choices must be the same on Days 1 and 42. Subjects should be consulted to ensure that the meals provided are not excessive.

In Part A, subjects will be asked to report the feasibility of consuming GSK2890457 and then breakfast (Days 1 and Day 42) and dinner meals (Days 1 and 42). The composition of the standardized meals in Parts B and C may be modified based on emergent data from Part A.

Parts B and C

Standardized meals will be provided on Days -2 and 41 (dinner) and Days -1 and 42 (all three meals) and will consistently contain approximately 60% carbohydrate, 18% fat, and 22% protein. Within these limits, some variation of components is allowed based on subject preference, but the choices must be the same on Days -2 and 41 and the same on Days -1 and 42. Subjects should be consulted to ensure that the meals provided are not excessive.

On Days -1 and 42 in Part B and C, the breakfast meal will constitute a ‘meal tolerance test’ and should contain approximately 400-500kcal (unless modified based on emergent data from Part A). The breakfast meal composition will be the same on Days -1 and 42.

If a standardized meal cannot be eaten completely, the percentage of the meal eaten should be recorded.

In Parts B and C, all standardized meals should *not* contain berry fruits

8.2.2. Caffeine, Alcohol, and Tobacco

- Subjects will abstain from ingesting caffeine- or xanthine-containing products (e.g. coffee, tea, cola drinks, and chocolate) for 24 hours prior to admission into the clinical unit until collection of the final safety, pharmacokinetic and or PD sample during each inpatient session.
- Subjects will abstain from alcohol for 24 hours prior to admission into the clinical unit until collection of the final safety, pharmacokinetic and or PD sample during each session. Subjects taking metformin should be cautioned against excessive alcohol intake (either acute or chronic) as this may potentiate the effect of metformin on lactate metabolism.
- Subjects who use tobacco products will be instructed that use of nicotine-containing products (including nicotine patches) will not be permitted while they are in the Clinical Unit.

8.3. Activity

Subjects will abstain from strenuous exercise for 48 hours prior to each blood collection for clinical laboratory tests. Subjects may participate in light recreational activities during studies (e.g., watch television, read).

9. CONCOMITANT MEDICATIONS AND NON-DRUG THERAPIES

9.1. Part A – Healthy Subjects

9.1.1. Permitted Medications

Ibuprofen, at doses of <1.2grams/day, and acetaminophen, at doses of ≤ 1.5 grams/day, are permitted for use any time during the study [James, 2009]. Other concomitant medications may be considered on a case by case basis by the GSK Medical Monitor.

Thyroid replacement to maintain the subject in a euthyroid state.

The Investigator, in consultation with the GSK Medical Monitor, may use loperamide for temporary relief of diarrhea when GSK2890457 and metformin are co-dosed.

Other medications may be permitted following consultation with the GSK Medical Monitor if they are not considered to affect subject safety or the objectives of the study.

9.1.2. Prohibited Medications

Subjects must abstain from taking prescription or non-prescription drugs , within 7 days or 5 half-lives (whichever is longer) prior to the start of the Treatment Period until completion of the follow-up visit, unless in the opinion of the Investigator and GSK Medical Monitor the medication will not interfere with subject safety and wellbeing or the study outcomes and conclusions.

- Use of dietary supplements within 14 days prior to randomization which may have an effect on weight or appetite, including but not limited to nutrition and/or OTC products marketed for appetite or weight control.
- Use of fiber supplements within 14 days prior to randomization.
- Use of any medications within 14 days before start of Treatment Period that may have the potential to interact with the effects of GSK2890457, including oral antibiotics, bile acid sequestrants, protein-pump inhibitors , H2 antagonists, probiotics, herbal and nutraceutical products intended to impact gut health and use of stomach ‘coating’ agents, e.g., Pepto-Bismol, Kaopectate.

9.1.3. Non-Drug Therapies

Subjects must abstain from taking any vitamins, herbal and dietary supplements within 7 days or 5 half-lives (whichever is longer) prior to the start of the Treatment Period until completion of the follow-up visit, unless in the opinion of the Investigator and the GSK Medical Monitor the medication will not interfere with the study objectives and conclusions.

9.2. Parts B and C – T2D Subjects

9.2.1. Permitted Medications

Ibuprofen, at doses of <1.2grams/day, and acetaminophen, at doses of ≤ 1.5 grams/day are permitted for use any time during the study [James, 2009]. Other concomitant medications may be considered on a case by case basis by the GSK Medical Monitor.

All concomitant medications taken during the study will be recorded in the CRF. Subjects using the following medications must be on stable doses during the 3 months prior to Screening:

Antihypertensives (e.g., beta blockers, ACE inhibitors, angiotensin II receptor antagonists, calcium channel blockers and thiazide diuretics). The dosage(s) of antihypertensive medications should, if medically appropriate, remain unchanged while the subject is enrolled in the study. If a dosage change is necessary, information pertaining to the change must be documented on the concomitant medication pages in the eCRF.

Thyroid hormone to maintain euthyroidism.

Lipid modifying medications (e.g., statins, Vytorin, niacin, fibrates). The dosage(s) of the lipid-modifying medications should, if medically appropriate, remain unchanged while the subject is enrolled in the study. If a dosage change is necessary, information pertaining to the change must be documented on the concomitant medication pages in the eCRF.

The use of other non-steroidal anti-inflammatory drugs (NSAIDs), e.g., aspirin, is allowed only if prescribed by a physician on a regular schedule for cardiovascular prophylaxis or chronic pain control.

In Part C, the Investigator, in consultation with the GSK Medical Monitor, may use loperamide for temporary relief of diarrhea when GSK2890457 and metformin are co-dosed.

Permitted concomitant medications should be taken as prescribed at the usual time(s), and may be taken with water prior to scheduled study clinic visits without being considered to have broken their fast.

Other medications may be permitted following consultation with the GSK Medical Monitor if they are not considered to affect subject safety or the objectives of the study.

9.2.2. Prohibited Medications

Other than the permitted medications in Section 9.2.1, subjects must abstain from taking prescription or non-prescription drugs , within 7 days or 5 half-lives (whichever is longer) prior to the start of the Treatment Period until completion of the follow-up visit, unless in the opinion of the Investigator and GSK Medical Monitor the medication will not interfere with the study.

- Use of any medication or dietary supplements within 14 days prior to the Run-in Period which may have an effect on weight or appetite, including but not limited to: Orlistat, benzphetamine, diethylpropion hydrochloride, phendimetrazine, phentermine, nutrition and/or OTC products marketed for appetite or weight control.
- Use of fiber supplements within 14 days prior to run-in.
- Use of any medications within 14 days before start of Treatment Period that may have the potential to interact with the effects of GSK2890457, including oral antibiotics (within 4 weeks prior to Treatment Period), bile acid sequestrants, protein-pump inhibitors, H2 antagonists, probiotics , herbal and nutraceutical products intended to impact gut health and use of stomach 'coating' agents e.g., Pepto-Bismol, Kaopectate.
- Use of medications that affect GI motility within 14 days before start of Treatment Period including, but not limited to metoclopramide (Reglan), Temporary use of loperamide is permitted in Part C as indicated in Section 9.2.1.
- Use of medications known to influence carbohydrate metabolism, including but not limited to systemic (non-topical) corticosteroids within 4 weeks before start of Treatment Period.
- For Part B, previous exposure to any GLP-1 mimetic or dipeptidyl peptidase-IV (DPP-IV) inhibitor unless approved by the GSK Medical Monitor.

The Investigator should record all medicines and supplements a subject has taken within one month of screening to determine eligibility and discuss eligibility with the GSK Medical Monitor if in doubt.

9.2.3. Non-Drug Therapies

Subjects must abstain from taking any vitamins, herbal and dietary supplements within 7 days or 5 half-lives (whichever is longer) prior to the start of the Treatment Period until completion of the follow-up visit, unless in the opinion of the Investigator and the GSK Medical Monitor the medication will not interfere with the study objectives and conclusions.

T2D subjects must not take creatine supplements from the Screening visit to the end of the Treatment Period.

9.3. Anti-Viral Therapies for Influenza (Parts A, B and C)

In all parts of the study, Tamiflu and RELENZA™ are permitted for the treatment of influenza, following consultation with the GSK Medical Monitor. GSK will not supply flu treatment or vaccines.

10. COMPLETION OR EARLY WITHDRAWAL OF SUBJECTS

10.1. Subject Completion

A completed subject is one who has completed all phases of the study including the follow-up visit.

The end of the study is defined as the last subject's last visit.

10.2. Subject Withdrawal Criteria

Subjects who are unable or unwilling to demonstrate 80% compliance with the telemonitoring requirements during the run-in "procedures familiarization" period will be withdrawn from the study and will not proceed to the Stabilization Period. Subjects not using the telemonitoring glucose and weight equipment appropriately will be counselled on correct use, and technical issues will be addressed, if necessary. If poor compliance continues and does not improve with counselling, this may result in withdrawal from the study, after consultation with the GSK Medical Monitor.

Refer to Section 4.6 for dose adjustment/stopping/withdrawal criteria based on safety and/or tolerability criteria.

A subject may withdraw from study treatment at any time at his/her own request, or may be withdrawn at any time at the discretion of the Investigator for safety, behavioral or administrative reasons.

10.2.1. Parts B and C Glucose Withdrawal Criteria

- Subjects clearly experiencing episodes of significant symptomatic hypoglycemia or episodes of significant biochemical hypoglycemia regardless of symptoms should be withdrawn if the episodes are not ameliorated by reducing the dose of study medication(s). Subjects may continue if the episodes of hypoglycemia are not considered clinically significant (e.g., because they are a physiological response to fasting/reduced food intake).
- An episode of severe hypoglycemia that requires Category 4 intervention (Section 4.6.1.4) occurring in 1 or more subjects will result in termination of dosing, if in the judgment of the Investigator and GSK Medical Monitor there is a low probability that modification of the dosing regimen or general supportive measures (e.g., food intake) will reduce the severity of the hypoglycemia.
- If fasting CBG/blood glucose levels are >270mg/dL on 3 consecutive days, the subject will be withdrawn and appropriate anti-diabetic therapy reinstated, unless, in the judgment of the Investigator and GSK Medical Monitor, the subject is well and there are circumstances that indicate that the high fasting CBG/blood glucose levels are temporary and would not compromise subject safety and wellbeing or study objectives and conclusions.

10.3. Subject Withdrawal Procedures

10.3.1. Subject Withdrawal from Study

Subjects who are withdrawn completely from the study should have Follow-up (Day 56) procedures performed at the time of withdrawal.

Subjects randomized to liraglutide will stop this medication and restart their usual dose of metformin, but the investigator may adjust the dose based on clinical judgment.

10.3.2. Subject Withdrawal from Study Treatment

Subjects who elect to withdraw from treatment should be encouraged to return for Follow-up (Day 56) procedures, to permit appropriate monitoring for safety.

Subjects randomized to liraglutide will stop this medication and restart their usual dose of metformin, but the Investigator may adjust the dose based on clinical judgment.

10.4. Treatment After the End of the Study

Subjects will not receive any additional treatment from GSK after completion of the study because i) only healthy volunteers are eligible for Part A, or, ii) for Parts B and C (T2D), other treatment options are available.

The Investigator is responsible for ensuring that consideration has been given to the post-study care of the patient’s medical condition, whether or not GSK is providing specific post-study treatment.

11. STUDY TREATMENT

Study treatment dosage and administration details are listed in Section 4.4.1.

11.1. Blinding

Subjects and site personnel, with the exception of specified pharmacy staff and unblinded clinical unit staff involved with instructing subjects on appropriate medication administration, will be blinded to subject treatment. Unblinded clinical site personnel will not take part in any study assessments.

GSK study staff will be unblinded.

The Investigator or treating physician may unblind a subject’s treatment assignment **only in the case of an emergency**, when knowledge of the study treatment is essential for the appropriate clinical management or welfare of the subject. Whenever possible, the Investigator must first discuss options with the GSK Medical Monitor or appropriate GSK study personnel **before** unblinding the subject’s treatment assignment. If this is impractical, the Investigator must notify GSK as soon as possible, but without revealing the treatment assignment of the unblinded subject, unless that information is important

for the safety of subjects currently in the study. The date and reason for the unblinding must be recorded in the appropriate data collection tool.

GSK's Global Clinical Safety and Pharmacovigilance (GCSP) staff may unblind the treatment assignment for any subject with an SAE. If the SAE requires that an expedited regulatory report be sent to one or more regulatory agencies, a copy of the report, identifying the subject's treatment assignment, may be sent to clinical Investigators in accordance with local regulations and/or GSK policy.

11.2. Packaging and Labeling

The contents of the label will be in accordance with all applicable regulatory requirements.

11.3. Preparation/Handling/Storage/Accountability

A description of the methods and materials required for subject administration of GSK2890457 are provided in Appendix 5: Directions for Taking Study Medicine.

Study treatment must be dispensed or administered according to procedures described herein. Only subjects enrolled in the study may receive study treatment. Only authorized site staff may supply or administer study treatment. All study treatment must be stored in a secure area with access limited to the Investigator and authorized site staff. Study treatment is to be stored as indicated on the product label. Maintenance of a temperature log (manual or automated) is required.

The Investigator, institution, or the head of the medical institution (where applicable) is responsible for study treatment accountability, reconciliation, and record maintenance. The Investigator or the head of the medical institution (where applicable), or designated site staff (e.g., storage manager, where applicable) must maintain study treatment accountability records throughout the course of the study. The responsible person(s) will document the amount of study treatment received from and returned to GSK and the amount supplied and/or administered to and/or returned by subjects. The required accountability unit for this study will be a kit. Discrepancies are to be reconciled or resolved. Procedures for final disposition of unused study treatment are listed in the SPM.

Investigational product is not expected to pose significant occupational safety risk to site staff under normal conditions of use and administration. However, precautions are to be taken to avoid direct skin contact, eye contact, and generating aerosols or mists. In the case of unintentional occupational exposure notify the monitor, Medical Monitor and/or study manager. Take adequate precautions to avoid direct eye or skin contact and the generation of aerosols or mists. Notify the monitor of any unintentional occupational exposure. A Material Safety Data Sheet (MSDS)/equivalent document describing occupational hazards and recommended handling precautions is available upon request from GSK.

11.4. Assessment of Compliance

When subjects are dosed at the study site, they will receive study treatment directly from the designated unblinded study staff member, under medical supervision. The date and time of each dose administered in the clinic will be recorded in the source documents. The dose of study treatment and study participant identification will be confirmed at the time of dosing by a member of the study site staff other than the person administering the study treatment.

When dosing at home, subjects will complete a brief diary to indicate if they changed how the dose was taken from the initial instructions.

Study site personnel will ensure that each subject knows how to mix the components of GSK2890457 correctly, and how to take the study treatment so that it is ingested completely.

In Part B, study site personnel will instruct each subject how to correctly administer liraglutide subcutaneously.

11.5. Treatment of Study Treatment Overdose

For this study, any dose of:

- GSK2890457 > 40g within a 24 hour time period [±1 hour]
- Liraglutide >1.8mg within a 24 hour time period [± 1 hour]

will be considered an overdose.

11.5.1. GSK2890457 Overdose

GSK does not recommend specific treatment for an overdose. The Investigator will use clinical judgment to treat any overdose.

11.5.2. Metformin Overdose

An overdose of metformin should be managed as recommended in the Prescribing Information.

Overdose of metformin hydrochloride has occurred, including ingestion of amounts greater than 50 grams. Hypoglycemia was reported in approximately 10% of cases, but no causal association with metformin hydrochloride has been established. Lactic acidosis has been reported in approximately 32% of metformin overdose cases. Metformin is dialyzable with a clearance of up to 170mL/min under good hemodynamic conditions. Therefore, hemodialysis may be useful for removal of accumulated drug from patients in whom metformin overdosage is suspected [GLUCOPHAGE (Metformin) Package Insert].

11.5.3. Victoza (Liraglutide) Overdose

In a clinical trial, one patient with T2D experienced single overdose of Victoza 17.4mg subcutaneous (10 times the maximum recommended dose). Effects of the overdose included severe nausea and vomiting requiring hospitalization. No hypoglycemia was reported. The patient recovered without complications. In the event of overdosage, appropriate supportive treatment should be initiatied according to the patient's clinical signs and symptoms.

12. ADVERSE EVENTS (AE) AND SERIOUS ADVERSE EVENTS (SAE)

The Investigator or site staff is responsible for detecting, documenting and reporting events that meet the definition of an AE or SAE.

In Part A, AEs will be collected from the start of dosing until the Follow-up Visit. In Parts B and C, AEs will be collected from the start of the Stabilization Period until the Follow-up Visit.

SAEs will be collected over the same time period as stated above for AEs. However, any SAEs assessed as related to study participation (e.g. study treatment, protocol-mandated procedures, invasive tests, or change in existing therapy) or related to a GSK product will be recorded from the time a subject consents to participate in the study up to and including any follow-up contact. All SAEs will be recorded and reported to GSK within 24 hours, as indicated in Section 12.6.

Investigators are not obligated to actively seek AEs or SAEs in former study participants. However, if the Investigator learns of any SAE, including a death, at any time after a subject has been discharged from the study, and he/she considers the event reasonably related to the study treatment or study participation, the Investigator would promptly notify GSK.

12.1. Definition of Adverse Events

An AE is any untoward medical occurrence in a patient or clinical investigation subject, temporally associated with the use of a medicinal product, whether or not considered related to the medicinal product.

Note: An AE can therefore be any unfavorable and unintended sign (including an abnormal laboratory finding), symptom, or disease (new or exacerbated) temporally associated with the use of a medicinal product.

Events meeting the definition of an AE **include**:

- Any abnormal laboratory test results (hematology, clinical chemistry, or urinalysis) or other safety assessments (e.g., ECGs, radiological scans, vital signs measurements), including those that worsen from baseline, and felt to be clinically significant in the medical and scientific judgment of the Investigator.

- Exacerbation of a chronic or intermittent pre-existing condition including either an increase in frequency and/or intensity of the condition.
- New conditions detected or diagnosed after study treatment administration even though it may have been present prior to the start of the study.
- Signs, symptoms, or the clinical sequelae of a suspected interaction.
- Signs, symptoms, or the clinical sequelae of a suspected overdose of either study treatment or a concomitant medication (overdose per se will not be reported as an AE/SAE unless this is an intentional overdose taken with possible suicidal/self-harming intent. This should be reported regardless of sequelae.).

Events that **do not** meet the definition of an AE include:

- Any clinically significant abnormal laboratory findings or other abnormal safety assessments that are associated with the underlying disease, unless judged by the Investigator to be more severe than expected for the subject's condition.
- The disease/disorder being studied, or expected progression, signs, or symptoms of the disease/disorder being studied, unless more severe than expected for the subject's condition.
- Medical or surgical procedure (e.g., endoscopy, appendectomy); the condition that leads to the procedure is an AE.
- Situations where an untoward medical occurrence did not occur (social and/or convenience admission to a hospital).
- Anticipated day-to-day fluctuations of pre-existing disease(s) or condition(s) present or detected at the start of the study that do not worsen.

12.2. Definition of Serious Adverse Events

If an event is not an AE per Section 12.1, then it can not be an SAE even if serious conditions are met (e.g., hospitalization for signs/symptoms of the disease under study, death due to progression of disease, etc).

An SAE is any untoward medical occurrence that, at any dose:

a. Results in death
b. Is life-threatening

   NOTE: The term 'life-threatening' in the definition of 'serious' refers to an event in which the subject was at risk of death at the time of the event. It does not refer to an event, which hypothetically might have caused death, if it were more severe.

c. Requires hospitalization or prolongation of existing hospitalization

   NOTE: In general, hospitalization signifies that the subject has been detained (usually involving at least an overnight stay) at the hospital or emergency ward for observation and/or treatment that would not have been appropriate in the physician's office or out-patient setting. Complications that occur during hospitalization are AEs. If a complication prolongs hospitalization or fulfills any other serious criteria,

the event is serious. When in doubt as to whether "hospitalization" occurred or was necessary, the AE should be considered serious.

Hospitalization for elective treatment of a pre-existing condition that did not worsen from baseline is not considered an AE.

d. Results in disability/incapacity, or

NOTE: The term disability means a substantial disruption of a person's ability to conduct normal life functions. This definition is not intended to include experiences of relatively minor medical significance such as uncomplicated headache, nausea, vomiting, diarrhea, influenza, and accidental trauma (e.g. sprained ankle) which may interfere or prevent everyday life functions but do not constitute a substantial disruption.

e. Is a congenital anomaly/birth defect

f. Medical or scientific judgment should be exercised in deciding whether reporting is appropriate in other situations, such as important medical events that may not be immediately life-threatening or result in death or hospitalization but may jeopardize the subject or may require medical or surgical intervention to prevent one of the other outcomes listed in the above definition. These should also be considered serious. Examples of such events are invasive or malignant cancers, intensive treatment in an emergency room or at home for allergic bronchospasm, blood dyscrasias or convulsions that do not result in hospitalization, or development of drug dependency or drug abuse.

g. Is associated with liver injury **and** impaired liver function defined as:

- ALT ≥ 3xULN and total bilirubin* ≥ 2xULN (>35% direct), **or**
- ALT ≥ 3xULN and INR** > 1.5.

* Serum bilirubin fractionation should be performed if testing is available; if unavailable, measure urinary bilirubin via dipstick. If fractionation is unavailable and ALT ≥ 3xULN and total bilirubin ≥ 2xULN, then the event is still to be reported as an SAE.

** INR testing not required per protocol and the threshold value does not apply to subjects receiving anticoagulants. If INR measurement is obtained, the value is to be recorded on the SAE form.

12.3. Method of Detecting AEs and SAEs

Care will be taken not to introduce bias when detecting AEs and/or SAEs. Open-ended and non-leading verbal questioning of the subject is the preferred method to inquire about AE occurrence. Appropriate questions include:

- "How are you feeling?"
- "Have you had any (other) medical problems since your last visit/contact?"
- "Have you taken any new medicines, other than those provided in this study, since your last visit/contact?"

12.4. Recording of AEs and SAEs

When an AE/SAE occurs, it is the responsibility of the Investigator to review all documentation (e.g., hospital progress notes, laboratory, and diagnostics reports) relative to the event. The Investigator will then record all relevant information regarding an AE/SAE in the appropriate data collection tool.

It is not acceptable for the Investigator to send photocopies of the subject's medical records to GSK in lieu of completion of the GSK, AE/SAE data collection tool. However, there may be instances when copies of medical records for certain cases are requested by GSK. In this instance, all subject identifiers, with the exception of the subject number, will be blinded on the copies of the medical records prior to submission of to GSK.

The Investigator will attempt to establish a diagnosis of the event based on signs, symptoms, and/or other clinical information. In such cases, the diagnosis will be documented as the AE/SAE and not the individual signs/symptoms.

Subject-completed questionnaires and the collection of AE data are independent components of the study. Responses to each question in the questionnaire will be treated in accordance with standard scoring and statistical procedures detailed by the scale's developer, where applicable. The use of a single question from a multidimensional health survey to designate a cause-effect relationship to an AE is inappropriate.

12.5. Evaluating AEs and SAEs

12.5.1. Assessment of Intensity

The Investigator will make an assessment of intensity for each AE and SAE reported during the study and will assign it to one of the following categories:

- Mild: An event that is easily tolerated by the subject, causing minimal discomfort and not interfering with everyday activities.
- Moderate: An event that is sufficiently discomforting to interfere with normal everyday activities.
- Severe: An event that prevents normal everyday activities.

An AE that is assessed as severe will not be confused with an SAE. Severity is a category utilized for rating the intensity of an event; and both AEs and SAEs can be assessed as severe. An event is defined as 'serious' when it meets at least one of the pre-defined outcomes as described in the definition of an SAE.

12.5.2. Assessment of Causality

The Investigator is obligated to assess the relationship between study treatment and the occurrence of each AE/SAE. A "reasonable possibility" is meant to convey that there are facts/evidence or arguments to suggest a causal relationship, rather than a relationship cannot be ruled out. The Investigator will use clinical judgment to determine the relationship. Alternative causes, such as natural history of the underlying diseases, concomitant therapy, other risk factors, and the temporal relationship of the event to the study treatment will be considered and investigated. The Investigator will also consult the Investigator Brochure (IB) and/or Product Information, for marketed products, in the determination of his/her assessment.

For each AE/SAE the Investigator must document in the medical notes that he/she has reviewed the AE/SAE and has provided an assessment of causality.

There may be situations when an SAE has occurred and the Investigator has minimal information to include in the initial report to GSK. However, **it is very important that the Investigator always make an assessment of causality for every event prior to the initial transmission of the SAE data to GSK.** The Investigator may change his/her opinion of causality in light of follow-up information, amending the SAE data collection tool accordingly. The causality assessment is one of the criteria used when determining regulatory reporting requirements.

12.6. Follow-up of AEs and SAEs

After the initial AE/SAE report, the Investigator is required to proactively follow each subject at subsequent visits/contacts. All AEs and SAEs will be followed until resolution, until the condition stabilizes, until the event is otherwise explained, or until the subject is lost to follow-up.

The Investigator is obligated to perform or arrange for the conduct of supplemental measurements and/or evaluations as may be indicated or as requested by GSK to elucidate as fully as possible the nature and/or causality of the AE or SAE. The Investigator is obligated to assist. This may include additional laboratory tests or investigations, histopathological examinations or consultation with other health care professionals. If a subject dies during participation in the study or during a recognized follow-up period, the Investigator will provide GSK with a copy of any post-mortem findings, including histopathology.

New or updated information will be recorded in the originally completed data collection tool. The Investigator will submit any updated SAE data to GSK within the designated reporting time frames.

12.7. Prompt Reporting of SAEs to GSK

Once the Investigator determines that an event meets the protocol definition of an SAE, the SAE will be reported to GSK **within 24 hours**. Any follow-up information on a previously reported SAE will also be reported to GSK within 24 hours.

If the Investigator does not have all information regarding an SAE, he/she will not wait to receive additional information before notifying GSK of the event and completing the appropriate data collection tool. The Investigator will always provide an assessment of causality at the time of the initial report as described in Section 12.5.2, Assessment of Causality.

The primary mechanism for reporting SAEs to GSK will be the electronic data collection tool. If the electronic system is unavailable for greater than 24 hours, the site will use a back-up paper SAE data collection tool and fax it to the GSK Medical Monitor. Then the site will enter the serious adverse event data into the electronic system as soon as they become available.

After the study is completed at a given site, the electronic data collection tool (e.g., InForm system) will be taken off-line to prevent the entry of new data or changes to existing data. If a site receives a report of a new SAE from a study participant or receives updated data on a previously reported SAE after the electronic data collection tool has been taken off-line, the site can report this information on a paper SAE form or to their GSK protocol contact by telephone.

GSK contacts for SAE receipt can be found at the beginning of this protocol on the Sponsor/Medical Monitor Contact Information page.

12.8. Regulatory Reporting Requirements for SAEs

Prompt notification of SAEs by the Investigator to GSK is essential so that legal obligations and ethical responsibilities towards the safety of subjects are met.

GSK has a legal responsibility to notify both the local regulatory authority and other regulatory agencies about the safety of a product under clinical investigation. GSK will comply with country specific regulatory requirements relating to safety reporting to regulatory authorities, IRBs/IECs and Investigators.

Investigator safety reports are prepared for suspected unexpected serious adverse reactions according to local regulatory requirements and GSK policy and are forwarded to Investigators as necessary. An Investigator who receives an Investigator safety report describing an SAE(s) or other specific safety information (e.g., summary or listing of SAEs) from GSK will file it with the IB and will notify the IRB/IEC, if appropriate according to local requirements.

13. LIVER CHEMISTRY FOLLOW-UP PROCEDURES

Refer to the diagram in Appendix 1 for a visual presentation of the procedures listed below.

The procedures listed below are to be followed if a subject meets the liver chemistry stopping criteria defined in Section 4.6.1.1:

- Immediately withdraw the subject from study treatment

- Notify the GSK Medical Monitor within 24 hours of learning of the abnormality to confirm the subject's study treatment cessation and follow-up.
- Complete the "Safety Follow-Up Procedures" listed below.
- Complete the liver event case report forms. If the event also meets the criteria of an SAE (see Section 12.2), the SAE data collection tool will be completed separately with the relevant details.
- Upon completion of the safety follow-up withdraw the subject from the study unless further safety follow up is required or GSK Medical Governance approval of drug restart is granted.
- Do not restart investigational product unless written approval is granted by GSK Medical Governance (see Section 13), whereupon the subject continues in the study after completion of the liver chemistry monitoring.

Safety Follow-Up Procedures for subjects with ALT ≥ 3xULN:

- Monitor subjects weekly until liver chemistries (ALT, AST, alkaline phosphatase, bilirubin) resolve, stabilize or return to within baseline values.

Safety Follow-Up Procedures for subjects with ALT ≥3xULN and total bilirubin ≥2xULN (>35% direct bilirubin); or ALT ≥ 3xULN and INR[1] > 1.5:

- This event is considered an SAE (see Section 12.2). Serum bilirubin fractionation should be performed if testing is available. If fractionation is unavailable, urinary bilirubin is to be measured via dipstick (a measurement of direct bilirubin, which would suggest liver injury).
- Make every reasonable attempt to have subjects return to the clinic within 24 hours for repeat liver chemistries, additional testing, and close monitoring (with specialist or hepatology consultation recommended).
- Monitor subjects twice weekly until liver chemistries (ALT, AST, alkaline phosphatase, bilirubin) resolve, stabilize or return to within baseline values.

In addition, for all subjects with ALT ≥ 3xULN, every attempt must be made to also obtain the following:

- Viral hepatitis serology including:
  - Hepatitis A IgM antibody.
  - Hepatitis B surface antigen and Hepatitis B Core Antibody (IgM).
  - Hepatitis C RNA.
  - Cytomegalovirus IgM antibody.

[1] INR testing not required per protocol and the threshold value does not apply to subjects receiving anticoagulants.

- Epstein-Barr viral capsid antigen IgM antibody (or if unavailable, obtain heterophile antibody or monospot testing).
- Hepatitis E IgM antibody.

- Blood sample for pharmacokinetic (PK) analysis will not be obtained.
- Serum creatine phosphokinase (CPK) and lactate dehydrogenase (LDH).
- Fractionate bilirubin, if total bilirubin ≥ 2xULN.
- Assess eosinophilia
- Record the appearance or worsening of clinical symptoms of hepatitis (fatigue, nausea, vomiting, right upper quadrant pain or tenderness, fever, rash or eosinophilia) on the AE CRF.
- Record use of concomitant medications, acetaminophen, herbal remedies, other over the counter medications, or putative hepatotoxins on the Concomitant Medications CRF.
- Record alcohol use on the Liver Events CRF.

The following are required for subjects with ALT ≥ 3xULN **and** bilirubin ≥ 2xULN (>35% direct) but are optional for other abnormal liver chemistries:

- Anti-nuclear antibody, anti-smooth muscle antibody, and Type 1 anti-liver kidney microsomal antibodies.
- Serum acetaminophen adduct HPLC assay (quantifies potential acetaminophen contribution to liver injury in subjects with definite or likely acetaminophen use in the preceding week [James, 2009]).
- Liver imaging (ultrasound, magnetic resonance, or computerized tomography) to evaluate liver disease.
- The Liver Imaging and/or Liver Biopsy CRFs are also to be completed if these tests are performed.

14. STUDY CONDUCT CONSIDERATIONS

14.1. Posting of Information on Publicly Available Clinical Trial Registers

Study information from this protocol will be posted on publicly available clinical trial registers before enrollment of subjects begins.

14.2. Regulatory and Ethical Considerations, Including the Informed Consent Process

The study will be conducted in accordance with all applicable regulatory requirements.

The study will also be conducted in accordance with ICH Good Clinical Practice (GCP), all applicable subject privacy requirements, and, the guiding principles of the 2008 Declaration of Helsinki. This includes, but is not limited to, the following:

- IRB/IEC review and favorable opinion/approval to conduct the study and of any subsequent relevant amended documents.
- Written informed consent (and any amendments) to be obtained for each subject before participation in the study.
- Investigator reporting requirements (e.g., reporting of AEs/SAEs/protocol deviations to IRB/IEC).

Written informed consent must be obtained from each subject prior to participation in the study.

In approving the clinical protocol the IEC/IRB and, where required, the applicable regulatory agency are also approving the optional assessments unless otherwise indicated. Where permitted by regulatory authorities, approval of the optional assessments can occur after approval is obtained for the rest of the study. If so, then the written approval will clearly indicate approval of the optional assessments is being deferred and the study, except for the optional assessments, can be initiated. When the optional assessments are not approved, then the approval for the rest of the study will clearly indicate this and therefore, the optional assessments will not be conducted.

14.3. Quality Control (Study Monitoring)

In accordance with applicable regulations including GCP, and GSK procedures, GSK monitors will contact the site prior to the start of the study to review with the site staff the protocol, study requirements, and their responsibilities to satisfy regulatory, ethical, and GSK requirements. When reviewing data collection procedures, the discussion will also include identification, agreement and documentation of data items for which the eCRF will serve as the source document.

GSK will monitor the study and site activity to verify that the:

- Data are authentic, accurate, and complete.
- Safety and rights of subjects are being protected.
- Study is conducted in accordance with the currently approved protocol and any other study agreements, GCP, and all applicable regulatory requirements.

The Investigator and the head of the medical institution (where applicable) agrees to allow the monitor direct access to all relevant documents.

14.4. Quality Assurance

To ensure compliance with GCP and all applicable regulatory requirements, GSK may conduct a quality assurance assessment and/or audit of the site records, and the regulatory agencies may conduct a regulatory inspection at any time during or after completion of the study. In the event of an assessment, audit or inspection, the Investigator (and institution) must agree to grant the advisor(s), auditor(s) and inspector(s) direct access to all relevant documents and to allocate their time and the time of their staff to discuss the conduct of the study, any findings/relevant issues and to implement any corrective and/or preventative actions to address any findings/issues identified.

14.5. Study and Site Closure

Upon completion or premature discontinuation of the study, the monitor will conduct site closure activities with the Investigator or site staff, as appropriate, in accordance with applicable regulations including GCP, and GSK procedures.

In addition, GSK reserves the right to temporarily suspend or prematurely discontinue this study at any time for reasons including, but not limited to, safety or ethical issues or severe non-compliance. For multicenter studies, this can occur at one or more or at all sites. If GSK determines such action is needed, GSK will discuss this with the Investigator or the head of the medical institution (where applicable), including the reasons for taking such action. When feasible, GSK will provide advance notification to the Investigator or the head of the medical institution, where applicable, of the impending action prior to it taking effect.

If the study is suspended or prematurely discontinued for safety reasons, GSK will promptly inform Investigators or the head of the medical institution (where applicable) and the regulatory authorities of the suspension or premature discontinuation of the study and the reason(s) for the action. If required by applicable regulations, the Investigator or the head of the medical institution (where applicable) must inform the IRB/IEC promptly and provide the reason for the suspension or premature discontinuation.

14.6. Records Retention

Following closure of the study, the Investigator or the head of the medical institution (where applicable) must maintain all site study records, except for those required by local regulations to be maintained by someone else, in a safe and secure location. The records must be maintained to allow easy and timely retrieval, when needed (e.g., audit or inspection), and, whenever feasible, to allow any subsequent review of data in conjunction with assessment of the facility, supporting systems, and staff. Where permitted by local laws/regulations or institutional policy, some or all of these records can be maintained in a format other than hard copy (e.g., microfiche, scanned, electronic); however, caution needs to be exercised before such action is taken. The Investigator must assure that all reproductions are legible and are a true and accurate copy of the original, and meet accessibility and retrieval standards, including re-generating a hard copy, if required. Furthermore, the Investigator must ensure there is an acceptable back-up of these reproductions and that an acceptable quality control process exists for making these reproductions.

GSK will inform the Investigator of the time period for retaining these records to comply with all applicable regulatory requirements. The minimum retention time will meet the strictest standard applicable to that site for the study, as dictated by any institutional requirements or local laws or regulations, or GSK standards/procedures; otherwise, the retention period will default to 15 years.

The Investigator must notify GSK of any changes in the archival arrangements, including, but not limited to, archival at an off-site facility or transfer of ownership of the records in the event the Investigator leaves the site.

14.7. Provision of Study Results to Investigators, Posting of Information on Publically Available Clinical Trials Registers and Publication

Where required by applicable regulatory requirements, an Investigator signatory will be identified for the approval of the clinical study report. Investigators will be provided reasonable access to statistical tables, figures, and relevant reports and will have the opportunity to review the complete study results at a GSK site or other mutually-agreeable location.

GSK will also provide Investigators with the full summary of the study results. Investigators are encouraged to share the summary results with the study subjects, as appropriate.

GSK will provide the Investigator with the randomization codes for their site after completion of the full statistical analysis.

GSK aims to post a results summary to the GSK Clinical Study Register and other publicly available registers no later than 8 months after the last subject's last visit (LSLV) [this applies to each data analysis phase for studies with multiple phases, e.g., primary analysis, follow up analysis etc]. In addition, the aim is to submit a manuscript to a peer-reviewed journal for publication within 18 months of LSLV. GSK also aims to publish the full study protocol on the GSK Clinical Study Register at the time the results of the study are published as a manuscript in the scientific literature.

When manuscript publication in a peer-reviewed journal is not feasible, further study information will be posted to the GSK Clinical Study Register to supplement the results summary.

14.8. Data Management

For this study subject data will be entered into GSK defined electronic case report forms (eCRFs), transmitted electronically to GSK or designee and combined with data provided from other sources in a validated data system.

Management of clinical data will be performed in accordance with applicable GSK standards and data cleaning procedures to ensure the integrity of the data, e.g., removing errors and inconsistencies in the data. Adverse events and concomitant medications terms will be coded using MedDRA (Medical Dictionary for Regulatory Activities) and an internal validated medication dictionary, GSKDrug. eCRFs (including queries and audit trails) will be retained by GSK, and copies will be sent to the Investigator to maintain as the Investigator copy. Subject initials will not be collected or transmitted to GSK according to GSK policy.

15. REFERENCES

Angulo P, et al. The NAFLD Fibrosis Score: A Noninvasive System that Identifies Liver Fibrosis in Patients with NAFLD. *Hepatology.* 2007;45:846-54.

Atassi N, Rataj EM, Greenglatt DJ, et al. A phase I pharmacokinetic dosage escalation study of creatine monohydrate in subjects with amyotrophic lateral sclerosis. *Amyo Lat Sclerosis.* 2010;11:508-13.

Bland JM, Altman DG. Statistical methods for assessing agreement between two methods of clinical measurement. *Lancet.* 1986;327:307-10.

GlaxoSmithKline Document Number 2012N141885_00 Study ID GSK2890457 Investigator's Brochure. Report Date 24-Aug-2012

GlaxoSmithKline Document Number 2011N113387_00 Study ID SGA112534 Study Report. Report Date 24-May-2011

GLUCOPHAGE (metformin) Product Information. January, 2009.

Gualano B, Painelli VS, Roschel H, et al. Creatine supplementation does not impair kidney function in type 2 diabetic patients; a randomized double-blind placeco controlle clinical trial. *Eur J Appl Physiol.* 2010;111(5):749-56.

James LP. Pharmacokinetics of acetominophen - protein adducts. *Liver Failure Drg Metab Disp.* 2009;37:1779-84.

Mendes RR, Pires I, Oliveira A, Tirapegui J. Effects of creatine supplementation on the performance and body composition of competitive swimmers. *J Nutr Biochem.* 2004;15:473-8.

VICTOZA (liraglutide) Product Information. April, 2012.

Appendices

Appendix 1: Liver Safety Algorithms

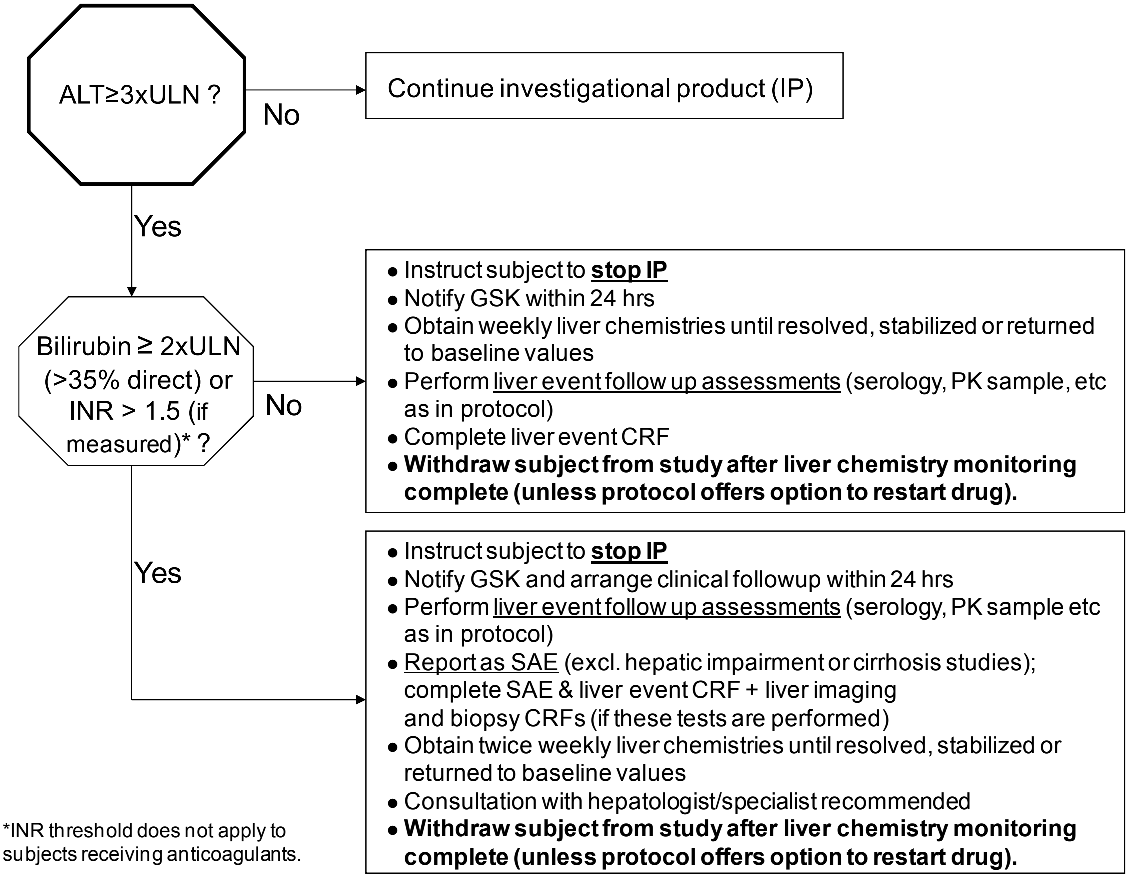

*INR threshold does not apply to subjects receiving anticoagulants.

Appendix 2: Hunger Questionnaire

GlaxoSmithKline Confidential *The Purple One Study*

STUDY SMP116623

SUBJECT NUMBER

HUNGER QUESTIONNAIRE WORKSHEET

Treatment Period
Day -1 and Day 42
See schedule in Section 4.7.6

Complete the Hunger Questionnaire score below.
Rate the following on a scale from 0 to 10. Circle one:

Example: *How strong is your desire to eat?*	0 1 2 3 4 (5) 6 7 8 9 10 — *Very Weak* … *Very Strong*
1. How strong is your desire to eat?	0 1 2 3 4 5 6 7 8 9 10 — *Very Weak* … *Very Strong*
2. How hungry do you feel?	0 1 2 3 4 5 6 7 8 9 10 — *Not Hungry at All* … *As Hungry As I Have Ever Been*
3. How full do you feel?	0 1 2 3 4 5 6 7 8 9 10 — *Not at all* … *Very Full*
4. How much food do you think you could eat?	0 1 2 3 4 5 6 7 8 9 10 — *Nothing* … *Very Much*
Date: ☐☐ ☐☐☐ ☐☐ (*day month year*)	Time: ☐☐ : ☐☐ (*00:00-23:99*)

Appendix 3: GI Symptom Rating Scale (GSRS) Questionnaire

THE GASTROINTESTINAL SYMPTOM RATING SCALE
(GSRS)

Please read this first:

This survey contains questions about how you have been feeling and what it has been like DURING THE PAST WEEK. Mark the choice that best applies to you and your situation with an "X" in the box.

1. Have you been bothered by PAIN OR DISCOMFORT IN YOUR UPPER ABDOMEN OR THE PIT OF YOUR STOMACH during the past week?

☐ No discomfort at all
☐ Minor discomfort
☐ Mild discomfort
☐ Moderate discomfort
☐ Moderately severe discomfort
☐ Severe discomfort
☐ Very severe discomfort

2. Have you been bothered by HEARTBURN during the past week? (By heartburn we mean an unpleasant stinging or burning sensation in the chest.)

☐ No discomfort at all
☐ Minor discomfort
☐ Mild discomfort
☐ Moderate discomfort
☐ Moderately severe discomfort
☐ Severe discomfort
☐ Very severe discomfort

©AstraZeneca, 1995. All rights reserved. (AMOS 97:04)
GSRS - US-English.

These questions should not be used, copied or distributed in any form without permission from AstraZeneca R&D, HEOR, S-431 83 Mölndal, Sweden, PROinformation@astrazeneca.com

3. Have you been bothered by ACID REFLUX during the past week? (By acid reflux we mean the sensation of regurgitating small quantities of acid or flow of sour or bitter fluid from the stomach up to the throat.)

- ☐ No discomfort at all
- ☐ Minor discomfort
- ☐ Mild discomfort
- ☐ Moderate discomfort
- ☐ Moderately severe discomfort
- ☐ Severe discomfort
- ☐ Very severe discomfort

4. Have you been bothered by HUNGER PAINS in the stomach during the past week? (This hollow feeling in the stomach is associated with the need to eat between meals.)

- ☐ No discomfort at all
- ☐ Minor discomfort
- ☐ Mild discomfort
- ☐ Moderate discomfort
- ☐ Moderately severe discomfort
- ☐ Severe discomfort
- ☐ Very severe discomfort

5. Have you been bothered by NAUSEA during the past week? (By nausea we mean a feeling of wanting to throw up or vomit.)

- ☐ No discomfort at all
- ☐ Minor discomfort
- ☐ Mild discomfort
- ☐ Moderate discomfort
- ☐ Moderately severe discomfort
- ☐ Severe discomfort
- ☐ Very severe discomfort

©AstraZeneca, 1995. All rights reserved. (AMOS 97:04)
GSRS - US-English.

These questions should not be used, copied or distributed in any form without permission from AstraZeneca R&D, HEOR, S-431 83 Mölndal, Sweden, PROinformation@astrazeneca.com

6. Have you been bothered by RUMBLING in your stomach during the past week? (Rumbling refers to vibrations or noise in the stomach.)

- ☐ No discomfort at all
- ☐ Minor discomfort
- ☐ Mild discomfort
- ☐ Moderate discomfort
- ☐ Moderately severe discomfort
- ☐ Severe discomfort
- ☐ Very severe discomfort

7. Has your stomach felt BLOATED during the past week? (Feeling bloated refers to swelling often associated with a sensation of gas or air in the stomach.)

- ☐ No discomfort at all
- ☐ Minor discomfort
- ☐ Mild discomfort
- ☐ Moderate discomfort
- ☐ Moderately severe discomfort
- ☐ Severe discomfort
- ☐ Very severe discomfort

8. Have you been bothered by BURPING during the past week? (Burping refers to bringing up air or gas from the stomach via the mouth, often associated with easing a bloated feeling.)

- ☐ No discomfort at all
- ☐ Minor discomfort
- ☐ Mild discomfort
- ☐ Moderate discomfort
- ☐ Moderately severe discomfort
- ☐ Severe discomfort
- ☐ Very severe discomfort

©AstraZeneca, 1995. All rights reserved. (AMOS 97:04)
GSRS - US-English.

These questions should not be used, copied or distributed in any form without permission from AstraZeneca R&D, HEOR, S-431 83 Mölndal, Sweden, PROinformation@astrazeneca.com

9. Have you been bothered by PASSING GAS OR FLATUS during the past week? (Passing gas or flatus refers to the need to release air or gas from the bowel, often associated with easing a bloated feeling.)

- ☐ No discomfort at all
- ☐ Minor discomfort
- ☐ Mild discomfort
- ☐ Moderate discomfort
- ☐ Moderately severe discomfort
- ☐ Severe discomfort
- ☐ Very severe discomfort

10. Have you been bothered by CONSTIPATION during the past week? (Constipation refers to a reduced ability to empty the bowels.)

- ☐ No discomfort at all
- ☐ Minor discomfort
- ☐ Mild discomfort
- ☐ Moderate discomfort
- ☐ Moderately severe discomfort
- ☐ Severe discomfort
- ☐ Very severe discomfort

11. Have you been bothered by DIARRHEA during the past week? (Diarrhea refers to a too frequent emptying of the bowels.)

- ☐ No discomfort at all
- ☐ Minor discomfort
- ☐ Mild discomfort
- ☐ Moderate discomfort
- ☐ Moderately severe discomfort
- ☐ Severe discomfort
- ☐ Very severe discomfort

©AstraZeneca, 1995. All rights reserved. (AMOS 97.04)
GSRS - US-English.

These questions should not be used, copied or distributed in any form without permission from AstraZeneca R&D, HEOR, S-431 83 Mölndal, Sweden, PROinformation@astrazeneca.com

12. Have you been bothered by LOOSE STOOLS during the past week? (If your stools (motions) have been alternately hard and loose, this question only refers to the extent you have been bothered by the stools being loose.)

- ☐ No discomfort at all
- ☐ Minor discomfort
- ☐ Mild discomfort
- ☐ Moderate discomfort
- ☐ Moderately severe discomfort
- ☐ Severe discomfort
- ☐ Very severe discomfort

13. Have you been bothered by HARD STOOLS during the past week? (If your stools (motions) have been alternately hard and loose, this question only refers to the extent you have been bothered by the stools being hard.)

- ☐ No discomfort at all
- ☐ Minor discomfort
- ☐ Mild discomfort
- ☐ Moderate discomfort
- ☐ Moderately severe discomfort
- ☐ Severe discomfort
- ☐ Very severe discomfort

14. Have you been bothered by an URGENT NEED TO HAVE A BOWEL MOVEMENT during the past week? (This urgent need to go to the toilet is often associated with a feeling that you are not in full control.)

- ☐ No discomfort at all
- ☐ Minor discomfort
- ☐ Mild discomfort
- ☐ Moderate discomfort
- ☐ Moderately severe discomfort
- ☐ Severe discomfort
- ☐ Very severe discomfort

©AstraZeneca, 1995. All rights reserved. (AMOS 97:04)
GSRS - US-English.

These questions should not be used, copied or distributed in any form without permission from AstraZeneca R&D, HEOR, S-431 83 Mölndal, Sweden, PROinformation@astrazeneca.com

15. When going to the toilet during the past week, have you had the SENSATION OF NOT COMPLETELY EMPTYING THE BOWELS? (This feeling of incomplete emptying means that you still feel a need to pass more stool despite having exerted yourself to do so.)

- ☐ No discomfort at all
- ☐ Minor discomfort
- ☐ Mild discomfort
- ☐ Moderate discomfort
- ☐ Moderately severe discomfort
- ☐ Severe discomfort
- ☐ Very severe discomfort

PLEASE CHECK THAT ALL QUESTIONS HAVE BEEN ANSWERED!

THANK YOU FOR YOUR CO-OPERATION.

©AstraZeneca, 1995. All rights reserved. (AMOS 97:04)
GSRS - US-English.

These questions should not be used, copied or distributed in any form without permission from AstraZeneca R&D, HEOR, S-431 83 Mölndal, Sweden, PROinformation@astrazeneca.com

Appendix 4: Background and End-of-Study Questions

Background Info to be collected at baseline:

- Duration of diabetes
- How has subject's weight changed since started taking metformin
    - Duration of metformin use should be captured on Con Med form
    - Record ± pounds (or 'unknown')
    - Note "according to subject" or "according to medical records"
- How long has it been since this subject most recently took systemic antibiotics?
    - Record time (months/years)
    - Note "according to subject" or "according to medical records"
- How often in the last two years has subject taken antibiotics?
- Have there, at any time in the subject's life, been periods where antibiotics have taken for periods lasting three months or more? Provide details, if known.
- Was this subject delivered by C-section or by vaginal delivery?
- Was subject bottle-fed, breast-fed, both, or don't know?
- In a typical week, subject consumes how many bottles/cans of DIET soft drink? (Note – non-diet drinks are captured in the food-frequency questionnaire, but it doesn't ask for diet drinks.)

End-of-Study Questions:

- During the time you've been taking study drug have you noticed any changes in the types of foods you choose to eat? Yes/No If Yes, Specify:
- Think about any side effects you may have experienced during the dosing phase of this study (not stabilization phase) and about any weight or blood glucose change you may have seen. Assuming the drug is sold in a good-tasting, easy-to-take form, such as a chew or small bar, would this be a product you would want to take? Yes/No/ Comment
- It may not have been easy for you to take the study medicine as a drink and capsules. We are trying to make this easier for other subjects.
    - Is there anything that you did that helped you prepare the study medicine that we should add to the instructions?
    - Is there anything that you did that helped you avoid mistakes in making up the study medicine?
    - Is there anything that you did that helped you take the study medicine?

Appendix 5: Directions for Taking Study Medicine

SMP116623 Confidential *The Purple One Study*

Directions for Taking Study Medicine

Study medicine will be provided to you in kits, each containing a 5 gram dose.
Each kit contains:

- 3 foil packets which contain powders of different color
- A bottle containing 3 capsules

Take your medication as instructed by the site study staff.

- When you are taking a **15-gram** daily dose, you will take 1 complete kit before breakfast and 2 complete kits before dinner.
- When you are taking a **30-gram** daily dose, you will take 3 complete kits before breakfast and 3 complete kits before dinner.
- When you are taking a **40-gram** daily dose, you will take 4 complete kits before breakfast and 4 complete kits before dinner.

Take all of the medicine from all the foil packets, and all of the capsules from the designated number of kits at each scheduled time.

Mixing Instructions:

You will need:

- Blender bottle with wire mixing ball (provided by study site)
- MiO flavoring (provided by study site)
- Study medicine kits (provided by study site). The number of kits you need each time depends on the dose you are scheduled to take (schedule provided by the study site)
- Scissors
- Water, at room temperature
- Plastic bag to collect the empty packets and seals (you will need to bring the empty packets and empty capsule bottles to the clinic for the study staff to check).

1) Fill the Blender bottle with about 12 ounces of water. Use the lines on the side of the bottle to measure. Make sure the wire mixing ball is in the bottle.

- Note:
  i. If you would like the drink less thick, it's ok to add more water.

ii. If you would prefer the drink cool, you may add a little ice, but only **AFTER** mixing all the packets well in the room temperature water.

iii. Do not mix with hot water, as it may affect the medicine.

2) Squeeze the MiO flavoring bottle to add one squirt of MiO flavoring to the water.

   - **Note:** if you decide you like more flavor, it's ok to add more. You don't have to use the flavor at all, but you may not like the taste without it.

3) Powders can cause nose, throat and eye irritation in sensitive people. When you take the study medicine, you will be dissolving 3 different powders in water and these instructions will minimize the risk of irritation for you and people around you:

   a) Choose a preparation area close to a sink so that you can wipe the surface easily after you have finished.

   b) Don't have other people or children around you when you are preparing the drink because they might distract you or they might be sensitive to powders.

   c) Have a plastic bag nearby so that you can easily dispose of empty packets. Keep a damp paper towel close by to wipe up after you have finished making the drink.

   d) The foil packets are rectangular. Hold each foil packet vertically along one of the shorter edges and tap the packet gently to get the contents to the bottom. Using scissors, one at a time cut right across the top of each foil packet, making sure that you cut the packet just below the seal. Empty the contents gently into the water. With the opening of the packet over the bottle, tap the bottom of the packet gently to empty it as completely as possible, but DO NOT shake it vigorously as this can make a dust cloud. Some particles may still cling to the inside of the packet, and this is ok. Put the packet in the plastic bag before you start the next one. You should do this one packet at a time to avoid spilling the powder, and you want to make sure to get the full dose into the water.

   e) When you are taking multiple kits at the same time, empty all of the packets from all of the kits you are dosing into the bottle. (The number of kits is determined by the scheduled dose; the study site will provide this schedule to you.)

   f) Screw the cap on tightly and make sure the white snap-cover is tightly closed (push until you hear the "snap" – the mixture may stain if spilled). Place a finger over the cap and then **shake the bottle until well mixed (about 10 seconds)**. **This is important.**

   g) If you find that the mixture is too thick for you, you can add more water AFTER the powders have been dissolved. Do not add more water while there are powders sitting on the water because this can cause dust to form.

   h) Wipe the preparation surface with a damp paper towel when you have finished.

4) Drink all of the contents within 15 minutes of mixing. You may notice some lumps as you drink, that is ok. When done, add a little more water (2 or more ounces) to the container. Once again, screw the cap tightly and make sure the white snap-cover is tightly closed. Place a finger over the cap and **shake well** and drink as before to get as much of the medicine as possible. Some bits may stick to the inside of the bottle, and this is ok.

5) After this, take the capsules from the kit(s) with 8 ounces of plain water. Be sure to take one capsule at a time. Also make sure that you take a drink of water before and after each capsule so that it is swallowed properly. This will clean your mouth of the color from the powder drink.

6) Wash the Blender bottle and the wire mixing ball with soap and hot water immediately to clean before next use. You may need to use a brush to clean the cap and the snap-on lid. Dried spots are more difficult to remove. Rinse well. Always be sure that you put the wire mixing ball back in the bottle for the next dose.

Important note: Some subjects may find it difficult to take GSK2890457, especially at the highest dose of 40g. These instructions may be modified to make it easier for you to take the study medicine. Please contact the study coordinator for alternatives that may make it easier to take GSK2890457. Once you mix up the powders with the water, you must take all of it at that time.

If you have found a simple way to make it easier to take the study medicine, please let the study coordinator know as it may help other subjects in the study.

Make sure you keep well hydrated during the day by drinking water, rather than sugared sodas, flavoured drinks or fruit juices.

<u>**Frequent Questions:**</u>

1) **My teeth and tongue are purple! Is this OK?**
   a) The drink will be purple to dark red-purple in color, and your mouth and teeth may look purple after drinking. This is not harmful. Drinking the water to take the capsules or brushing your teeth will make the color go away. If your fingers turn purple from handling the powder, the color will come off with soap and water.
2) **I'm thirsty after I drink this medicine. Is this OK?**
   a) Yes. Some people are thirsty when they are taking this medicine (this is not harmful). You can drink extra water, but as usual, you shouldn't drink high-calorie drinks like sugared-sodas or fruit juice.
3) **What if I skip breakfast some days, or I'm not hungry?**
   a) It's fine to take your study medicine without food. Make sure you drink water to keep hydrated. Some people find they have less of an appetite after drinking the medicine. Just make sure that the morning and evening doses are spaced out during the day (i.e., approximately breakfast time and dinner time).
4) **What if I miss a dose? Or if some of the dose spills?**
   a) If you miss a dose, and it is less than 3 hours since you were supposed to take it, go ahead and take the dose. If it is more than 3 hours since the dose you missed, skip that dose and take the next dose at the regularly scheduled time. Do not take 2 doses at the same time. Be sure to tell the study coordinator about missed doses or partial doses at your next visit so we can keep track of what you actually take. Also, be sure to tell the study coordinator if you were unable to take some parts of the study medicine.

5) **I normally take my metformin with a meal, but sometimes I do not feel like eating after taking the study medicine. What shall I do?**
   a) It is OK to take the metformin with water after taking the study medicine, even though you have not eaten food.
   b) Tell the study staff if taking the metformin like this makes you feel unwell. They will help you find an alternative way of taking the study medicine or the metformin.

6) **What should I do if I have irritation of the nose, throat or eyes?**
   a) If this happens because of a large spill (for example, you have accidentally dropped open packets)
      i) Move away from the preparation area and keep other people away.
      ii) Wash your hands with soap and water. Then wash you face and eyes with plenty of water.
      iii) Place the mask provided over your nose and mouth and then go to clean up the area with paper wipes that have been dampened with water. Continue cleaning until there are no more powders in or around the spillage area.
      iv) Next time you visit the clinical unit, tell the study nurse that this happened. You will be asked how much of the study medicine was lost.
   b) If this happens while you are preparing the drink normally
      i) Make sure that you are not creating dust clouds by shaking the open packets or bottle. Handle the open packet gently and don’t squeeze it while it has powder inside. Immediately put each empty packet and its seal into a plastic bag.
      ii) Next time you visit the clinical unit, tell the study nurse. The nurse will check that you are mixing the powders in the drink properly.
      iii) If simple adjustments solve the irritation problem, you can continue making up the drink without extra precautions. If not, you will be told to wear a mask, and possibly eye glasses, while preparing the drink.

Thank you for participating in this clinical study!

Appendix 6: Mindful Eating Guide

Mindful Eating
The Purple One Study

The treatment you will take in this study may cause you to be less hungry. We're not asking you to count calories or to try to 'starve' yourself to lose weight. To lose weight during the study, it will be important for you to "listen to your body". This means that you should eat when you are hungry, but stop eating when you aren't.

This is easy to say, but much harder to do. Most of us eat in a pattern. The pattern may be driven by time of day ("If it's noon, it must be time for lunch"), by habit ("I always eat a sandwich, chips, and an apple"), or by training ("Clean your plate!"). Sometimes we eat because we're tired, or worried, or bored.

Mindful Eating is an approach to eating that helps you pay attention to whether you are actually hungry or not. Here are some things that may help you pay attention:

- Ask yourself "Am I actually hungry?" Try to eat when your body tells you to, instead of out of habit.
- Take smaller servings than usual. When you've eaten them, ask yourself if you really want more. If you're still hungry – take another serving. But if not, stop eating. If you get hungry later, you can always have a snack.
- Slow down. This medicine may make you feel uncomfortably full with less food. By eating more slowly, you can judge how much you actually need.
- Enjoy your food. Pick foods you like, and that are consistent with any healthy-eating advice your doctor has given you. Pay attention to colors, smells, and tastes. You may find that eating less can mean enjoying more.
- When you are eating, try to focus only on the food. Watching TV, reading, or playing video games while you eat can distract you from listening to your body.

What if the treatment doesn't work for me? What if I receive placebo (a drug that looks the same, but has no effect)?

Mindful Eating can help people lose weight even if they are not taking any medicine. Listening to your body and thinking about why you are eating is always a good approach. You will be given this handout at the beginning of your Stabilization Period in the study, before you start taking study treatment. Use this time to practice Mindful Eating and to get used to thinking about your level of hunger. The study staff will remind you of the importance of Mindful Eating at several visits during the study.

Thank you for participating in this clinical study!

Appendix 7: Victoza (Liraglutide) Medication Guide for Patients

Patient Instructions for Use
Victoza® (liraglutide [rDNA origin]) injection

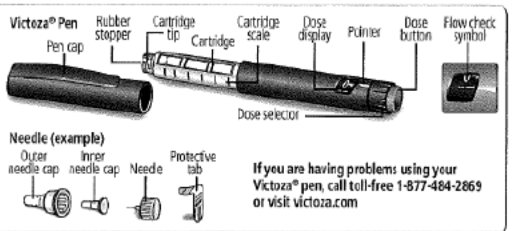

If you are having problems using your Victoza® pen, call toll-free 1-877-484-2869 or visit victoza.com

First read the Medication Guide that comes with your Victoza® pen and then read these Patient Instructions for Use for information about how to use your Victoza® pen the right way.

These instructions do not take the place of talking with your healthcare provider about your medical condition or your treatment.

Your Victoza® pen contains 3 mL of Victoza® and will deliver doses of 0.6 mg, 1.2 mg or 1.8 mg. The number of doses that you can take with a Victoza® pen depends on the dose of medicine that is prescribed for you. Your healthcare provider will tell you how much Victoza® to take.

Victoza® pen should be used with Novo Nordisk disposable needles. Talk to your healthcare provider or pharmacist for more information about needles for your Victoza® pen.

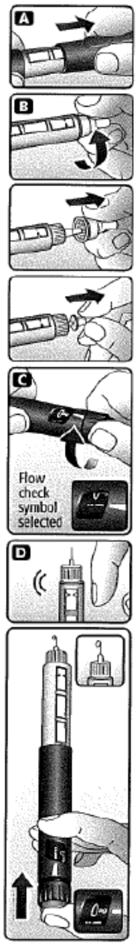

Important Information

- Do not share your Victoza® pen or needles with anyone else. You may give an infection to them or get an infection from them.
- Always use a new needle for each injection.
- Keep your Victoza® pen and all medicines out of the reach of children.
- If you drop your Victoza® pen, repeat "First Time Use For Each New Pen" (steps A through D).
- Be careful not to bend or damage the needle.
- Do not use the cartridge scale to measure how much Victoza® to inject.
- Be careful when handling used needles to avoid needle stick injuries.
- You can use your Victoza® pen for up to 30 days after you use it the first time.

First Time Use for Each New Pen

Step A. Check the Pen

- Take your new Victoza® pen out of the refrigerator.
- Wash hands with soap and water before use.
- Check pen label before each use to make sure it is your Victoza® pen.
- Pull off pen cap.
- Check Victoza® in the cartridge. The liquid should be clear, colorless and free of particles. If not, do not use.
- Wipe the rubber stopper with an alcohol swab.

Step B. Attach the Needle

- Remove protective tab from outer needle cap.
- Push outer needle cap containing the needle straight onto the pen, then screw needle on until secure.
- Pull off outer needle cap. Do not throw away.
- Pull off inner needle cap and throw away. A small drop of liquid may appear. This is normal.

Step C. Dial to the Flow Check Symbol

- Turn dose selector until flow check symbol (⌐) lines up with pointer.

Step D. Prepare the Pen

- Hold pen with needle pointing up.
- Tap cartridge gently with your finger a few times to bring any air bubbles to the top of the cartridge.
- Keep needle pointing up and press dose button until 0 mg lines up with pointer. Repeat steps C and D, up to 6 times, until a drop of Victoza® appears at the needle tip.

If you still see no drop of Victoza®, use a new pen and contact Novo Nordisk at 1-877-484-2869.

Continue to Step G under "Routine Use" ⇨

Routine Use

Step E. Check the Pen

- Take your Victoza® pen from where it is stored.
- Wash hands with soap and water before use.
- Check pen label before each use to make sure it is your Victoza® pen.
- Pull off pen cap.
- Check Victoza® in the cartridge. The liquid should be clear, colorless and free of particles. If not, do not use.
- Wipe the rubber stopper with an alcohol swab.

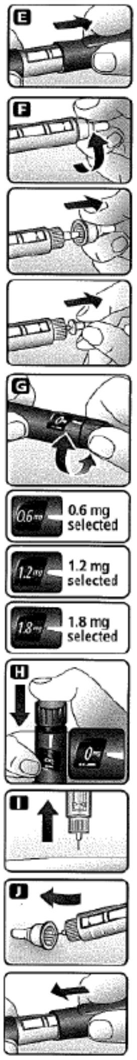

Step F. Attach the Needle

- Remove protective tab from outer needle cap.
- Push outer needle cap containing the needle straight onto the pen, then screw needle on until secure.
- Pull off outer needle cap. Do not throw away.
- Pull off inner needle cap and throw away. A small drop of liquid may appear. This is normal.

Step G. Dial the Dose

- Victoza® pen can give a dose of 0.6 mg (starting dose), 1.2 mg or 1.8 mg. Be sure that you know the dose of Victoza® that is prescribed for you.
- Turn the dose selector until your needed dose lines up with the pointer (0.6 mg, 1.2 mg or 1.8 mg).
- You will hear a "click" every time you turn the dose selector. **Do not set the dose by counting the number of clicks you hear.**
- If you select a wrong dose, change it by turning the dose selector backwards or forwards until the correct dose lines up with the pointer. Be careful not to press the dose button when turning the dose selector. This may cause Victoza® to come out.

Step H. Injecting the Dose

- Insert needle into your skin in the stomach, thigh or upper arm. Use the injection technique shown to you by your healthcare provider. **Do not inject Victoza® into a vein or muscle.**
- Press down on the center of the dose button to inject until 0 mg lines up with the pointer.
- Be careful not to touch the dose display with your other fingers. This may block the injection.
- Keep the dose button pressed down and make sure that you keep the needle under the skin for a full count of 6 seconds to make sure the full dose is injected. Keep your thumb on the injection button until you remove the needle from your skin.

Step I. Withdraw Needle

- You may see a drop of Victoza® at the needle tip. This is normal and it does not affect the dose you just received. If blood appears after you take the needle out of your skin, apply light pressure, but **do not rub the area.**

Step J. Remove and Dispose of the Needle

- Carefully put the outer needle cap over the needle. Unscrew the needle.
- Safely remove the needle from your Victoza® pen after each use.
- Place used needles in a closeable, puncture-resistant container. If your Victoza® pen is empty or if you have been using it for 30 days (even if it is not empty), throw away the used pen. You may use a sharps container (such as a red biohazard container), a hard plastic container (such as an empty detergent bottle), or metal container with a screw top (such as an empty coffee can).
- Ask your healthcare provider for instructions on the right way to dispose of your used needles, pens, and the container. Do not throw the disposal container in the household trash. Do not recycle.

Caring for your Victoza® pen

- After removing the needle, put the pen cap on your Victoza® pen and store your Victoza® pen without the needle attached.
- Do not try to refill your Victoza® pen – it is prefilled and is disposable.
- Do not try to repair your pen or pull it apart.
- Keep your Victoza® pen away from dust, dirt and liquids.
- If cleaning is needed, wipe the outside of the pen with a clean, damp cloth.

How should I store Victoza?

__Before use:__

- Store your new, unused Victoza® pen in the refrigerator at 36°F to 46°F (2°C to 8°C).
- If Victoza® is stored outside of refrigeration (by mistake) prior to first use, it should be used or thrown away within 30 days.
- Do not freeze Victoza® or use Victoza® if it has been frozen. Do not store Victoza® near the refrigerator cooling element.

__Pen in use:__

- Store your Victoza® pen for 30 days at 59°F to 86°F (15°C to 30°C), or in a refrigerator at 36°F to 46°F (2°C to 8°C).
- When carrying the pen away from home, store the pen at a temperature between 59°F to 86°F (15°C to 30°C).
- If Victoza® has been exposed to temperatures above 86°F (30°C), it should be thrown away.
- Protect your Victoza® pen from heat and sunlight.
- Keep the pen cap on when your Victoza® pen is not in use.
- Use a Victoza® pen for only 30 days. Throw away a used Victoza® pen after 30 days, even if some medicine is left in the pen.

Appendix 8: Country Specific Requirements

No country-specific requirements exist.

Appendix 9: Protocol Amendment Changes

AMENDMENT 2

Where the Amendment Applies

This amendment applies to all sites.

Summary of Amendment Changes with Rationale

- Amendment 2 adds that instructions will be given to subjects on how to manage hypoglycemia.
- Papillary thyroid cancer is added to the risks of liraglutide use.
- Guidance is given for management of subjects who are not using the telemonitoring equipment correctly.
- Allowance for flexibility around the time of day that subjects may come in for scheduled visits, allowing for non-fasted sampling, as long as it is noted in the case report form.
- In response to preliminary data from Part A, metformin in Part C will be dosed one hour prior to GSK2890457.
- Clarification that doses of GSK2890457 are to be taken before breakfast and dinner.
- Additional language allowing for dose reduction based on safety and/or tolerability issues.
- Additional information about proceeding with subjects' normal dose of metformin in Part C, by separating it by 1 hour from the GSK2890457. The time interval between the GSK2890457 and liraglutide doses can be adjusted to improve the ability to take GSK2890457.
- Additional details about fasting glucose sampling being conducted by the subject at home.
- Visit window added for visit Days 4 through 35 in Parts B and C (± 1 day).
- Section 4.7.4 corrected to reflect dispensing of study drug on Day 1 (with randomization).
- Section 4.7.7 added to show the updated blood sampling scheme and metformin dosing for P Potential Risks of GSK2890457 and Their Mitigation art B.
- C-Peptide measurements added to table in Section 7.3.5.
- Modified description of the fecal sample collection tube.

List of Specific Changes

Table 1 Potential Risks of GSK2890457 and Their Mitigation

ADDED TEXT

Hypoglycemia	In animal efficacy studies, GSK2890457 has reduced elevated glucose levels to normal when used in combination with a GLP-1 mimetic – no hypoglycaemia has been observed. In these studies, insulin levels were either significantly reduced or showed non-significant elevations. This is consistent with the potential mechanisms of action of GSK2890457 which are believed to be effective only when glucose levels are elevated. Nevertheless, a risk of hypoglycemia in T2D subjects cannot be excluded when co-dosed with other anti-diabetic medications.	Subjects with T2D will monitor fasting and pre-dinner capillary blood glucose (CBG) values daily using a telemonitored glucometer, and will be advised to report any symptoms of hypoglycemia promptly.<br><br>**Subjects will be given instructions on how to handle hypoglycaemia.**

Table 2 Potential Risks of Liraglutide, Alone and Co-Administered with GSK2890457 and Their Mitigation*

ADDED TEXT

Hypoglycemia	In patients with T2D, hypoglycemia has been associated with administration of liraglutide when given in conjunction with other agents, such as insulin and insulin secretagogues such as sulphonylureas that have an intrinsic hypoglycemic risk.<br><br>Experience with liraglutide indicates that the risk of hypoglycemia is very low when it is used as monotherapy in T2D patients. [VICTOZA, 2012 Prescribing Information]	As GSK2890457 does not produce hypoglycemia in animal models and glucose improvement is observed in the absence of elevation of circulating insulin levels, it is unlikely that GSK2890457 will augment the very low risk of hypoglycemia seen with liraglutide.<br><br>Nevertheless, careful monitoring for hypoglycemia is advisable because it may occur when caloric intake is reduced,	-- Exclude subjects with hypoglycemic unawareness<br><br>--Fasting and pre-dinner CBG will be monitored daily using a telemonitored device while subjects are at home.<br><br>--Subjects will be closely monitored for signs and symptoms of hypoglycemia and will be treated appropriately.<br><br>**-Subjects will be given instructions on how to handle hypoglycaemia.**<br>…

ADDED TEXT

Medullary **and Papillary** Thyroid Carcinoma	Liraglutide may be associated with an increased risk of developing medullary thyroid carcinoma. This association is based on chronic exposure studies in rodents and the relevance to humans is unclear.<br><br>**In clinical trials with liraglutide, there were 7 reported cases of papillary thyroid cancer in patients treated with liraglutide and 1 case in a comparator-treated patient (1.5 versus 0.5 cases per 1000 patient-years).**	As the components of GSK2890457 are food ingredients and there were no thyroid lesions in the 6 week rat toxicity study, it is not anticipated that co-dosing for 6 weeks will materially increase the risk to subjects.	--Subjects with a personal or family history of medullary or papillary carcinoma of the thyroid or multiple endocrine neoplasia type 2, or with plasma calcitonin levels at screening >50pg/mL are excluded from the study.<br><br>- A careful thyroid physical examination will be conducted at screening and before the start of the 6-week Treatment Period. A subject should be excluded if an abnormal thyroid mass is detected.

Table 3 Potential Risks of Metformin and Co-administration with GSK2890457

ADDED TEXT

Hypoglycemia	While hypoglycemia has been reported in ≥1.0% to ≤5.0% of metformin patients (and more commonly than placebo) in clinical studies, the risk is very low when it is used as monotherapy in T2D patients.<br><br>This is because metformin improves fasting and prandial glucose levels, without increasing circulating insulin levels.	As GSK2890457 does not produce hypoglycemia in animal models and glucose improvement is observed in the absence of elevation of circulating insulin levels, it is unlikely that GSK2890457 will augment the very low risk of hypoglycemia seen with metformin.<br><br>Nevertheless, careful monitoring for hypoglycemia is advisable because it may occur when caloric intake is reduced.	--Exclude subjects with hypoglycemic unawareness<br><br>--Fasting and pre-dinner CBG will be monitored daily using a telemonitored device while subjects are at home.<br><br>--Subjects will be closely monitored for signs and symptoms of hypoglycemia and will be treated appropriately.<br><br>**-Subjects will be given instructions on how to handle hypoglycaemia.**<br>…

Section 4.2.2. Stabilization Period (12 weeks)

ADDED TEXT

- **Subjects not using the telemonitoring glucose and weight equipment appropriately will be counselled on correct use, and technical issues will be addressed, if necessary. If poor compliance continues and does not improve**

with counselling, this may result in withdrawal from the study, after consultation with the GSK Medical Monitor.

Section 4.2.3. Treatment Period (6 weeks)

ADDED TEXT

- **Subjects not using the telemonitoring glucose and weight equipment appropriately will be counselled on correct use, and technical issues will be addressed, if necessary. If poor compliance continues and does not improve with counselling, this may result in withdrawal from the study, after consultation with the GSK Medical Monitor.**

...

- Subjects will return for clinic visits on Days 7, 14, and 28 for safety evaluations, potential modification of dose (Days 7 and 14), and dispensing of study medications. Drug accountability will be performed to ensure subject compliance with dosing. **Note: If a subject cannot attend the clinic in the morning of these scheduled visits for fasting predose assessments, the subject should take the study treatment at home before eating breakfast and then come to the clinic in the afternoon for study assessments. It will be noted in the CRF that measurements were not completed in a fasted state.**

Section 4.3.2. Stabilization Period (4 weeks)

ADDED TEXT

- **Subjects not using the telemonitoring glucose and weight equipment appropriately will be counselled on correct use, and technical issues will be addressed, if necessary. If poor compliance continues and does not improve with counselling, this may result in withdrawal from the study, after consultation with the GSK Medical Monitor.**

Section 4.3.3. Treatment Period (6 weeks)

ADDED TEXT

- **Subjects not using the telemonitoring glucose and weight equipment appropriately will be counselled on correct use, and technical issues will be addressed, if necessary. If poor compliance continues and does not improve with counselling, this may result in withdrawal from the study, after consultation with the GSK Medical Monitor.**

...

- Subjects will return for clinic visits on Days 7, 14, and 28 for safety evaluations, potential modification of dose (Days 7 and 14), and dispensing of study medications. Drug accountability will be performed to ensure subject compliance with dosing.
  Note: If a subject cannot attend the clinic in the morning of these scheduled

visits for fasting predose assessments, the subject should take the study treatment at home before eating breakfast and then come to the clinic in the afternoon for study assessments. It will be noted in the CRF that measurements were not completed in a fasted state.

PREVIOUS TEXT

The order at dosing should be: take metformin followed by GSK2890457 or placebo, and then the breakfast meal.

REVISED TEXT

The order at dosing should be: take metformin ~~followed by~~ one hour prior to GSK2890457 or placebo, and then the breakfast meal. If the subject is taking metformin BID, the evening dose should be taken one hour prior to dosing of GSK2890457/placebo, and then dinner. Metformin should be taken with a small portion of the meal, with the remainder of the meal consumed after GSK2890457.

Section 4.4.1. GSK2890457 Dosage and Administration (Parts A-C)

ADDED TEXT

- **If a subject comes on Day 4 and says they were having problems with 15 g, then the investigator and GSK Medical Monitor will decide whether the subject can continue in the study if the side effects are tolerable.**
- **If a subject escalated to 30g on Day 4 and then contacts the clinical site to say that they cannot tolerate that dose, the subject should follow the following plan:**
  - **If the subject took 15g on the morning of the day of contact, they should skip the evening dose, and restart 15 g again the next day (5 g in morning and 10 g in evening). The investigator and GSK Medical Monitor can then decide whether to try the subject again on 30 g at a later time.**
  - **If the subject did not take a dose on the day of contact, they can start on 15 g (5 g in morning and 10 g in evening) that same day. The investigator and GSK Medical Monitor can then decide whether to try the subject again on 30g at a later time.**

PREVIOUS TEXT

- If the subject is experiencing minimal side effects at 30g and is willing to attempt a higher dose, the dose should be increased to 40g for the next week, to be taken as 20g (4 unit doses) with breakfast and 20g (4 unit doses) with dinner.

REVISED TEXT

- If the subject is experiencing minimal side effects at 30g and is willing to attempt a higher dose, the dose should be increased to 40g for the next week, to be taken

as 20g (4 unit doses) ~~**with**~~ **before** breakfast and 20g (4 unit doses) ~~**with**~~ **before** dinner.

ADDED TEXT

From Day 15 through Day 42, each subject will remain on a constant dose, **unless in the opinion of the investigator and GSK Medical Monitor the dose should be reduced because of tolerability or safety issues**.

Section 4.4.2. Liraglutide (Part B)

ADDED TEXT

During the Treatment period, the morning dose of GSK2890457 should be taken before administration of liraglutide. As the gastric emptying delay produced by liraglutide may impact the ability of a subject to take the full dose of GSK2890457, the time interval between the GSK2890457 and liraglutide doses can be adjusted to improve tolerability and compliance with GSK2890457 dosing.

Section 4.4.5. Metformin (Part C)

PREVIOUS TEXT

In Part C, T2D subjects will stay on their usual dose and regimen. Subjects taking metformin once-daily should take that dose in the morning throughout the study, to permit appropriate pharmacokinetic evaluation. Subjects can drink GSK2890457 to take the metformin dose.

REVISED TEXT

In Part C, T2D subjects will stay on their usual dose and regimen **of metformin. As preliminary data from Part A indicate that GSK2890457 may reduce the AUC and Cmax of metformin,** subjects taking metformin once-daily should take that dose in the morning **one hour prior to GSK2890457/placebo, followed by breakfast**, throughout the ~~study~~ Treatment period, to **minimize a possible** pharmacokinetic ~~**evaluation**~~ interaction. ~~**Subjects can drink GSK2890457 to take the metformin dose.**~~ **Throughout the Treatment period, subjects taking metformin twice-daily should take the morning dose as above, and the evening dose one hour prior to GSK2890457/placebo, and then eat dinner.**

ADDED TEXT

This protocol allows the Investigator, in consultation with the GSK Medical Monitor, to modify the metformin dose and/or **dosing regimens and/or the** GSK2890457 dose and/or dosing regimen~~s~~ in Part C based on emergent PK and tolerability data from Part A.

Section 4.6. Dose Adjustment/Stopping Criteria

ADDED TEXT

Subjects will remain on their usual metformin dose in Part C **unless the dose and/or dosing regimen is altered based on emergent PK and tolerability data from Part A.**

Section 4.6.1.4. Dose Adjustment/Stopping Safety Criteria Based on Blood Glucose Monitoring (Part B and C)

ADDED TEXT

- When they have fasting CBG values that are >270mg/dL or < 70mg/dL. (When the subject calls the site, site staff should confirm that values being reported were taken correctly, e.g., in the fasted state. Any deviations must be noted in the eCRF).

Section 4.7.2. Part B Time and Events Table: Screening, 1-Week Telemonitoring Run-in and 12-Week Stabilization

ADDED TEXT

C-peptide	X							

Section 4.7.3. Part C Time and Events Table: Screening, 1-Week Telemonitoring Run-in and 4-Week Stabilization for Subjects Remaining on Metformin

ADDED TEXT

C-peptide	X							

Section 4.7.4. Parts B and C Time and Events Table Day -2 through Study End

ADDED TEXT

Procedure	Day -2	Day -1	Day 1	Day 4 (±1 Day)	Day 7 (±1 Day)	Day 14 (±1 Day)	Day 21 (±1 Day)	Day 28 (±1 Day)	Day 35 (±1 Day)	Day 41	Day 42	Day 43	Follow-up (±1 Day)

PREVIOUS TEXT

Dispense/Instruct: Study Medication		X			X	X		X					

REVISED TEXT

Dispense/Instruct: Study Medication		~~X~~	**X**		X	X		X					

Section 4.7.6. Part B: Day -1 and Day 42: Sampling Schedule for Glucose, Insulin, Biomarkers, and PK; Satiety Questionnaire Timepoints

PREVIOUS TEXT

Timepoint	Glucose and Insulin Sample	Other Biomarker Samples	Pharmacokinetic Sample[1] (metformin, liraglutide)	Fasting Exploratory Biomarker Samples	Blood for GSK2890457 components/metabolites[2] Day -1 and Day 42	Hunger Questionnaire	Urine for GSK2890457 components/ metabolites Day -1 and Day 42[3]
**Fasting**	**X[4]**						
**Fasting**	**X[4]**	**X**	**X (predose on Day 42 within 15 minutes of dose)**	**X**	**X (pre-dose)**	**X**	
Time 0 minutes: Dose liraglutide, metformin alone (Day -1) or with GSK2890457 (Day 42)							
Begin eating breakfast immediately after taking study medication(s) and finishing eating in 15min[5]							
15minutes			X		X		
30 minutes	X	X	X		X		
1 hour	X	X	X		X	X	
1.5 hours	X	X					
2 hours	X	X	X		X	X	
4 hours (Pre-Lunch)	X	X	X		X	X	
4 hours	Eat Lunch						Pool 0-12hr
5.5 hours	X	X	X		X		
6 hours						X	
8 hours			X		X	X	
10 hours (Pre-Dinner)	X	X	X		X		
10 hours	Take second dose of metformin (if subject is taking immediate release BID) alone (Day -1) or with GSK2890457 (Day 42) and then eat dinner/evening meal						
11.5 hours	X	X	X		X	X	
12 hours							Pool 12-24hr
14 (bedtime)	X	X					
24 hours	X	X	X (predose, morning dose)				

1. See Section 7.5 for instructions for PK samples. Samples for analysis of metformin or liraglutide will be collected in EDTA containing tubes.

2. A single sample will be collected at each timepoint and quickly processed as described in Section 7.5.2. As some of the components of GSK2890457 are unstable in plasma, the blood for GSK2890457 PK needs to be collected in special tubes containing Potassium Oxalate/sodium fluoride and the blood quickly processed as described in Section 7.5.
3. Urine will be collected at the various intervals and the volume will be determined. A 100mL aliquot from each time interval will be used for the investigation of GSK2890457 metabolites.
4. Two fasting samples 5min apart will be taken for insulin. Baseline insulin level will be the average of the 2 fasting samples.
5. Subjects should make their best effort to consume dose and meal in the allotted time; if unable to complete in time, this will not be considered a protocol violation.

REVISED TEXT

Timepoint	Glucose and Insulin Sample	Other Biomarker Samples	Pharmacokinetic Sample[1] (liraglutide)	Fasting Exploratory Biomarker Samples	Blood for GSK2890457 components/metabolites[2] Day -1 and Day 42	Hunger Questionnaire	Urine for GSK2890457 components/ metabolites Day -1 and Day 42[3]
**Fasting**	**X[4]**						
**Fasting**	**X[4]**	**X**	**X (predose)**	**X**	**X (pre-dose)**	**X**	
Time 0 minutes: Dose liraglutide[6] alone (Day -1) or with GSK2890457 (Day 42)							Pool 0-12hr
Begin eating breakfast immediately after taking study medication(s) and finishing eating in 15min[5]							
15minutes			X		X		
30 minutes	X	X	X		X		
1 hour	X	X	X		X	X	
1.5 hours	X	X					
2 hours	X	X	X		X	X	
4 hours (Pre-Lunch)	X	X	X		X	X	
4 hours	Eat Lunch						
5.5 hours	X	X	X		X		
6 hours						X	
8 hours			X		X	X	
10 hours (Pre-Dinner)	X	X	X		X		
10 hours	Take evening dose of GSK2890457 and then eat dinner/evening meal						

Timepoint	Glucose and Insulin Sample	Other Biomarker Samples	Pharmacokinetic Sample[1] (liraglutide)	Fasting Exploratory Biomarker Samples	Blood for GSK2890457 components/metabolites[2] Day -1 and Day 42	Hunger Questionnaire	Urine for GSK2890457 components/ metabolites Day -1 and Day 42[3]
**Fasting**	**X[4]**						
**Fasting**	**X[4]**	**X**	**X (predose)**	**X**	**X (pre-dose)**	**X**	
11.5 hours	X	X	X		X	X	
12 hours							Pool 12-24hr
14 (bedtime)	X	X					
24 hours	X	X	X (predose, morning dose)				

1. See Section 7.5 for instructions for PK samples. Samples for analysis of liraglutide will be collected in EDTA containing tubes.
2. A single sample will be collected at each timepoint and quickly processed as described in Section 7.5.2. As some of the components of GSK2890457 are unstable in plasma, the blood for GSK2890457 PK needs to be collected in special tubes containing Potassium Oxalate/sodium fluoride and the blood quickly processed as described in Section 7.5.
3. Urine will be collected at the various intervals and the volume will be determined. A 100mL aliquot from each time interval will be used for the investigation of GSK2890457 metabolites.
4. Two fasting samples 5min apart will be taken for insulin. Baseline insulin level will be the average of the 2 fasting samples.
5. Subjects should make their best effort to consume dose and meal in the allotted time; if unable to complete in time, this will not be considered a protocol violation.
6. The time interval between the dose of liraglutide and GSK2890457 may be varied if gastric emptying delay impacts the ability to take the full dose of GSK2890457.

Section 4.7.7. Part C: Day -1 and Day 42: Sampling Schedule for Glucose, Insulin, Biomarkers, and PK; Satiety Questionnaire Timepoints

ADDED TEXT

Timepoint	Glucose and Insulin Sample	Other Biomarker Samples	Pharmacokinetic Sample[1] (metformin)	Fasting Exploratory Biomarker Samples	Blood for GSK2890457 components/metabolites[2] Day -1 and Day 42	Hunger Questionnaire	Urine for GSK2890457 components/ metabolites Day -1 and Day 42[3]
**Fasting (F1)**	**X**						
**Metformin Dose + Snack**			**1 hour prior to GSK2890457 dose**				
**F2**	**X**	**X**	**X (pre GSK2890457 dose)**	**X**	**X (pre-dose)**	**X**	
Time 0 minutes: DoseGSK2890457 (finish within 5 min) (Day 42)							Pool 0-12hr
Begin eating breakfast immediately after taking GSK2890457 and finishing eating in 15min[4]							
15minutes			X		X		
30 minutes	X	X	X		X		
1 hour	X	X	X		X	X	
1.5 hours	X	X					
2 hours	X	X	X		X	X	
4 hours (Pre-Lunch)	X	X	X		X	X	
4 hours	Eat Lunch						
5.5 hours	X	X	X		X		
6 hours						X	
8 hours			X		X	X	
9 hours Metformin + snack			1 hour prior to dinner				
10 hours (Pre-Dinner)	X	X	X		X		
10 hours	Take GSK2890457 (Day 42) and then eat dinner/evening meal						

Timepoint	Glucose and Insulin Sample	Other Biomarker Samples	Pharmacokinetic Sample[1] (metformin)	Fasting Exploratory Biomarker Samples	Blood for GSK2890457 components/metabolites[2] Day -1 and Day 42	Hunger Questionnaire	Urine for GSK2890457 components/ metabolites Day -1 and Day 42[3]
**Fasting (F1)**	**X**						
**Metformin Dose + Snack**			**1 hour prior to GSK2890457 dose**				
**F2**	**X**	**X**	**X (pre GSK2890457 dose)**	**X**	**X (pre-dose)**	**X**	
11.5 hours	X	X	X		X	X	
12 hours							Pool 12-24hr
14 (bedtime)	X	X					
24 hours	X	X	X (predose, morning dose)				

1. See Section 7.5 for instructions for PK samples. Samples for analysis of metformin or liraglutide will be collected in EDTA containing tubes.
2. A single sample will be collected at each timepoint and quickly processed as described in Section 7.5.2. As some of the components of GSK2890457 are unstable in plasma, the blood for GSK2890457 PK needs to be collected in special tubes containing Potassium Oxalate/sodium fluoride and the blood quickly processed as described in Section 7.5.
3. Urine will be collected at the various intervals and the volume will be determined. A 100mL aliquot from each time interval will be used for the investigation of GSK2890457 metabolites.
4. Subjects should make their best effort to consume dose and meal in the allotted time; if unable to complete in time, this will not be considered a protocol violation.

Section 7.3.5. Clinical Laboratory Assessments

ADDED TEXT

Additional tests conducted at Screening
**C-peptide (Parts B and C)**

Section 7.6.1.3. Telemonitoring of Weight and CBG

ADDED TEXT

Subjects will be provided with a specific scale, glucometer and strips, and a telemonitoring unit that will transmit weight and glucose data to a central database. Each Investigator will be able to review data in real time for subjects enrolled at his site. Subjects will be asked to weigh themselves unclothed each morning before breakfast, and to collect a fasting glucose sample **and a pre-dinner glucose sample**, which will need to be downloaded to the telemonitoring device at least every other day.

Section 7.6.1.4. Fecal Microbiome Analysis

PREVIOUS TEXT

Using a 15mL conical vial with spoon attached, collect 3 spoons of each stool sample and transfer into a pre-labelled Stool Collection Tube (which contains 24mL of Stool DNA Stabilizer). The sample is mixed by shaking the tube for a short duration. After collection, tubes should be immediately stored frozen at -20°C prior to shipment.

REVISED TEXT

~~**Using a 15mL conical vial with spoon attached, c**~~ **C**ollect 3 spoons of each stool sample and transfer into a pre-labelled Stool Collection Tube **provided by GSK**~~**(which contains 24mL of Stool DNA Stabilizer). The sample is mixed by shaking the tube for a short duration.**~~ After collection, **immediately place** tubes **in freezer and store** ~~**should be immediately stored**~~ frozen at -20°C prior to shipment.

Section 10.2 Subject Withdrawal Criteria

ADDED TEXT

Subjects who are unable or unwilling to demonstrate 80% compliance with the telemonitoring requirements during the run-in “procedures familiarization” period will be withdrawn from the study and will not proceed to the Stabilization Period. **Subjects not using the telemonitoring glucose and weight equipment appropriately will be counselled on correct use, and technical issues will be addressed, if necessary. If poor compliance continues and does not improve with counselling, this may result in withdrawal from the study, after consultation with the GSK Medical Monitor.**

AMENDMENT 1

Where the Amendment Applies

This amendment applies to all sites.

Summary of Amendment Changes with Rationale

This amendment changes (i) the blood glucose definition of hypoglycaemia in Parts B and C to <70 mg/dL, (ii) adds blood glucose measurement pre-dinner in Parts B and C, (iii) clarifies acceptable surgical sterilization procedures, (iv) clarifies procedures to be followed if a pregnancy occurs during the study; (v) provides more flexibility in the calorie content of standardized breakfast meals, (vi) clarifies the procedures that are required to re-screen subjects who have fulfilled inclusion/exclusion requirements at an earlier visit, (vii) adds more information on the preparation of GSK2890457 and how to handle spills, (viii) adds the potential risk of upper respiratory and ocular irritation from powders in GSK2890457, (ix) excludes subjects with significant hypoglycaemia unawareness, (x) adds conditional wording relating to deuterated creatine procedures in case deuterated creatine is not available, and (xi) corrects minor inconsistencies and typographical and omission errors.

List of Specific Changes

Trademark Information

ADDED TEXT

Trademarks of the GlaxoSmithKline group of companies
RELENZA

Trademarks not owned by the GlaxoSmithKline group of companies
Chiron RIBA
Glucophage
InForm
Kaopectate
MiO
Pepto-Bismol
SAS
**Tamiflu**
Victoza
WinNonlin

Section 1.1 Background, Paragraph 3

PREVIOUS TEXT

...In this study, a new exploratory method will be evaluated against the DEXA method of measuring skeletal muscle mass....

REVISED TEXT

...In this study, a new exploratory method **may** be evaluated against the DEXA method of measuring skeletal muscle mass....

Section 1.3 Study Rationale, Final Paragraph

ADDED TEXT

An exploratory deuterated (non-radioactive, stable-label) creatine method for measuring skeletal muscle mass will be assessed in Parts B and C **if deuterated creatine is available by the start time of the Treatment period**.

Section 1.3.1 Dosing Rationale

ADDED TEXT

See Investigators Brochure [**GlaxoSmithKline Document Number 2012N141885_00**] for further details.

Section 1.4.1 Potential Risks of GSK2890457 and Their Mitigation, Paragraph 1

ADDED TEXT

Additional details relating to prior use of the individual components can be found in the Investigator's Brochure [**GlaxoSmithKline Document Number 2012N141885_00**].

Section 1.4.1 Potential Risks of GSK2890457 and Their Mitigation, Table 1

ADDED TEXT

Potential Risk	Background	Mitigation
Hypoglycemia	In animal efficacy studies, GSK2890457 has reduced elevated glucose levels to normal when used in combination with a GLP-1 mimetic – no hypoglycaemia has been observed. In these studies, insulin levels were either significantly reduced or showed non-significant elevations. This is consistent with the potential mechanisms of action of GSK2890457 which are believed to be effective only when glucose levels are elevated. Nevertheless, a risk of hypoglycemia in T2D subjects cannot be excluded when co-dosed with other anti-diabetic medications.	Subjects with T2D will monitor fasting **and pre-dinner** capillary blood glucose (CBG) values daily using a telemonitored glucometer, and will be advised to report any symptoms of hypoglycemia promptly.
**Contact exposure and inhalation of fine powder**	**The powders in this formulation of GSK2890457 may cause upper respiratory tract and ocular irritation in subjects who are sensitive to environmental dusts or allergens.**	**Exclude subjects with a past history of (i) sensitivity to environmental dusts or allergens, (ii) asthma or (iii) inhaler use.**

Section 1.4.2 Potential Risks of Liraglutide, Alone and Co-Administered with GSK2890457 and Their Mitigation, Table 2

ADDED TEXT

Potential Risk	Liraglutide	Liraglutide + GSK2890457	Mitigation
Hypoglycemia	In patients with T2D, hypoglycemia has been associated with administration of liraglutide when given in conjunction with other agents, such as insulin and insulin secretagogues such as sulphonylureas that have an intrinsic hypoglycemic risk.<br><br>Experience with liraglutide indicates that the risk of hypoglycemia is very low when it is used as monotherapy in T2D patients. [VICTOZA, 2012 Prescribing Information]	As GSK2890457 does not produce hypoglycemia in animal models and glucose improvement is observed in the absence of elevation of circulating insulin levels, it is unlikely that GSK2890457 will augment the very low risk of hypoglycemia seen with liraglutide.<br><br>Nevertheless, careful monitoring for hypoglycemia is advisable because it may occur when caloric intake is reduced,	**--Exclude subjects with hypoglycemic unawareness**<br><br>--Fasting **and pre-dinner** CBG will be monitored daily using a telemonitored device while subjects are at home.<br><br>--Subjects will be closely monitored for signs and symptoms of hypoglycemia and will be treated appropriately.<br><br>--Ad hoc capillary glucose measurements will be used to check blood glucose if a subject has symptoms of hypoglycemia **or is otherwise concerned about their blood glucose level**.<br><br>--T2D subjects clearly experiencing episodes of symptomatic hypoglycemia or episodes of significant confirmed hypoglycemia regardless of symptoms should have the dose of liraglutide and/or GSK2890457 reduced as indicated in Section 4.6.1.<br><br>-- If symptoms of hypoglycemia are reported, the Investigator will discuss with the GSK Medical Monitor if it is appropriate to modify doses (e.g., reduce the liraglutide dose to 1.2mg). A subject should be withdrawn if episodes of clinically significant hypoglycemia persist even when the dose of liraglutide has been reduced to 1.2mg.<br><br>-- Subjects may continue if the episodes of hypoglycemia are not considered clinically significant (e.g., because they are a physiological response to fasting).

PREVIOUS TEXT

Potential Risk	Liraglutide	Liraglutide + GSK2890457	Mitigation
Medullary Thyroid Carcinoma	Liraglutide may be associated with an increased risk of developing medullary thyroid carcinoma. This association is based on chronic exposure studies in rodents and the relevance to humans is unclear.	As the components of GSK2890457 are food ingredients and there were no thyroid lesions in the 6 week rat toxicity study, it is not anticipated that co-dosing for 6 weeks will materially increase the risk to subjects.	--Subjects with a personal or family history of medullary carcinoma of the thyroid or multiple endocrine neoplasia type 2, or with plasma calcitonin levels at screening ≥ 50pg/mL are excluded from the study.<br><br>- A careful thyroid physical examination will be conducted at screening and before the start of the 6-week Treatment Period. A subject should be excluded if an abnormal thyroid mass is detected.

REVISED TEXT

Potential Risk	Liraglutide	Liraglutide + GSK2890457	Mitigation
Medullary Thyroid Carcinoma	Liraglutide may be associated with an increased risk of developing medullary thyroid carcinoma. This association is based on chronic exposure studies in rodents and the relevance to humans is unclear.	As the components of GSK2890457 are food ingredients and there were no thyroid lesions in the 6 week rat toxicity study, it is not anticipated that co-dosing for 6 weeks will materially increase the risk to subjects.	--Subjects with a personal or family history of medullary **or papillary** carcinoma of the thyroid or multiple endocrine neoplasia type 2, or with plasma calcitonin levels at screening **>50pg/mL** are excluded from the study.<br><br>- A careful thyroid physical examination will be conducted at screening and before the start of the 6-week Treatment Period. A subject should be excluded if an abnormal thyroid mass is detected.

Section 1.4.3 Potential Risks of Co-administration of Metformin and GSK2890457 and Their Mitigation, Table 3

ADDED TEXT

Potential Risk	Metformin	Metformin + GSK2890457	Mitigation
Hypoglycemia	While hypoglycemia has been reported in ≥1.0% to ≤5.0% of metformin patients (and more commonly than placebo) in clinical studies, the risk is very low when it is used as monotherapy in T2D patients.<br><br>This is because metformin improves fasting and prandial glucose levels, without increasing circulating insulin levels.	As GSK2890457 does not produce hypoglycemia in animal models and glucose improvement is observed in the absence of elevation of circulating insulin levels, it is unlikely that GSK2890457 will augment the very low risk of hypoglycemia seen with metformin.<br><br>Nevertheless, careful monitoring for hypoglycemia is advisable because it may occur when caloric intake is reduced.	**--Exclude subjects with hypoglycemic unawareness**<br><br>--Fasting **and pre-dinner** CBG will be monitored daily using a telemonitored device while subjects are at home.<br><br>--Subjects will be closely monitored for signs and symptoms of hypoglycemia and will be treated appropriately.<br><br>--Ad hoc CBG measurements to check status if a subject has symptoms of hypoglycemia **or is otherwise concerned about their blood glucose level.**<br><br>--Subjects clearly experiencing episodes of symptomatic hypoglycemia or episodes of significant confirmed hypoglycemia regardless of symptoms should have the dose of GSK2890457 and/or metformin reduced as indicated in Section 4.6.1.<br><br>If symptoms of hypoglycemia are reported, the Investigator can discuss with the GSK Medical Monitor if it is appropriate to modify the doses of study medication. A subject should be withdrawn if episodes of clinically-significant hypoglycemia persist even after dose adjustment.<br><br>Subjects may continue if the episodes of hypoglycemia are not considered clinically significant (e.g., because they are a physiological response to fasting).

Section 2.3 Exploratory Objectives

ADDED TEXT

- To explore biomarkers of obesity, T2D and/or related metabolic diseases **and/or inflammatory diseases or conditions**, and biomarkers of GSK2890457 pharmacodynamics and/or safety when co-administered to subjects with T2D for six weeks with liraglutide (Part B) or metformin (Part C).
- To evaluate a novel method of measuring total skeletal muscle mass using deuterated creatine **if it is available for dosing** (Parts B and C).

Section 3.3 Exploratory Endpoints

PREVIOUS TEXT

- DEXA measurement of total lean mass and appendicular on Day 42 as compared to Day -1(Parts B and C).
- Exploratory analyses relating to biomarkers of obesity, T2D and/or related metabolic diseases, and biomarkers of GSK2890457 pharmacodynamics or safety may be performed using (i) small molecular weight metabolites, (ii) blood polypeptide analytes, and (iii) novel biomarkers derived from peptidomic, lipidomic and metabolomic analysis of blood and/or urine, as data permit. Exploratory analyses may be conducted to biomarkers of adiposity, inflammation, insulin sensitivity, beta cell function, gut peptide secretion and other exploratory biomarkers, as data permit.

REVISED TEXT

- DEXA measurement of total lean mass and appendicular on Day 4**1** as compared to Day **-2**(Parts B and C).
- Exploratory analyses relating to biomarkers of obesity, T2D and/or related metabolic diseases **and/or inflammatory diseases or conditions**, and biomarkers of GSK2890457 pharmacodynamics or safety may be performed using (i) small molecular weight metabolites, (ii) blood polypeptide analytes, and (iii) novel biomarkers derived from peptidomic, lipidomic and metabolomic analysis of blood and/or urine, as data permit. Exploratory analyses may be conducted **with** biomarkers of adiposity, inflammation, insulin sensitivity, beta cell function, gut peptide secretion and other exploratory biomarkers, as data permit.

Section 4.1 Part A, Bullets 1 and 2

ADDED TEXT

- Healthy subjects who fulfil the Screening requirements will be randomized to receive either GSK2890457 or placebo for 6 weeks, to evaluate safety and tolerability. **Note: subjects who have fulfilled inclusion/exclusion requirements, but who do not start the Treatment period within 28 days of Screening may be re-screened using the procedures outline in Section 7.3.6.**

- The subjects will be instructed how to mix **a 5g dose of** the study medication and will take it under the supervision of an unblinded site staff member at dinner on Day 1, the time of randomization.

Section 4.1 Part A, Bullet 3

PREVIOUS TEXT

- On Days 1 and 42, subjects will come in to the unit fasting (nothing after midnight the night before) and receive a single 500mg IR tablet of metformin just before eating breakfast, and will stay in the clinical unit for 10h to allow collection of blood samples for analysis of metformin concentrations. On Day 42, the subjects will also take GSK2890457/placebo immediately before breakfast (the order will be: take metformin followed by GSK2890457 or placebo and then breakfast). On Day 1 and Day 42, GSK2890457 will be dosed immediately prior to dinner...

REVISED TEXT

- On Days 1 and 42, subjects will come in to the unit fasting (nothing after midnight the night before**; minimum 8-hour fast**) and receive a single 500mg IR tablet of metformin just before eating breakfast, and will stay in the clinical unit for 10h to allow collection of blood samples for analysis of metformin concentrations. **On Day 1, GSK2890457 will be dosed immediately prior to dinner.** On Day 42, the subjects will also take GSK2890457/placebo immediately before breakfast (the order will be: take metformin followed by GSK2890457 or placebo and then breakfast) **and then they will take the second dose**~~. On Day 1and Day 42, GSK2890457 will be dosed immediately prior to~~ **before** dinner...

Section 4.2.1 Run-in Period (1 week)

ADDED TEXT

- Remain on their usual dose of metformin **during the Run-in only**.
- Check fasting (**minimum 8-hour fast**) **and pre-dinner** CBG daily at home, and transmit values daily if possible, but at least every other day via the telemonitoring unit (glucometer, strips and telemonitoring unit will be provided).

Note: subjects who have fulfilled inclusion/exclusion requirements, but who do not start the Stabilisation period within 28 days of Screening may be re-screened using the procedures outlined in Section 7.3.6.

Section 4.2.2 Stabilization Period (12 weeks)

ADDED TEXT

- Check fasting **(minimum 8-hour fast) and pre-dinner** CBG daily at home, and transmit values daily if possible, but at least every other day via the telemonitoring unit (glucometer, strips and telemonitoring unit will be

provided). Subjects may measure their CBG at other times if they are concerned about potential hypoglycaemia **or their blood glucose levels. Note: Subjects do not need to bring their telemonitoring wireless hub into the unit for routine visits, only at the end of the study**.

Section 4.2.3 Treatment Period (6 weeks)

ADDED TEXT

- Check fasting **(minimum 8-hour fast) and pre-dinner** CBG daily at home, and transmit values daily if possible, but at least every other day via the telemonitoring unit (glucometer, strips and telemonitoring unit will be provided). Subjects may measure their CBG at other times if they are concerned about potential hypoglycaemia **or their blood glucose levels. Note: Subjects do not need to bring their telemonitoring wireless hub into the unit for routine visits, only at the end of the study.**

- On Day -2, subjects will enter the clinic and will be provided with a standardized dinner. DEXA evaluation of fat/lean body mass, baseline Food Frequency Questionnaire and GSRS will be completed. A dose of 30mg deuterated (non-radioactive) creatine will be administered to subjects in the afternoon about 2 hours after lunch **if it is available**.
- On Day 1, subjects will be instructed how to mix the study medication and then they will take it under the supervision of an unblinded site staff member prior to breakfast. Study medication will be dispensed at the time of randomization. Subjects will be discharged following final blood sampling and breakfast. A morning urine sample will also be taken before discharge to measure deuterated-creatine method analytes **if the deuterated creatine dose has been administered**.
- On Day 41, subjects will enter the clinic, and the Day -2 procedures will be repeated, with the exception that the Food Frequency Questionnaire will not be completed. A urine sample will also be taken prior to the second dose of deuterated-creatine to measure baseline deuterated-creatine method analytes if **the deuterated creatine has been dosed**. The second dose of **deuterated** creatine will be administered in the afternoon similar to Day **-**2 **if available**. GSK2890457 or placebo will be administered just prior to dinner...
- On Day 43, subjects will be discharged following final blood sampling and breakfast. A morning urine sample will also be taken before discharge to measure **deuterated** creatine method analytes similar to collection and time of day as on Day ~~-2~~ **1 if the deuterated creatine dose has been administered**

Section 4.3.1 Run-in Period (1 week)

DELETED TEXT

- Weigh themselves once daily at home fasted, first thing in the morning, using a telemonitoring scale (provided). ~~**When weighing at home, subjects should be**~~

~~**nude. When subjects are in-house, they will be weighed on a designated scale for thise study; they do not need to bring their home telemonitored scale in to the unit for routine visits, only at the end of the study. When weighed in the unit, subjects should be weighed without shoes in light clothing. The same scale should be used throughout the study for all subjects at every designated study visit.**~~

ADDED TEXT

- Check fasting **(minimum 8-hour fast) and pre-dinner** CBG daily at home, and transmit values daily if possible, but at least every other day via the telemonitoring unit (glucometer, strips and telemonitoring unit will be provided)...

Note: subjects who have fulfilled inclusion/exclusion requirements, but who do not start the Stabilisation period within 28 days of Screening may be re-screened using the procedures outline in Section 7.3.6.

Section 4.3.2 Stabilization Period (4 weeks)

ADDED TEXT

- Check fasting **(minimum 8-hour fast) and pre-dinner** CBG daily at home, and transmit values daily if possible, but at least every other day via the telemonitoring unit (glucometer, strips and telemonitoring unit will be provided). Subjects may measure their CBG at other times if they are concerned about potential hypoglycaemia **or their blood glucose levels**. **Note: Subjects do not need to bring their telemonitoring wireless hub into the unit for routine visits, only at the end of the study.**

Section 4.3.3 Treatment Period (6 weeks)

PREVIOUS TEXT

- Check fasting blood glucose daily at home, and transmit values daily if possible, but at least every other day via the telemonitoring unit (glucometer, strips and telemonitoring unit will be provided). Subjects may measure their capillary blood glucose at other times if they are concerned about potential...
- On Day 41, subjects will enter the clinic, and the Day -2 procedures will be repeated, with the exception that the Food Frequency Questionnaire will not be completed. A urine sample will also be taken prior to the second dose of deuterated-creatine to measure baseline d-3-creatine method analytes. The second dose of deuterated creatine will be administered in the afternoon similar to Day -2...
- On Day 43, subjects will be discharged following final blood sampling and breakfast. A morning urine sample will also be taken before discharge to measure deuterated creatine method analytes similar to collection and time of day as on Day 1.

REVISED TEXT

- Check fasting **(minimum 8-hour fast) and pre-dinner CBG** daily at home, and transmit values daily if possible, but at least every other day via the telemonitoring unit (glucometer, strips and telemonitoring unit will be provided). Subjects may measure their capillary blood glucose at other times if they are concerned about potential hypoglycaemia **or their blood glucose levels**. **Note: Subjects do not need to bring their telemonitoring wireless hub into the unit for routine visits, only at the end of the study**.

- **Subjects will receive guidance on Mindful Eating.**
- On Day 41, subjects will enter the clinic, and the Day -2 procedures will be repeated, with the exception that the Food Frequency Questionnaire will not be completed. If the deuterated creatine has been dosed, a urine sample will also be taken prior to the second dose of deuterated-creatine to measure baseline

deuterated-creatine method analytes. The second dose of deuterated creatine will be administered in the afternoon similar to Day -2 if it is available...

- On Day 43, subjects will be discharged following final blood sampling and breakfast. **If the deuterated creatine dose has been administered, a** morning urine sample will also be taken before discharge to measure deuterated creatine method analytes similar to collection and time of day as on Day 1.

Section 4.3.3 Treatment Period (6 weeks)

ADDED TEXT

- On Day -2, subjects will enter the clinic and will be provided with a standardized dinner. DEXA evaluation of fat/lean body mass, baseline Food Frequency Questionnaire and GSRS will be completed. A dose of 30mg deuterated (non-radioactive) creatine will be administered to subjects in the afternoon about 2 hours after lunch **if it is available**.
- On Day 1... A morning urine sample will also be taken before discharge to measure deuterated creatine method analytes (e.g., creatine, creatinine and deuterated creatinine) **if the dose of deuterated creatine has been administered**.
- On Day 41, subjects will enter the clinic, and the Day -2 procedures will be repeated, with the exception that the Food Frequency Questionnaire will not be completed. **If the deuterated creatine has been dosed**, a urine sample will also be taken prior to the second dose of deuterated-creatine to measure baseline deuterated-creatine method analytes. The second dose of **deuterated** creatine will be administered in the afternoon similar to Day -2 **if it is available**.
- On Day 43, subjects will be discharged following final blood sampling and breakfast. **If the deuterated creatine dose has been administered**, a morning urine sample will also be taken before discharge to measure deuterated creatine method analytes similar to collection and time of day as on Day 1.

Section 4.4.1 GSK2890457 Dosage and Administration (Parts A-C), First Bullet

ADDED TEXT

- The planned dosing regimen for Parts A through C is shown in the diagram below (in Part A the Day 1 dose is 5g)...

Section 4.4.1 GSK2890457 Dosage and Administration (Parts A-C), Third Bullet

ADDED TEXT

- All subjects will begin taking a 15g daily dose of either GSK2890457 or placebo on Day 1, consuming 5g just prior to breakfast and 10g just prior to dinner **(except for Part A as noted above)**...

Section 4.4.1 GSK2890457 Dosage and Administration (Parts A-C), Note 1

PREVIOUS TEXT

- **Important Note 1:** GSK2890457 and placebo are packaged identically, but differences are discernible when the powders are directly compared, both when dry and when mixed with water. For this reason, a specified unblinded member of the study site staff must demonstrate appropriate mixing of study medication with water according to instructions in Appendix 5: Directions for Taking Study Medicine and supervise drug administration in the clinic. (One dose on Day 1 for Parts A, B and C, one dose on Day 41 for Parts B and C, and two doses on Day 42 for Parts A, B, and C.)

REVISED TEXT

- **Important Note 1:** GSK2890457 and placebo are packaged identically, but differences are discernible when the powders are directly compared, both when dry and when mixed with water. For this reason, a specified unblinded member of the study site staff must demonstrate appropriate mixing of study medication with water according to instructions in Appendix 5: Directions for Taking Study Medicine and supervise drug administration in the clinic. **When subjects are dosed at the study site, they will receive study treatment directly from the designated unblinded study staff member.** ~~**(One dose on Day 1 for Parts A, B and C, one dose on Day 41 for Parts B and C, and two doses on Day 42 for Parts A, B, and C.)**~~

Section 4.4.1 GSK2890457 Dosage and Administration (Parts A-C), Note 2

ADDED TEXT

- **Important Note 2:** Some subjects may find it difficult to take GSK2890457, especially at the highest dose of 40g. To improve or maintain compliance with dosing, this protocol allows the Investigator some flexibility, in consultation with the GSK Medical Monitor, to modify the way a subject takes the components of GSK2890457 or placebo, during the 6-week Treatment Period. In all cases, once sachets **are mixed with water they must be consumed soon after mixing. Subjects should be encouraged to remain well hydrated by drinking water, b**~~**ut not sugar containing sodas, flavored drinks or fruit juices.**~~

Section 4.5 Metformin (Part C) [UPDATED HEADER]

Section 4.5 Investigational Product and Other Study Treatment Dosage/Administration

PREVIOUS TEXT

	Study Treatment				
**Product name:**	**GSK2890457**	**Placebo**	**Liraglutide**	**Metformin**	**Deuterated Creatine**
**Route/ Administration/ Duration:**	Oral<br>6 weeks dosing (Treatment Period)	Oral<br>6 weeks dosing (Treatment Period)	Subcutaneous injection<br>18 weeks dosing (Stabilization and Treatment Periods, Part C only)	Oral<br>Part A: Single doses on Day 1 and Day 42:<br><br>Part B: Subject continues usual metformin dose through Run-in, and resumes after Treatment Period<br><br>Part C: Subject continues usual metformin dose throughout study	Oral<br>A single dose on Day -2 and on Day 41<br>Parts B and C only
**Manufacturer/ source of procurement:**	GSK	GSK	Site will provide Victoza (liraglutide) and will provide subject with the Victoza 'Medication Guide'	Part A: Site will supply metformin<br><br>Parts B & C: Subject will take their own supplies.	GSK

REVISED TEXT

	Study Treatment				
**Product name:**	**GSK2890457**	**Placebo**	**Liraglutide**	**Metformin**	**Deuterated Creatine (if available)**
**Route/ Administration/ Duration:**	Oral<br>6 weeks dosing (Treatment Period)	Oral<br>6 weeks dosing (Treatment Period)	Subcutaneous injection<br>18 weeks dosing (Stabilization and Treatment Periods, Part **B** only)	Oral<br>Part A: Single doses on Day 1 and Day 42:<br><br>Part B: Subject continues usual metformin dose through Run-in, and resumes after Treatment Period<br><br>Part C: Subject continues usual metformin dose throughout study	Oral<br>A single dose on Day -2 and on Day 41 Parts B and C only
**Manufacturer/ source of procurement:**	GSK	GSK	Site will provide Victoza (liraglutide) **and needles**, and will provide subject with the Victoza 'Medication Guide'	Part A: Site will supply metformin<br><br>Parts B & C: Subject will take their own supplies.	GSK

Section 4.6.1.2 QTc Withdrawal Criteria

ADDED TEXT

Withdrawal decisions are to be based on an average QTc**F** value of triplicate ECGs. If an ECG demonstrates a prolonged QT interval, obtain 2 more ECGs over a brief period, and then use the averaged QTc**F** values of the 3 ECGs to determine whether the subject should be discontinued from the study.

Section 4.6.1.4 Dose Adjustment/Stopping Safety Criteria Based on Blood Glucose Monitoring (Part B and C)

PREVIOUS TEXT

Subjects who complete successfully the screening procedures prior to dosing in Parts B and C must check fasting CBG at least once daily (fasting pre-breakfast), and at any time symptoms of hypoglycemia or hyperglycemia (e.g., polyuria, polydipsia) are experienced must transmit the values for 'real-time' monitoring by the Investigator and GSK Medical Monitor.

Hypoglycemia is defined as symptoms consistent with hypoglycemia (e.g. dizziness, light-headedness, shakiness) which are confirmed by glucometer measurement of CBG or plasma glucose value of <50mg/dL (if possible, CBG values should be confirmed with a laboratory measurement). In situations when no glucose sample can be measured at the time of the event, the investigator may, at his or her discretion, characterize an event as 'hypoglycemia' based on reported signs and symptoms alone.

REVISED TEXT

Subjects who complete successfully the screening procedures prior to dosing in Parts B and C must check fasting (pre-breakfast**; minimum 8-hour fast**) **and pre-dinner CBG**, and at any time symptoms of hypoglycemia or hyperglycemia (e.g., polyuria, polydipsia) are experienced **or they are concerned about their blood glucose level. They** must transmit the values for 'real-time' monitoring by the Investigator and GSK Medical Monitor.

Subjects enrolled in Parts B and C will be given written instructions on how to recognize and manage hypoglycaemia.

Hypoglycemia is defined as symptoms consistent with hypoglycemia (e.g. dizziness, light-headedness, shakiness) which are confirmed by glucometer measurement of CBG or plasma glucose value of <50mg/dL **for Part A or <70mg/dL for Parts B and C** (if possible, CBG values should be confirmed with a laboratory measurement). In situations when no glucose sample can be measured at the time of the event, the investigator may, at his or her discretion, characterize an event as 'hypoglycemia' based on reported signs and symptoms alone. **Note: healthy subjects may have asymptomatic blood glucose values <70mg/dL as a physiological response to altered food intake (e.g., fasting).**

Section 4.6.1.4 Dose Adjustment/Stopping Safety Criteria Based on Blood Glucose Monitoring (Part B and C), Table 4

PREVIOUS TEXT

Category	Description
Mild	An event that is easily tolerated by the subject, causing minimal discomfort and not interfering with normal everyday activities. This includes events of asymptomatic 'biochemical' hypoglycemia when CBG or plasma glucose is <50mg/dL. Note: physiological asymptomatic hypoglycemia can be observed in healthy subjects as part of the response to fasting.

REVISED TEXT

Category	Description
Mild	An event that is easily tolerated by the subject, causing minimal discomfort and not interfering with normal everyday activities. This includes events of asymptomatic 'biochemical' hypoglycemia when CBG or plasma glucose is <50mg/dL **(Part A) or <70mg/dL (Parts B and C).** ~~**Note: physiological asymptomatic hypoglycemia can be observed in healthy subjects as part of the response to fasting.**~~

Section 4.6.1.4.1 Part A (Healthy Subjects)

PREVIOUS TEXT

- In the unlikely event that the healthy subjects report clinically significant symptoms of hypoglycemia or plasma glucose values are <50mg/dL on 2 occasions separated by a few hours, the dose of GK2890457 may be reduced after consultation with the GSK Medical Monitor.

REVISED TEXT

- In the unlikely event that the healthy subjects report clinically significant symptoms of hypoglycemia or ~~**plasma glucose**~~ **CBG** values are <50mg/dL on 2 occasions separated by a few hours, the dose of GK2890457 may be reduced after consultation with the GSK Medical Monitor.

Section 4.6.1.4.2 Part B (Liraglutide Add-on)

PREVIOUS TEXT

Stabilization Period: In the unusual event that a T2D subject randomized to liraglutide experiences symptoms/signs of hypoglycemia confirmed by a CBG value or laboratory plasma glucose value <50mg/dL, the dose of liraglutide may be reduced from 1.8mg to 1.2mg.

Treatment Period: In the unlikely event that a T2D subject who has progressed uneventfully through the Stabilization Period experiences symptoms/signs of hypoglycemia confirmed by a capillary glucose value or laboratory plasma glucose value <50mg/dL, reduction of the dose of liraglutide or GSK2890457 or discontinuation of further dosing should be considered for the affected subject, in consultation with the GSK Medical Monitor.

REVISED TEXT

Stabilization Period: In the unusual event that a T2D subject randomized to liraglutide experiences symptoms/signs of hypoglycemia confirmed by a CBG value or laboratory plasma glucose value <**70**mg/dL, the dose of liraglutide may be reduced from 1.8mg to 1.2mg…

Treatment Period: In the unlikely event that a T2D subject who has progressed uneventfully through the Stabilization Period experiences symptoms/signs of hypoglycemia confirmed by a capillary glucose value or laboratory plasma glucose value <**70**mg/dL, reduction of the dose of liraglutide or GSK2890457 or discontinuation of further dosing should be considered for the affected subject, in consultation with the GSK Medical Monitor.

Section 4.6.1.4.3 Part C (Metformin Add-on)

PREVIOUS TEXT

If hypoglycemia occurs, a reduction of the dose of GSK2890457 and/or metformin or discontinuation of further dosing should be considered for the affected subject, in consultation with the GSK Medical Monitor.

REVISED TEXT

If **a subject experiences symptoms/signs of hypoglycemia confirmed by a capillary glucose value or laboratory plasma glucose value <70mg/dL**, a reduction of the dose of GSK2890457 and/or metformin or discontinuation of further dosing should be considered for the affected subject, in consultation with the GSK Medical Monitor.

Section 4.7.1 Part A Time and Events Table – Healthy Subjects

PREVIOUS TEXT

	Screening	Day 1	Day 4 (± 1 Day)	Day 7 (± 1 Day)	Day 14 (± 1 Day)	Day 21 (± 1 Day)	Day 28 (± 1 Day)	Day 35 (± 1 Day)	Day 42 (± 1 Day)	Follow-up Visit
**Visit Timing (relative to Day 1)**	**Maximum of -28 days pre-Run-in**									**Day 56 (± 1 Day)**
Clinic Visit	X	X		X	X		X		X	X
Phone Contact			X			X		X		
Informed Consent	X									
Demographics	X									
Complete Physical	X									
Brief Physical										X
Medical/medication/drug/alcohol history	X									
Vital Signs	X	X (predose)		X	X		X		X	X
12-Lead ECG Single	X								X	X
12-Lead ECG Triplicate		X (predose)								
Body Weight (in clinic)	X	X		X	X		X		X	X
Urine drug/alcohol screen	X	X (predose)		X	X		X		X	
Hematology/Chemistry/Urinalysis	X	X (pre- metformin)		X	X		X		X	X
HIV, Hep B and Hep C, TSH Screen	X									
Waist circumference		X							X	
GI Symptoms Rating Scale Questionnaire (GSRS)		X (predose)		X	X				X	
Metformin PK		X							X	
Fasting Glucose		X (predose)		X	X		X		X	
Dispense/Instruct: Study Medication		X		X	X		X			
Randomization		X (predose)								
Twice-Daily Dosing GSK2890457			---X---							
Evening Dose GSK2890457		X								
Metformin Single 500mg dose		X							X	

	Screening	Day 1	Day 4 (± 1 Day)	Day 7 (± 1 Day)	Day 14 (± 1 Day)	Day 21 (± 1 Day)	Day 28 (± 1 Day)	Day 35 (± 1 Day)	Day 42 (± 1 Day)	Follow-up Visit
**Visit Timing (relative to Day 1)**	**Maximum of -28 days pre-Run-in**									**Day 56 (± 1 Day)**
Identical Meals		X							X	
Concomitant Medication Review	X	X	X	X	X	X	X	X	X	X
AE Assessment		X	X	X	X	X	X	X	X	X

REVISED TEXT

	Screening	Day 1	Day 4 (± 1 Day)	Day 7 (± 1 Day)	Day 14 (± 1 Day)	Day 21 (± 1 Day)	Day 28 (± 1 Day)	Day 35 (± 1 Day)	Day 42 (± 1 Day)	Follow-up Visit
**Visit Timing (relative to Day 1)**	**Maximum of -28 days ~~pre Run-in~~**									**Day 56 (± 1 Day)**
Clinic Visit	X	X		X	X		X		X	X
Phone Contact			X			X		X		
Informed Consent	X									
Demographics	X									
Complete Physical	X									
Brief Physical										X
Medical/medication/drug/alcohol history	X									
Vital Signs	X	X **(pre-metformin)**		X	X		X		X	X
12-Lead ECG Single	X								X	X
12-Lead ECG Triplicate		X **(pre-metformin)**								
**Height**	**X**									
Body Weight (in clinic)	X	X		X	X		X		X	X
Urine drug/alcohol screen	X	X **(pre-metformin)**		X	X		X		X	
**Urine beta-HCG (women)**		**X (pre-metformin**								
Hematology/Chemistry/Urinalysis	X	X (pre-metformin)		X	X		X		X	X
HIV, Hep B and Hep C, TSH Screen	X									
Waist circumference		X							X	
GI Symptoms Rating Scale Questionnaire (GSRS)		X **(pre-metformin)**		X	X				X	
Metformin PK		X							X	
Fasting Glucose **(minimum 8-hour fast)**		X **(pre-metformin)**		X	X		X		X	

	Screening	Day 1	Day 4 (± 1 Day)	Day 7 (± 1 Day)	Day 14 (± 1 Day)	Day 21 (± 1 Day)	Day 28 (± 1 Day)	Day 35 (± 1 Day)	Day 42 (± 1 Day)	Follow-up Visit
**Visit Timing (relative to Day 1)**	**Maximum of -28 days ~~pre Run-in~~**									**Day 56 (± 1 Day)**
Dispense/Instruct: Study Medication		X		X	X		X			
Randomization		X (pre-metformin)								
Twice-Daily Dosing GSK2890457			-----------------------	-----------------------	-----------------------	----------X-----------	-----------------------	-----------------------	-----------------------	
Evening Dose GSK2890457		X								
Metformin Single 500mg dose		X							X	
Identical Meals		X							X	
Concomitant Medication Review	X	X	X	X	X	X	X	X	X	X
AE Assessment		X	X	X	X	X	X	X	X	X

Section 4.7.2 Part B Time and Events Table: Screening, 1-Week Telemonitoring Run-in and 12-Week Stabilization

ADDED TEXT

	Screening	Telemonitoring "Procedures Familiarization" Run-In	Stabilization					
**Visit Timing**	**Maximum of -28 days pre-Stabilization**	**13 weeks prior to Treatment Period**	**Week 1 of Stabilization Period**	**Week 2 of Stabilization Period**	**Week 3 of Stabilization Period**	**Week 5 of Stabilization Period**	**Week 7 of Stabilization Period**	**Week 10 of Stabilization Period**
Clinic Visit	X	X	X		X[1]		X	X
Phone Contact				X		X		
Informed Consent	X							
Demographics	X							
Complete Physical (including detailed psychiatric history)	X							

	Screening	Telemonitoring "Procedures Familiarization" Run-In	Stabilization					
**Visit Timing**	**Maximum of -28 days pre-Stabilization**	**13 weeks prior to Treatment Period**	**Week 1 of Stabilization Period**	**Week 2 of Stabilization Period**	**Week 3 of Stabilization Period**	**Week 5 of Stabilization Period**	**Week 7 of Stabilization Period**	**Week 10 of Stabilization Period**
Brief Physical (including thyroid examination)			X		X		X	X
Suicidality Monitoring with C-SSRS	X		X				X	
Medical/medication/drug/alcohol history	X							
Vital Signs	X		X				X	X
12-Lead ECG Single	X							
**Height**	**X**							
Body Weight (in clinic)	X		X		X		X	X
Waist circumference			X		X		X	X
Urine drug/alcohol screen	X		X		X		X	X
Hematology/Chemistry/Urinalysis (urine albumin and creatinine only at Screening)	X		X					
HbA1c	X		X					
Fasting Glucose and Insulin **(minimum 8-hour fast)**	X		X				X	
HIV, Hep B and Hep C Screen, TSH	X							
Calcitonin	X							
Lipase and Amylase	X		X		X			X
Urine beta-HCG (women)			X					
Dispense/instruct: scale and glucometer		X						
Daily fasting **and pre-dinner** glucometer readings at home		---X---						
Daily fasting weight at home		---X---						
GI Symptom Rating Scale Questionnaire (GSRS)			X				X	

	Screening	Telemonitoring "Procedures Familiarization" Run-In	Stabilization					
**Visit Timing**	**Maximum of -28 days pre-Stabilization**	**13 weeks prior to Treatment Period**	**Week 1 of Stabilization Period**	**Week 2 of Stabilization Period**	**Week 3 of Stabilization Period**	**Week 5 of Stabilization Period**	**Week 7 of Stabilization Period**	**Week 10 of Stabilization Period**
Dispense/ Instruct/Reinforce: Mindful Eating Guide to Losing Weight			X		X		X	X
Dose Liraglutide			--X--					
Increase Liraglutide Dose				X	X			
Concomitant Medication Review	X	X	X	X	X	X	X	X
AE Assessment		X	X	X	X	X	X	X

1. At Week 3 of the Stabilization Period, a clinic visit will include a short review in unit, and dispensing liraglutide. Because the 1.8 pen lasts less than a month, subject re-supply needs to be managed closely.

Section 4.7.3 Part C Time and Events Table: Screening, 1-Week Telemonitoring Run-in and 4-Week Stabilization for Subjects Remaining on Metformin

ADDED TEXT

	Screening	Telemonitoring "Procedures Familiarization" Run-In	Stabilization	
**Visit Timing**	**Maximum of -28 days pre-Stabilization**	**5 weeks prior to Treatment Period**	**Week 1 of Stabilization Period**	**Week 3 of Stabilization Period**
Clinic Visit	X	X	X	
Phone Contact				X
Informed Consent	X			
Demographics	X			
Complete Physical	X			
Brief Physical				
Medical/medication/drug/alcohol history	X			

	Screening	Telemonitoring "Procedures Familiarization" Run-In	Stabilization	
**Visit Timing**	**Maximum of -28 days pre-Stabilization**	**5 weeks prior to Treatment Period**	**Week 1 of Stabilization Period**	**Week 3 of Stabilization Period**
Vital Signs	X		X	
12-Lead ECG Single	X			
**Height**	**X**			
Body Weight (in clinic)	X		X	
Waist circumference			X	
Urine drug/alcohol screen	X		X	
Hematology/Chemistry/Urinalysis (urine albumin and creatinine only at Screening)	X		X	
HbA1c	X		X	
Fasting Glucose and Insulin **(minimum 8-hour fast)**	X		X	
HIV, Hep B and Hep C Screen, TSH	X			
Urine beta-HCG (women)			X	
Dispense/instruct: scale and glucometer		X		
Daily fasting **and pre-dinner** glucometer readings at home		--X--		
Daily fasting weight at home		--X--		
GI Symptom Rating Scale (GSRS)			X	
Dispense/ Instruct/Reinforce: Mindful Eating Guide to Losing Weight			X	X
Dose Metformin		--X--		
Concomitant Medication Review	X	X	X	X
AE Assessment			X	X

Section 4.7.4 Parts B and C Time and Events Table Day -2 through Study End

PREVIOUS TEXT

Procedure	Day -2	Day -1	Day 1	Day 4	Day 7	Day 14	Day 21	Day 28	Day 35	Day 41	Day 42	Day 43	Follow-up
**Visit Timing (relative to Day 1)**													**Day 56**
Clinic Visit	--In-clinic---				X	X		X		--In-clinic---			X
Phone Contact				X			X[1]		X[1]				
Brief Physical (including thyroid examination in Part B only)	X									X			X
Suicidality monitoring with C-SSRS (Part B only)	X									X			X
Vital Signs		X	X		X	X		X			X	X	X
12-Lead ECG Triplicate		X											
12-Lead ECG Single											X		X
Body Weight (in clinic)		X	X		X	X		X			X	X	X
Waist circumference		X			X	X		X			X		
Urine drug/alcohol screen	X				X	X		X		X			
Hematology/Chemistry/Urinalysis		X			X	X		X			X		X
HbA1c		X									X		
T3/T4/TSH		X									X		
Urine beta-HCG (women)	X												
Liraglutide subjects: continue liraglutide (Part B)	--X------------------------------												
Restart Metformin (Part B)												X	
Metformin subjects: continue metformin (Part C)	--X--												
Daily fasting glucometer readings at home	--X--												
Daily fasting weight at home	--X--												
Standardized evening meal	X									X			
Standardized breakfast, lunch and dinner		X									X		
Food Frequency Questionnaire	X												
GI Symptom Questionnaire (GSRS)	X				X	X		X		X			
Hunger Questionnaire		X									X		
Background Questions/ End of Study Questions	X									X			
NAFLD Score		X									X		
CRP, Lipid Panel		X									X		

Procedure	Day -2	Day -1	Day 1	Day 4	Day 7	Day 14	Day 21	Day 28	Day 35	Day 41	Day 42	Day 43	Follow-up
Deuterated creatine dose (in afternoon)	X									X			
Morning urine for deuterated-creatine analytes			X									X	
25 Hydroxy Vitamin D		X											
Lipase and Amylase (Part B only)		X									X		X
PK Blood sampling		X									X		
Glucose/Insulin Blood Sampling (See Section 4.7.6)		X									X		
Fasting Biomarker Samples		X									X		
Drug Metabolism10 (DM10) Sampling – blood		X									X		
24-hour urine for assessment of GSK2890457 metabolites		X									X		
DEXA	X									X			
Fecal Sampling	X									X			
Dispense/Instruct: Study Medication		X			X	X		X					
Dispense/ Instruct/Reinforce: Mindful Eating Guide to losing weight		X				X		X					
Randomization			X										
Twice-Daily Dosing of Study Medication			----------------------------X----------------------------										
Concomitant Medication Review	X				X	X		X		X			X
AE Assessment	X	X	X	X	X	X		X		X	X	X	X
Subject Returns Telemonitoring Equipment													X

1. If subjects have difficulty in carrying 2-weeks drug supply home, they may come in to the clinic for weekly drug supply, and the status check performed by the phone calls done at this brief clinic visit.

REVISED TEXT

**Procedure**	**Day -2**	**Day -1**	**Day 1**	**Day 4**	**Day 7**	**Day 14**	**Day 21**	**Day 28**	**Day 35**	**Day 41**	**Day 42**	**Day 43**	**Follow-up**
**Visit Timing (relative to Day 1)**													**Day 56**
Clinic Visit		--In-clinic---			X	X		X			--In-clinic---		X
Phone Contact				X			X[1]		X[1]				
Brief Physical (including thyroid examination in Part B only)	X									X			X
Suicidality monitoring with C-SSRS (Part B only)	X									X			X
Vital Signs		X	X		X	X		X			X	X	X
12-Lead ECG Triplicate		X											
12-Lead ECG Single											X		X
Body Weight (in clinic)		X	X		X	X		X			X	X	X
Waist circumference		X			X	X		X			X		
Urine drug/alcohol screen	X				X	X		X		X			
Hematology/Chemistry/Urinalysis		X			X	X		X			X		X
HbA1c		X									X		
T3/T4/TSH		X									X		
Urine beta-HCG (women)	X												
Liraglutide subjects: continue liraglutide (Part B)							---X---						
Restart Metformin (Part B)												X	
Metformin subjects: continue metformin (Part C)								---X---					
Daily fasting **and pre-dinner** glucometer readings at home								---X---					
Daily fasting weight at home								---X---					
Standardized evening meal	X									X			
Standardized breakfast, lunch and dinner		X									X		
Food Frequency Questionnaire	X												
GI Symptom Questionnaire (GSRS)	X				X	X		X		X			
Hunger Questionnaire		X									X		
Background Questions/ End of Study Questions	X									X			
~~NAFLD Score~~		~~X~~									~~X~~		
CRP, Lipid Panel		X									X		
Deuterated creatine dose (in afternoon**, if available**)	X									X			
Morning urine for deuterated-creatine analytes **(if**			X									X	

**Procedure**	**Day -2**	**Day -1**	**Day 1**	**Day 4**	**Day 7**	**Day 14**	**Day 21**	**Day 28**	**Day 35**	**Day 41**	**Day 42**	**Day 43**	**Follow-up**
**deuterated creatine dose administered)**													
**Urine for background deuterated-creatine (prior to second deuterated creatine dose, if deuterated creatine dose administered)**										**X**			
25 Hydroxy Vitamin D		X											
Lipase and Amylase (Part B only)		X									X		X
PK Blood sampling		X									X		
Glucose/Insulin Blood Sampling (See Section 4.7.6)		X									X		
Fasting Biomarker Samples		X									X		
Drug Metabolism~~10 (DM10)~~ Sampling – blood		X									X		
24-hour urine for assessment of GSK2890457 metabolites		X									X		
DEXA	X									X			
Fecal Sampling	X									X			
Dispense/Instruct: Study Medication		X			X	X		X					
Dispense/ Instruct/Reinforce: Mindful Eating Guide to losing weight		X				X		X					
Randomization			X										
Twice-Daily Dosing of Study Medication			-------------------------------X-------------------------------										
Concomitant Medication Review	X				X	X		X		X			X
AE Assessment	X	X	X	X	X	X		X		X	X	X	X
Subject Returns Telemonitoring Equipment													X

1. If subjects have difficulty in carrying 2-weeks drug supply home, they may come in to the clinic for weekly drug supply, and the status check performed by the phone calls done at this brief clinic visit.

Section 4.7.5 Part A: Day 1 and Day 42: Sampling Schedule of Metformin PK Timepoints

PREVIOUS TEXT

Timepoint	Pharmacokinetic Sample[1] (metformin)
Fasting	X (Pre-metformin on Day 1 and predose on Day 42 within 15 minutes of dose)
Time 0 minutes	Dose metformin alone (Day 1) or with GSK2890457 (Day 42)
Begin eating breakfast immediately after taking study medication(s) and finish eating in 15min[1]	
15minutes	X
30 minutes	X
1 hour	X
1.5 hours	
2 hours	X
4 hours (Pre-Lunch)	X
4 hours	Eat Lunch
5.5 hours	X
6 hours	
8 hours	X
10 hours (Pre-Dinner)	X
10 hours (Pre-Dinner)	GSK2890457 Dose

1. See Section 7.5 for instructions for PK samples. Samples for analysis of metformin will be collected in EDTA containing tubes.
2. Subjects should make their best effort to consume dose and meal in the allotted time; if unable to complete in time, this will not be considered a protocol violation.

REVISED TEXT

Timepoint	Pharmacokinetic Sample[1] (metformin)
Fasting	X<br>(Pre-metformin on Day 1 and predose on Day 42 within 15 minutes of dose)
Time 0 minutes	Dose metformin alone (Day 1) or with GSK2890457 (Day 42)
Begin eating breakfast immediately after taking study medication(s) and finish eating in 15min[2]	
15minutes	X
30 minutes	X
1 hour	X
1.5 hours	
2 hours	X
4 hours (Pre-Lunch)	X
4 hours	Eat Lunch
5.5 hours	X
6 hours	
8 hours	X
10 hours (Pre-Dinner)	X
10 hours (Pre-Dinner)	GSK2890457 Dose

1. See Section 7.5 for instructions for PK samples. Samples for analysis of metformin will be collected in EDTA containing tubes.
2. Subjects should make their best effort to consume dose and meal in the allotted time; if unable to complete in time, this will not be considered a protocol violation.

Section 4.7.6 Parts B and C: Day -1 and Day 42: Sampling Schedule for Glucose, Insulin, Biomarkers, and PK; Satiety Questionnaire Timepoints

PREVIOUS TEXT

Timepoint	Glucose and Insulin Sample	Other Biomarker Samples	Pharmacokinetic Sample[1] (metformin, liraglutide)	Fasting Exploratory Biomarker Samples	Blood for DM10 and GSK2890457 component/metabolites[2] Day -1 and Day 42	Hunger Questionnaire	Urine for DM10 Day -1 and Day 42[3]
Fasting	X[4]						
Fasting	X[4]	X	X (predose on Day 42 within 15 minutes of dose)	X	X (pre-dose)	X	
Time 0 minutes: Dose liraglutide, metformin alone (Day -1) or with GSK2890457 (Day 42)							Pool 0-12hr
Begin eating breakfast immediately after taking study medication(s) and finishing eating in 15min[5]							
15minutes			X		X		
30 minutes	X	X	X		X		
1 hour	X	X	X		X	X	
1.5 hours	X	X					
2 hours	X	X	X		X	X	
4 hours (Pre-Lunch)	X	X	X		X	X	
4 hours			Eat Lunch				
5.5 hours	X	X	X		X		
6 hours						X	
8 hours			X		X	X	
10 hours (Pre-Dinner)	X	X	X		X		Pool 12-24hr
10 hours	Take second dose of metformin (if subject is taking immediate release BID) alone (Day -1) or with GSK2890457 (Day 42) and then eat dinner/evening meal						
11.5 hours	X	X	X		X	X	
12 hours							
14 (bedtime)	X	X					
24 hours	X	X	X (predose, morning dose)				

1. See Section 7.5 for instructions for PK samples. As some of the components of GSK2890457 are unstable in plasma, the blood for GSK2890457 PK needs to be collected in special tubes containing Potassium Oxalate/sodium fluoride and the blood quickly processed as described in Section 7.5. Samples for analysis of metformin or liraglutide will be

collected in EDTA containing tubes.

2. A single sample will be collected at each timepoint and quickly processed as described in Section 7.5.2.
3. Urine will be collected at the various intervals and the volume will be determined. A 100mL aliquot from each time interval will be used for the investigation of GSK2890457 metabolites.
4. Two fasting samples 5min apart will be taken for insulin. Baseline insulin level will be the average of the 2 fasting samples.
5. Subjects should make their best effort to consume dose and meal in the allotted time; if unable to complete in time, this will not be considered a protocol violation.

REVISED TEXT

Timepoint	Glucose and Insulin Sample	Other Biomarker Samples	Pharmacokinetic Sample[1] (metformin, liraglutide)	Fasting Exploratory Biomarker Samples	Blood for ~~DM10 and~~ GSK2890457 components/metabolites[2] Day -1 and Day 42	Hunger Questionnaire	Urine for ~~DM10~~ GSK2890457 components/ metabolites Day -1 and Day 42[3]
Fasting	X[4]						
Fasting	X[4]	X	X (predose on Day 42 within 15 minutes of dose)	X	X (pre-dose)	X	
Time 0 minutes: Dose liraglutide, metformin alone (Day -1) or with GSK2890457 (Day 42)							Pool 0-12hr
Begin eating breakfast immediately after taking study medication(s) and finishing eating in 15min[5]							
15minutes			X		X		
30 minutes	X	X	X		X		
1 hour	X	X	X		X	X	
1.5 hours	X	X					
2 hours	X	X	X		X	X	
4 hours (Pre-Lunch)	X	X	X		X	X	
4 hours	Eat Lunch						
5.5 hours	X	X	X		X		
6 hours						X	
8 hours			X		X	X	
10 hours (Pre-Dinner)	X	X	X		X		
10 hours	Take second dose of metformin (if subject is taking immediate release BID) alone (Day -1) or with GSK2890457 (Day 42) and then eat dinner/evening meal						

Timepoint	Glucose and Insulin Sample	Other Biomarker Samples	Pharmacokinetic Sample[1] (metformin, liraglutide)	Fasting Exploratory Biomarker Samples	Blood for ~~DM10 and~~ GSK2890457 components/metabolites[2] Day -1 and Day 42	Hunger Questionnaire	Urine for ~~DM10~~ GSK2890457 components/ metabolites Day -1 and Day 42[3]
**Fasting**	**X[4]**						
**Fasting**	**X[4]**	**X**	**X (predose on Day 42 within 15 minutes of dose)**	**X**	**X (pre-dose)**	**X**	
11.5 hours	X	X	X		X	X	
12 hours							Pool 12-24hr
14 (bedtime)	X	X					
24 hours	X	X	X (predose, morning dose)				

1. See Section 7.5 for instructions for PK samples~~. As some of the components of GSK2890457 are unstable in plasma, the blood for GSK2890457 PK needs to be collected in special tubes containing Potassium Oxalate/sodium fluoride and the blood quickly processed as described in Section 7.5.~~ Samples for analysis of metformin or liraglutide will be collected in EDTA containing tubes.
2. A single sample will be collected at each timepoint and quickly processed as described in Section 7.5.2. **As some of the components of GSK2890457 are unstable in plasma, the blood for GSK2890457 PK needs to be collected in special tubes containing Potassium Oxalate/sodium fluoride and the blood quickly processed as described in Section 7.5.**
3. Urine will be collected at the various intervals and the volume will be determined. A 100mL aliquot from each time interval will be used for the investigation of GSK2890457 metabolites.
4. Two fasting samples 5min apart will be taken for insulin. Baseline insulin level will be the average of the 2 fasting samples.
5. Subjects should make their best effort to consume dose and meal in the allotted time; if unable to complete in time, this will not be considered a protocol violation.

Section 5.1.1 Screening Inclusion Criteria for Part A (Healthy Subjects)

ADDED TEXT

5. Female Subjects

- Females who are > 3 months postpartum and who have undergone **one of the following** surgical sterilization procedure**s** are eligible to participate:
    - **A documented hysterectomy, or**
    - **A documented bilateral oophorectomy, or**
    - **A documented tubal ligation.**

Section 5.1.2 Screening Exclusion Criteria for Part A (Healthy Subjects), Criteria 1 and 4

ADDED TEXT

1. Disease

- **History of sensitivity to environmental dusts or allergens (including hayfever), unless of mild severity in the opinion of the investigator and approved by the GSK Medical Monitor.**
- **History of asthma and/or use of inhaled bronchodilators or steroids.**

...

4. Substance Abuse

- A positive urine drug **of abuse** screen at Screening.

Section 5.1.2 Screening Exclusion Criteria for Part A (Healthy Subjects), Criteria 7

PREVIOUS TEXT

7. Clinical Study/Experimental Medication Participation

- Where participation in the study would result in donation or blood or blood products in excess of 500mL within a 56-day period.

REVISED TEXT

7. Clinical Study/Experimental Medication Participation

- Where **donation of blood or blood products pre-study and** participation in the study would result ~~in donation or blood or blood products~~ in excess of 500mL **blood depletion** within a 56-day period.

Section 5.1.3 Screening Inclusion Criteria for Type 2 Diabetic Subjects (Parts B and C), Criteria 4 and 5

ADDED TEXT

4. General Health

   - QTcF < 450msec; or QTc**F** < 480msec in subjects with right Bundle Branch Block…

5. Female Subjects

   - **Females who are >3 months postpartum and who have undergone documented bilateral oophorectomy are eligible to participate.**

Section 5.1.3 Screening Inclusion Criteria for Type 2 Diabetic Subjects (Parts B and C), Criteria 8 and 9

ADDED TEXT

8. For Part B **only:** Subjects must be willing to discontinue metformin and replace it with daily liraglutide administered by subcutaneous injection during the twelve-week Stabilization Period and the six-week Treatment Period. In addition, they must meet label recommendations for liraglutide (Victoza), including:

9. **For Part B only:** No personal history or family history of medullary **or papillary** thyroid carcinoma or Multiple Endocrine Neoplasia syndrome type 2.

Specific information regarding warnings, precautions, contraindications, adverse events, and other pertinent information on the GSK investigational product or other study treatment that may impact subject eligibility is provided in the Investigator Brochure for GSK2890457, and Prescribing Information for liraglutide and metformin [**VICTOZA** 2012 Prescribing Information and Medication Guide**, GLUCOPHAGE** 2009 Prescribing Information**]**.

Section 5.1.4 Screening Exclusion Criteria for Type 2 Diabetic Subjects (Parts B and C), Criteria 1 and 2

ADDED TEXT

1. Disease

   - **History of sensitivity to environmental dusts or allergens (including hayfever), unless of mild severity in the opinion of the investigator and approved by the GSK Medical Monitor.**
   - **History of asthma and/or use of inhaled bronchodilators or steroids.**

2. Hypoglycemia unawareness

- **T2D subjects are excluded if, in the opinion of the investigator, they have significant hypoglycaemia unawareness (for example, no symptoms of hypoglycaemia when the blood glucose level is <60mg/dL.**

DELETED TEXT

8. Nicotine Use

- Unwilling to stop smoking~~, **including nicotine patch use**~~, while in the clinic.

Section 5.1.4 Screening Exclusion Criteria for Type 2 Diabetic Subjects (Parts B and C), Criteria 10

PREVIOUS TEXT

- Where participation in the study would result in donation of blood or blood products in excess of 500mL within a 56-day period.

REVISED TEXT

- Where **donation of blood or blood products pre-study and** participation in the study would result in ~~donation of blood or blood products in~~ excess of 500mL **blood depletion** within a 56-day period.

Section 5.2 Screen and Run-in Failures

ADDED TEXT

Data for screen and run-in failures will be collected in source documentation at the site but will not be transmitted to GSK **via InForm. However, some data for screen failures is captured in RAMOS.**

Section 6.3.2.5 Exploratory Analyses

ADDED TEXT

Skeletal muscle mass (kg) will be estimated from the enrichment of deuterated creatinine in urine **if the deuterated creatine has been dosed…**

… DEXA appendicular lean mass will be compared to the deuterated creatine method for muscle mass using Bland-Altman methodology [Bland, 1986] **if the deuterated creatine has been dosed.**

Section 6.3.2.6 Pharmacodynamic/Biomarker Analyses (Part A will include a subset of these)

PREVIOUS TEXT

- o DEXA measurement of fat and skeletal muscle mass on Day 42 as compared to Day -1.

REVISED TEXT

- DEXA measurement of fat and skeletal muscle mass on Day **41** as compared to Day **-2**.

Section 6.3.2.7 Novel and Other Biomarker Analyses in Parts B and C

ADDED TEXT

Exploratory analyses relating to biomarkers of obesity, T2D and/or related metabolic diseases **and/or inflammatory diseases or conditions**, and other biomarkers of GSK2890457 PD or safety may be performed using (i) small molecular weight metabolites, (ii) blood polypeptide analytes, and, (iii) novel biomarkers derived from peptidomic, lipidomic and metabolomic analysis of blood and/or urine

Section 7.3 Physical Exam

PREVIOUS TEXT

For females of NCBP, best efforts should be made to obtain records to document hysterectomy (Parts A, B, and C) or tubal ligation (Part A).

REVISED TEXT

For females of NCBP, best efforts should be made to obtain records to document ~~hysterectomy~~ bilateral oophorectomy (Parts A, B, and C) **or hysterectomy** or tubal ligation (Part A).

Section 7.3.2 Electrocardiogram (ECG)

ADDED TEXT

Unless otherwise stated, single 12-lead ECGs will be obtained at each timepoint during the study using an ECG machine that automatically calculates the heart rate and measures PR, QRS, QT, and QTc**F** intervals. Refer to Section 4.6.1.2 for QTc**F** withdrawal criteria and additional QTc**F** readings that may be necessary.

Section 7.3.5 Clinical Laboratory Assessments, Clinical Chemistry

ADDED TEXT

Clinical Chemistry

BUN	Potassium	AST (SGOT)	Total and direct bilirubin
Creatinine	Chloride	ALT (SGPT)	Uric Acid
Glucose, fasting	Total CO_2	GGT	Albumin
Sodium	Calcium	Alkaline phosphatase	Total Protein
Magnesium	Lipase (Part B only)	Amylase (Part B only)	Phosphate
T3/T4/TSH (Treatment Period of Parts B and C only)	25 Hydroxy Vitamin D at Day -1, Parts B and C only	High sensitivity CRP (Treatment Period of Parts B and C only)	Leptin (Treatment Period of Parts B and C only)

HDL cholesterol (Treatment Period of Parts B and C only)	LDL cholesterol (Treatment Period of Parts B and C only)	Fasting triglycerides (Treatment Period of Parts B and C only)	Insulin, fasting
**HbA1c (Parts B and C only)**			
**Alcohol and drug screen (to include at minimum: amphetamines, barbiturates, cocaine, opiates, cannabinoids and benzodiazepines)**			

Routine Urinalysis

Specific gravity
pH, glucose, protein, blood and ketones by dipstick
Microscopic examination (if blood or protein is abnormal)
**Urine beta-HCG (women) as indicated in Section 4.7**

Section 7.3.5 Clinical Laboratory Assessments, Additional tests conducted at Screening

DELETED TEXT

Additional tests conducted at Screening

HIV
Hepatitis B (HBsAg)
Hepatitis C (Hep C antibody -- if second generation Hepatitis C antibody positive, a hepatitis C antibody Chiron RIBA immunoblot assay (or other third generation immunoassay) should be reflexively performed **on the same sample** to confirm the result)
Urine albumin and creatinine (Parts B and C)
Calcitonin (Part B only)
FSH and estradiol (as needed in women of non-child bearing potential only)
~~Alcohol and drug screen (to include at minimum: amphetamines, barbiturates, cocaine, opiates, cannabinoids and benzodiazepines)~~
~~TSH~~

Section 7.3.6 Re-Screening [NEW SECTION]

Subjects who have fulfilled the Inclusion/Exclusion criteria at Screening, but do not start the Treatment period in Part A or the Stabilization period in Parts B or C within 28 days, may be re-screened within 2 months of the initial screening visit using the following abbreviated procedures:

- **Medical history updated from the last Screening visit**
- **Concomitant medications updated from the last Screening visit**
- **Vital signs**

- **Hematology, clinical chemistry and urinalysis to be tested at Re-Screening are listed below:**

Hematology

Platelet Count	*RBC Indices*:	*Automated WBC Differential:*
RBC Count	MCV	Neutrophils
WBC Count (absolute)	MCH	Lymphocytes
Reticulocyte Count	MCHC	Monocytes
Hemoglobin		Eosinophils
Hematocrit		Basophils

Clinical Chemistry

BUN	Potassium	AST (SGOT)	Total and direct bilirubin
Creatinine	Chloride	ALT (SGPT)	Uric Acid
Glucose, fasting	Total CO_2	GGT	Albumin
Sodium	Calcium	Alkaline phosphatase	Total Protein
Magnesium	Lipase (Part B only)	Amylase (Part B only)	Phosphate
Alcohol and drug screen (to include at minimum: amphetamines, barbiturates, cocaine, opiates, cannabinoids and benzodiazepines)			

Routine Urinalysis

pH, glucose, protein, blood and ketones by dipstick
Microscopic examination (if blood or protein is abnormal)

Section 7.4 Pregnancy

PREVIOUS TEXT

Females of childbearing potential are not eligible to participate so pregnancies are not expected to occur in this study. In the unlikely event that a pregnancy should occur in a female subject, the guidelines outlined in Section 7.4 are to be followed.

REVISED TEXT

Females of childbearing potential are not eligible to participate so pregnancies are not expected to occur in this study. In the unlikely event that a pregnancy should occur in a female subject, **the investigator will attempt to collect pregnancy information following** the guidelines outlined in Section **7.4.2** ~~are to be followed~~.

The investigator will attempt to collect pregnancy information on any female partner of a male study subject who becomes pregnant while participating in this study. When a pregnancy occurs, the guidelines outlined in Section 7.4.2 are to be followed.

Section 7.4.2 Action to be Taken if Pregnancy Occurs

ADDED TEXT

The Investigator will collect pregnancy information on any female subject **and/or female partner of a male subject**, who becomes pregnant while participating in this study. The Investigator will record pregnancy information on the appropriate form and submit it to GSK within 2 weeks of learning of a subject's pregnancy. The **female** subject **or female partner of a male subject** will also be followed to determine the outcome of the pregnancy...

While pregnancy itself is not considered to be an AE or SAE, any pregnancy complication or elective termination of a pregnancy **in a female subject or the female partner of a male subject** for medical reasons will be recorded as an AE or SAE…

… While the Investigator is not obligated to actively seek this information in former **female** study participants **or female partners of male participants**, he or she may learn of an SAE through spontaneous reporting.

Section 7.5.1.1 Blood Samples for Metformin or Liraglutide PK

PREVIOUS TEXT

Transfer resulting plasma into a 1.8mL NUNC tube.

REVISED TEXT

Transfer resulting plasma into a 1.8mL **cryotube (NUNC).**

Section 7.5.1.2 Blood Samples for GSK2890457 PK, Paragraph 2

PREVIOUS TEXT

...Transfer a second aliquot of exactly 700µL plasma into an 2mL amber storage tube containing exactly 700µL 50mM citrate buffer.

REVISED TEXT

...Transfer a second aliquot of exactly **500µL** plasma into an 2mL amber storage tube containing exactly **500µL** 50mM citrate buffer.

Section 7.5.2 Urine Sample Collection

ADDED TEXT

Urine samples for pharmacokinetic analysis of GSK2890457 + metabolites will be collected at the timepoints listed in Section 4.7. The timing of urine samples may be altered and/or samples may be obtained at additional time points, based on emerging data, to ensure thorough PK monitoring. **Details of PK urine sample processing, storage procedures and shipping procedures are detailed in the SPM.**

Section 7.5.2 Urine Sample Collection

PREVIOUS TEXT

Urine samples for pharmacokinetic analysis of GSK2890457 + metabolites will be collected at the timepoints listed in Section 4.7. The timing of urine samples may be altered and/or samples may be obtained at additional time points, based on emerging data, to ensure thorough PK monitoring.

Collect urine in an amber container. Transfer 100mL into a 150mL amber bottle and immediately store at -80ºC.

REVISED TEXT

Urine samples for pharmacokinetic analysis of GSK2890457 + metabolites will be collected at the timepoints listed in Section 4.7. The timing of urine samples may be altered and/or samples may be obtained at additional time points, based on emerging data, to ensure thorough PK monitoring. **Details of PK urine sample processing, storage procedures and shipping procedures are detailed in the SPM**.

On Days -1 and 42, the first urine of the day will be used for urinalysis and any remaining urine from the first void will be discarded. Collect **all subsequent** urine **samples** in an amber container **for each interval**. **The first urine sample voided on Days 1 and 43 will be added to the collection for the 24-hour interval. At the end of each collection interval,** transfer 100mL into a 150mL amber bottle and immediately store at -80ºC.

Section 7.5.3 Sample Analysis

ADDED TEXT

Pharmacokinetic analyses may include quantitation tracer to trace **th**e ratio of deuterium-labeled creatinine to total creatinine by LC-MS, total creatine and creatinine by LC-MS.

Section 7.6.2.1 Other Exploratory Biomarkers

ADDED TEXT

With the subject's consent, blood and/or urine sample(s) will be collected during this study and may be used for the purposes of measuring novel biomarkers to identify factors that may influence T2D, obesity and/or medically related conditions **and/or inflammatory diseases or conditions**, as well as the biological and clinical responses to GSK2890457. If relevant, this approach will be extended to include the identification of biomarkers associated with adverse events.

Section 7.6.2.2 Metabolomic Research

ADDED TEXT

This may include analysis of identified or uncharacterized metabolites and lipids that are known to be or emerge in the future as being important in the pathogenesis of T2D, obesity and/or related metabolic disorders **and/or inflammatory diseases or conditions, and/or** the subject's response to GSK2890457 or adverse events.

Section 7.6.2.3 Proteome/Peptide Research [NEW SECTION]

Plasma proteome studies may be performed by 2-D gel separation, and/or peptide mass mapping, or an alternative equivalent procedure. Proprietary algorithms and standard statistical techniques, such as ANOVA and ANCOVA, may be used to identify individual proteins exhibiting statistically acceptable changes in their levels between samples, and between groups of samples. These differentially expressed proteins will be identified by mass spectrometry or equivalent technology. This will enable the evaluation of changes in proteome profiles that may correlate with biological response relating to type 2 diabetes mellitus, obesity and the metabolic syndrome and/or related medical conditions and/or inflammatory diseases or conditions, and/or the biological and clinical responses to GSK2890457 or adverse events.

The same samples may also be used to confirm findings by application of alternative technologies.

7.6.3.1 Blood Sample Collection for Glucose and Insulin, Paragraph 2

ADDED TEXT

Immediately after collection, gently invert (DO NOT SHAKE) the evacuated blood collection tube 8-10 times to mix the EDTA anticoagulant with the whole blood and place the sample(s) on **crushed** ice or in a refrigerator. **Within 1 hour of sample collection, separate the plasma by refrigerated (4°C) centrifugation at 1,500 to 2,000 x g for a minimum of 10 minutes. Transfer resulting plasma into 1.8mL cryotubes (NUNC); do not overfill. Store in freezer at approximately -80°C.**

7.6.3.2 Blood Sample Collection for Other Biomarkers, Paragraph 2

ADDED TEXT

Immediately after collection, gently invert (DO NOT SHAKE) the evacuated blood collection tube 8-10 times to mix the EDTA anticoagulant with the whole blood and place the sample(s) on **crushed** ice or in a refrigerator. **Within 1 hour of sample collection, separate the plasma by refrigerated (4° C) centrifugation at 1,500 to 2,000 x g for a minimum of 10 minutes. Transfer resulting plasma into 1.8mL cryotubes (NUNC); do not overfill. Store in freezer at approximately -80ºC.**

7.6.3.3 Blood Sample Collection for Fasting Exploratory Biomarkers, Paragraph 2

ADDED TEXT

Immediately after collection, gently invert (DO NOT SHAKE) the evacuated blood collection tube 8-10 times to mix the EDTA anticoagulant with the whole blood and place the sample(s) on crushed ice or in a refrigerator. Within 1 hour of sample collection, separate the plasma by refrigerated (4° C) centrifugation at 1,500 to 2,000 x g for a minimum of 10 minutes. Transfer resulting plasma into 1.8mL cryotubes (NUNC); do not overfill. Store in freezer at approximately -80ºC.

7.6.4 Deuterated Creatine Method for Measuring Total Skeletal Muscle Mass

ADDED TEXT

If available prior to the Treatment period, a capsule containing 30mg of deuterated creatine will be taken with ~200mL of water 2h after lunch in the clinic on Days -2 and 41 at the timepoints listed in Section 4.7.

On Days 1 and 43, a fasting morning urine sample, but not the first void, will be collected within four hours of waking, approximately 38 – 46 hrs after the deuterated-creatine dose **(if the deuterated creatine has been dosed)**. Urine collection will be in 2 tubes: 1 tube (1.4mL Matrix TrakMate) for a 500uL urine sample, labelled for deuterated-creatinine/unlabelled creatinine, and a second tube (1.4mL Matrix TrakMate) for a 500uL urine sample for assay of unlabeled creatine.

In addition, on Day 41 prior to second dose of deuterated-creatine a urine sample will be collected for assessment of background deuterated-creatinine **if deuterated creatine has been dosed**.

The time of day for dosing and time of day for urine collections will be the same at baseline and end of the 6-week Treatment Period.

If deuterated creatine has been dosed, urine samples for pharmacokinetic analysis of deuterium labelled creatinine, total creatine, and total creatinine, will be collected at the timepoints listed in Section 4.7. The timing of urine samples may be altered and/or samples may be obtained at additional time points to ensure thorough PK monitoring.

8.2.1 Standard Test Meals, Part A, Paragraph 1

ADDED TEXT

...The breakfast meal should contain approximately 400**-500**kcal....

8.2.1 Standard Test Meals, Parts B and C, Paragraph 2

ADDED TEXT

On Days -1 and 42 in Part B and C, the breakfast meal will constitute a 'meal tolerance test' and should contain approximately 400**-500**kcal (unless modified based on emergent data from Part A). **The breakfast meal composition will be the same on Days -1 and 42.**

10.2.1 Parts B and C Glucose Withdrawal Criteria, Third Bullet

PREVIOUS TEXT

- If fasting glucose levels are >270mg/dL on 3 consecutive days, the subject will be withdrawn and appropriate anti-diabetic therapy reinstated, unless, in the judgment of the Investigator and GSK Medical Monitor, there are circumstances that indicate that the high fasting glucose levels are temporary and would not compromise subject safety and wellbeing or study objectives and conclusions.

REVISED TEXT

- If fasting **CBG/blood** glucose levels are >270mg/dL on 3 consecutive days, the subject will be withdrawn and appropriate anti-diabetic therapy reinstated, unless, in the judgment of the Investigator and GSK Medical Monitor, **the subject is well and** there are circumstances that indicate that the high **CBG/blood** glucose levels are temporary and would not compromise subject safety and wellbeing or study objectives and conclusions.

REFERENCES

ADDED

GlaxoSmithKline Document Number 2012N141885_00 Study ID GSK2890457 Investigator's Brochure. Report Date 24-Aug-2012

Appendix 5: Directions for Taking Study Medicine, Paragraph 1

ADDED TEXT

Each kit contains:

- 3 foil packets which contain powders of different color

Appendix 5: Directions for Taking Study Medicine, Mixing Instructions

ADDED TEXT

Mixing Instructions:...

- **Plastic bag to collect the empty packets and seals (you will need to bring the empty packets and empty capsule bottles to the clinic for the study staff to check).„**

3) **Powders can cause nose, throat and eye irritation in sensitive people. When you take the study medicine, you will be dissolving 3 different powders in water and these instructions will minimize the risk of irritation for you and people around you:**

   a) **Choose a preparation area close to a sink so that you can wipe the surface easily after you have finished.**

   b) **Don't have other people or children around you when you are preparing the drink because they might distract you or they might be sensitive to powders.**

   c) **Have a plastic bag nearby so that you can easily dispose of empty packets. Keep a damp paper towel close by to wipe up after you have finished making the drink.**

   d) **The foil packets are rectangular. Hold each foil packet vertically along one of the shorter edges and** tap **the** packet **gently** to get the contents to **the bottom**. Using scissors, **one at a time** cut **right across** the top of each foil packet, **making sure that you cut the packet just below the seal**. Empty the contents **gently** into the water. **With the opening of the packet over the bottle**, tap **the bottom** of the packet **gently** to empty **it** as completely as possible, **but DO NOT shake it vigorously as this can make a dust cloud.** Some particles may still cling to the inside of the packet, and this is ok. **Put the packet in the plastic bag before you start the next one. You should do this one packet at a time to avoid spilling the powder, and you want to make sure to get the full dose into the water.**

   e) When you are taking multiple kits at the same time, empty all of the packets from all of the kits you are dosing into the bottle. (The number of kits is determined by the scheduled dose; the study site will provide this schedule to you.)

   f) Screw the cap on tightly and make sure the white snap-cover is tightly closed (push until you hear the "snap" – the mixture may stain if spilled). **Place a finger over the cap and** then **shake the bottle until well mixed (about 10 seconds)**. **This is important.**

   g) **If you find that the mixture is too thick for you, you can add more water AFTER the powders have been dissolved. Do not add more water while there are powders sitting on the water because this can cause dust to form.**

   h) **Wipe the preparation surface with a damp paper towel when you have finished.**

4) Drink all of the contents within 15 minutes of mixing. **You may notice some lumps as you drink, that is ok.** When done, add a little more water (2 or more ounces) to the container. Once again, screw the cap tightly and make sure the white snap-cover is tightly closed. **Place a finger over the cap and** s**hake well** and drink as before to get as much of the medicine as possible. Some bits may stick to the inside of the bottle, and this is ok.

5) **After this**, take the capsules from the kit(s) with **8 ounces** of plain water. Be sure to take one capsule at a time. Also make sure that you take a drink of **water** before and after each capsule so that it is swallowed properly. **This will clean your mouth of the color from the powder drink.**

6) Wash the Blender bottle and the wire mixing ball with soap and hot water immediately to clean before next use. **You may need to use a brush to clean the cap and the snap-on lid.** Dried spots are more difficult to remove. Rinse well. Always be sure that **you put** the wire mixing ball back in the bottle for the next dose.

Important note: Some subjects may find it difficult to take GSK2890457, especially at the highest dose of 40g. These instructions may be modified to make it easier for you to take the study medicine. Please contact the study coordinator for alternatives that may make it easier to take GSK2890457. Once you mix up the powders with the water, you must take all of it at that time.

If you have found a simple way to make it easier to take the study medicine, please let the study coordinator know as it may help other subjects in the study.

Make sure you keep well hydrated during the day by drinking water, rather than sugared sodas, flavoured drinks or fruit juices.

<u>Frequent Questions:</u>

1) **My teeth and tongue are purple! Is this OK?**

a) The drink will be purple to dark red-purple in color, and your mouth and teeth may look purple after drinking. This is not harmful. **Drinking the water to take the capsules or** brushing your teeth **will make** the color go away. If your fingers turn purple from handling the powder, the color will come off with soap and water.

2) **I'm thirsty after I drink this medicine. Is this OK?**

a) Yes. Some people are thirsty when they are taking this medicine (this is not harmful). You can drink extra water, but as usual, you shouldn't drink high-calorie drinks like sugared-sodas or fruit juice.

3) **What if I skip breakfast some days, or I'm not hungry?**

a) It's fine to take your study medicine without food. **Make sure you drink water to keep hydrated.** Some people find they have less of an appetite after drinking the medicine. Just make sure that the morning and evening doses are spaced out during the day (i.e., approximately breakfast time and dinner time).

4) **What if I miss a dose? Or if some of the dose spills?**

a) If you miss a dose, and it is **less than** 3 hours since you were supposed to take it, go ahead and take the dose. If it is more than 3 hours since the dose you missed, skip that dose and take the next dose at the regularly scheduled time. Do not take 2 doses at the same time. Be sure to tell the study coordinator about missed doses or partial doses at your next visit so we can keep track of what you actually take. Also, be sure to tell the study coordinator if you were unable to take some parts of the study medicine.

5) **I normally take my metformin with a meal, but sometimes I do not feel like eating after taking the study medicine. What shall I do?**

   a) It is OK to take the metformin **with water** after taking the study medicine, even though you have not eaten food.

   b) Tell the study staff if taking the metformin like this makes you feel unwell. They will help you find an alternative way of taking the study medicine or the metformin.

6) **What should I do if I have irritation of the nose, throat or eyes?**

   a) If this happens because of a large spill (for example, you have accidentally dropped open packets)

      i) Move away from the preparation area and keep other people away.

      ii) Wash your hands with soap and water. Then wash you face and eyes with plenty of water.

      iii) Place the mask provided over your nose and mouth and then go to clean up the area with paper wipes that have been dampened with water. Continue cleaning until there are no more powders in or around the spillage area.

      iv) Next time you visit the clinical unit, tell the study nurse that this happened. You will be asked how much of the study medicine was lost.

   b) If this happens while you are preparing the drink normally

      i) Make sure that you are not creating dust clouds by shaking the open packets or bottle. Handle the open packet gently and don't squeeze it while it has powder inside. Immediately put each empty packet and its seal into a plastic bag.

      ii) Next time you visit the clinical unit, tell the study nurse. The nurse will check that you are mixing the powders in the drink properly.

      iii) If simple adjustments solve the irritation problem, you can continue making up the drink without extra precautions. If not, you will be told to wear a mask, and possibly eye glasses, while preparing the drink.
