# Supplementary material for: Weight and Glucose Reduction Observed with a Combination of Nutritional Agents in Rodent Models Does Not Translate to Humans in a Randomized Clinical Trial with Healthy Volunteers and Subjects with Type 2 Diabetes
Source: PLoS One. 2016 Apr 19;11(4):e0153151. doi: 10.1371/journal.pone.0153151 (PMC4836696; doi:10.1371/journal.pone.0153151)
Supplement: S1 Table — (DOCX) [file pone.0153151.s022.docx]

## S1 Table. Serum Chemistry Parameters in DIO Mice Treated with GSK457 ± Exendin-4 AlbudAb
