# Supplementary material for: Weight and Glucose Reduction Observed with a Combination of Nutritional Agents in Rodent Models Does Not Translate to Humans in a Randomized Clinical Trial with Healthy Volunteers and Subjects with Type 2 Diabetes
Source: PLoS One. 2016 Apr 19;11(4):e0153151. doi: 10.1371/journal.pone.0153151 (PMC4836696; doi:10.1371/journal.pone.0153151)
Supplement: S5 Table — (DOCX) [file pone.0153151.s026.docx]

## S5 Table. Results of the ANCOVA of Change from Baseline Weighted Mean Glucose and Fasting Plasma Glucose – Clinical Study Part B (Subjects with T2D taking Liraglutide)

| **Parameter** | |  | **Placebo (N=6)** | **GSK457**  **(N=14)** |
| --- | --- | --- | --- | --- |
|  |  | n^1^ | 6 | 13 |
| 24-hourAUC Weighted Mean Glucose (mmol/L) | Baseline | Mean | 9.177 | 9.676 |
|  |  | SD | 2.3153 | 2.1487 |
|  | Day 42 | Mean | 8.638 | 8.673 |
|  |  | SD | 1.7047 | 2.0855 |
|  | Change from Baseline | Mean | −0.540 | −1.002 |
|  |  | SD | 1.2751 | 0.9869 |
|  | Model−Adjusted Change^2^ | Mean | −0.613 | −0.968 |
|  |  | SE | 0.410 | 0.278 |
|  | Difference from Placebo^2^ | Mean | − | −0.356 |
|  |  | 95% CI |  | (−1.409, 0.698) |
| 4-hour Post−Breakfast AUC Weighted Mean Glucose (mmol/L) | Baseline | Mean | 8.546 | 9.813 |
|  |  | SD | 1.9817 | 2.3234 |
|  | Day 42 | Mean | 8.833 | 9.525 |
|  |  | SD | 1.9538 | 2.0822 |
|  | Change from Baseline | Mean | 0.288 | −0.288 |
|  |  | SD | 0.7622 | 1.7382 |
|  | Model−Adjusted Change^2^ | Mean | 0.018 | −0.164 |
|  |  | SE | 0.583 | 0.390 |
|  | Difference from Placebo^2^ | Mean | − | −0.182 |
|  |  | 95% CI |  | (-1.694, 1.331) |
| Fasting Glucose (mmol/L) | Baseline | Mean | 7.918 | 8.778 |
|  |  | SD | 1.6361 | 2.5911 |
|  | Day 42 | Mean | 7.712 | 8.466 |
|  |  | SD | 1.8956 | 2.173 |
|  | Change from Baseline | Mean | −0.206 | −0.312 |
|  |  | SD | 0.6316 | 1.7244 |
|  | Model−Adjusted Change^2^ | Mean | −0.384 | −0.23 |
|  |  | SE | 0.556 | 0.375 |
|  | Difference from Placebo^2^ | Mean | − | 0.155 |
|  |  | 95% CI |  | (−1.277, 1.587) |

1. Number of subjects with a value at Baseline and at specified visit.

2. Based on ANCOVA performed on change from baseline during the treatment phase. Terms for treatment, and Baseline were included in the model.
