# Supplementary material for: Weight and Glucose Reduction Observed with a Combination of Nutritional Agents in Rodent Models Does Not Translate to Humans in a Randomized Clinical Trial with Healthy Volunteers and Subjects with Type 2 Diabetes
Source: PLoS One. 2016 Apr 19;11(4):e0153151. doi: 10.1371/journal.pone.0153151 (PMC4836696; doi:10.1371/journal.pone.0153151)
Supplement: S6 Table — (DOCX) [file pone.0153151.s027.docx]

## S6 Table. Results of the ANCOVA of Change from Baseline HbA1c − Clinical Study Part B (Subjects with T2D taking Liraglutide)

| **Parameter** | |  | **Placebo**  **(N=6)** | **GSK457**  **(N=14)** |
| --- | --- | --- | --- | --- |
|  |  | n^1^ | 6 | 13 |
| Glycosylated Hemoglobin A1C  (% Total Hemoglobin) | Baseline | Mean | 8.133 | 8.231 |
|  |  | SD | 1.1039 | 0.9384 |
|  | Day 42 | Mean | 7.917 | 7.954 |
|  |  | SD | 1.2592 | 1.0413 |
|  | Change from Baseline | Mean | −0.217 | −0.277 |
|  |  | SD | 0.2787 | 0.4419 |
|  | Model−Adjusted Change^2^ | Mean | −0.214 | −0.278 |
|  |  | SE | 0.167 | 0.113 |
|  | Difference from Placebo^2^ | Mean | − | −0.065 |
|  |  | 95% CI |  | (−0.495, 0.365) |
| Glycosylated Hemoglobin A1C (mmol/mol) | Baseline | Mean | 65.392 | 66.457 |
|  |  | SD | 12.0649 | 10.2560 |
|  | Day 42 | Mean | 63.024 | 63.430 |
|  |  | SD | 13.7622 | 11.3806 |
|  | Change from Baseline | Mean | −2.368 | −3.026 |
|  |  | SD | 3.0458 | 4.8293 |
|  | Model−Adjusted Change^2^ | Mean | −2.334 | −3.042 |
|  |  | SE | 1.832 | 1.244 |
|  | Difference from Placebo^2^ | Mean | − | −0.708 |
|  |  | 95% CI |  | (−5.407, 3.991) |
| 1. Number of subjects with a value at baseline and at specified visit.  2. Based on ANCOVA performed change from baseline during the treatment phase. Terms for treatment, and baseline were included in the model. | | | | |
