# Supplementary material for: Weight and Glucose Reduction Observed with a Combination of Nutritional Agents in Rodent Models Does Not Translate to Humans in a Randomized Clinical Trial with Healthy Volunteers and Subjects with Type 2 Diabetes
Source: PLoS One. 2016 Apr 19;11(4):e0153151. doi: 10.1371/journal.pone.0153151 (PMC4836696; doi:10.1371/journal.pone.0153151)
Supplement: S7 Table — (DOCX) [file pone.0153151.s028.docx]

## S7 Table. Results of ANCOVA of Change and % Change from Baseline In−Clinic Body Weight − Clinical Study Part C (Subjects with T2D taking Metformin)

|  |  | **Placebo**  **(N=6)** | **GSK457 40g**  **(N=12)** |
| --- | --- | --- | --- |
|  | n ^1^ | 6 | 12 |
| Baseline (kg) | Mean | 84.74 | 92.27 |
|  | SD | 10.119 | 14.339 |
| Day 42 (kg) | Mean | 84.28 | 92.68 |
|  | SD | 9.953 | 14.227 |
| Change from Baseline (kg) | Mean | −0.47 | 0.42 |
|  | SD | 1.104 | 1.221 |
| Percent Change from Baseline | Mean | −0.53 | 0.50 |
|  | SD | 1.504 | 1.365 |
| Model−Adjusted Change^2^ (kg) | Mean | −0.60 | 0.48 |
|  | SE | 0.492 | 0.346 |
| Difference from Placebo^2^ (kg) | Mean | − | 1.08 |
|  | 95% CI |  | (−0.20, 2.36) |
| Model−Adjusted Percent Change^2^ | Mean | −0.68 | 0.58 |
|  | SE | 0.573 | 0.404 |
| Percent Change from Placebo^2^ | Mean | − | 1.26 |
|  | 95% CI |  | (−0.24, 2.75) |
| 1. Number of subjects with a value at Baseline and at specified visit.  2. Based on repeated measures analysis of variance performed separately on both change from Baseline and percent change from Baseline during the treatment phase. Terms for treatment, visit, treatment by visit interaction and Baseline were included in the model. Baseline = Average of Day −1 and Day 1. Day 42 = Average of Day 42 and 43. | | | |
