# Supplementary material for: Weight and Glucose Reduction Observed with a Combination of Nutritional Agents in Rodent Models Does Not Translate to Humans in a Randomized Clinical Trial with Healthy Volunteers and Subjects with Type 2 Diabetes
Source: PLoS One. 2016 Apr 19;11(4):e0153151. doi: 10.1371/journal.pone.0153151 (PMC4836696; doi:10.1371/journal.pone.0153151)
Supplement: S8 Table — (DOCX) [file pone.0153151.s029.docx]

## S8 Table. Results of the ANCOVA of Change from Baseline Weighted Mean Glucose − Clinical Study Part C (Subjects with T2D taking Metformin)

|  | | | **Placebo**  **(N=6)** | **GSK457 40g**  **(N=12)** |
| --- | --- | --- | --- | --- |
| 24-Hour AUC Weighted Mean Glucose (mmol/L) |  | n^1^ | 6 | 12 |
|  | Baseline | Mean | 9.064 | 10.627 |
|  |  | SD | 1.6024 | 2.0601 |
|  | Day 42 | Mean | 10.453 | 10.777 |
|  |  | SD | 1.8474 | 2.2978 |
|  | Change from Baseline | Mean | 1.389 | 0.150 |
|  |  | SD | 0.7716 | 1.1337 |
|  | Model−Adjusted Change^2^ | Mean | 1.376 | 0.156 |
|  |  | SE | 0.459 | 0.316 |
|  | Difference from Placebo^2^ | Mean | − | −1.219 |
|  |  | 95% CI |  | (−2.447, 0.009) |
| 4-hour Post−Breakfast AUC Weighted Mean Glucose (mmol/L) | Baseline | Mean | 9.333 | 10.889 |
|  |  | SD | 2.0178 | 2.2461 |
|  | Day 42 | Mean | 10.673 | 11.157 |
|  |  | SD | 1.8104 | 2.3718 |
|  | Change from Baseline | Mean | 1.340 | 0.267 |
|  |  | SD | 0.9309 | 1.3294 |
|  | Model−Adjusted Change^2^ | Mean | 1.194 | 0.341 |
|  |  | SE | 0.518 | 0.359 |
|  | Difference from Placebo^2^ | Mean | − | −0.853 |
|  |  | 95% CI |  | (−2.232, 0.526) |
| Parameter Fasting Glucose (mmol/L) | Baseline | Mean | 8.780 | 10.979 |
|  |  | SD | 1.3466 | 2.2039 |
|  | Day 42 | Mean | 9.136 | 10.482 |
|  |  | SD | 1.4772 | 2.2250 |
|  | Change from Baseline | Mean | 0.356 | −0.497 |
|  |  | SD | 0.8361 | 1.2831 |
|  | Model−Adjusted Change^2^ | Mean | 0.136 | −0.387 |
|  |  | SE | 0.520 | 0.351 |
|  | Difference from Placebo^2^ | Mean | - | −0.524 |
|  |  | 95% CI |  | (−1.939, 0.892) |
| 1. Number of subjects with a value at Baseline and at specified visit.  2. Based on ANCOVA performed change from Baseline during the treatment phase. Terms for treatment, and Baseline were included in the model. | | | | |
