# Supplementary material for: Weight and Glucose Reduction Observed with a Combination of Nutritional Agents in Rodent Models Does Not Translate to Humans in a Randomized Clinical Trial with Healthy Volunteers and Subjects with Type 2 Diabetes
Source: PLoS One. 2016 Apr 19;11(4):e0153151. doi: 10.1371/journal.pone.0153151 (PMC4836696; doi:10.1371/journal.pone.0153151)
Supplement: S9 Table — (DOCX) [file pone.0153151.s030.docx]

## S9 Table. Results of the ANCOVA of Change from Baseline HbA1c − Clinical Study Part C (Subjects with T2D taking Metformin)

| **Parameter** | |  | **Placebo**  **(N=6)** | **GSK457**  **(N=12)** |
| --- | --- | --- | --- | --- |
|  |  | n^1^ | 6 | 12 |
| Glycosylated Hemoglobin A1C  (% Total Hemolobin) | Baseline | Mean | 8.250 | 8.342 |
|  |  | SD | 0.8068 | 0.9615 |
|  | Day 42 | Mean | 8.283 | 8.133 |
|  |  | SD | 1.1107 | 0.8403 |
|  | Change from Baseline | Mean | 0.033 | -0.208 |
|  |  | SD | 0.5820 | 0.7012 |
|  | Model−Adjusted Change^2^ | Mean | 0.018 | -0.201 |
|  |  | SE | 0.264 | 0.187 |
|  | Difference from Placebo^2^ | Mean | − | −0.219 |
|  |  | 95% CI |  | (−0.910, 0.472) |
| Glycosylated Hemoglobin A1C (mmol/mol) | Baseline | Mean | 66.67 | 67.67 |
|  |  | SD | 8.818 | 10.508 |
|  | Day 42 | Mean | 67.03 | 65.39 |
|  |  | SD | 12.139 | 9.183 |
|  | Change from Baseline | Mean | 0.36 | -2.28 |
|  |  | SD | 6.360 | 7.664 |
|  | Model−Adjusted Change^2^ | Mean | 0.20 | -2.20 |
|  |  | SE | 2.892 | 2.044 |
|  | Difference from Placebo^2^ | Mean | − | -2.40 |
|  |  | 95% CI |  | (−9.949, 5.159) |
| 1. Number of subjects with a value at Baseline and at specified visit.  2. Based on ANCOVA performed change from Baseline during the treatment phase. Terms for treatment, and Baseline were included in the model. | | | | |
