# Supplementary material for: Weight and Glucose Reduction Observed with a Combination of Nutritional Agents in Rodent Models Does Not Translate to Humans in a Randomized Clinical Trial with Healthy Volunteers and Subjects with Type 2 Diabetes
Source: PLoS One. 2016 Apr 19;11(4):e0153151. doi: 10.1371/journal.pone.0153151 (PMC4836696; doi:10.1371/journal.pone.0153151)
Supplement: S10 Table — (DOCX) [file pone.0153151.s031.docx]

## S10 Table. Summary of Plasma Metformin Pharmacokinetic Parameters Following Dose Administration in Clinical Study Part A (Healthy Subjects)

| **Treatment** | **Visit** | **n** | **Cmax**  **(ng/mL)** | **Tmax^1^**  **(h)** | **AUC(0−10)**  **(ng.h/mL)** |
| --- | --- | --- | --- | --- | --- |
| **Placebo** | **Day 1** | 4 | 682  (19.0) | 3.01  (2.00−4.03) | 4347  (19.1) |
|  | **Day 42** | 4 | 860  (18.8) | 4.00  (2.00−4.03) | 5082  (21.1) |
| **GSK457** | **Day 1** | 11 | 576  (24.4) | 2.00  (1.00−4.00) | 3403  (26.4) |
|  | **Day 42** | 10 | 374  (31.2) | 2.00  (2.00−5.50) | 2232  (38.6) |
| Data is presented as geometric mean (CV%)  1 Tmax is presented as median (range) | | | | | |
