# Supplementary material for: Weight and Glucose Reduction Observed with a Combination of Nutritional Agents in Rodent Models Does Not Translate to Humans in a Randomized Clinical Trial with Healthy Volunteers and Subjects with Type 2 Diabetes
Source: PLoS One. 2016 Apr 19;11(4):e0153151. doi: 10.1371/journal.pone.0153151 (PMC4836696; doi:10.1371/journal.pone.0153151)
Supplement: S11 Table — (DOCX) [file pone.0153151.s032.docx]

## S11 Table. Statistical Comparison of Plasma Metformin Pharmacokinetic Parameters in Clinical Study Part A (Healthy Subjects)

| **Comparison** | **Ratio of GLS Means [90% CI]** |
| --- | --- |
|  | **Metformin + GSK457 vs metformin alone** |
| **AUC(0−10)** | 0.675 [0.585, 0.779] |
| **Cmax** | 0.66 [0.578, 0.757] |
|  | |
