# Supplementary material for: Weight and Glucose Reduction Observed with a Combination of Nutritional Agents in Rodent Models Does Not Translate to Humans in a Randomized Clinical Trial with Healthy Volunteers and Subjects with Type 2 Diabetes
Source: PLoS One. 2016 Apr 19;11(4):e0153151. doi: 10.1371/journal.pone.0153151 (PMC4836696; doi:10.1371/journal.pone.0153151)
Supplement: S12 Table — (DOCX) [file pone.0153151.s033.docx]

## S12 Table. Summary of Plasma Liraglutide Pharmacokinetic Parameters Following Dose Administration – Clinical Study Part B (Subjects with T2D taking Liraglutide)

| **Treatment** | **Visit** | **n** | **AUC(0−t)^1^**  **(ng.h/mL)** | **Cmax^1^**  **(ng/mL)** | **Tlast^2^**  **(h)** | **Tmax^2^**  **(h)** |
| --- | --- | --- | --- | --- | --- | --- |
| Placebo | Day -1 | 6 | 2210  (74.9) | 120  (66.8) | 23.83  (23.8−24.0) | 9.74  (2.0−11.5) |
|  | Day 42 | 5 | 2505  (99.7) | 129  (86.9) | 23.80  (23.8−24.1) | 9.92  (2.1−11.5) |
| GSK457 | Day -1 | 12 | 1269  (91.8) | 72.2  (70.5) | 23.88  (23.0−25.9) | 8.00  (0.0−11.5) |
|  | Day 42 | 11 | 1266  (79.4) | 70.9  (63.5) | 24.02  (23.8−24.3) | 9.98  (0.8−23.8) |
| 1. Data are presented as geometric mean (CV%)  2. Data are presented as median (range) | | | | | | |
