# Supplementary material for: Weight and Glucose Reduction Observed with a Combination of Nutritional Agents in Rodent Models Does Not Translate to Humans in a Randomized Clinical Trial with Healthy Volunteers and Subjects with Type 2 Diabetes
Source: PLoS One. 2016 Apr 19;11(4):e0153151. doi: 10.1371/journal.pone.0153151 (PMC4836696; doi:10.1371/journal.pone.0153151)
Supplement: S13 Table — (DOCX) [file pone.0153151.s034.docx]

## S13 Table. Statistical Comparison of Plasma Liraglutide Pharmacokinetic Parameters – Clinical Study Part B (Subjects with T2D taking Liraglutide)

| **Comparison** | **Ratio of GLS Means [90% CI]** |
| --- | --- |
|  | **liraglutide + GSK457 vs liraglutide alone** |
| AUC(0−t) | 1.05 [0.729, 1.50] |
| Cmax | 1.03 [0.781, 1.37] |
|  | |
